# Accurate discrimination of the wake-sleep states of mice using non-invasive whole-body plethysmography

**DOI:** 10.1038/srep41698

**Published:** 2017-01-31

**Authors:** Stefano Bastianini, Sara Alvente, Chiara Berteotti, Viviana Lo Martire, Alessandro Silvani, Steven J. Swoap, Alice Valli, Giovanna Zoccoli, Gary Cohen

**Affiliations:** 1 Prism Lab, Department of Biomedical and Neuromotor Sciences, University of Bologna, Bologna, I-40126, Italy; 2 Department of Biology, Williams College, Williamstown, Massachusetts, MA 01267, USA; 3 Department of Women’s and Children’s Health, Neonatal Unit, Karolinska Institutet, Stockholm, 17176, Sweden

## Abstract

A major limitation in the study of sleep breathing disorders in mouse models of pathology is the need to combine whole-body plethysmography (WBP) to measure respiration with electroencephalography/electromyography (EEG/EMG) to discriminate wake-sleep states. However, murine wake-sleep states may be discriminated from breathing and body movements registered by the WBP signal alone. Our goal was to compare the EEG/EMG-based and the WBP-based scoring of wake-sleep states of mice, and provide formal guidelines for the latter. EEG, EMG, blood pressure and WBP signals were simultaneously recorded from 20 mice. Wake-sleep states were scored based either on EEG/EMG or on WBP signals and sleep-dependent respiratory and cardiovascular estimates were calculated. We found that the overall agreement between the 2 methods was 90%, with a high Cohen’s Kappa index (0.82). The inter-rater agreement between 2 experts and between 1 expert and 1 naïve sleep investigators gave similar results. Sleep-dependent respiratory and cardiovascular estimates did not depend on the scoring method. We show that non-invasive discrimination of the wake-sleep states of mice based on visual inspection of the WBP signal is accurate, reliable and reproducible. This work may set the stage for non-invasive high-throughput experiments evaluating sleep and breathing patterns on mouse models of pathophysiology.

The characterization of breathing, particularly during sleep is a topic of considerable ongoing interest^1^,^2^,^3^,^4^. Many of these studies focus on the pathogenesis of common sleep-related breathing disorders, such as obstructive or central sleep apneas, and associated cardiovascular and metabolic comorbidities^1^,^3^,^5^,^6^. The availability of genetically-modified mice models has the potential to accelerate our understanding of the underlying pathophysiology of such disorders^1^. Mice which lack leptin^7^, orexin^8^ or the endocannabinoid receptor-1^9^, are particularly susceptible to subtle disturbances in the regulation of breathing during sleep. Mouse studies typically require a combination of the techniques e.g. whole-body plethysmography (WBP) for non-invasive evaluation of breathing and respiratory control, and discrimination of wake-sleep states based on electroencephalographic/electromyographic (EEG/EMG) signals. The latter is an invasive procedure that involves surgical implantation of cranial electrodes and considerable post-operative recovery. Moreover, surgical techniques on such a small mammal requires great skill and training, quite complex recording apparatus and expertise to analyse the bio-signals obtained^10^. These limitations (and the need for some degree of restraint by a tether) mean that such procedures are unsuitable for large-scale phenotyping or genome-wide cohort association screening studies. WBP recordings entail costs for equipment (WBP chamber and pressure transducer) but no live expenses other than compressed air cylinders, which are inexpensive compared with surgical consumables, anaesthetics, and electrodes. While breathing can be recorded by only one mouse per WBP chamber, there is no intrinsic limit to the number of WBP chambers that may be operated in parallel in the context of high-throughput phenotyping studies.

We have already used continuous visual inspection of the WBP signal features coupled with the direct observation of the mice inside the WBP chamber to discriminate wake-sleep states non-invasively^11^,^12^,^13^. We also have extensive experience in the conventional EEG/EMG-based discrimination of mouse wake-sleep states^9^,^14^–17. Since no formal evaluation of concordance between the two methods has yet been undertaken, we assessed how well the WBP-based sleep scoring method compares with conventional, “gold standard” EEG/EMG-based scoring of murine wake-sleep behaviour.

We performed WBP-based and EEG/EMG-based scoring of wake-sleep states of the same cohort of mice to determine the extent of concordance between both scoring methods regarding (i) the discrimination of wake-sleep states and (ii) the estimation of basal respiratory (as well as cardiovascular) variables in each sleep state (Fig. 1).

## Methods

The study protocol was approved by the Committees on the Ethics of Animal Experiments of the University of Bologna and of the Italian Ministry of Health (Authorization n.245/2015-PR issued April 10, 2015) All the experimental procedures were conducted in conformity with the institutional guidelines in compliance with the national (Legislative Decree n. 26, March 4, 2014) and international law and policies (EEC Council Directive 2010/63/EU), and in accordance with the recommendations in the Guide for the Care and Use of Laboratory Animals of the National Institutes of Health.

### Experimental Protocol

C57BL/6J mice were housed at 25 °C in the animal facility of the Department of Biomedical and Neuromotor Sciences (Bologna, Italy) with a 12:12 hours’ light-dark cycle and free access to water and food. Twenty-two adult (17.2 ± 0.5 weeks) male C57BL/6J mice underwent surgery for the implantation of EEG/EMG electrodes and the insertion of an abdominal catheter for the telemetric recording of blood pressure^18^. Since blood pressure measurements are routinely incorporated in our studies of mice, we took the opportunity to compare the extent of concordance between basal cardiovascular (as well as respiratory) measurements in sleep states discriminated by the WBP-based versus EEG/EMG-based scoring methods (i.e. blood pressure is reported as an ancillary finding and was not used to score wake-sleep states). As previously^18^, a telemetric blood pressure transducer (TA11PA-C10, DSI, Tilburg, the Netherlands) was implanted subcutaneously on the right flank, with the catheter inserted through the right femoral artery into the abdominal aorta. A pair of Teflon-coated stainless-steel electrodes (Cooner Wire, Chatsworth, CA, USA) soldered to miniature stainless-steel screws (2.4 mm length, Plastics One, Roanoke, VA, USA) were positioned in contact with the dura mater through burr holes in the frontal and parietal bones to obtain a differential EEG signal. A second pair of these electrodes was inserted bilaterally in the nuchal muscles to obtain a differential EMG signal. All electrodes were connected to a miniature custom-built socket, which was cemented to the skull with stainless-steel screws, dental cement (RelyX Unicem 2, 3 M ESPE, Segrate, Milano, Italy), and dental acrylic (Respal NF, SPD, Mulazzano, Italy). After at least 10 days of postoperative recovery, mice were briefly anesthetized to plug the EEG/EMG recording cable and then individually placed inside a modified 2-chamber WBP (PLY4223, Buxco, Wilmington, NC, USA). The mouse chamber was modified by inserting a solid, machined 10-cm diameter plexiglas block, which reduced the internal volume to 0.97 L. The tower and chamber accommodated a rotating electrical swivel (SL6C/SB, Plastics One, Roanoke, VA, USA) and probes to measure temperature and humidity (PC52–4-SX-T3 sensor, Rense Instruments, Rowley, MA, USA). The differential pressure between the chamber which contained the mouse and a 2^nd^ reference chamber was measured with a high-precision differential pressure transducer (DP103–06 + CD223 digital transducer indicator; Validyne Engineering, Northridge, CA, USA)^9^,^19^. Recordings were always performed at the same Zeitgeber time (ZT5–ZT6 with ZT0 corresponding to lights on) for a total of 25 minutes after the mouse habituated to the new environment for 5 hours. During habituation and recordings, the WBP chamber was continuously purged at 2.0 L/h with air fed directly from a cylinder (no warming or humidification). EEG/EMG, blood pressure and respiratory signals were continuously recorded together with WBP chamber humidity and temperature. The system was calibrated dynamically with a 100 μL micro-syringe (Hamilton, Reno, NV, USA) at the termination of each recording. The EEG and EMG signals were amplified and filtered (EEG: 0.3-100 Hz; EMG: 100–1000 Hz; 7P511J amplifiers, Grass, West Warwick, RI, USA). The EEG and EMG amplifier gains were adjusted for each mouse to avoid signal saturation^9^,^19^. The TA11PA-C10 transducer transmitted the blood pressure signal by means of radio waves to a receiver (RPC-1, DSI) below the WBP. The blood pressure signal was then routed to a calibrated analog adapter (R11CPA, DSI) with compensation for barometric pressure (APR-1, DSI). All signals were digitized together at 16-bit and 1024 Hz with a PCI-6224 board (National Instruments, Austin, TX, USA) and customized software (Labview; National Instruments) and stored on digitally. Signals were down-sampled at 128 Hz (EEG, EMG and WBP chamber pressure) 4 Hz (WBP humidity and temperature).

### Wake-sleep cycle discrimination

Visual scoring of wake-sleep states based on EEG/EMG signals was performed by 3 trained investigators (S.B., C.B., V.L.M.) on all consecutive 4 s epochs. Wakefulness (W) was scored when the EMG tone was high and the EEG was at a low voltage. Non-rapid-eye-movement sleep (NREMS) was scored when the EMG tone was lower than in W and the EEG was at a high voltage with prominent δ (0.5–4 Hz) frequency components. Rapid-eye-movement sleep (REMS) was scored when the EMG indicated muscle atonia with occasional muscle twitches and the EEG was at a low voltage with predominant θ (6–9 Hz) frequency components. If the EEG/EMG signals did not conform to any of these cases, epochs were scored as undetermined (UN)^20^.

The same investigators also performed scoring of wake-sleep states based on WBP breathing signal whilst blinded to the EEG/EMG signals. The criteria for the sleep scoring based on the pattern (regularity, rate and depth) of breathing efforts, and the frequency and duration of gross body movements recorded in the WBP chamber have been already described^11^,^12^,^13^,^21^,^22^. Briefly, the WBP-based sleep scoring method relied on three components of the WBP trace: (i) frequency, (ii) amplitude and (iii) baseline, each assessed from qualitative visual inspection of the raw signal. W was scored when the baseline was highly irregular, largely obscuring individual breaths (Fig. 2; cf. Supplementary Fig. S1 and Fig. S2 for more examples). NREMS was scored when: (1) breathing frequency and amplitude were stable and regular, and (2) baseline was steady (Fig. 2 and Fig. 3; Supplementary Fig. S1 and Fig. S2 for more examples). REMS was scored when: (1) breathing frequency and amplitude were irregular, and (2) baseline was steady (Fig. 3; cf. Supplementary Figs S1, S2 and S3 for more examples). Large environmental pressure perturbations (e.g. due to opening/closing of a laboratory door) that transiently swamped the WBP signal were considered as external signal artefact. The transition from W to NREMS was identified as the point when the baseline became steady and breathing rhythm (frequency and amplitude) regular (Supplementary Fig. S2). Occasionally, at the end of W, baseline steadied but breathing rhythm remained relatively rapid compared to subsequent NREMS; such epochs were scored as W since mice were considered to be drowsy. Bouts of NREMS ended in either 1) the sudden onset of REMS, the beginning of which was scored at the first detectable sign of irregularity of the WBP signal (Fig. 3 with additional examples in Supplementary Figs S1, S2 and S3), or 2) sudden onset of W (large and irregular baseline perturbations that completely obscured breathing efforts). Brief (3–4 s) arousals during NREMS were evident as sudden, large, transient baseline perturbations. Augmented breaths (sighs) were abrupt, high-amplitude excursions of the raw WBP signal with a waveform similar to that of surrounding breaths (Supplementary Fig. S4). Epochs with sighs were typically scored as NREMS unless accompanied by a concomitant large, irregular baseline perturbation, in which case the epoch was scored as W (Supplementary Fig. S4). Using these criteria, WBP-defined epochs were assigned as W, NREMS or REMS i.e. no epoch was scored as UN. The Dataset 1 (file PRISM.doc) and Supplementary Fig. S1 include a worked example of WBP-based vs. EEG/EMG-based wake-sleep scoring. LabChart 7 Pro (ADInstruments, UK) was used to display the raw, high-resolution (128 Hz) WBP signal, to facilitate visual interpretation and wake-sleep discrimination. WBP epochs were assigned the wake-sleep state that prevailed for >2 s (i.e. >50% of each 4 s epoch), and two complementary databases (one based on the WBP method and another generated by EEG/EMG-based method) were generated for each mouse.

### Inter-scorer agreement of the WBP-based wake-sleep scoring

The inter-scorer agreement of the WBP-based sleep scoring method was assessed for a random subset of 5 mice. Inter-scorer agreement was estimated: 1) between 2 experienced murine sleep research investigators (S.B. and C.B); 2) between an experienced sleep investigator (S.B.) and a mouse researcher with no specific training or experience in murine respiratory/sleep physiology (S.S). For each of these evaluations of inter-rater agreement, we calculated the total percentage of epochs scored in the same way by the 2 investigators (overall agreement) and the Cohen’s Kappa index (which estimates inter-rater agreement taking into account agreement that may occur by chance)^23^.

### Comparison between the WBP-based and EEG/EMG-based wake-sleep scoring methods

The WBP-based and the EEG/EMG-based wake-sleep scoring methods were compared using several approaches. As a first step, overall agreement and the Cohen’s Kappa index between the 2 scoring methods were computed for 20 mice. For each wake-sleep state, Bland-Altman plots were produced to identify systematic differences between the two methods. For the purpose of these analyses, the EEG/EMG-based scoring method was considered the “gold standard”, and epochs scored as UN according to the EEG/EMG method were discarded. For each wake-sleep state (i.e., W, NREMS, and REMS), 4-s epochs correctly or incorrectly assigned to that state or to a different state by the WBP-based scoring were defined as true positives (TP) or false positives (FP), respectively, and epochs correctly or incorrectly assigned to a different state by the WBP-based scoring were defined as true negatives (TN) or false negatives (FN), respectively. We combined these to estimate overall sensitivity (TP/(TP + FN)), specificity (TN/(TN + FP)), and accuracy ((TP + TN)/(TP + TN + FP + FN)) of the WBP-based vs. the EEG/EMG-based scoring method for each wake-sleep state. To assess the capacity of the WBP-based scoring to identify brief arousals from sleep compared to EEG/EMG-based scoring, the estimated mean duration of sleep episodes (sleep bouts ≥12 s) was compared between the 2 methods.

### Comparison between quantitative estimates of EEG power spectra, rate and depth of breathing, and mean values of blood pressure and heart period obtained with each scoring method

**Figure 1:**
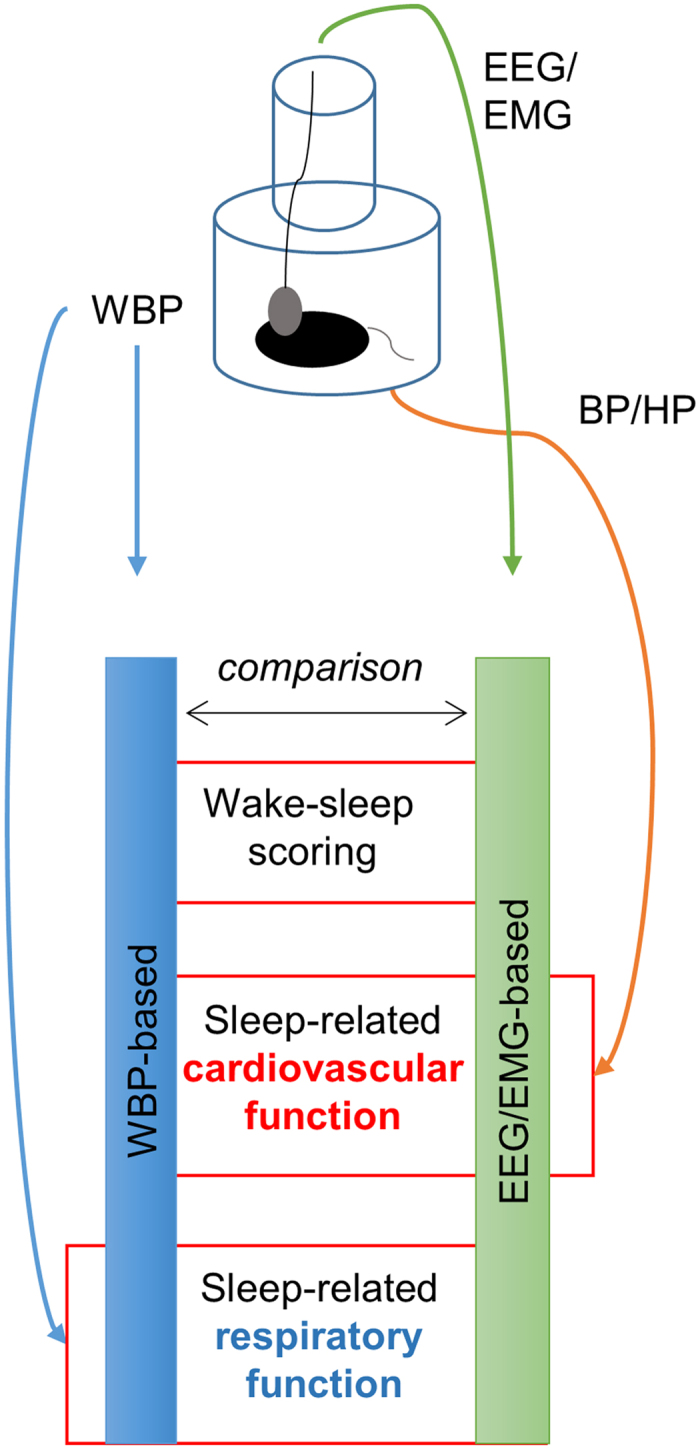
Experimental design. The scheme represents the rational we followed for the present study. The whole-body plethysmography (WBP), the electroencephalographic (EEG) and the electromyographic (EMG) signals were recorded together with the telemetric acquisition of blood pressure (BP) and heart period (HP) values in each mouse. The wake-sleep scoring method based on WBP was then compared to that based on EEG/EMG signals by evaluating the performance in correctly discriminating the wake-sleep states and comparing estimates of respiratory and cardiovascular variables in each sleep state computed on the basis of each method.

**Figure 2:**
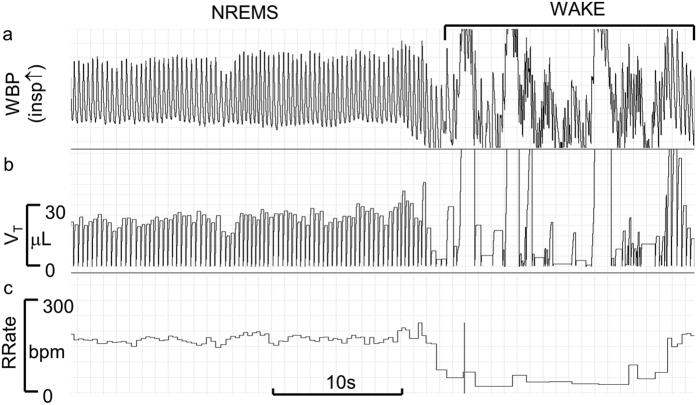
The WBP signal at the transition between NREMS and wakefulness. Panel a shows a representative raw tracing of the whole-body plethysmography signal (WBP, upwards deflection indicating inspiration) at a transition between non-rapid-eye-movement sleep (NREMS) and wakefulness. For the sake of clarity, the respiratory rate (RRate) and the instantaneous breath-to-breath tidal volume (V_T_), computed on the basis of the raw WBP signal, are reported in panel b and c, respectively.

**Figure 3:**
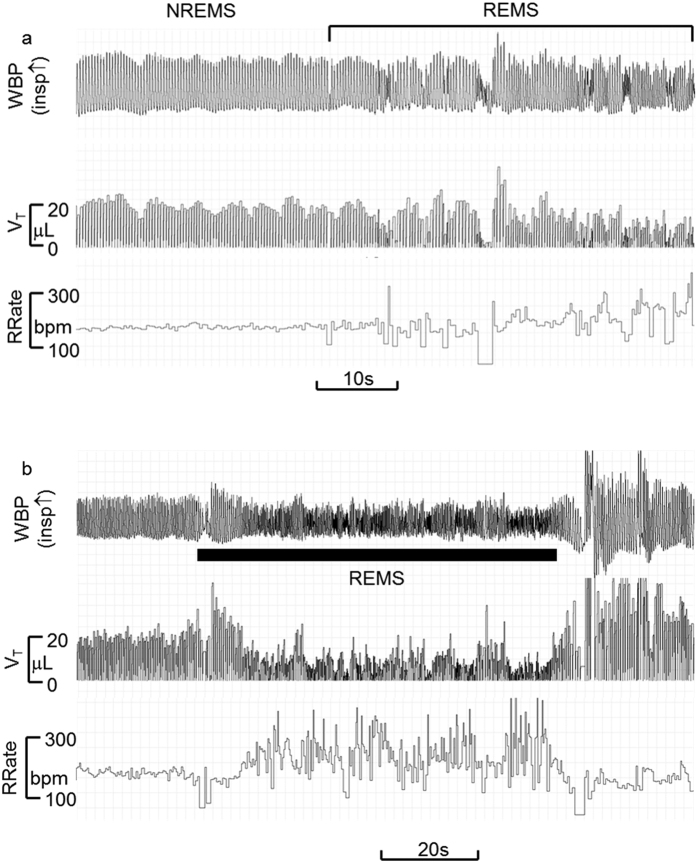
The WBP signal at the transition between NREMS and REMS. Panel a shows a representative raw tracing of the whole-body plethysmography signal (WBP, upward deflection indicating inspiration) at a transition between non-rapid-eye-movement sleep (NREMS) and rapid-eye-movement sleep (REMS). Panel b shows at a lower magnification a different raw tracing of the WBP signal at the transition between NREMS and REMS. The respiratory rate (RRate) and the instantaneous breath-to-breath tidal volume (V_T_), tracings computed on the basis of the raw WBP signals were not made available to the scorers in the present study, but are nonetheless included in the figure panels for the sake of explanation.

Spectral analysis of the EEG signal was performed using discrete Fourier transform after linear detrending on 4-s epochs. Inter-individual differences in EEG spectral power were accounted for by expressing EEG power spectral density of each 4-s epoch as % of total EEG power in the 0.5–20 Hz range for that epoch^17^. The analysis of respiratory and cardiovascular variables in each sleep state was repeated twice for each mouse, i.e., once using the EEG/EMG-based and again using the WBP-based wake-sleep scoring (Fig. 1). This analysis was confined to periods of NREMS and REMS (W was excluded due to the prevalence of breath-obscuring artefact). We confined the comparison to bouts of stable sleep lasting ≥12 s (i.e., at least 3 consecutive 4-s epochs)^18^,^19^. Individual breaths were identified automatically from the upward (+) WBP pressure deflection peak. Errors in breath detection (as well as pressure artefacts due to movements etc.) were manually excluded from the analyses. Total breath duration (i.e. interval between successive breaths, T_TOT_), tidal (V_T_) and minute volume (V_E_ = V_T_/T_TOT_) were calculated, and volumes expressed per gram body weight. For each mouse in each sleep state, augmented breaths (sighs) were defined as V_T_ > 3 times average V_T_, and breathing pauses (apneas) as T_TOT_ > 3 times average T_TOT_^9^,^14^. Each sigh and apnea was visually inspected and artefacts were excluded. Beat-to-beat values of systolic blood pressure (SBP) and heart period (HP, i.e., the time interval between the onset of successive systolic upstrokes) were computed from the raw arterial pressure signal with manual rejection of artefacts^18^,^19^.

### Statistical analysis

All the variables included in the study resulted normally distributed in each wake-sleep state (Shapiro-Wilk test, P > 0.05). Data were analyzed by paired t-tests with sample size N = 20 except for the comparisons of sleep-dependent respiratory and cardiovascular estimates during REMS. In particular, due to the fact that some mice did not show any REMS period during the recordings, we had to restrict these analyses to n = 16 mice. Statistical analyses were performed using SPSS software (SPSS software, SPSS Inc., Chicago, IL, USA) and results are shown as mean ± SEM or, for Cohen’s Kappa index, 95% confidence interval (CI) with significance at P < 0.05.

## Results

### Subject exclusion

Two out 22 mice were excluded from the analysis because the WBP signal was unreliable (markedly irregular signal due to repetitive, unexplained large negative pressure fluctuations Supplementary Fig. S5).

### Inter-scorer agreement of the WBP-based wake-sleep scoring

Table 1 shows the overall percentage of agreement and the Cohen’s Kappa index calculated between the 2 expert sleep investigators and between one expert and one naïve mouse sleep investigator. These measures indicated that inter-scorer agreement was high and not due to chance.

### Comparison between the WBP-based and EEG/EMG-based wake-sleep scoring methods

**Figure 4:**
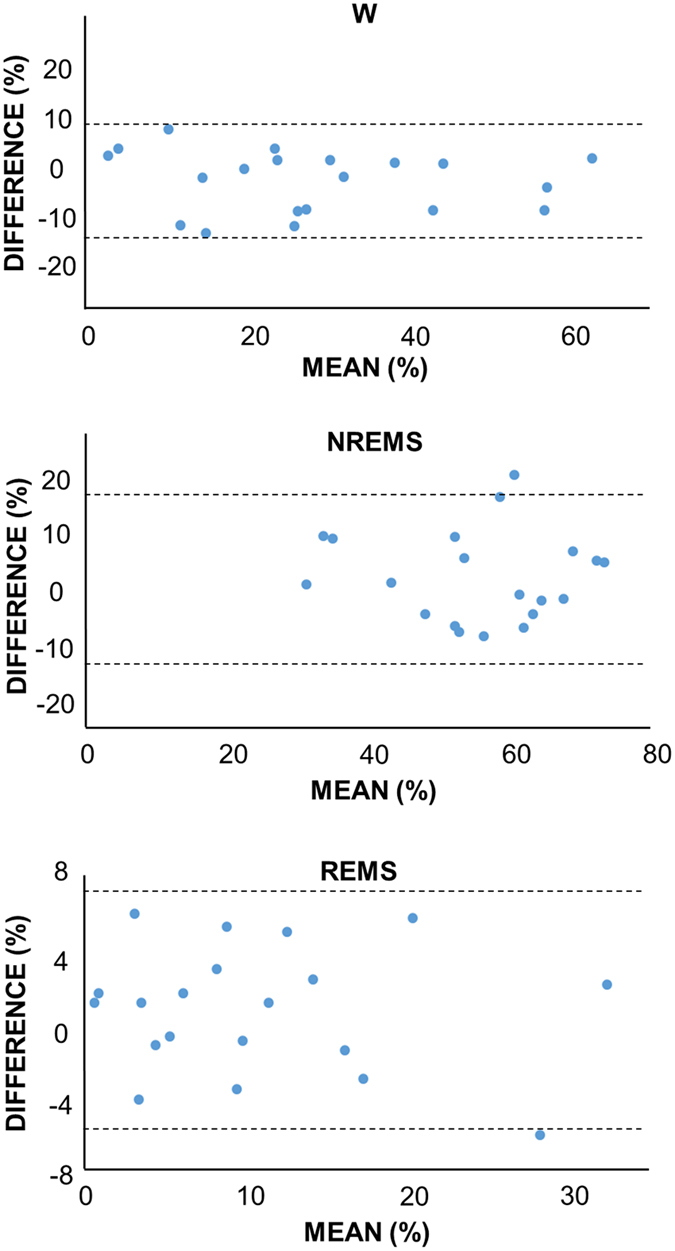
Application of Bland-Altman plots to compare the EEG/EMG- and the WBP-based sleep scoring methods. Comparison by Bland-Altman plots of the percentage of recording time assigned to each wake-sleep state by the sleep scoring methods based on electroencephalography/electromyography- and whole-body plethysmography. The 3 Bland-Altman plots show the mean values (x axis) of the percentages of recording time scored as wakefulness (W), non-rapid-eye-movement sleep (NREMS) and rapid-eye-movement sleep (REMS) with the 2 scoring methods on 20 wild-type mice vs. the differences (y axis) of the same quantities between the two scoring methods. Horizontal lines delimit the 95% confidence interval of the distribution of these differences.

As explained (cf. Methods), the EEG/EMG method (but not the WBP method) included epochs designated as UN (5.8 ± 0.6% of EEG/EMG recording time). Such epochs were predominately assigned as either NREMS (54%) or W (39%) using the WBP method. Excluding UN epochs, the overall agreement between the WBP-based and EEG/EMG-based wake-sleep scoring methods was 90%, with a high and significant Cohen’s Kappa index of 0.82 (95% CI, 0.81–0.83; P < 0.001). Table 2 shows values of accuracy, specificity and sensitivity of the WBP-based scoring method in detecting W, NREMS and REMS compared with the EEG/EMG-based method. Figure 4 shows the Bland-Altman plots comparing the percentage of recording time spent in W, NREMS and REMS according to the WBP-based or the EEG/EMG-based scoring methods. The % of recording time spent in each wake-sleep state according to either the WBP- or the EEG/EMG-based sleep scoring method (Table 3) was in line with previous data reported on mice studied outside the WBP chamber at the same circadian time (ZT5–ZT6)^15^. However, the WBP-based scoring method slightly but significantly overestimated W time compared to the EEG/EMG-based method (Table 3). This systematic difference in W time between the two scoring methods did not significantly correlate with the average W time estimate of the two methods. Differences between estimated NREMS and REMS time scored by the two methods were not statistically significant (Table 3). Consequently, the mean duration of NREMS and REMS episodes did not differ (P = 0.439) when estimated with the WBP- (165 ± 9 s and 37 ± 6 s, respectively) versus the EEG/EMG-method (170 ± 9 s and 38 ± 6 s, respectively).

### Comparison between quantitative estimates of EEG power spectra, rate and depth of breathing, and mean values of blood pressure and heart period obtained by the two scoring methods

The analysis of the EEG spectral power revealed that the spectral peaks during W, NREMS and REMS scored based on the WBP method occurred at similar frequencies (4.6 ± 0.4 Hz, 2.8 ± 0.3 Hz and 6.9 ± 0.2 Hz, respectively) to that derived from the EEG/EMG scoring method (4.6 ± 0.4 Hz, 2.4 ± 0.2 Hz and 6.9 ± 0.1 Hz, respectively; P > 0.153). Table 4 and Table 5 compare mean respiratory and cardiovascular parameters, respectively, obtained with the two scoring methods. The WBP-based method led to estimates of the mean value of HP during NREMS that were slightly but significantly lower than those obtained with the EEG/EMG-based method (Table 5). Save for this exception, mean values were comparable regardless of which scoring method was used. The estimated occurrence rate of sighs and apneas during NREMS was also comparable (P = 0.479 and P = 0.580, respectively) for the EEG/EMG- (11.3 ± 2.0 and 4.6 ± 1.2 events/h of NREMS, respectively) versus the WBP-method (13.9 ± 2.9 and 5.8 ± 2.4 events/h of NREMS, respectively).

## Discussion

Our results lead to 3 main conclusions: (a) discrimination of mouse wake-sleep states based only on a sensitive, non-invasive method that simultaneously registers breathing efforts and body movements (WBP) is reliable and comparable to the classical EEG/EMG-based scoring method (high overall agreement and Cohen’s Kappa index > 0.80 indicating an “almost perfect strength of agreement”^23^), (b) the scheme we outline to interpret and analyze the WBP signal of a mouse is simple and clear, and the results highly reproducible; (c) the two scoring methods provide highly comparable quantitative estimates of sleep-dependent respiratory (as well as cardiovascular) variables.

Scoring of murine sleep-wake cycle based on the visual inspection of the WBP signal features coupled with the direct observation of the mice inside the WBP chamber has been used successfully before^11^,^13^,^24^. In the present study, we took advantage of this background to perform the first formal evaluation of the concordance between wake-sleep scoring based only on a raw WBP signal vs. the “gold standard” EEG/EMG-based method. We excluded only a small number of studies (2/22; Supplementary Fig. S5) due to an anomalous WBP signal (this prevented application of the WBP-scoring criteria, did not occur in any other mice, and was attributed to unexplained technical problems). We therefore emphasize the importance of visually inspecting and verifying the integrity of the WBP signal at the time of recording, and rectifying technical problems before proceeding. Since the WBP is transparent, investigators inexperienced in the WBP-method should initially be encouraged to make simultaneous notes of the position and movements of the mouse, as observed through the WBP. These on-line observations can assist in later (off-line) scoring, and aid in developing the confidence needed to reliably interpret and correlate the WBP signal with various phases of the murine wake-sleep cycle. For the 20 mice for which WBP-based scoring was successfully used, agreement in the application of the WBP-based sleep scoring method between expert sleep investigators was excellent and a naïve investigator performed as well as an expert investigator (Table 1). Moreover, the inter-rater agreement of the WBP-based sleep scoring was in line with previously published values (86–92%) for the gold standard EEG/EMG-based method^25^,^26^,^27^. We achieved exceptionally high overall agreement (90%) between the WBP-based and the EEG/EMG-based method, with only 10% of misclassified epochs. This level of agreement is better than that previously reported using a different non-invasive technique (video recordings), for which the overall error rate was 23.3%^28^. Our WBP-method showed slightly lower specificity (−5%, −7% and −7% for W, NREMS and REMS detection, respectively) compared with another non-invasive (piezoelectric sensor-based) behavioral scoring method^29^, but performed much better in terms of sensitivity (+6%, +17%, and +14%, for W, NREMS and REMS detection, respectively). The WBP and piezoelectric sensor technologies are, however, very different: the latter relies on physical contact between the mouse and detector on the cage floor whereas the former does not. The WBP has a crucial advantage: it provides simultaneous information on the rate and depth of breathing, which makes it a valuable method for physiological, pathophysiological and pharmacological phenotyping of mice used to model a wide variety of sleep-dependent breathing disorders. The fine behavioral manifestations detected with such fidelity by the WBP–method (e.g. rapid shallow breathing) are crucial for accurately scoring REMS, when muscle atonia prevents gross body movements. This explains why the WBP-method performs so exceptionally well in terms of sensitivity and specificity for detecting REMS – a remarkable observation given that REMS is a such small % of murine total sleep time, and that differences of even a few epochs between scoring methods can substantially decrease sensitivity and specificity of REMS estimates. Accordingly, the sensitivity of an automatic EEG/EMG based scoring algorithm that we recently developed (SCOPRISM) had the lowest sensitivity values during REMS^10^,^20^. Piezoelectric sensors are much less sensitive in discriminating REMS^29^ – perhaps their most serious limitation. Video is relatively accurate in discriminating W from sleep (error rate = 8.8%) but less so when REMS is included (error rate = 23.3%)^28^.

Performing recordings in a WBP chamber does have limitations: the chamber is not the same as the home cage in terms of size, general environment and overall comfort, which must inevitably impact on behavior, including sleep and/or arousal. No experimental situation, however, can be considered entirely natural. Nevertheless, the % of recording time spent in each wake-sleep state by the mice we studied in the WBP chamber (Table 3) was in line with data from mice studied outside a WBP^15^. This observation suggests that mice adapt rapidly and their behavior is relatively normal inside a WBP. The relatively small WBP chamber may not be suitable for undisturbed long-term (e.g. circadian) sleep studies, since waste accumulates. However, experience with indirect calorimetry chambers comparable in size to our WBP chamber demonstrated that mice can be successfully recorded in these environments for at least 24 hours^30^. Because the mouse is enclosed and sealed in the WBP, some techniques (e.g. gentle handling to produce sleep deprivation and study sleep homeostasis) are impractical^17^. Technical refinements may allow other techniques to be used to induce experimental sleep fragmentation inside a WBP e.g. acoustic stimulation. Moreover, the WBP may be used to study sleep rebound after sleep deprivation in a normal rodent cage.

Sighs assist in WBP-based sleep scoring (cf. Methods) but are also of physiological interest because they are typically followed by a brief central apnea. Estimates of the rate of sighing (and apneas) during NREMS were similar for the two methods – perhaps not surprising given that WBP directly measures breathing rhythm, and that sighs are a characteristic feature of NREMS^31^.

In conclusion, WBP-recorded breathing and body movement patterns of mice can be used to score non-invasively wake-sleep states with an accuracy close to that attained by trained observers who had only EEG/EMG signals available. The WBP technique is economical, affordable and less technically demanding than EEG/EMG recording and provides, in addition, a continuous, quantitative measure of breathing. It does not replace, but complements EEG/EMG recordings and is, we believe, a valuable, practical, cost-effective alternative technique. Robust, relatively inexpensive techniques that require minimal labor to simultaneously quantify wake-sleep behavior and breathing with minimal or no restraint have, we believe, enormous appeal for routine, relatively rapid screening and phenotyping.

## Additional Information

**How to cite this article:** Bastianini, S. *et al*. Accurate discrimination of the wake-sleep states of mice using non-invasive whole-body plethysmography. *Sci. Rep.* **7**, 41698; doi: 10.1038/srep41698 (2017).

**Publisher's note:** Springer Nature remains neutral with regard to jurisdictional claims in published maps and institutional affiliations.

## Supplementary Material

Supplementary Information

Supplementary Dataset 1

## Figures and Tables

**Table 1:** Inter-scorer reliability of the WBP-based wake-sleep scoring method.

	Overall Agreement	Cohen’s Kappa (95% confidence interval)
Expert Inter-scorer agreement	91%	0.83* (0.81–0.85)
Expert vs. untrained inter-scorer agreement	87%	0.78* (0.75–0.81)

The overall percentage of agreement and the Cohen’s Kappa indexes were calculated to compare the sleep scoring method based on whole-body plethysmography (WBP) between 2 expert sleep investigators and between one expert sleep investigator and an investigator with no specific training on mouse sleep. *P < 0.001.

**Table 2:** Accuracy, specificity and sensitivity of the wake-sleep scoring method based on WBP with respect to that based on EEG/EMG signals.

	Wakefulness	NREMS	REMS
Accuracy (%)	91.6 ± 0.3	91.1 ± 0.4	84.3 ± 1.1
Specificity (%)	85.8 ± 0.2	84.7 ± 0.4	85.6 ± 0.5
Sensitivity (%)	95.5 ± 0.2	97.9 ± 0.1	80.9 ± 1.1

Comparison of the performance of the wake-sleep scoring method based on whole-body plethysmography (WBP) with that of the scoring method based on electroencephalographic (EEG) and electromyographic (EMG) signals in correctly discriminating wakefulness, non-rapid-eye-movement sleep (NREMS) and rapid-eye-movement sleep (REMS).

**Table 3:** Percentage of recording time spent in each wake-sleep state based on WBP with respect to that based on EEG/EMG signals.

	Wakefulness	NREMS	REMS
EEG/EMG	28.1 ± 4.1	59.9 ± 3.2	12.0 ± 2.1
WBP	31.5 ± 4.3*	57.2 ± 3.5	11.3 ± 2.2

Percentage of recording time spent in wakefulness, non-rapid-eye-movement sleep (NREMS) and rapid-eye-movement sleep (REMS) computed on the base of whole-body plethysmography (WBP) with respect to that based on electroencephalographic and electromyographic signals (EEG/EMG). *P < 0.05 vs. EEG/EMG.

**Table 4:** Comparison between estimates of sleep-related respiratory variables obtained with the wake-sleep scoring method based on WBP with respect to that based on EEG/EMG signals.

		EEG/EMG	WBP	P
V_T_ (μl/g)	NREMS	9.5 ± 0.5	9.5 ± 0.5	0.691
	REMS	7.8 ± 0.5	7.8 ± 0.5	0.950
T_TOT_ (ms)	NREMS	361.9 ± 7.9	363.9 ± 7.7	0.288
	REMS	357.8 ± 14.4	359.2 ± 14.1	0.835
V_E_ (ml/min*g)	NREMS	1.6 ± 0.1	1.6 ± 0.1	0.806
	REMS	1.3 ± 0.1	1.3 ± 0.1	0.788

NREMS, non-rapid-eye-movement sleep; REMS, rapid-eye-movement sleep; V_T_, tidal volume; T_TOT_, total breath duration; V_E_, minute volume; WBP, sleep scoring based on whole-body plethysmography; EEG/EMG, sleep scoring based on electroencephalographic/electromyographic signals; P, p-values of the paired t-tests for EEG/EMG vs. WBP.

**Table 5:** Comparison between estimates of sleep-related cardiovascular variables obtained with the wake-sleep scoring method based on WBP with respect to that based on EEG/EMG signals.

		EEG/EMG	WBP	P
HP (ms)	NREMS	116.9 ± 2.2*	116.0 ± 2.2	0.012
	REMS	106.5 ± 1.8	108.4 ± 2.1	0.077
SBP (mmHg)	NREMS	134.1 ± 3.0	134.0 ± 2.2	0.841
	REMS	129.5 ± 2.7	129.5 ± 3.0	0.976

NREMS, non-rapid-eye-movement sleep; REMS, rapid-eye-movement sleep; HP, heart period; SBP, systolic blood pressure; WBP, sleep scoring based on whole-body plethysmography; EEG/EMG, sleep scoring based on electroencephalographic/electromyographic signals; P, p-values of the paired t-tests for EEG/EMG Vs. WBP. *P < 0.05 vs. WBP.
