# Supplementary material for: Accurate discrimination of the wake-sleep states of mice using non-invasive whole-body plethysmography
Source: Sci Rep. 2017 Jan 31;7:41698. doi: 10.1038/srep41698 (PMC5282481; doi:10.1038/srep41698)

ACCURATE DISCRIMINATION OF THE WAKE-SLEEP STATES OF MICE USING NON-INVASIVE WHOLE-BODY PLETHYSMOGRAPHY

Stefano Bastianini[1], Sara Alvente[1], Chiara Berteotti[1], Viviana Lo Martire[1], Alessandro Silvani[1], Steven J. Swoap[2], Alice Valli[1], Giovanna Zoccoli*[1] and Gary Cohen[3].

[1] Prism Lab, Department of Biomedical and Neuromotor Sciences, University of Bologna, Italy.

[2] Department of Biology, Williams College, Williamstown, Massachusetts, USA.

[3] Department of Women's and Children's Health, Neonatal Unit, Karolinska Institute, Stockholm, Sweden. Present affiliation: Sleep Investigation Laboratory, Centre for Sleep Health and Research, Royal North Shore Hospital, Sydney, Australia.

Supplementary Material

The Dataset 1 (PRISM.doc) file is a Word document (Office 97-2003) file including 10 minutes of raw tracings of the uncalibrated whole-body plethysmography (WBP; column 1), electroencephalographic (EEG; column 2), and electromyographic (EMG; column 3) signals and the WBP-based sleep scoring (SCO; column 4). All signals are reported with 128 samples per second, with one sample per row. Semicolons are used at delimiters. The code of the SCO signal is the following: values of 1 indicate wakefulness; values of 2 indicate non-rapid-eye-movement sleep (NREMS); values of 3 indicate rapid-eye-movement sleep (REMS).

Supplementary Figure S1. Worked example of the application of the criteria to score the wake-sleep states based on the WBP signal.

This figure represents 2 screenshots of the raw tracings included as a supplementary data file (file PRISM.txt) with a worked example of the criteria to score (SCO trace) the states of wakefulness (W, code 1), non-rapid-eye-movement sleep (NREMS, code 2) and rapid-eye-movement sleep (REMS, code 3) based on the whole-body plethysmography (WBP) signal. For the sake of comparison, we also included the relative electroencephalographic (EEG) and electromyographic (EMG) signals. Panel a shows a transition between NREMS and W whereas panel b shows a transition between NREMS and REMS

a

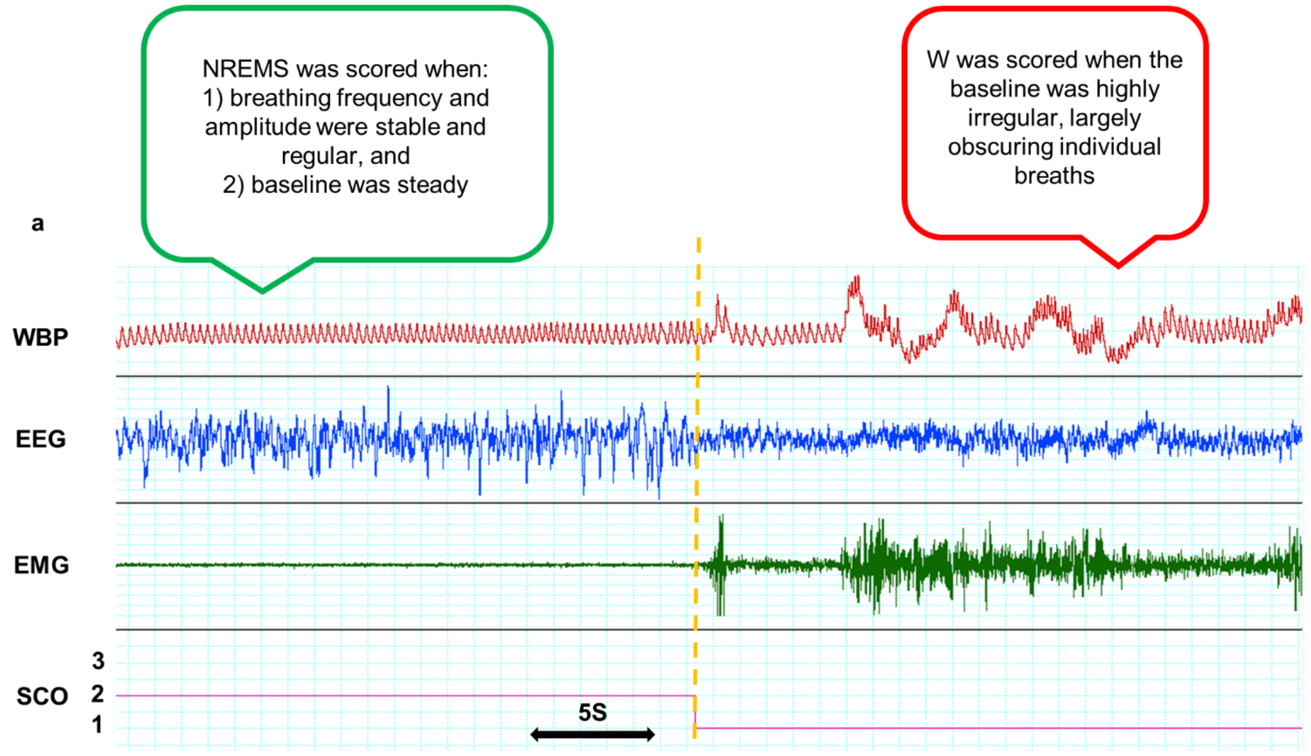
NREMS was scored when:
1) breathing frequency and amplitude were stable and regular, and
2) baseline was steady
W was scored when the baseline was highly irregular, largely obscuring individual breaths
WBP
EEG
EMG
SCO
3
2
1
5S

b

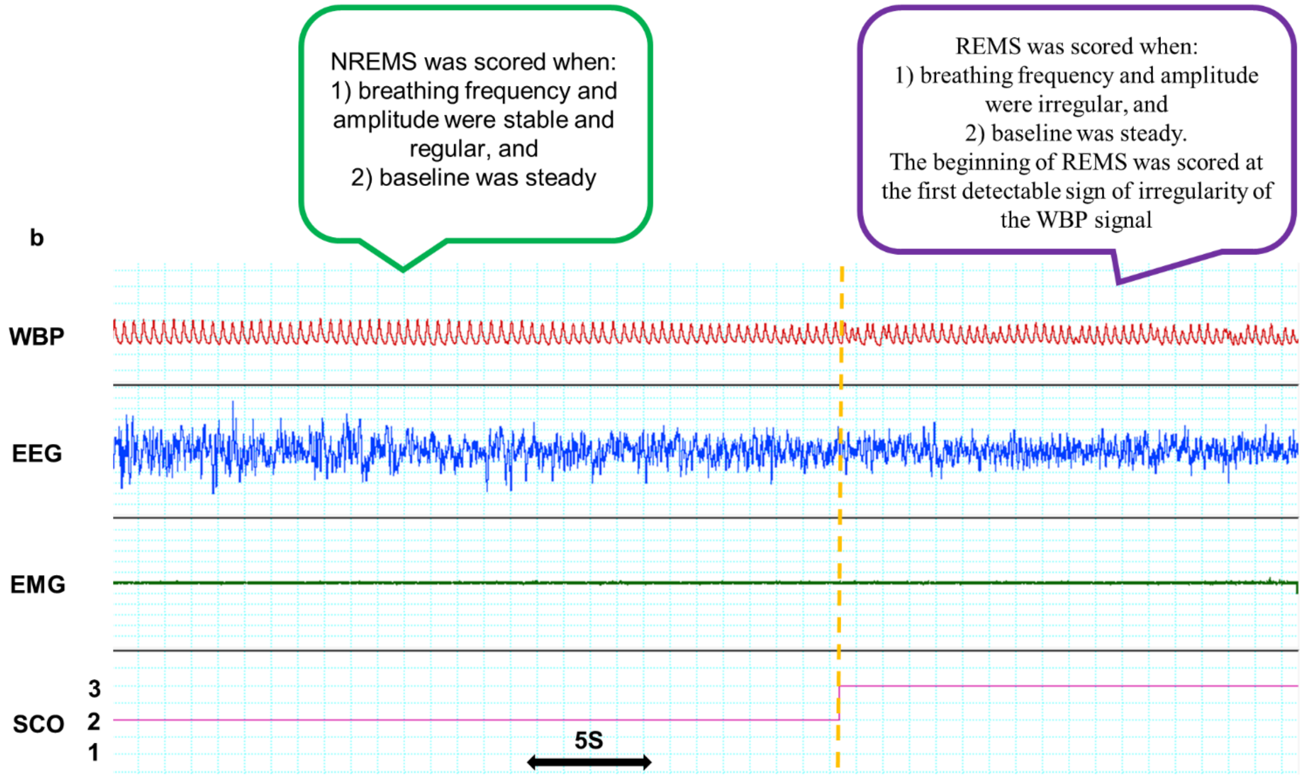
NREMS was scored when:
1) breathing frequency and amplitude were stable and regular, and
2) baseline was steady
REMS was scored when:
1) breathing frequency and amplitude were irregular, and
2) baseline was steady.
The beginning of REMS was scored at the first detectable sign of irregularity of the WBP signal
WBP
EEG
EMG
SCO
3
2
1
5S

Supplementary Figure S2. The WBP signal at transitions between wake-sleep states.

Representative raw tracings of the whole-body plethysmography (WBP) signals at the transitions between wakefulness (W) and non-rapid-eye-movement sleep (NREMS; panels a and c), between NREMS and rapid-eye-movement sleep (REMS; panel b), and between REMS and W (panel c). On the upper-right part of each panel is shown a magnification of the transition period between states (frame).

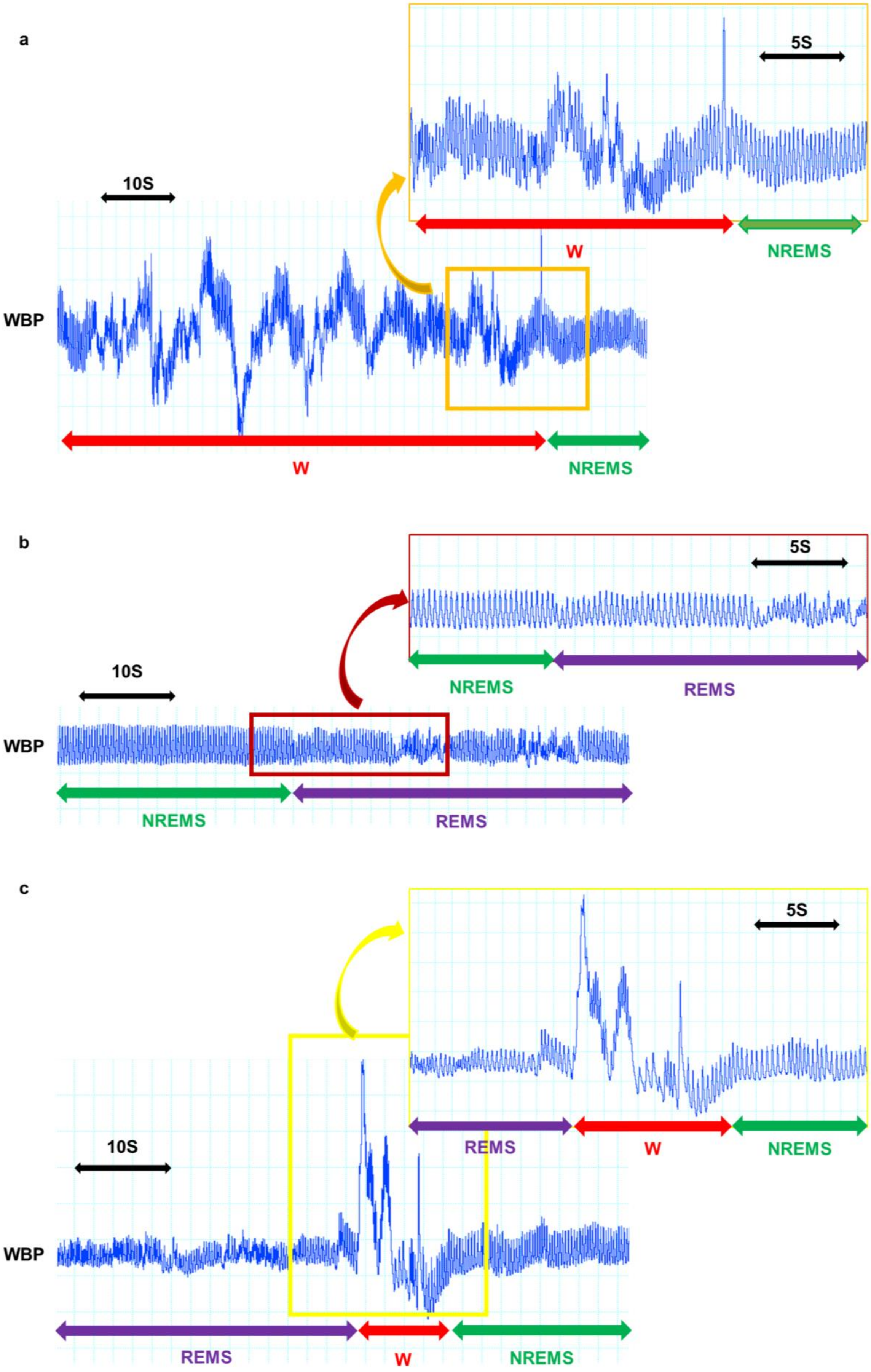

a
10S
5S
WBP
W
NREMS
W
NREMS
b
10S
5S
WBP
NREMS
REMS
NREMS
REMS
c
10S
5S
WBP
REMS
W
NREMS
REMS
W
NREMS

Supplementary Figure S3. The WBP signal at the beginning of REMS episodes.

Representative raw tracings of the electromyographic (EMG), electroencephalographic (EEG) and whole-body plethysmography (WBP) signals at the transition between non-rapid-eye-movement sleep (NREMS) and rapid-eye-movement sleep (REMS). On the upper-right part of the figure is shown a magnification of the NREMS to REMS transition (frame). Red arrows indicate the first detectable sign of irregularity of the WBP signal, which corresponds to the beginning of REMS.

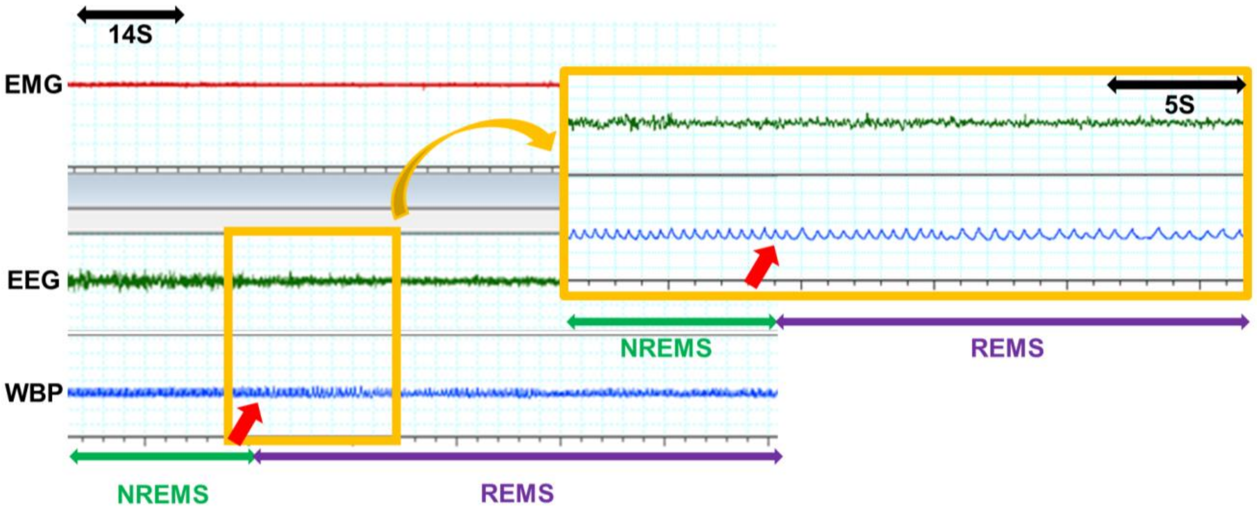

Supplementary Figure S4. Sighs occurrence in the WBP signal.

This figure shows 2 representative raw tracings of the whole-body plethysmography (WBP) signal during non-rapid-eye-movement sleep (NREMS). A sigh occurred in both panels. However, only in panel b was it concomitant with an evident WBP signal oscillation. Thus, according to our proposed scoring criteria (cf. Methods), the NREMS episode in panel b was interrupted by a brief period of wakefulness, whereas the NREMS episode in panel a was not.

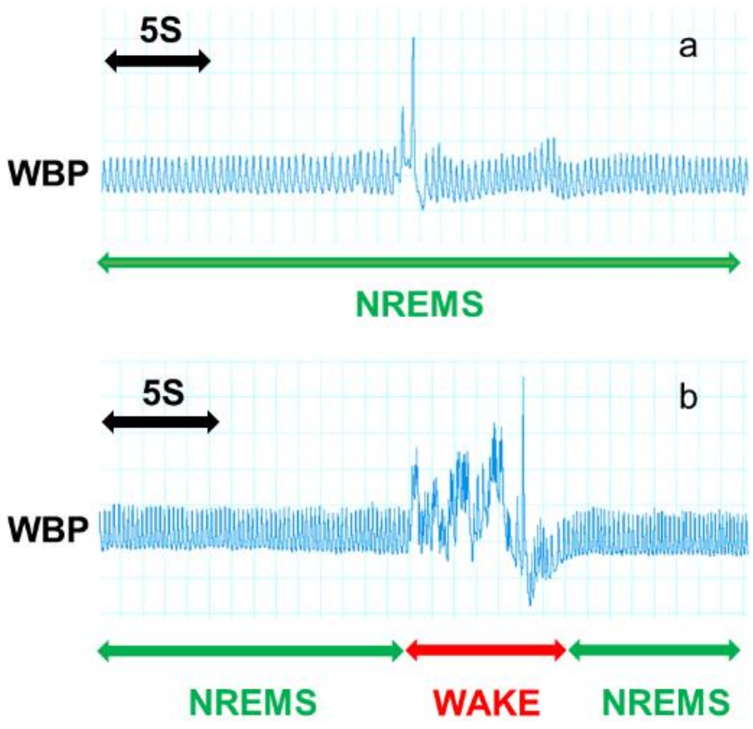

Supplementary Figure S5. Examples of abnormal raw tracings of the WBP signal.

This figure shows 2 raw tracings with abnormal whole-body plethysmography (WBP) signal in 2 different mice. In panel a, the WBP signal baseline resulted markedly unstable with repeated negative deflections, which did not match any of the scoring criteria that we proposed (cf. Methods). In panel b, unexplained and stereotyped negative fluctuations also occurred. Both these conditions prevented the correct interpretation of WBP signal variability.

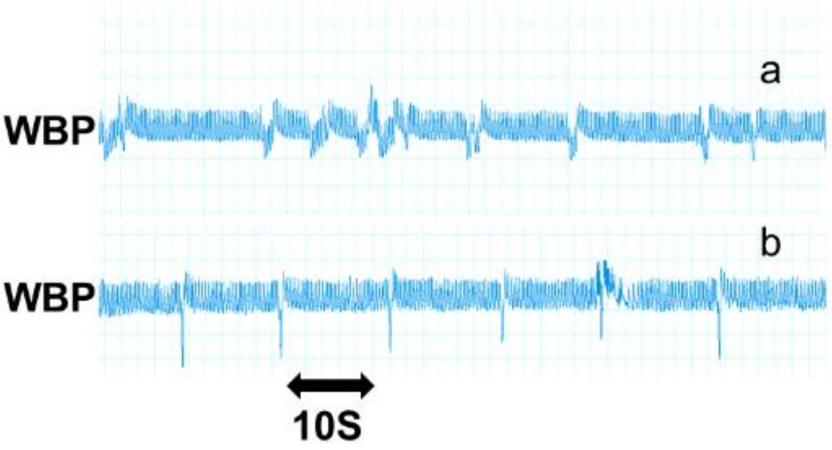

Supplement: Supplementary Information [file srep41698-s1.pdf]
