# Supplementary material for: Accurate discrimination of the wake-sleep states of mice using non-invasive whole-body plethysmography
Source: Sci Rep. 2017 Jan 31;7:41698. doi: 10.1038/srep41698 (PMC5282481; doi:10.1038/srep41698)
Supplement: Supplementary Dataset 1 [file srep41698-s2.doc]

-1081;-1,189;0,045;2

-1106;-1,091;-0,059;2

-1129;-0,939;0,074;2

-1156;-0,899;-0,018;2

-1173;-0,789;0,005;2

-1191;-0,312;0,075;2

-1202;0,555;-0,068;2

-1213;1,283;0,042;2

-1218;1,524;-0,007;2

-1220;1,772;-0,045;2

-1219;1,539;0,09;2

-1215;0,556;-0,06;2

-1216;-0,452;-0,072;2

-1218;-0,778;0,033;2

-1226;-0,661;-0,068;2

-1236;-0,439;0,083;2

-1239;0,613;-0,065;2

-1235;1,185;0,017;2

-1218;0,091;0,017;2

-1192;-0,457;-0,1;2

-1154;-0,525;0,079;2

-1109;-0,397;-0,029;2

-1051;-0,092;0,013;2

-989;-0,028;0,063;2

-924;0,537;-0,094;2

-853;1,188;0,102;2

-780;1,752;-0,03;2

-710;2,681;-0,022;2

-644;2,274;0,072;2

-581;0,746;-0,079;2

-534;-0,7;0,048;2

-507;-1,177;-0,018;2

-504;-1,183;-0,054;2

-532;-1,471;0,02;2

-584;-1,295;-0,071;2

-642;-1,051;-0,028;2

-704;-0,963;-0,009;2

-753;-0,772;-0,064;2

-796;-0,616;0,065;2

-829;0,085;-0,036;2

-859;1,384;0,003;2

-886;2,205;0,055;2

-915;2,828;-0,132;2

-938;2,085;0,077;2

-964;0,636;-0,038;2

-990;0,031;0,006;2

-1022;-0,317;0,071;2

-1056;-0,912;-0,06;2

-1090;-1,157;0,027;2

-1120;-0,323;-0;2

-1142;0,847;-0,026;2

-1159;0,739;0,068;2

-1164;0,188;-0,049;2

-1167;-0,321;0,034;2

-1164;-1,225;0,012;2

-1161;-1,583;-0,061;2

-1163;-1,832;0,108;2

-1164;-1,84;-0,052;2

-1169;-1,661;0,007;2

-1171;-1,405;0,02;2

-1174;-0,984;-0,074;2

-1178;-0,865;0,022;2

-1190;-0,792;-0,045;2

-1194;-0,443;0,003;2

-1194;-0,601;0,043;2

-1181;-0,668;-0,073;2

-1162;-0,471;0,115;2

-1138;-0,178;0;2

-1105;0,627;-0,027;2

-1060;1,148;0,082;2

-1004;1,3;-0,061;2

-938;0,702;0,1;2

-867;-0,601;0,027;2

-800;-1,348;-0,034;2

-733;-1,717;0,091;2

-671;-1,436;-0,073;2

-608;-0,881;0,012;2

-557;-0,247;0,005;2

-515;0,073;-0,065;2

-495;-0,289;0,098;2

-497;-0,393;-0,047;2

-526;-0,277;-0,001;2

-573;-0,286;0,052;2

-637;0,369;-0,09;2

-697;1,019;0,079;2

-755;1,051;-0,028;2

-800;0,84;-0,004;2

-841;0,333;0,046;2

-873;0,018;-0,071;2

-907;-0,302;0,065;2

-938;-0,849;-0,003;2

-966;-0,756;0;2

-990;-0,651;0,072;2

-1014;-0,419;-0,072;2

-1036;-0,259;0,039;2

-1052;-0,075;0,022;2

-1069;0,221;-0,055;2

-1078;0,097;0,09;2

-1084;0,18;-0,101;2

-1090;0,887;0,005;2

-1095;2,744;0,009;2

-1107;3,108;-0,107;2

-1116;1,187;0,104;2

-1131;0,246;-0,045;2

-1144;-0,264;0,014;2

-1158;-0,617;0,049;2

-1169;-0,175;-0,076;2

-1180;0,001;0,083;2

-1184;-0,009;0,027;2

-1187;0,12;0,018;2

-1185;-0,064;0,078;2

-1177;-0,021;-0,056;2

-1161;-0,085;0,082;2

-1136;0,081;-0,013;2

-1104;0,694;-0,043;2

-1068;0,601;0,149;2

-1032;0,385;-0,069;2

-994;0,493;0,042;2

-957;0,518;0,007;2

-912;0,72;-0,043;2

-864;0,505;0,072;2

-808;0,376;-0,051;2

-751;0,403;0,009;2

-689;0,179;-0,001;2

-628;-0,014;-0,09;2

-567;-0,502;0,043;2

-516;-0,311;-0,04;2

-475;0,222;0,009;2

-464;0,133;0,055;2

-484;0,41;-0,085;2

-529;0,259;0,064;2

-589;-0,145;-0,043;2

-650;-0,235;-0,005;2

-710;-0,401;0,075;2

-760;-0,45;-0,068;2

-807;-0,486;0,061;2

-846;-0,619;0,013;2

-884;-0,25;-0,026;2

-916;-0,238;0,079;2

-948;-0,336;-0,081;2

-975;-0,763;0,011;2

-995;-1,344;0,07;2

-1015;-1,14;-0,073;2

-1024;-1,066;0,098;2

-1037;-1,121;-0,019;2

-1043;-0,778;-0,004;2

-1056;-0,244;0,038;2

-1068;-0,161;-0,096;2

-1085;-0,648;0,075;2

-1105;-0,483;-0,01;2

-1129;-0,264;-0,042;2

-1146;-0,409;0,059;2

-1160;-0,054;-0,103;2

-1166;0,153;0,04;2

-1167;-0,09;0,012;2

-1167;-0,126;-0,011;2

-1166;-0,184;0,259;2

-1166;-0,105;-0,08;2

-1163;-0,028;0,053;2

-1157;-0,311;0,005;2

-1142;-0,327;-0,039;2

-1120;-0,437;0,1;2

-1090;-0,252;-0,069;2

-1054;-0,058;0,028;2

-1006;-0,249;0,014;2

-957;-0,036;-0,049;2

-900;-0,203;0,094;2

-843;-0,243;-0,071;2

-778;0,025;0,008;2

-708;0,232;0,096;2

-633;0,404;-0,084;2

-562;0,354;0,053;2

-498;0,318;-0,007;2

-454;0,328;-0,017;2

-435;0,419;0,095;2

-446;0,669;-0,085;2

-488;0,481;0,037;2

-548;0,115;-0,007;2

-620;0,083;-0,023;2

-689;-0,065;0,053;2

-748;0,107;-0,07;2

-796;0,331;0,037;2

-836;0,468;-0,031;2

-868;0,565;-0,053;2

-895;0,23;0,079;2

-918;0,017;-0,069;2

-941;-0,221;-0,004;2

-962;-0,518;0,013;2

-982;-0,564;-0,064;2

-1000;-0,347;0,086;2

-1009;-0,324;-0,008;2

-1017;-0,235;0,008;2

-1024;-0,309;0,073;2

-1042;-0,131;-0,078;2

-1066;-0,159;0,101;2

-1098;-0,029;0,012;2

-1126;0,262;-0,013;2

-1155;0,251;0,078;2

-1169;0,547;-0,081;2

-1174;0,779;0,028;2

-1165;0,574;0,023;2

-1151;0,628;-0,056;2

-1132;0,553;0,101;2

-1119;0,53;-0,063;2

-1110;0,697;0,012;2

-1110;0,808;0,008;2

-1110;0,93;-0,079;2

-1114;0,71;0,098;2

-1109;0,302;-0,052;2

-1103;0,163;-0,019;2

-1079;-0,004;0,036;2

-1041;0,136;-0,113;2

-978;-0,024;0,073;2

-901;-0,124;-0,003;2

-813;-0,245;0,003;2

-725;-0,454;0,088;2

-643;-0,298;-0,088;2

-568;-0,674;0,053;2

-510;-0,9;0,02;2

-468;-1,092;-0,031;2

-447;-1,328;0,081;2

-456;-1,633;-0,06;2

-492;-1,685;0,055;2

-552;-1,291;-0,02;2

-621;-0,976;-0,009;2

-693;-0,998;0,073;2

-753;-0,842;-0,045;2

-806;-0,685;0,025;2

-840;-1,001;0,04;2

-868;-0,829;-0,083;2

-890;-0,737;0,022;2

-909;-0,701;-0,038;2

-933;-0,44;-0,006;2

-961;-0,563;0,059;2

-986;-0,566;-0,146;2

-1009;-0,567;0,061;2

-1031;-0,644;-0,067;2

-1044;-0,703;-0,051;2

-1059;-0,641;0,07;2

-1072;-0,534;-0,081;2

-1089;-0,405;0,049;2

-1106;-0,509;0,015;2

-1132;-0,411;-0,059;2

-1155;-0,566;0,082;2

-1175;-0,529;-0,049;2

-1185;-0,361;0,016;2

-1187;-0,48;0,006;2

-1174;-0,225;-0,075;2

-1165;-0,163;0,089;2

-1157;0,23;-0,071;2

-1153;0,424;0,014;2

-1156;0,281;0,039;2

-1162;0,444;-0,06;2

-1167;0,293;0,05;2

-1163;0,252;-0,05;2

-1151;0,336;0,017;2

-1126;0,384;0,029;2

-1092;0,316;-0,07;2

-1042;0,352;0,083;2

-983;0,333;0,021;2

-915;0,686;-0,013;2

-835;0,722;0,082;2

-754;0,67;-0,037;2

-673;0,337;0,051;2

-605;0,061;0,004;2

-551;0,01;-0,058;2

-520;-0,025;0,059;2

-510;0,036;-0,068;2

-526;0,053;0,02;2

-571;0,116;-0,033;2

-627;0,154;-0,041;2

-686;-0,266;0,091;2

-732;0,038;-0,056;2

-765;0,285;0,007;2

-787;0,3;0,042;2

-810;0,783;-0,057;2

-832;0,685;-0,016;2

-865;0,446;-0,119;2

-901;0,592;0,009;2

-948;0,776;0,07;2

-993;0,958;-0,073;2

-1033;0,842;0,054;2

-1064;0,503;-0,017;2

-1084;0,525;-0,006;2

-1091;0,318;0,086;2

-1087;0,347;-0,061;2

-1074;0,443;0,016;2

-1061;0,213;-0,046;2

-1055;0,202;-0,05;2

-1061;0,029;0,096;2

-1079;0,054;-0,081;2

-1102;0,21;0,017;2

-1134;0,052;0,042;2

-1154;0,153;-0,06;2

-1170;0,031;0,053;2

-1170;0,113;-0,061;2

-1161;0,14;0,024;2

-1145;-0,171;0,048;2

-1126;-0,053;-0,08;2

-1104;-0,253;0,065;2

-1075;-0,522;-0,024;2

-1046;-0,349;-0,004;2

-1013;-0,378;0,072;2

-976;-0,05;-0,079;2

-936;-0,045;0,057;2

-885;0,017;-0,004;2

-828;0,425;-0,04;2

-763;0,532;0,09;2

-696;0,92;-0,044;2

-626;1,185;0,029;2

-559;1,321;-0,003;2

-493;1,388;-0,069;2

-448;1,203;0,067;2

-432;0,996;-0,085;2

-453;0,967;0,018;2

-503;0,605;0,054;2

-576;0,527;-0,078;2

-646;-0,123;0,07;2

-715;-1,001;-0,118;2

-769;-1,455;-0,011;2

-815;-1,395;0,074;2

-850;-1,561;-0,096;2

-879;-1,679;0,075;2

-903;-1,615;-0,038;2

-929;-1,133;-0,025;2

-958;-0,844;0,075;2

-990;-0,583;-0,065;2

-1016;-0,615;0,057;2

-1042;-0,602;0,001;2

-1056;-0,424;-0,035;2

-1065;-0,421;0,094;2

-1075;-0,237;-0,07;2

-1082;-0,261;0,012;2

-1088;-0,052;-0,008;2

-1094;0,206;-0,084;2

-1100;-0,299;0,087;2

-1114;-0,443;-0,079;2

-1135;-0,438;-0,002;2

-1156;-0,661;0,061;2

-1174;-0,478;0,04;2

-1185;-0,478;0,061;2

-1194;-0,243;-0,015;2

-1195;-0,054;0,003;2

-1186;-0,322;0,052;2

-1160;-0,271;-0,086;2

-1119;-0,18;0,136;2

-1077;-0,284;-0,041;2

-1029;-0,387;-0,009;2

-988;-0,478;0,071;2

-951;-0,502;-0,079;2

-916;-0,494;0,027;2

-875;-0,45;0,026;2

-835;-0,397;-0,017;2

-787;-0,346;0,079;2

-735;-0,276;-0,07;2

-669;-0,056;0,013;2

-597;0,006;0,08;2

-523;0,046;-0,082;2

-458;-0,406;0,061;2

-422;-0,361;-0,041;2

-427;-0,364;-0,007;2

-470;-0,854;0,038;2

-539;-0,865;-0,06;2

-621;-0,576;0,093;2

-695;-0,128;-0,034;2

-760;-0,061;-0,037;2

-811;-0,033;0,073;2

-849;0,259;-0,07;2

-876;0,222;0,037;2

-899;-0,23;-0,011;2

-916;0,158;-0,032;2

-938;0,275;0,085;2

-959;0,271;-0,05;2

-985;0,205;0,021;2

-1010;0,189;0,029;2

-1038;0,427;-0,072;2

-1057;0,061;0,081;2

-1077;0,191;-0,071;2

-1091;0,719;-0,007;2

-1102;0,577;0,034;2

-1114;0,634;-0,05;2

-1117;0,493;0,073;2

-1120;0,252;-0,03;2

-1122;0,146;0,017;2

-1126;-0,231;0,032;2

-1129;-0,101;-0,086;2

-1138;-0,216;0,062;2

-1150;-0,115;-0,012;2

-1155;0,171;-0,047;2

-1159;-0,007;0,075;2

-1150;0,057;-0,087;2

-1129;0,314;0,079;2

-1094;0,257;0,009;2

-1052;0,623;-0,033;2

-995;0,788;0,071;2

-936;0,564;-0,068;2

-870;0,694;0,045;2

-808;0,726;0,002;2

-742;0,884;-0,008;2

-677;0,658;0,086;2

-611;0,447;-0,062;2

-555;0,548;-0,016;2

-505;0,01;0,016;2

-474;0,032;-0,09;2

-453;0,139;0,05;2

-459;0,101;0,007;2

-490;0,138;-0,021;2

-547;-0,012;0,025;2

-616;-0,003;-0,1;2

-680;-0,146;0,014;2

-737;-0,43;-0,004;2

-787;-0,144;-0,02;2

-833;-0,284;0,08;2

-875;-0,318;-0,087;2

-916;-0,043;0,058;2

-946;-0,146;-0,001;2

-973;-0,114;-0,066;2

-988;-0,091;0,13;2

-1004;0,059;-0,061;2

-1016;-0,064;-0,009;2

-1030;-0,351;0,03;2

-1045;-0,266;-0,095;2

-1061;-0,15;0,076;2

-1081;-0,234;-0,041;2

-1099;-0,198;0,03;2

-1121;-0,338;0,058;2

-1136;-0,015;-0,082;2

-1145;0,187;0,039;2

-1150;0,048;-0,016;2

-1151;-0,307;-0,007;2

-1158;-0,715;0,077;2

-1165;-0,705;-0,119;2

-1175;-1,063;0,046;2

-1178;-1,163;-0,1;2

-1182;-0,89;-0,022;2

-1172;-0,773;0,073;2

-1152;-0,644;-0,102;2

-1118;-0,443;0,038;2

-1076;-0,483;0,001;2

-1026;-0,335;-0,053;2

-970;-0,377;0,131;2

-905;-0,392;0,023;2

-838;-0,32;0,036;2

-765;-0,309;0,031;2

-694;-0,102;-0,019;2

-625;0,062;0,05;2

-559;-0,088;-0,034;2

-504;-0,064;-0,018;2

-464;-0,401;0,047;2

-452;-0,624;-0,08;2

-472;-0,47;0,07;2

-525;-0,412;0,024;2

-590;-0,181;-0,048;2

-656;-0,106;0,045;2

-708;-0,154;-0,063;2

-752;0,129;0,053;2

-787;0,291;0,016;2

-824;0,105;-0,011;2

-854;-0,423;0,099;2

-888;-0,253;-0,062;2

-927;0,435;0,021;2

-965;0,653;0,007;2

-1004;0,81;-0,131;2

-1034;0,882;0,095;2

-1052;0,922;-0,09;2

-1062;0,916;-0,001;2

-1065;0,682;0,025;2

-1070;0,864;-0,094;2

-1074;0,903;0,143;2

-1080;0,463;-0,047;2

-1085;0,443;-0,007;2

-1099;0,166;0,055;2

-1112;0,092;-0,117;2

-1130;-0,074;0,067;2

-1145;-0,323;0,02;2

-1154;-0,42;-0,037;2

-1156;-0,696;0,043;2

-1157;-0,615;-0,032;2

-1159;-0,374;0,047;2

-1156;-0,366;0,02;2

-1150;-0,339;-0,047;2

-1129;-0,669;0,067;2

-1099;-0,567;-0,076;2

-1061;-0,678;0,039;2

-1026;-0,622;0,045;2

-983;-0,206;-0,034;2

-938;-0,507;0,081;2

-881;-0,357;-0,038;2

-820;0,028;0,007;2

-757;0,1;0,035;2

-693;0,332;-0,093;2

-633;0,285;0,073;2

-571;0,254;-0,014;2

-515;0,285;-0,005;2

-466;0,146;0,066;2

-444;0,234;-0,078;2

-452;0,5;0,047;2

-492;0,518;-0,035;2

-551;0,696;-0,032;2

-620;0,712;0,083;2

-682;0,746;-0,06;2

-741;0,714;0,049;2

-793;0,646;-0,02;2

-838;1,01;-0,042;2

-879;1,123;0,101;2

-912;1,124;-0,081;2

-945;1,062;0,031;2

-975;0,78;0,015;2

-995;0,975;-0,047;2

-1016;0,516;0,062;2

-1027;0,11;-0,045;2

-1039;0,332;-0,009;2

-1050;0,237;0,067;2

-1063;0,108;-0,04;2

-1076;-0,366;0,054;2

-1094;-1,045;-0,033;2

-1109;-0,985;-0,023;2

-1125;-0,902;0,193;2

-1135;-0,918;-0,088;2

-1146;-0,623;0,039;2

-1152;-0,301;0,012;2

-1159;-0,228;-0,038;2

-1167;-0,502;0,06;2

-1173;-0,349;-0,078;2

-1180;-0,014;0,02;2

-1184;0,239;0,017;2

-1182;0,661;-0,028;2

-1171;0,523;0,067;2

-1146;0,188;-0,064;2

-1109;-0,143;0,003;2

-1072;-0,159;0,002;2

-1027;0,186;-0,124;2

-986;0,25;0,082;2

-943;0,365;-0,002;2

-897;0,751;-0,023;2

-838;1,014;0,046;2

-773;1,526;-0,105;2

-694;1,671;0,086;2

-618;1,626;-0,025;2

-543;1,721;-0,081;2

-485;1,876;0,082;2

-447;2,232;-0,072;2

-444;2,269;0,043;2

-480;1,986;0,017;2

-542;1,75;-0,012;2

-616;1,381;0,081;2

-683;0,85;-0,071;2

-741;0,69;0,011;2

-792;0,845;0,021;2

-835;0,783;-0,05;2

-876;0,353;0,077;2

-908;0,071;-0,046;2

-932;0,074;0,009;2

-948;-0,101;0,045;2

-965;0,17;-0,071;2

-980;0,016;0,073;2

-1004;-0,485;-0,042;2

-1031;-0,939;-0,037;2

-1059;-1,296;0,052;2

-1077;-1,271;-0,09;2

-1096;-1,674;0,061;2

-1106;-1,912;0,006;2

-1113;-1,875;-0,042;2

-1115;-1,755;0,128;2

-1118;-1,431;-0,089;2

-1121;-1,544;0,047;2

-1134;-1,725;0,045;2

-1151;-1,316;-0,051;2

-1164;-1,031;0,09;2

-1163;-0,745;-0,056;2

-1145;-0,524;0,014;2

-1112;-0,481;0,018;2

-1072;-0,023;-0,078;2

-1028;0,117;0,079;2

-981;0,098;-0,046;2

-927;0,356;-0,01;2

-866;0,17;0,065;2

-801;-0,299;-0,083;2

-732;-0,544;0,071;2

-664;-0,597;-0,037;2

-592;-0,326;-0,015;2

-523;-0,4;0,053;2

-461;-0,52;-0,075;2

-424;-0,711;0,064;2

-421;-0,686;-0,002;2

-452;-0,285;-0,024;2

-514;-0,137;0,047;2

-583;-0,064;-0,077;2

-655;-0,163;0,047;2

-718;-0,133;0,029;2

-773;0,089;-0,127;2

-818;0,086;0,077;2

-854;0,253;-0,058;2

-884;0,211;0,014;2

-911;0,257;0,022;2

-935;0,695;-0,084;2

-957;0,657;0,026;2

-980;0,351;-0,021;2

-998;0,1;0,02;2

-1019;-0,018;0,078;2

-1038;-0,148;-0,078;2

-1057;-0,258;0,044;2

-1074;-0,285;-0,039;2

-1092;-0,125;-0,016;2

-1103;-0,154;0,023;2

-1115;-0,047;-0,047;2

-1123;0,168;0,011;2

-1133;0,217;0,004;2

-1137;0,202;-0,027;2

-1144;0,038;0,077;2

-1152;-0,1;-0,048;2

-1156;-0,104;0,036;2

-1154;0,061;0,06;2

-1140;0,376;-0,062;2

-1111;-0,147;0,086;2

-1075;-0,605;-0,026;2

-1034;-0,54;-0,007;2

-983;-0,412;0,029;2

-927;-0,13;-0,109;2

-864;0,167;0,063;2

-805;0,137;-0,047;2

-741;-0,087;-0,004;2

-677;-0,539;0,035;2

-608;-0,505;-0,1;2

-538;-0,871;0,085;2

-473;-0,76;-0,006;2

-426;-0,494;-0,009;2

-415;-0,653;0,09;2

-439;-0,821;-0,044;2

-496;-0,978;0,014;2

-567;-0,859;0,012;2

-640;-0,691;-0,032;2

-708;-0,925;0,118;2

-765;-0,909;-0,068;2

-814;-0,657;0,026;2

-850;-0,896;0,005;2

-883;-0,984;-0,074;2

-909;-0,595;0,081;2

-938;-0,361;-0,02;2

-965;-0,542;0,003;2

-992;-0,768;0,017;2

-1014;-0,781;-0,092;2

-1035;-0,972;0,081;2

-1059;-0,996;-0,024;2

-1079;-1,096;-0,079;2

-1102;-0,948;0,08;2

-1117;-0,516;0,047;2

-1133;-0,605;0,112;2

-1143;-0,932;0,008;2

-1153;-0,532;-0,044;2

-1157;-0,6;0,067;2

-1163;-0,291;-0,06;2

-1164;0,32;0,027;2

-1161;0,173;0,02;2

-1149;0,413;-0,036;2

-1130;0,654;0,132;2

-1105;0,685;-0,091;2

-1069;0,797;0,019;2

-1021;0,839;-0,005;2

-961;1,056;-0,031;2

-898;1,091;0,063;2

-830;0,784;-0,022;2

-763;0,48;0,003;2

-692;0,255;0,049;2

-617;0,178;-0,06;2

-541;0,042;0,034;2

-475;-0,033;-0,016;2

-427;-0,087;-0,008;2

-402;-0,032;0,056;2

-418;0,051;-0,078;2

-469;0,275;0,04;2

-543;-0,066;-0,015;2

-624;-0,021;-0,036;2

-695;-0,221;0,076;2

-756;-0,115;-0,084;2

-800;0,141;0,011;2

-838;-0,109;-0,008;2

-869;0,035;-0,087;2

-899;0,001;0,052;2

-923;-0,21;-0,052;2

-949;-0,356;0,022;2

-975;-0,059;0,007;2

-1000;0,482;-0,098;2

-1026;0,464;0,042;2

-1050;0,543;-0,017;2

-1075;0,824;0,004;2

-1095;0,925;0,092;2

-1118;1,349;-0,117;2

-1130;1,365;0,086;2

-1143;1,188;0,017;2

-1148;1,389;-0,046;2

-1152;1,088;0,176;2

-1157;0,963;-0,072;2

-1167;0,936;-0,021;2

-1172;0,695;0;2

-1179;0,869;-0,094;2

-1179;0,765;0,077;2

-1173;0,621;-0,041;2

-1156;0,635;0,03;2

-1130;0,691;0,009;2

-1097;0,691;-0,04;2

-1059;0,495;0,056;2

-1014;0,444;-0,03;2

-965;0,728;0,007;2

-909;0,757;0,028;2

-843;0,882;-0,1;2

-773;0,741;0,045;2

-697;0,651;-0,033;2

-624;0,45;0,009;2

-555;0,254;0,097;2

-493;0,456;-0,076;2

-446;0,141;0,054;2

-422;-0,182;-0,008;2

-436;0,05;-0,028;2

-480;-0,113;0,07;2

-549;-0,466;-0,042;2

-626;-0,181;-0,017;2

-695;-0,122;-0,007;2

-757;-0,166;-0,046;2

-807;0,175;0,079;2

-851;0,156;-0,093;2

-884;-0,017;0,027;2

-912;0,128;0,091;2

-929;-0,067;-0,052;2

-952;-0,411;0,063;2

-972;-0,416;-0,019;2

-1000;-0,44;-0,048;2

-1024;-0,467;0,061;2

-1046;-0,291;-0,068;2

-1063;-0,267;0,064;2

-1080;-0,38;-0,016;2

-1097;-0,415;0,007;2

-1113;-0,619;0,056;2

-1126;-0,562;-0,086;2

-1140;-0,578;0,046;2

-1150;-0,376;0,013;2

-1157;0,217;-0,075;2

-1167;0,351;0,093;2

-1169;0,226;0,089;2

-1173;0,305;0,033;2

-1169;0,313;0,022;2

-1156;0,57;-0,075;2

-1134;0,568;0,068;2

-1104;0,543;-0,077;2

-1062;0,329;-0,051;2

-1016;-0,016;0,058;2

-956;-0,01;-0,072;2

-894;-0,226;0,074;2

-831;0,016;-0,055;2

-763;0,319;-0,001;2

-697;0,285;0,09;2

-625;0,434;-0,082;2

-553;0,31;0,052;2

-483;0,082;-0,019;2

-421;-0,045;-0,023;2

-375;-0,35;0,079;2

-351;-0,411;-0,027;2

-365;-0,405;0,028;2

-409;-0,568;-0,006;2

-486;-0,305;-0,054;2

-570;-0,178;0,1;2

-650;0,051;-0,069;2

-714;0,465;0,035;2

-765;0,61;-0,004;2

-803;0,825;-0,076;2

-838;0,567;0,088;2

-868;0,039;-0,079;2

-898;-0,167;0,016;2

-928;-0,69;0,157;2

-956;-1,308;-0,058;2

-985;-1,719;0,064;2

-1007;-1,863;-0,002;2

-1029;-1,514;-0,007;2

-1049;-1,267;0,066;2

-1068;-1,196;-0,081;2

-1083;-1,186;0,074;2

-1099;-1,44;0,013;2

-1107;-1,053;-0,012;2

-1121;-1,209;0,044;2

-1130;-1,135;-0,089;2

-1142;-0,539;0,054;2

-1153;-0,206;0;2

-1164;-0,116;-0,094;2

-1170;-0,261;0,061;2

-1177;0,022;-0,125;2

-1178;0,292;0,046;2

-1170;0,168;0,029;2

-1151;0,318;-0,108;2

-1116;0,359;0,088;2

-1078;0,835;-0,065;2

-1032;1,131;0,034;2

-988;0,906;0,067;2

-939;0,83;-0,087;2

-883;0,659;0,081;2

-820;0,785;-0,031;2

-748;0,797;0,049;2

-676;0,333;0,184;2

-605;0,283;-0,083;2

-538;0,617;0,05;2

-475;0,661;-0,019;2

-424;0,702;-0,025;2

-404;0,393;0,083;2

-421;0,49;-0,05;2

-474;0,149;0,034;2

-545;-0,211;0,059;2

-617;0,068;-0,043;2

-683;-0,139;0,105;2

-742;-0,377;-0,053;2

-789;-0,459;0,029;2

-833;-0,48;0,025;2

-865;-0,122;-0,099;2

-894;0,085;0,051;2

-913;0,166;-0,035;2

-938;-0,054;-0,014;2

-964;-0,613;0,061;2

-992;-0,524;-0,137;2

-1016;-0,248;0,073;2

-1040;-0,482;-0,003;2

-1062;-0,292;-0,019;2

-1081;-0,04;0,083;2

-1096;0,043;-0,04;2

-1106;-0,053;0,081;2

-1111;0,18;0,005;2

-1110;0,151;-0,067;2

-1113;-0,348;0,058;2

-1120;-0,119;-0,061;2

-1129;0,221;0,012;2

-1141;0,195;0,029;2

-1155;0,169;-0,047;2

-1162;0,173;0,082;2

-1173;-0,458;-0,052;2

-1177;-0,573;0,011;2

-1176;-0,602;0,063;2

-1164;-0,373;-0,08;2

-1135;-0,515;0,108;2

-1099;-0,5;-0,039;2

-1055;0,014;-0,017;2

-1011;0,187;0,064;2

-961;0,436;-0,072;2

-912;0,354;0,045;2

-864;0,417;0,005;2

-816;0,555;-0,032;2

-766;0,228;0,084;2

-710;0,334;-0,079;2

-644;0,344;0,043;2

-574;0,386;-0,016;2

-504;0,53;-0,018;2

-453;0,213;0,096;2

-433;0,05;-0,095;2

-450;0,326;-0,01;2

-496;0,143;0,011;2

-561;0,259;-0,077;2

-632;0,155;0,066;2

-701;0,091;-0,052;2

-770;0,067;-0,007;2

-832;-0,459;0,087;2

-887;-0,117;-0,067;2

-931;-0,222;0,049;2

-968;-0,308;-0,025;2

-992;0,134;-0,016;2

-1013;0,261;0,085;2

-1025;0,348;-0,085;2

-1032;0,446;0,067;2

-1037;0,232;-0,017;2

-1041;0,326;0,005;2

-1039;0,53;0,09;2

-1045;0,729;-0,052;2

-1051;0,895;0,03;2

-1069;0,125;0,016;2

-1087;0,348;-0,056;2

-1108;0,651;0,037;2

-1126;0,529;-0,101;2

-1143;0,55;0,006;2

-1164;0,518;-0,018;2

-1183;0,824;-0,034;2

-1202;0,569;0,07;2

-1211;0,471;-0,037;2

-1214;0,45;-0,039;2

-1213;0,086;0,055;2

-1196;0,283;-0,012;2

-1162;0,184;0,076;2

-1114;0,065;-0,046;2

-1058;0,195;-0,023;2

-1000;0,256;0,063;2

-950;0,542;-0,008;2

-897;0,663;0,049;2

-848;0,876;0,029;2

-794;1,09;-0,026;2

-737;0,954;0,073;2

-677;1,073;-0,066;2

-618;1,191;0,034;2

-554;0,904;-0,002;2

-502;0,895;-0,011;2

-460;0,743;0,101;2

-447;0,617;0,004;2

-465;0,563;0,018;2

-515;0,091;0,042;2

-579;-0,023;-0,086;2

-651;-0,178;0,063;2

-716;-0,187;-0,014;2

-774;-0,125;-0,017;2

-825;-0,484;0,067;2

-867;-0,279;-0,064;2

-898;0,033;0,07;2

-920;0,078;-0,012;2

-934;-0,099;0,022;2

-942;-0,544;0,06;2

-957;-0,933;-0,082;2

-974;-0,733;0,03;2

-998;-0,443;0,042;2

-1025;-0,326;-0,078;2

-1056;-0,411;0,105;2

-1090;-0,347;-0,044;2

-1120;-0,13;0,023;2

-1145;-0,37;0,038;2

-1161;-0,062;-0,076;2

-1170;-0,202;0,054;2

-1170;-0,471;-0,051;2

-1164;-0,465;0,014;2

-1154;-0,551;0,084;2

-1146;-0,661;-0,071;2

-1140;-0,683;0,062;2

-1138;-0,706;-0,029;2

-1144;-0,529;0,002;2

-1156;-0,76;0,062;2

-1172;-0,692;-0,049;2

-1188;-0,868;0,07;2

-1197;-1,302;-0,022;2

-1187;-0,918;-0,028;2

-1163;-0,753;0,094;2

-1123;-0,932;-0,09;2

-1079;-0,814;0,034;2

-1022;-0,607;0,003;2

-966;-0,062;-0,058;2

-905;0,092;0,101;2

-847;0,406;-0,076;2

-781;0,571;0,013;2

-714;0,384;0,025;2

-642;0,2;-0,057;2

-574;-0,073;-0,041;2

-510;0,014;-0,042;2

-458;0,165;0,028;2

-426;0,012;0,055;2

-424;0,172;-0,071;2

-459;0,524;0,051;2

-524;0,663;-0,016;2

-602;0,917;-0,048;2

-673;0,881;0,091;2

-735;1,019;-0,046;2

-784;1,012;0,043;2

-826;0,67;-0,013;2

-860;0,539;-0,045;2

-888;0,448;0,081;2

-908;0,62;-0,079;2

-928;0,73;0,017;2

-950;0,839;0,032;2

-974;1,226;-0,053;2

-999;1,179;0,121;2

-1019;1,082;-0,051;2

-1038;1,176;0,005;2

-1053;0,99;0,026;2

-1067;1,009;-0,023;2

-1081;0,775;0,098;2

-1093;0,875;-0,019;2

-1104;0,908;-0,023;2

-1112;0,468;0,058;2

-1124;0,323;-0,058;2

-1137;-0,181;0,086;2

-1152;-0,612;-0,043;2

-1164;-0,501;0,002;2

-1177;-0,769;0,07;2

-1182;-0,553;-0,065;2

-1191;-0,584;0,041;2

-1192;-0,907;0,009;2

-1191;-0,786;-0,029;2

-1175;-0,838;0,112;2

-1150;-0,756;-0,068;2

-1113;-0,749;0,032;2

-1072;-1,077;0,037;2

-1028;-0,941;-0,089;2

-985;-0,824;0,093;2

-942;-0,832;-0,1;2

-896;-0,591;0,011;2

-843;-0,351;0,024;2

-790;0,258;-0,122;2

-733;0,425;0,084;2

-673;0,369;-0,033;2

-607;0,368;-0,017;2

-542;0,54;0,075;2

-490;0,919;-0,073;2

-466;0,817;0,037;2

-482;0,626;0,022;2

-527;0,808;-0,031;2

-588;0,602;0,103;2

-646;0,514;-0,109;2

-703;0,513;0,052;2

-755;0,54;0,03;2

-796;0,775;-0,045;2

-838;0,651;0,145;2

-872;0,432;-0,061;2

-907;0,205;0,005;2

-939;0,019;0,003;2

-965;0,26;-0,063;2

-989;-0,097;0,097;2

-1010;-0,274;-0,037;2

-1030;-0,138;-0,003;2

-1050;-0,285;0,087;2

-1068;-0,323;-0,108;2

-1084;-0,677;0,081;2

-1100;-0,892;-0,03;2

-1114;-0,358;-0,045;2

-1128;-0,077;0,078;2

-1132;0,343;-0,06;2

-1137;0,659;0,173;2

-1134;0,618;0,031;2

-1138;0,689;-0,02;2

-1143;0,368;0,097;2

-1151;-0,052;-0,064;2

-1156;-0,073;0,089;2

-1169;-0,273;0,013;2

-1176;-0,477;-0,04;2

-1189;-1,015;0,09;2

-1193;-1,181;-0,077;2

-1182;-0,914;0,04;2

-1153;-1,068;0,029;2

-1114;-0,81;-0,062;2

-1066;-0,63;0,102;2

-1013;-0,599;-0,053;2

-950;-0,837;-0,055;2

-886;-1,444;0,074;2

-821;-1,627;-0,098;2

-763;-1,431;0,091;2

-704;-0,886;-0,03;2

-640;-0,817;-0,081;2

-583;-1,016;0,06;2

-524;-1,068;-0,004;2

-482;-0,957;0,045;2

-463;-1,045;-0,017;2

-474;-0,841;-0,042;2

-509;-0,83;0,08;2

-558;-0,606;-0,049;2

-613;-0,078;0,035;2

-664;-0,032;0,035;2

-716;-0,23;-0,07;2

-771;-0,763;0,089;2

-829;-0,844;-0,054;2

-879;-0,659;0,02;2

-925;-0,968;0,049;2

-963;-0,811;-0,076;2

-996;-0,865;0,047;2

-1020;-0,898;-0,016;2

-1034;-0,759;-0,027;2

-1041;-0,648;0,079;2

-1043;-0,396;-0,087;2

-1049;-0,235;0,058;2

-1059;0,028;-0,012;2

-1070;-0,011;-0,012;2

-1076;-0,356;0,105;2

-1082;-0,406;-0,091;2

-1086;-0,36;-0,041;2

-1097;-0,314;-0,018;2

-1102;-0,264;-0,052;2

-1110;-0,608;0,086;2

-1117;-0,322;0,008;2

-1129;-0,096;0,024;2

-1142;-0,595;0,029;2

-1158;-0,589;-0,049;2

-1176;-0,661;0,07;2

-1193;-0,468;-0,027;2

-1202;-0,047;0,018;2

-1206;0,18;0,037;2

-1199;0,261;-0,103;2

-1183;0,121;0,056;2

-1166;0,099;-0,01;2

-1137;0,084;0,072;2

-1108;-0,306;0,06;2

-1073;0,139;-0,096;2

-1038;0,576;0,09;2

-1005;0,877;0;2

-967;1,168;-0,021;2

-928;0,953;0,1;2

-879;0,84;-0,062;2

-821;0,98;0,038;2

-754;0,961;0,007;2

-680;0,864;-0,027;2

-605;0,54;0,091;2

-532;0,428;-0,067;2

-474;0,417;0,027;2

-443;-0,05;0,02;2

-450;-0,186;-0,061;2

-494;-0,177;0,056;2

-567;0,02;-0,05;2

-644;0,532;-0,02;2

-721;0,426;0,073;2

-782;0,544;-0,092;2

-829;0,435;0,055;2

-859;0,312;-0,018;2

-879;0,358;-0,033;2

-896;0,285;0,057;2

-912;0,485;-0,071;2

-927;0,656;0,064;2

-947;0,61;-0,087;2

-971;0,501;-0,046;2

-998;0,09;0,135;2

-1029;-0,337;-0,061;2

-1060;-0,617;0,06;2

-1088;-0,96;0,033;2

-1108;-1,34;-0,055;2

-1124;-1,471;0,125;2

-1134;-0,477;-0,094;2

-1144;-0,489;0,025;2

-1145;-0,939;0,022;2

-1142;-0,682;-0,075;2

-1136;-0,755;0,098;2

-1133;-0,752;-0,014;2

-1130;-1,097;-0,021;2

-1132;-1,299;0,058;2

-1134;-0,802;-0,091;2

-1133;-0,71;0,016;2

-1139;-0,714;-0,004;2

-1145;-0,372;-0,026;2

-1149;-0,46;0,081;2

-1153;-0,529;-0,062;2

-1151;-0,139;-0,018;2

-1145;0,026;0,01;2

-1128;0,312;-0,014;2

-1109;0,015;0,082;2

-1080;-0,596;-0,058;2

-1040;-0,501;0,02;2

-986;-0,342;0,005;2

-919;-0,348;-0,061;2

-843;-0,115;0,072;2

-771;-0,314;0,067;2

-698;-0,267;0,012;2

-636;0,047;0,029;2

-576;0,202;-0,065;2

-529;-0,176;0,072;2

-495;-0,284;-0,027;2

-486;0,134;0,056;2

-504;0,262;0,089;2

-546;0,524;-0,09;2

-603;0,264;0,075;2

-664;0,548;-0,031;2

-718;0,552;0,014;2

-762;0,444;0,062;2

-799;0,355;-0,041;2

-829;0,064;0,096;2

-857;-0,023;0,008;2

-884;0,106;-0,06;2

-913;-0,146;0,109;2

-939;-0,281;-0,062;2

-968;-0,123;0,029;2

-992;-0,09;0,025;2

-1015;0,243;-0,065;2

-1030;0,08;0,082;2

-1045;0,049;0,007;2

-1052;-0,007;0,001;2

-1062;0,001;0,029;2

-1069;-0,079;-0,079;2

-1076;-0,156;0,065;2

-1087;-0,08;0,033;2

-1101;0,105;-0,011;2

-1116;-0,231;0,06;2

-1129;-0,218;-0,093;2

-1144;-0,191;0,056;2

-1150;0,04;0,033;2

-1154;0,571;-0,004;2

-1156;0,387;0,081;2

-1153;0,458;-0,083;2

-1150;0,596;0,05;2

-1146;0,612;0,009;2

-1143;0,901;-0,073;2

-1136;0,645;0,106;2

-1131;0,558;-0,06;2

-1116;0,596;0,103;2

-1100;0,637;-0,021;2

-1076;1,025;-0,06;2

-1048;0,705;0,051;2

-1007;0,667;-0,047;2

-964;0,817;0,035;2

-914;0,402;0,048;2

-858;0,515;-0,068;2

-796;0,211;0,073;2

-724;0,099;0,005;2

-656;0,207;-0,034;2

-582;-0,023;0,077;2

-520;0,349;-0,069;2

-473;0,46;0,057;2

-455;0,548;-0,07;2

-466;0,671;-0,033;2

-512;0,803;0,089;2

-573;1,485;-0,063;2

-644;1,867;0,035;2

-706;1,498;-0,01;2

-763;1,106;-0,026;2

-809;0,812;0,099;2

-853;0,539;-0,047;2

-896;0,411;0,002;2

-932;0,119;0,025;2

-963;0,678;-0,121;2

-985;0,96;0,073;2

-998;0,669;-0,039;2

-1011;0,586;-0,021;2

-1022;0,324;0,052;2

-1036;0,486;-0,096;2

-1042;0,752;0,074;2

-1055;0,393;-0,001;2

-1071;0,474;-0,051;2

-1091;0,451;0,089;2

-1110;0,84;-0,061;2

-1128;0,794;0,054;2

-1144;0,703;-0,014;2

-1154;0,565;-0,037;2

-1167;0,137;0,044;2

-1173;0,415;-0,055;2

-1178;0,461;0,032;2

-1175;-0,01;-0,007;2

-1176;0,006;-0,076;2

-1173;0,123;0,093;2

-1173;0,411;-0,043;2

-1168;0,553;0,012;2

-1165;0,024;0,07;2

-1158;-0,682;-0,072;2

-1152;-1,197;0,045;2

-1136;-0,754;-0,024;2

-1116;-0,88;-0,017;2

-1087;-1,184;0,109;2

-1045;-0,623;-0,087;2

-992;-0,323;0,099;2

-929;-0,458;0,002;2

-859;-0,058;-0,042;2

-787;0,152;0,114;2

-714;-0,345;-0,068;2

-643;-0,367;0,028;2

-582;-0,321;0,002;2

-532;-0,293;-0,022;2

-501;-0,385;0,092;2

-495;-0,671;-0,027;2

-524;-0,551;0,028;2

-574;-0,665;0,044;2

-639;-0,551;-0,075;2

-703;-0,512;0,071;2

-751;-0,27;-0,121;2

-793;0,156;0,003;2

-827;0,2;0,055;2

-861;0,653;-0,069;2

-894;0,493;0,061;2

-925;0,213;-0,037;2

-948;0,326;0,011;2

-972;-0,018;0,045;2

-990;0,118;-0,102;2

-1010;0,184;0,07;2

-1029;-0,197;-0,032;2

-1046;-0,203;-0,036;2

-1062;-0,769;0,075;2

-1079;-0,742;-0,069;2

-1093;-0,894;0,099;2

-1107;-0,869;0,024;2

-1120;-0,532;-0,054;2

-1124;-0,426;0,113;2

-1128;-0,018;-0,074;2

-1128;0,096;-0,001;2

-1130;-0,081;0,034;2

-1129;0,051;-0,092;2

-1130;0,007;0,06;2

-1128;-0,17;-0,042;2

-1135;-0,078;0,004;2

-1140;-0,299;0,052;2

-1150;-0,276;-0,071;2

-1154;-0,449;0,061;2

-1153;-0,236;-0,036;2

-1134;0,224;0,079;2

-1110;0,084;0,14;2

-1075;0,161;-0,012;2

-1034;0,123;0,082;2

-978;0,077;0,016;2

-912;0,343;-0,054;2

-835;0,385;0,101;2

-758;0,464;-0,068;2

-684;0,522;0,003;2

-615;0,388;0,045;2

-554;0,279;-0,047;2

-500;0,099;0,103;2

-465;0,25;-0,063;2

-463;0,485;0,007;2

-502;0,103;0,003;2

-562;0,341;-0,031;2

-633;0,404;0,085;2

-699;0,442;-0,045;2

-751;0,372;0,016;2

-802;0,146;0,068;2

-841;0,245;-0,083;2

-878;0,313;0,068;2

-909;0,187;0,007;2

-935;0,314;-0,08;2

-960;0,389;0,095;2

-986;0,787;-0,044;2

-1009;0,921;0,05;2

-1031;0,694;0,036;2

-1053;0,644;-0,037;2

-1070;0,694;0,069;2

-1086;0,582;-0,04;2

-1097;0,496;0,023;2

-1111;0,349;0,047;2

-1121;0,155;-0,06;2

-1132;-0,465;0,072;2

-1139;-0,789;-0,037;2

-1148;-0,851;0,012;2

-1159;-1,355;0,069;2

-1167;-1,251;-0,075;2

-1171;-1,041;0,068;2

-1177;-0,636;-0,063;2

-1175;-0,501;-0,025;2

-1178;-0,899;0,068;2

-1179;-0,827;-0,101;2

-1178;-0,551;0,022;2

-1172;-0,517;0,018;2

-1155;-0,21;-0,024;2

-1125;-0,011;0,115;2

-1087;0,465;-0,036;2

-1043;0,768;0,019;2

-988;0,615;-0;2

-932;0,645;-0,028;2

-870;0,348;0,079;2

-804;0,048;-0,054;2

-735;-0,018;-0,05;2

-664;-0,093;0,042;2

-593;-0,311;-0,078;2

-527;-0,315;0,1;2

-478;-0,167;-0,02;2

-451;-0,018;0,017;2

-456;0,192;0,028;2

-493;0,317;-0,067;2

-549;0,119;0,095;2

-618;-0,129;-0,036;2

-682;0,34;-0;2

-739;0,562;0,044;2

-785;0,637;-0,088;2

-828;0,539;0,026;2

-866;0,365;0,003;2

-906;0,863;-0,047;2

-940;1,009;0,126;2

-975;0,949;-0,109;2

-1003;0,728;0,034;2

-1031;0,434;0,02;2

-1050;0,672;-0,101;2

-1067;0,748;0,089;2

-1074;1,005;-0,081;2

-1077;0,797;0,009;2

-1080;0,388;0,034;2

-1084;0,589;-0,073;2

-1092;0,657;0,075;2

-1106;0,322;-0,109;2

-1119;0,229;0,044;2

-1132;-0,327;0,074;2

-1149;-0,49;-0,08;2

-1162;-0,297;0,075;2

-1174;-0,217;-0,029;2

-1180;-0,278;-0,093;2

-1182;-0,291;0,064;2

-1177;-0,028;-0,062;2

-1172;0,071;0,03;2

-1164;0,099;0,009;2

-1154;-0,106;-0,007;2

-1140;-0,4;0,097;2

-1117;-0,179;-0,068;2

-1092;0,008;0,016;2

-1066;-0,027;0,004;2

-1040;0,144;-0,111;2

-1011;-0,161;0,095;2

-973;-0,365;-0,06;2

-923;-0,646;-0,004;2

-860;-1,061;0,103;2

-793;-1,113;-0,113;2

-717;-0,891;0,087;2

-642;-0,804;-0,019;2

-562;-0,759;0,021;2

-495;-0,721;0,021;2

-447;-0,449;-0,086;2

-437;-0,388;0,017;2

-459;-0,142;-0,004;2

-512;0,201;0,02;2

-579;-0,231;0,071;2

-646;-0,267;-0,079;2

-706;0,313;0,061;2

-760;0,381;0,002;2

-807;0,162;-0,052;2

-847;-0,134;0,066;2

-888;-0,662;-0,053;2

-921;-0,316;0,022;2

-950;-0,3;0,012;2

-971;-0,388;-0,056;2

-984;-1,017;0,091;2

-995;-1,332;-0,031;2

-1007;-1,363;-0,028;2

-1017;-1,925;0,074;2

-1030;-2,024;-0,077;2

-1042;-1,843;0,036;2

-1060;-1,647;-0,008;2

-1078;-1,059;0,066;2

-1098;-0,523;0,078;2

-1112;0,041;-0,082;2

-1127;0,47;0,176;2

-1140;0,253;0,004;2

-1153;0,082;-0,022;2

-1162;0,072;0,063;2

-1166;-0,207;-0,136;2

-1164;-0,345;0,051;2

-1156;-0,388;0,009;2

-1151;-0,237;-0,045;2

-1145;-0,294;0,076;2

-1145;-0,155;-0,059;2

-1141;-0,33;-0,02;2

-1130;-0,749;0,007;2

-1118;-0,831;-0,06;2

-1095;-0,88;0,075;2

-1062;-1,009;-0,062;2

-1017;-1,195;0,027;2

-956;-1,669;0,015;2

-881;-1,376;-0,086;2

-804;-1,3;0,073;2

-722;-1,489;-0,001;2

-646;-1,124;0,005;2

-576;-0,929;0,084;2

-515;-0,957;-0,051;2

-470;-0,874;0,043;2

-457;-0,822;0,016;2

-476;-0,061;-0,017;2

-529;0,526;0,085;2

-600;0,965;-0,074;2

-675;1,294;0,056;2

-740;1,287;0,018;2

-795;1,014;0,026;2

-833;0,923;0,103;2

-865;0,766;-0,042;2

-882;0,729;0,01;2

-900;0,126;0,027;2

-918;0,045;-0,101;2

-946;-0,08;0,158;2

-976;0,008;-0,021;2

-1012;-0,117;-0,001;2

-1037;-0,314;0,072;2

-1061;-0,008;-0,063;2

-1074;0,164;0,086;2

-1083;-0,113;-0,001;2

-1087;-0,111;-0,013;2

-1091;-0,195;0,08;2

-1102;0,22;-0,095;2

-1109;0,159;0,171;2

-1122;-0,349;-0,001;2

-1134;-0,319;-0,046;2

-1144;0,096;0,156;2

-1152;-0,074;-0,08;2

-1157;-0,179;0,039;2

-1162;0,279;0,034;2

-1169;0,104;-0,033;2

-1176;-0,097;0,07;2

-1177;0,061;-0,037;2

-1176;0,104;0,014;2

-1170;-0,106;0,077;2

-1163;-0,012;-0,065;2

-1149;-0,29;0,095;2

-1130;-0,024;-0,032;2

-1098;0,273;0;2

-1064;-0,283;0,083;2

-1019;0,059;-0,066;2

-969;0,394;0,098;2

-906;0,504;-0,004;2

-840;0,85;-0,025;2

-771;0,646;0,071;2

-707;0,77;-0,078;2

-647;0,863;0,03;2

-592;0,835;0,019;2

-543;0,851;-0,058;2

-511;0,737;0,054;2

-497;0,623;-0,094;2

-519;0,543;0,025;2

-559;0,393;0,006;2

-613;0,534;-0,069;2

-662;0,35;0,113;2

-706;0,083;-0,042;2

-750;0,265;-0,016;2

-792;0,157;0,039;2

-839;0,157;-0,064;2

-886;0,168;0,056;2

-935;0,401;-0,025;2

-980;0,267;-0,008;2

-1028;0,004;0,064;2

-1062;0,218;-0,118;2

-1081;0,146;0,108;2

-1084;-0,108;0,004;2

-1077;-0,078;-0,039;2

-1071;-0,436;0,096;2

-1066;-0,681;-0,044;2

-1072;-0,889;0,031;2

-1078;-1,284;0,002;2

-1095;-1,377;-0,072;2

-1110;-1,137;0,082;2

-1127;-0,647;-0,058;2

-1145;-0,641;-0,004;2

-1171;-0,397;0,043;2

-1193;0,223;-0,08;2

-1217;0,56;0,063;2

-1228;0,471;-0,059;2

-1229;0,439;0,024;2

-1218;0,458;0,071;2

-1193;0,341;-0,088;2

-1156;-0,126;0,091;2

-1110;-0,273;0,011;2

-1063;-0,046;-0,011;2

-1022;-0,357;0,14;2

-984;-0,183;-0,052;2

-942;0,278;0,053;2

-891;0,18;0,013;2

-828;0,303;-0,041;2

-760;-0,112;0,143;2

-683;-0,22;-0,057;2

-608;-0,092;0,041;2

-535;-0,221;0,019;2

-480;0,203;-0,09;2

-452;0,334;0,067;2

-463;0,534;-0,021;2

-506;1,124;0,012;2

-577;1,334;0,029;2

-651;1,119;-0,106;2

-724;0,578;0,068;2

-790;0,194;0,007;2

-840;0,358;0,027;2

-880;0,517;0,068;2

-906;0,989;-0,053;2

-926;0,871;0,081;2

-940;0,706;-0,011;2

-953;0,767;-0,023;2

-971;0,369;0,086;2

-991;0,27;-0,035;2

-1017;0,274;0,044;2

-1040;0,421;0,013;2

-1063;0,801;-0,098;2

-1082;-0,077;0,08;2

-1096;-0,579;-0,076;2

-1106;-0,759;-0,001;2

-1122;-0,925;0,005;2

-1132;-0,497;-0,069;2

-1148;-0,326;0,07;2

-1159;-0,375;-0,067;2

-1172;-0,322;0,007;2

-1179;-0,131;-0,03;2

-1185;0,003;-0,066;2

-1185;0,058;0,081;2

-1185;0,112;-0,061;2

-1179;0,272;0,006;2

-1174;0,038;0,073;2

-1162;0,282;-0,063;2

-1149;0,308;0,031;2

-1128;0,155;-0,019;2

-1108;0,276;-0,044;2

-1090;0,242;0,07;2

-1070;0,355;-0,097;2

-1047;0,41;0,049;2

-1017;0,262;-0,006;2

-978;0,501;-0,019;2

-925;0,481;0,091;2

-863;0,868;-0,043;2

-788;2,04;0,02;2

-708;2,723;0,022;2

-623;1,995;-0,058;2

-549;0,44;0,089;2

-495;-0,616;-0,051;2

-473;-0,857;-0,053;2

-490;-1,068;0,037;2

-539;-0,669;-0,103;2

-607;-0,371;0,051;2

-676;0,356;-0,001;2

-741;0,635;0,009;2

-791;-0,092;0,077;2

-831;-0,511;-0,078;2

-861;-0,662;0,072;2

-888;-0,556;-0,014;2

-912;0;-0,015;2

-941;0,336;0,081;2

-972;0,511;-0,078;2

-999;0,418;0,03;2

-1028;0,216;0,013;2

-1046;-0,208;-0,073;2

-1062;-0,318;0,08;2

-1071;0,154;-0,071;2

-1079;0,251;-0,012;2

-1083;0,072;0,054;2

-1094;0,659;-0,089;2

-1098;0,482;0,064;2

-1106;-0,164;-0,054;2

-1116;-0,428;-0,008;2

-1133;-0,272;0,071;2

-1147;0,037;-0,049;2

-1166;-0,009;0,069;2

-1178;-0,288;-0,038;2

-1186;-0,321;-0,048;2

-1188;-0,179;0,052;2

-1188;0,216;-0,096;2

-1178;0,535;0,027;2

-1162;0,337;0,001;2

-1130;0,241;-0,035;2

-1098;0,066;0,092;2

-1067;0,113;-0,045;2

-1036;0,333;-0,013;2

-995;0,155;0,035;2

-939;0,436;-0,066;2

-866;0,403;0,079;2

-779;0,064;-0,05;2

-694;-0,006;-0,004;2

-613;-0,385;0,04;2

-546;-0,544;-0,095;2

-498;-0,872;0,052;2

-475;-0,817;-0,049;2

-477;-0,447;-0,018;2

-506;-0,064;0,035;2

-558;0,329;-0,11;2

-622;0,557;0,101;2

-694;0,39;0,011;2

-757;0,431;-0,039;2

-811;0,29;0,079;2

-853;0,119;-0,072;2

-883;-0,172;0,016;2

-909;-0,786;0,029;2

-933;-0,878;-0,052;2

-952;-1,022;0,081;2

-973;-1,211;-0,116;2

-996;-1,433;-0,002;2

-1021;-1,624;0,08;2

-1045;-1,147;-0,075;2

-1063;-0,98;0,047;2

-1079;-1,094;-0,068;2

-1087;-0,962;0,02;2

-1098;-0,457;0,071;2

-1105;0,021;-0,122;2

-1117;-0,328;0,06;2

-1134;-0,231;-0,014;2

-1153;-0,198;-0,034;2

-1167;-0,562;0,085;2

-1181;-0,039;-0,064;2

-1181;0,364;0,036;2

-1181;0,445;0,013;2

-1170;0,281;-0,072;2

-1162;0,204;0,14;2

-1149;0,422;-0,069;2

-1130;0,364;0,029;2

-1101;0,197;0,042;2

-1068;0,358;-0,014;2

-1025;0,443;0,1;2

-975;0,471;-0,025;2

-924;0,592;-0,005;2

-866;0,373;0,033;2

-806;0,347;-0,054;2

-736;0,453;0,067;2

-656;0,391;-0,054;2

-571;0,305;0,003;2

-495;0,254;0,046;2

-444;0,692;-0,142;2

-423;0,777;0,097;2

-448;0,606;-0,013;2

-501;0,54;0,003;2

-578;0,206;0,1;2

-663;0,19;-0,05;2

-746;0,305;0,034;2

-813;0,316;0,006;2

-866;0,183;-0,046;2

-899;0,011;0,085;2

-923;0,416;-0,069;2

-937;0,704;-0,017;2

-950;0,332;0,014;2

-959;0,478;-0,071;2

-969;0,474;0,093;2

-984;0,492;-0,067;2

-1007;0,191;-0;2

-1037;-0,123;0,036;2

-1070;-0,044;-0,055;2

-1105;-0,181;0,056;2

-1130;-0,431;-0,039;2

-1152;-0,408;-0,003;2

-1163;-0,927;0,064;2

-1172;-1,043;-0,096;2

-1173;-0,695;0,07;2

-1172;-0,644;0,018;2

-1168;-0,588;-0,013;2

-1166;-0,578;0,078;2

-1162;-0,206;-0,034;2

-1166;0,192;0,045;2

-1170;-0,03;-0,023;2

-1181;-0,111;-0,053;2

-1184;0,106;-0,005;2

-1179;-0,095;-0,107;2

-1168;-0,127;0,051;2

-1144;-0,007;0,064;2

-1116;-0,225;-0,008;2

-1069;-0,059;0,088;2

-1012;0,32;-0,045;2

-947;0,226;-0,03;2

-873;0,476;0,084;2

-795;0,87;-0,068;2

-712;-0,027;0,079;2

-636;0,306;-0,045;2

-566;0,78;-0,085;2

-519;0,721;0,07;2

-493;0,53;-0,089;2

-495;0,416;0,051;2

-527;0,619;-0,012;2

-587;0,702;-0,029;2

-658;0,27;0,091;2

-725;-0,045;-0,072;2

-784;-0,495;0,045;2

-827;-1,025;0,002;2

-865;-1,928;-0,071;2

-895;-2,636;0,07;2

-920;-2,279;-0,056;2

-939;-1,286;0,019;2

-960;-0,619;0,098;2

-980;-0,155;0,036;2

-1005;-0,333;0,084;2

-1028;-0,121;-0,045;2

-1052;0,098;0,009;2

-1071;0,037;0,057;2

-1088;-0,203;-0,083;2

-1102;-0,315;0,082;2

-1115;-0,374;-0,019;2

-1125;-0,52;-0,032;2

-1132;-0,925;0,093;2

-1142;-0,831;-0,078;2

-1153;-0,669;0,044;2

-1167;-0,699;0,045;2

-1178;-0,32;-0,054;2

-1184;-0,253;0,079;2

-1179;-0,001;-0,064;2

-1174;0,387;0,032;2

-1170;0,105;-0,014;2

-1161;0,308;0,002;2

-1148;0,382;0,104;2

-1122;0,229;-0,064;2

-1092;0,137;0,01;2

-1054;0,041;0,03;2

-1012;0,447;-0,091;2

-955;0,277;0,063;2

-889;-0,294;-0,025;2

-812;-0,512;0,042;2

-736;-0,548;0,065;2

-661;-0,081;-0,009;2

-597;0,639;0,165;2

-542;0,394;-0,03;2

-504;0,428;-0,029;2

-487;0,321;0,059;2

-503;0,304;-0,086;2

-551;0,44;0,009;2

-613;0,746;0,026;2

-678;0,755;-0,085;2

-734;0,538;0,097;2

-778;0,547;-0,028;2

-816;0,345;0,016;2

-852;0,121;-0,002;2

-886;0,034;-0,078;2

-917;-0,374;0,04;2

-947;-0,596;-0,039;2

-976;-1,007;0,015;2

-1004;-1,633;0,027;2

-1028;-1,405;-0,068;2

-1050;-0,68;0,039;2

-1067;-0,5;0,008;2

-1075;-0,63;-0,007;2

-1083;-0,554;0,074;2

-1082;0,024;-0,052;2

-1086;-0,138;0,038;2

-1090;-0,541;0,014;2

-1102;-0,474;-0,016;2

-1118;-0,609;0,077;2

-1140;-0,385;-0,093;2

-1164;-0,011;0,023;2

-1184;0,183;-0;2

-1196;0,655;-0,052;2

-1195;0,614;0,088;2

-1186;0,567;-0,082;2

-1164;0,673;0,029;2

-1137;0,588;-0,006;2

-1097;0,677;-0,058;2

-1062;0,193;0,093;2

-1030;-0,161;-0,054;2

-1001;-0,279;0,013;2

-972;-0,403;0,152;2

-932;-0,652;-0,097;2

-881;-0,417;0,079;2

-821;-0,264;-0,025;2

-755;-0,399;-0,013;2

-689;-0,429;0,049;2

-616;-0,107;-0,086;2

-541;0,17;0,088;2

-479;0,228;0,018;2

-442;0,361;0,033;2

-446;0,446;0,085;2

-482;0,212;-0,067;2

-545;-0,147;0,04;2

-618;-0,634;0,011;2

-694;-0,542;-0,052;2

-765;-0,618;0,068;2

-832;-0,846;-0,082;2

-880;-0,686;0,03;2

-915;-0,374;0,022;2

-937;-0,254;-0,015;2

-954;-0,125;0,094;2

-962;0,372;-0,035;2

-974;0,861;0,01;2

-984;0,67;0,041;2

-1000;0,423;-0,083;2

-1021;0,426;0,071;2

-1053;0,6;-0,012;2

-1080;0,708;-0,006;2

-1107;0,44;0,098;2

-1123;0,663;-0,041;2

-1130;0,985;0,016;2

-1135;0,606;-0,035;2

-1134;0,677;0,008;2

-1141;0,418;0,066;2

-1143;0,527;-0,077;2

-1149;0,566;0,031;2

-1156;0,259;0,05;2

-1164;0,906;-0,12;2

-1171;0,706;0,082;2

-1175;0,5;-0,055;2

-1174;0,847;-0,02;2

-1161;0,386;0,042;2

-1137;0,232;-0,087;2

-1108;0,562;0,088;2

-1070;0,717;-0,08;2

-1024;0,475;0,016;2

-972;0,24;0,07;2

-904;0,177;-0,056;2

-836;-0,079;0,068;2

-764;-0,014;-0,032;2

-700;0,052;0,026;2

-637;-0,394;0,075;2

-583;-0,651;-0,053;2

-533;-0,707;0,041;2

-505;-0,943;0,014;2

-497;-0,863;-0,059;2

-523;-1,189;0,083;2

-566;-1,278;-0,063;2

-624;-1,015;0,067;2

-683;-0,661;0,036;2

-742;-0,344;-0,056;2

-794;-0,089;0,053;2

-843;0,32;-0,09;2

-880;0,773;0,011;2

-912;0,647;0,051;2

-929;0,203;-0,054;2

-946;0,026;0,074;2

-962;0,019;-0,036;2

-979;-0,048;0,015;2

-1004;0,053;0,043;2

-1028;0,063;-0,067;2

-1056;0,266;0,051;2

-1081;0,172;0,016;2

-1099;0,104;-0,004;2

-1113;0,122;0,068;2

-1122;0,34;-0,081;2

-1125;0,295;0,023;2

-1125;0,271;0,029;2

-1125;0,131;-0,054;2

-1123;-0,286;0,081;2

-1127;-0,454;-0,067;2

-1131;-0,662;0,013;2

-1140;-1,15;0,026;2

-1149;-0,927;-0,155;2

-1161;-0,86;0,046;2

-1173;-1,005;-0,029;2

-1180;-0,846;-0,016;2

-1172;-0,652;0,071;2

-1144;-0,817;-0,079;2

-1101;-0,814;0,132;2

-1045;-0,735;-0,012;2

-983;-0,551;-0,007;2

-915;-0,59;0,062;2

-852;-0,292;-0,053;2

-784;-0,279;0,04;2

-720;-0,271;0,005;2

-660;0,302;-0,016;2

-611;0,717;0,081;2

-568;0,504;-0,04;2

-540;-0,157;0,029;2

-528;-0,284;-0,039;2

-545;-0,567;-0,071;2

-583;-0,796;0,061;2

-638;-0,654;-0,075;2

-688;-0,504;0,016;2

-737;-0,074;0,014;2

-772;0,155;-0,081;2

-809;0,292;0,075;2

-849;0,069;-0,044;2

-890;0,283;0,045;2

-931;1,084;0,062;2

-966;1,335;-0,067;2

-997;1,298;0,064;2

-1020;1,393;-0,02;2

-1039;1,449;-0,055;2

-1051;0,899;0,063;2

-1059;0,753;-0,065;2

-1064;0,966;0,034;2

-1068;0,875;0,022;2

-1079;0,681;-0,023;2

-1092;0,736;0,093;2

-1109;0,596;-0,068;2

-1119;0,268;0,031;2

-1132;0,033;0,018;2

-1138;-0,09;-0,072;2

-1149;-0,132;0,1;2

-1156;-0,237;-0,049;2

-1162;-0,472;-0,006;2

-1164;-0,901;0,081;2

-1164;-0,957;-0,102;2

-1163;-1,296;0,036;2

-1160;-1,402;-0,036;2

-1149;-1,003;0,002;2

-1125;-0,779;0,041;2

-1091;-0,519;-0,087;2

-1050;-0,429;0,067;2

-1010;-0,571;0,006;2

-960;-0,134;-0,029;2

-906;-0,135;0,084;2

-841;0,027;-0,056;2

-772;-0,219;0,051;2

-699;-0,838;0,008;2

-632;-0,734;-0,04;2

-565;-0,622;0,071;2

-510;-0,284;-0,085;2

-470;-0,002;0,023;2

-464;0,137;0,014;2

-490;0,376;-0,102;2

-541;0,126;0,068;2

-600;0,13;-0,075;2

-657;0,385;-0,004;2

-708;-0,02;0,032;2

-750;-0,055;-0,107;2

-791;-0,147;0,045;2

-827;-0,058;-0,033;2

-866;0,265;-0,001;2

-900;0,439;0,046;2

-932;0,413;-0,073;2

-958;-0,101;0,069;2

-973;-0,099;-0,006;2

-988;-0,203;0,067;2

-995;-0,761;0,093;2

-1007;-0,526;-0,042;2

-1015;-0,504;0,006;2

-1029;-1,159;0,021;2

-1038;-1,034;-0,062;2

-1052;-1,007;0,085;2

-1066;-1,085;-0,063;2

-1086;-0,643;0,002;2

-1106;-0,536;0,038;2

-1124;-0,173;-0,087;2

-1134;-0,316;0,088;2

-1140;-0,185;-0,024;2

-1141;0,031;0,008;2

-1140;-0,249;0,072;2

-1137;0,077;-0,121;2

-1128;0,338;0,209;2

-1120;0,403;0,046;2

-1112;0,575;-0,014;2

-1104;0,375;0,054;2

-1091;0,082;-0,062;2

-1073;-0,343;0,041;2

-1041;-0,711;-0,013;2

-1005;-0,245;-0,034;2

-962;0,365;0,072;2

-913;0,922;-0,069;2

-849;1,205;0,031;2

-780;1,294;0,062;2

-707;0,788;-0,073;2

-637;0,364;0,101;2

-575;0,308;-0,008;2

-526;0,256;0,016;2

-490;0,346;0,053;2

-472;0,166;-0,149;2

-484;-0,132;0,076;2

-521;0,41;-0,043;2

-581;0,858;0,022;2

-646;0,252;0,072;2

-706;0,049;-0,061;2

-752;0,27;0,079;2

-782;0,078;0,006;2

-810;-0,044;-0,01;2

-835;-0,563;0,085;2

-862;-0,558;-0,074;2

-888;-0,782;0,056;2

-916;-0,826;0,006;2

-943;-0,157;-0,042;2

-973;0,167;0,07;2

-998;-0,286;-0,077;2

-1025;-0,518;0,027;2

-1041;-0,659;0,007;2

-1056;-0,712;-0,105;2

-1063;-1,161;0,1;2

-1073;-0,708;-0,061;2

-1079;-0,573;0,058;2

-1084;-0,862;0,057;2

-1088;-0,287;-0,094;2

-1096;-0,002;0,058;2

-1104;-0,114;-0,089;2

-1116;0,482;-0,017;2

-1126;0,576;0,086;2

-1136;0,36;-0,061;2

-1144;0,348;0,077;2

-1148;-0,285;0,023;2

-1137;-0,307;-0,014;2

-1114;-0,622;0,039;2

-1081;-0,806;-0,067;2

-1044;-0,352;0,117;2

-1003;-0,173;0,011;2

-956;0,167;-0,095;2

-904;0,173;0,067;2

-844;0,229;-0,067;2

-779;0,392;0,032;2

-709;0,335;0,029;2

-640;0,554;-0,113;2

-572;0,324;0,08;2

-513;0,464;-0,061;2

-473;0,854;0,011;2

-462;0,598;0,057;2

-490;0,531;-0,092;2

-545;0,476;0,132;2

-616;0,818;0,013;2

-684;0,816;0,012;2

-745;0,634;0,056;2

-787;0,674;-0,046;2

-822;0,61;0,056;2

-848;0,646;0,044;2

-865;0,839;-0,028;2

-880;0,741;0,089;2

-896;0,876;-0,089;2

-920;0,983;0,032;2

-952;1,027;0,049;2

-988;1,54;-0,085;2

-1018;1,625;0,058;2

-1046;1,437;-0,038;2

-1063;1,573;-0,005;2

-1081;1,364;-0,048;2

-1089;1,225;-0,082;2

-1093;1,451;0,042;2

-1087;1,253;-0,019;2

-1084;1,14;-0,008;2

-1080;0,853;0,062;2

-1085;0,672;-0,024;2

-1093;0,195;0,056;2

-1106;-0,252;-0,012;2

-1118;-0,135;-0,035;2

-1130;-0,397;0,089;2

-1138;-0,403;-0,073;2

-1141;-0,57;0,05;2

-1132;-1,257;0,001;2

-1113;-1,255;-0,053;2

-1082;-1,145;0,084;2

-1047;-1,363;0,008;2

-1009;-2,154;0,033;2

-964;-2,499;0,009;2

-919;-2,216;-0,058;2

-868;-1,661;0,076;2

-808;-0,974;-0,073;2

-748;-0,924;0,016;2

-684;-0,949;0,103;2

-624;-0,271;-0,039;2

-562;-0,117;0,054;2

-507;-0,003;0,001;2

-467;0,209;-0,009;2

-451;0,172;0,075;2

-466;0,69;-0,093;2

-509;1,078;0,065;2

-572;0,898;-0,01;2

-635;0,994;-0,013;2

-698;0,59;0,099;2

-749;0,187;-0,038;2

-797;0,074;0,02;2

-836;-0,155;-0,046;2

-869;-0,296;-0,076;2

-897;-0,531;0,083;2

-922;-0,209;-0,056;2

-946;0,238;0,012;2

-970;0,105;0,025;2

-992;0,714;-0,089;2

-1011;0,633;0,082;2

-1031;0,032;-0,044;2

-1043;-0,067;0,008;2

-1057;-0,311;0,054;2

-1065;-0,156;-0,072;2

-1077;0,087;0,087;2

-1086;0,094;-0,03;2

-1099;0,551;-0,033;2

-1112;0,749;0,072;2

-1127;0,811;-0,065;2

-1134;0,555;0,006;2

-1139;0,022;-0,002;2

-1135;0,23;-0,035;2

-1132;0,173;0,071;2

-1127;0,042;-0,077;2

-1123;-0,042;0,045;2

-1120;-0,521;0,006;2

-1105;-0,627;-0,035;2

-1086;-0,325;0,076;2

-1055;-0,171;-0,07;2

-1015;0,117;0,029;2

-969;0,192;0,054;2

-916;0,111;-0,087;2

-859;0,144;0,07;2

-797;0,51;-0,053;2

-732;0,315;0,019;2

-666;0,09;0,087;2

-604;0,084;-0,062;2

-542;0,166;0,068;2

-498;0,227;-0,02;2

-474;0,178;-0,023;2

-485;0,315;0,092;2

-523;0,28;-0,101;2

-586;0,37;0,021;2

-655;0,318;0,014;2

-726;0,478;-0,05;2

-783;0,833;0,072;2

-828;1,18;-0,051;2

-862;0,757;0,05;2

-891;0,423;0,019;2

-913;0,582;-0,085;2

-938;0,545;0,073;2

-954;0,381;-0,03;2

-974;0,29;-0,002;2

-995;0,231;0,038;2

-1019;0,58;-0,083;2

-1038;0,382;0,063;2

-1056;0,322;-0,016;2

-1065;0,498;0,004;2

-1076;0,454;0,094;2

-1087;0,09;-0,078;2

-1097;0,037;0,054;2

-1103;-0,301;0,003;2

-1108;-0,328;-0,028;2

-1111;-0,445;0,08;2

-1120;-0,254;-0,112;2

-1130;-0,201;0,078;2

-1137;-0,124;0,009;2

-1145;0,335;-0,046;2

-1147;0,439;0,131;2

-1148;0,8;-0,091;2

-1137;0,507;0,027;2

-1121;0,06;0,036;2

-1093;0,364;-0,063;2

-1054;0,682;0,083;2

-1009;0,407;-0,04;2

-956;0,311;0,001;2

-899;-0,1;0,062;2

-832;-0,04;-0,082;2

-763;-0,278;0,052;2

-694;-1,03;0,025;2

-629;-1,047;-0,017;2

-568;-0,603;0,078;2

-522;-0,343;-0,043;2

-491;0,029;0,068;2

-487;0,043;0,014;2

-513;0,574;-0,052;2

-564;0,608;0,098;2

-623;0,377;-0,088;2

-684;0,127;0,061;2

-738;0,082;0,02;2

-784;0,421;-0,07;2

-826;0,427;0,097;2

-866;0,74;-0,069;2

-898;0,715;0,029;2

-931;0,269;0,019;2

-961;0,136;-0,022;2

-986;-0,149;0,05;2

-1010;-0,134;-0,036;2

-1025;0,119;0,028;2

-1034;-0,09;0,041;2

-1043;-0,193;-0,062;2

-1054;-0,135;0,061;2

-1068;-0,274;-0,009;2

-1087;-0,299;-0,056;2

-1104;-0,03;0,074;2

-1121;-0,002;-0,056;2

-1131;-0,394;0,051;2

-1141;-0,719;0,029;2

-1145;-0,944;-0,057;2

-1149;-1,452;0,11;2

-1151;-1,757;-0,021;2

-1154;-1,87;0,051;2

-1164;-1,314;0,052;2

-1170;-0,668;-0,054;2

-1175;-0,846;0,095;2

-1164;-0,903;-0,052;2

-1141;-0,678;0,021;2

-1102;-1,137;0,027;2

-1056;-1,495;-0,104;2

-1005;-1,585;0,035;2

-952;-1,297;-0,031;2

-897;-0,541;0,003;2

-840;-0,492;0,121;2

-780;-0,556;-0,091;2

-721;-0,341;0,074;2

-662;0,105;-0,062;2

-608;0,257;-0,01;2

-558;0,547;0,067;2

-521;0,685;-0,064;2

-508;0,162;0,015;2

-526;-0,072;0,026;2

-577;-0,201;-0,072;2

-640;-0,613;0,094;2

-707;-0,449;0,034;2

-763;0,011;0,021;2

-813;0,348;0,022;2

-853;0,636;-0,083;2

-889;0,5;0,061;2

-917;0,541;-0,016;2

-943;0,52;0,092;2

-962;0,136;0,022;2

-983;0,15;-0,09;2

-1004;0,094;0,099;2

-1026;-0,096;-0,018;2

-1043;-0,37;-0,007;2

-1061;-1,109;0,058;2

-1071;-1,218;-0,08;2

-1082;-1,092;0,058;2

-1089;-0,797;0;2

-1093;-0,309;-0,013;2

-1099;0,071;0,098;2

-1102;0,334;-0,063;2

-1115;0,764;0,021;2

-1127;0,641;-0,005;2

-1147;0,876;-0,061;2

-1167;0,818;0,068;2

-1178;0,926;-0,061;2

-1185;0,615;-0,009;2

-1178;0,009;-0,049;2

-1162;-0,081;-0,066;2

-1134;-0,238;0,071;2

-1100;-0,051;-0,018;2

-1059;0,161;0,007;2

-1018;-0,195;0,055;2

-971;-0,218;-0,082;2

-923;-0,264;0,092;2

-870;-0,761;-0,01;2

-814;-0,298;-0,033;2

-751;0,111;0,148;2

-685;0,231;-0,061;2

-619;0,725;0,135;2

-565;0,518;-0,002;2

-531;0,3;-0,056;2

-523;0,263;0,071;2

-547;0,272;-0,04;2

-594;0,262;0,031;2

-652;0,06;-0,018;2

-714;0,081;-0,074;2

-773;0,085;0,083;2

-823;0,375;-0,068;2

-870;0,457;-0,018;2

-907;0,063;-0,024;2

-937;0,419;-0,04;2

-963;0,158;0,063;2

-985;0,303;-0,001;2

-1002;0,898;-0,003;2

-1021;0,729;0,062;2

-1035;0,607;-0,12;2

-1048;0,362;0,065;2

-1058;-0,292;-0,033;2

-1065;-0,428;-0,022;2

-1076;-0,359;0,073;2

-1087;-0,301;-0,058;2

-1104;-0,122;0,09;2

-1119;0,322;0,006;2

-1135;0,423;-0,033;2

-1149;-0,008;0,127;2

-1158;-0,512;-0,096;2

-1167;-0,89;-0,057;2

-1169;-0,768;0,042;2

-1171;-0,506;-0,068;2

-1168;-0,357;0,062;2

-1166;-0,209;-0,043;2

-1155;0,402;0,007;2

-1137;0,396;0,028;2

-1108;0,326;-0,067;2

-1077;0,098;0,123;2

-1037;0,301;-0,017;2

-994;0,342;-0,026;2

-941;0,391;0,083;2

-885;0,773;-0,096;2

-820;0,491;0,09;2

-754;0,267;-0;2

-689;0,513;0,001;2

-628;0,171;0,085;2

-570;-0,05;-0,066;2

-522;-0,077;0,031;2

-496;-0,119;0,012;2

-502;0,072;-0,066;2

-548;-0,08;0,101;2

-612;-0,105;-0,062;2

-684;0,087;0,001;2

-745;0,021;0,021;2

-794;0,097;-0,043;2

-836;0,002;0,124;2

-876;0,019;-0,029;2

-905;0,16;-0,131;2

-936;0,249;0,016;2

-958;0,062;-0,096;2

-986;-0,192;0,071;2

-1013;-0,113;-0,01;2

-1038;0,171;-0,016;2

-1061;0,107;0,077;2

-1074;0,183;-0,052;2

-1084;0,096;0,069;2

-1088;0,162;0,013;2

-1095;0,465;-0,018;2

-1105;0,208;0,059;2

-1114;0,097;-0,03;2

-1126;0,118;0,042;2

-1137;-0,178;0,081;2

-1148;-0,205;-0,076;2

-1155;-0,209;0,098;2

-1162;0,185;-0,044;2

-1161;0,148;-0,009;2

-1164;0,105;0,114;2

-1164;0,381;-0,035;2

-1169;0,184;0,11;2

-1170;-0,226;-0,066;2

-1158;-0,557;-0,02;2

-1135;-0,965;0,066;2

-1106;-1,111;-0,08;2

-1068;-0,492;0,127;2

-1026;0,402;0,02;2

-978;0,949;0,012;2

-925;0,668;0,081;2

-866;0,206;-0,052;2

-804;0,049;0,05;2

-739;-0,346;0,007;2

-670;-0,893;-0,033;2

-605;-1,389;0,116;2

-545;-1,151;-0,108;2

-509;-0,187;-0,018;2

-498;0,039;0,035;2

-518;0,51;-0,05;2

-568;0,907;0,097;2

-634;0,735;-0,045;2

-702;0,706;0,003;2

-770;0,912;0,049;2

-820;1,14;-0,088;2

-860;0,769;0,072;2

-887;0,524;0,015;2

-911;0,542;-0,017;2

-935;-0,074;0,075;2

-958;-0,353;-0,082;2

-986;-0,254;0,043;2

-1009;-0,234;0,01;2

-1032;-0,114;-0,009;2

-1056;0,209;0,099;2

-1077;0,655;-0,042;2

-1096;0,518;0,042;2

-1110;-0,196;0,004;2

-1120;-0,323;-0,041;2

-1128;-0,179;0,094;2

-1135;0,116;-0,072;2

-1136;0,526;0,075;2

-1143;0,183;0,001;2

-1144;-0,085;-0,06;2

-1149;-0,106;0,096;2

-1157;0,218;-0,039;2

-1168;0,683;0,005;2

-1176;0,452;0,038;2

-1188;-0,009;-0,111;2

-1192;0,005;0,063;2

-1195;0,286;-0,039;2

-1184;0,585;-0,017;2

-1161;0,363;0,034;2

-1121;0,274;-0,048;2

-1071;-0,312;0,035;2

-1017;-1,534;0,016;2

-961;-1,616;-0,02;2

-906;-1,592;0,076;2

-851;-1,251;-0,054;2

-794;-0,964;0,019;2

-740;-0,859;0,01;2

-684;-0,269;-0,11;2

-629;-0,188;0,106;2

-580;-0,158;-0,081;2

-536;-0,167;0,002;2

-515;-0,578;0,019;2

-520;-0,094;-0,06;2

-552;-0,128;0,058;2

-603;-0,571;-0,03;2

-663;-0,321;0;2

-720;-0,095;0,06;2

-774;0,001;-0,103;2

-825;0,403;0,104;2

-869;1,076;-0,011;2

-911;0,457;-0,007;2

-940;-0,606;0,048;2

-966;-0,259;-0,07;2

-988;0,215;0,026;2

-1009;-0,569;-0,002;2

-1028;-0,14;-0,023;2

-1048;-0,228;0,076;2

-1071;-0,372;-0,073;2

-1091;-0,229;-0,018;2

-1108;-0,071;0,018;2

-1119;0,417;-0,05;2

-1125;0,316;0,072;2

-1130;0,07;-0,053;2

-1138;0,293;-0,029;2

-1149;0,255;-0,008;2

-1161;0,315;-0,061;2

-1170;0,209;0,075;2

-1179;0,083;-0,042;2

-1180;-0,095;-0,023;2

-1183;-0,055;0,053;2

-1182;0,018;-0,014;2

-1185;0,165;0,067;2

-1189;-0,078;-0,026;2

-1192;-0,217;-0,042;2

-1185;-0,034;0,081;2

-1171;0,146;-0,058;2

-1140;-0,209;0,031;2

-1105;-0,37;-0,004;2

-1057;0,072;-0,051;2

-1010;-0,011;0,091;2

-957;0,112;-0,048;2

-913;0,419;0,018;2

-863;0,333;0,032;2

-812;0,403;-0,083;2

-751;0,589;0,069;2

-681;1,048;-0,059;2

-613;1,378;0,033;2

-551;0,692;0,046;2

-517;0,846;-0,059;2

-510;0,966;0,053;2

-538;0,474;-0,051;2

-588;0,461;-0,001;2

-648;0,222;0,099;2

-713;0,224;-0,062;2

-765;0,104;0,057;2

-808;0,171;-0,021;2

-843;0,607;-0,015;2

-877;0,407;0,102;2

-907;0,417;-0,056;2

-942;0,534;0,054;2

-968;0,628;0,038;2

-991;0,635;-0,065;2

-1004;0,082;0,198;2

-1022;-0,609;-0,032;2

-1038;-0,485;-0,005;2

-1050;-0,733;-0,012;2

-1062;-0,627;-0,07;2

-1074;-0,781;0,05;2

-1082;-1,098;-0,031;2

-1091;-0,83;0,018;2

-1102;-0,742;0,088;2

-1114;-0,401;-0,098;2

-1124;-0,376;0,053;2

-1132;-0,372;0,005;2

-1140;0,173;-0,026;2

-1145;0,468;0,081;2

-1151;0,305;-0,059;2

-1148;-0,068;0,056;2

-1137;-0,148;-0,013;2

-1114;0,004;-0,085;2

-1087;0,031;0,089;2

-1052;0,732;-0,057;2

-1018;1,05;0,051;2

-972;0,452;0,045;2

-919;-0,153;-0,052;2

-856;-0,6;0,094;2

-791;-0,776;-0,053;2

-722;-0,151;0,013;2

-653;0,067;-0,013;2

-592;0,328;-0,071;2

-538;0,402;0,159;2

-505;0,414;-0,026;2

-501;0,225;0,022;2

-531;-0,327;0,053;2

-581;-0,269;-0,106;2

-644;-0,462;0,084;2

-710;-0,46;-0,037;2

-766;-0,691;-0,029;2

-812;-0,87;0,049;2

-849;-0,464;-0,065;2

-879;-0,039;0,049;2

-903;-0,298;-0,022;2

-929;-0,212;-0,075;2

-951;-0,065;0,087;2

-972;0,32;-0,065;2

-992;0,564;0,022;2

-1016;0,686;0,021;2

-1038;0,844;-0,059;2

-1063;0,82;0,11;2

-1080;0,933;0,043;2

-1096;0,717;0,005;2

-1109;0,228;0,061;2

-1118;0,075;-0,081;2

-1125;-0,089;0,1;2

-1126;-0,609;-0,013;2

-1125;-0,558;-0,022;2

-1126;-1,022;0,078;2

-1127;-0,92;-0,059;2

-1135;-0,684;0,069;2

-1145;-0,544;-0,004;2

-1153;-0,243;-0,019;2

-1162;-0,03;0,089;2

-1164;-0,072;-0,082;2

-1168;0,07;0,053;2

-1158;-0,384;0,03;2

-1137;-0,414;-0,036;2

-1102;-0,572;0,072;2

-1058;-0,369;-0,071;2

-1007;0,146;0,091;2

-954;0,214;0,025;2

-900;0,253;-0,072;2

-844;0,2;0,069;2

-789;0,266;-0,056;2

-728;0,294;0,04;2

-669;0,096;0,037;2

-612;0,049;-0,072;2

-561;0,033;0,07;2

-526;0,371;-0,009;2

-515;0,381;-0,005;2

-536;-0,049;0,065;2

-582;0,263;-0,099;2

-645;0,398;0,046;2

-705;0,522;-0,015;2

-761;0,864;-0,021;2

-807;0,454;0,071;2

-852;0,616;-0,037;2

-891;0,427;0,034;2

-930;0,359;0,006;2

-960;0,404;-0,079;2

-988;0,309;0,071;2

-1006;0,748;-0,082;2

-1024;0,821;0,015;2

-1037;0,405;0,027;2

-1046;0,282;-0,053;2

-1057;-0,103;0,087;2

-1068;-0,147;-0,041;2

-1080;0,229;-0,022;2

-1098;0,599;0,063;2

-1118;1,14;-0,093;2

-1138;1,105;0,075;2

-1156;0,949;-0,035;2

-1169;1,249;-0,01;2

-1174;0,775;0,085;2

-1171;0,816;-0,122;2

-1167;0,827;0,058;2

-1158;0,638;0,025;2

-1152;0,888;-0,063;2

-1148;0,593;0,102;2

-1143;0,752;-0,089;2

-1131;1,307;0,062;2

-1113;1,131;0,016;2

-1087;1,004;0,053;2

-1052;0,852;0,095;2

-1007;0,751;-0,076;2

-950;1,028;0,02;2

-885;1,083;-0,008;2

-805;1,188;-0,069;2

-726;1,23;0,078;2

-649;1,216;-0,034;2

-578;1,324;-0,019;2

-522;0,965;0,113;2

-493;1,183;-0,054;2

-493;0,666;0,069;2

-521;0,515;0,012;2

-574;0,92;-0,028;2

-639;0,823;0,076;2

-699;0,636;-0,054;2

-748;0,53;0,047;2

-789;0,2;0,013;2

-818;-0,427;-0,054;2

-848;-1,093;0,118;2

-875;-1,1;-0,044;2

-904;-1,175;0,003;2

-931;-1,326;0,069;2

-963;-0,962;-0,061;2

-993;-0,589;0,093;2

-1022;-0,54;-0,038;2

-1042;-0,112;-0,023;2

-1061;-0,11;0,07;2

-1066;0,242;-0,126;2

-1073;0,306;0,067;2

-1077;0,039;-0,015;2

-1085;0,296;-0,033;2

-1093;0,148;0,073;2

-1106;0,327;-0,067;2

-1120;0,337;0,057;2

-1140;0,143;-0,013;2

-1157;0,421;-0,016;2

-1172;0,216;0,092;2

-1175;0,182;-0,043;2

-1173;0,502;0,098;2

-1170;0,366;0,03;2

-1161;0,53;-0,054;2

-1146;0,072;0,078;2

-1117;-0,097;-0,034;2

-1078;0,009;0,013;2

-1036;-0,213;0,031;2

-995;-0,178;0,009;2

-960;-0,732;0,067;2

-926;-0,965;-0,031;2

-885;-0,919;0,014;2

-831;-1,075;0,046;2

-767;-1,007;-0,079;2

-695;-0,743;0,06;2

-620;-0,846;0,057;2

-547;-0,479;-0;2

-496;-0,198;0,069;2

-476;0,289;-0,064;2

-502;0,351;0,031;2

-561;0,449;-0,022;2

-638;0,36;-0,064;2

-715;-0,048;0,058;2

-789;-0,125;-0,064;2

-845;-0,246;0,008;2

-891;-0,433;0,026;2

-918;-0,334;-0,094;2

-935;-0,255;0,166;2

-941;-0,057;-0,056;2

-947;0,289;-0,008;2

-955;0,375;0,076;2

-975;0,45;-0,034;2

-1000;0,386;0,087;2

-1033;0,463;-0,05;2

-1065;0,734;0,087;2

-1097;0,555;0,082;2

-1120;0,261;-0,077;2

-1139;0,232;0,058;2

-1144;-0,382;-0,034;2

-1142;-0,283;-0,022;2

-1137;-0,328;0,094;2

-1134;-0,449;-0,075;2

-1139;-0,271;0,053;2

-1149;-0,334;0,033;2

-1165;-0,134;-0,047;2

-1177;-0,13;0,084;2

-1186;0,456;-0,076;2

-1181;0,426;0,05;2

-1158;0,048;0,017;2

-1124;0,047;-0,063;2

-1074;0,079;0,13;2

-1020;-0,066;-0,023;2

-956;-0,232;0,002;2

-891;-0,2;0,054;2

-823;-0,315;-0,092;2

-761;-0,415;0,07;2

-702;-0,349;-0,005;2

-653;-0,227;-0,011;2

-610;-0,567;0,078;2

-574;-0,456;-0,083;2

-555;-0,558;0,083;2

-564;-0,769;0,02;2

-590;-0,494;-0,009;2

-635;-0,405;0,072;2

-684;-0,139;-0,078;2

-734;0,051;-0,002;2

-782;-0,063;0,01;2

-831;-0,169;-0,069;2

-881;-0,641;0,091;2

-932;-0,915;-0,05;2

-975;-0,907;0,008;2

-1011;-0,746;0,032;2

-1036;-0,349;-0,096;2

-1054;-0,18;0,079;2

-1065;-0,022;-0,041;2

-1072;0,463;-0,019;2

-1073;-0,022;0,049;2

-1072;-0,198;-0,08;2

-1071;-0,308;0,082;2

-1079;-0,423;-0,012;2

-1094;-0,286;-0,018;2

-1114;-0,981;0,078;2

-1141;-0,661;-0,083;2

-1160;-0,36;0,179;2

-1182;-0,934;-0,004;2

-1196;-0,542;-0,047;2

-1204;-0,642;0,086;2

-1201;-0,969;-0,056;2

-1180;-0,799;0,054;2

-1149;-0,129;0,005;2

-1107;-0,005;-0,071;2

-1069;-0,133;0,095;2

-1030;0,186;0,002;2

-994;0,465;0,004;2

-952;-0,08;0,02;2

-907;0,167;-0,073;2

-856;0,409;0,055;2

-805;0,482;-0,015;2

-745;0,637;0,004;2

-684;0,408;0,063;2

-618;0,287;-0,13;2

-563;-0,018;0,076;2

-530;-0,2;0,002;2

-530;0,471;-0,027;2

-562;0,615;0,057;2

-622;0,956;-0,134;2

-691;1,126;0,055;2

-765;0,914;-0,013;2

-830;0,735;-0,053;2

-884;0,488;0,09;2

-924;0,657;-0,075;2

-950;0,566;0,028;2

-962;0,249;0,101;2

-972;0,349;-0,05;2

-986;0,13;0,061;2

-1002;-0,366;0;2

-1024;-0,06;0,012;2

-1050;0,278;0,072;2

-1078;0,43;-0,096;2

-1103;0,431;0,1;2

-1123;0,558;0,004;2

-1132;0,98;-0,019;2

-1137;0,849;0,07;2

-1139;0,756;-0,081;2

-1144;0,713;0,053;2

-1146;0,56;-0,006;2

-1153;0,259;0,043;2

-1155;-0,457;0,072;2

-1162;-0,419;-0,011;2

-1175;-0,263;0,056;2

-1182;-0,049;0,015;2

-1194;0,634;-0,02;2

-1195;0,962;0,105;2

-1190;1,372;-0,072;2

-1169;1,491;0,01;2

-1135;1,197;0,023;2

-1087;1,125;-0,074;2

-1036;0,917;0,077;2

-977;0,862;-0,071;2

-922;0,725;0,005;2

-871;0,516;0,067;2

-825;0,063;-0,077;2

-780;-0,095;0,076;2

-731;-0,26;0,035;2

-680;-0,242;-0,014;2

-628;0,096;0,042;2

-585;0,209;-0,08;2

-561;0,148;0,057;2

-570;0,068;0,005;2

-609;-0,011;0;2

-665;0,223;0,074;2

-724;0,657;-0,08;2

-780;0,764;0,037;2

-825;0,695;-0,008;2

-864;0,92;-0,059;2

-898;0,605;0,078;2

-930;0,272;-0,017;2

-959;0,086;0,038;2

-987;-0,457;0,056;2

-1005;-1,639;-0,07;2

-1023;-1,805;0,075;2

-1037;-1,855;-0,048;2

-1054;-1,227;0,023;2

-1069;-0,409;0,061;2

-1086;-0,194;-0,063;2

-1100;0,161;0,061;2

-1112;0,709;-0,041;2

-1128;0,966;-0,02;2

-1138;0,956;0,077;2

-1145;0,732;-0,089;2

-1146;0,553;0,03;2

-1139;0,515;0,047;2

-1138;0,631;-0,014;2

-1143;0,004;0,084;2

-1152;-0,189;0,054;2

-1163;-0,313;0,023;2

-1176;-0,639;0,041;2

-1174;-1,031;-0,094;2

-1161;-1,63;0,062;2

-1134;-1,356;-0,08;2

-1097;-0,636;0,029;2

-1049;-0,602;0,036;2

-990;-0,104;-0,082;2

-926;0,095;0,044;2

-861;-0,134;-0,04;2

-796;-0,165;-0,032;2

-735;-0,277;0,122;2

-675;-0,214;-0,1;2

-616;-0,187;0,072;2

-567;-0,529;0,062;2

-525;-0,272;0,006;2

-510;0,144;0,104;2

-526;0,507;-0,094;2

-574;0,388;0,03;2

-636;0,03;-0,032;2

-704;0,277;0,024;2

-765;0,002;0,102;2

-812;-0,228;-0,084;2

-848;0,224;0,015;2

-885;0,061;0,015;2

-916;-0,091;-0,078;2

-946;-0,167;0,07;2

-973;-0,469;-0,05;2

-996;-0,161;-0,016;2

-1014;-0,598;-0,028;2

-1034;-0,404;-0,071;2

-1051;-0,2;0,081;2

-1060;-0,405;-0,042;2

-1071;-0,277;-0,015;2

-1078;0,004;0,074;2

-1083;0,027;-0,089;2

-1094;-0,355;0,061;2

-1102;-0,372;-0,002;2

-1110;-0;-0,034;2

-1117;-0,191;0,092;2

-1127;-0,201;-0,073;2

-1138;0,117;0,054;2

-1151;-0,125;0,019;2

-1160;0,037;-0,058;2

-1167;-0,221;0,069;2

-1168;-0,219;-0,043;2

-1161;0,182;0,017;2

-1141;-0,141;0,04;2

-1109;0,035;-0,082;2

-1069;-0,039;0,079;2

-1022;0,041;-0,062;2

-973;0,006;-0,004;2

-919;-0,36;0,031;2

-863;-0,609;-0,07;2

-803;-0,857;0,06;2

-738;-0,71;0,005;2

-670;-0,418;-0,012;2

-608;-0,515;0,063;2

-555;-0,475;0,011;2

-526;-0,502;0,07;2

-521;-0,765;-0,007;2

-547;-0,604;-0,052;2

-592;-0,886;0,086;2

-657;-0,727;-0,08;2

-716;-0,477;0,035;2

-773;-0,357;0,018;2

-818;-0,027;-0,072;2

-858;0,261;0,062;2

-890;0,645;-0,035;2

-924;1,144;-0,004;2

-952;0,942;0,06;2

-977;0,926;-0,069;2

-1002;0,399;0,06;2

-1025;-0,099;-0,088;2

-1050;-0,236;0,005;2

-1075;-0,005;-0,047;2

-1098;-0,351;-0,081;2

-1114;-0,209;0,061;2

-1124;0,061;-0,021;2

-1126;0,392;0,004;2

-1124;0,35;0,122;2

-1123;0,231;-0,047;2

-1125;-0,088;0,053;2

-1138;-0,24;0,016;2

-1151;-0,107;-0,042;2

-1168;-0,575;0,083;2

-1185;-0,584;-0,066;2

-1199;-0,477;0,063;2

-1198;0,034;0,005;2

-1177;0,469;-0,094;2

-1134;-0,015;0,089;2

-1086;0,015;-0,087;2

-1029;-0,156;0,005;2

-975;-0,271;0,047;2

-912;0,091;-0,066;2

-851;0,308;0,081;2

-787;0,421;-0,055;2

-725;0,84;-0,025;2

-663;0,439;0,1;2

-608;0,532;-0,085;2

-558;0,383;0,045;2

-528;0,347;0,017;2

-523;0,722;-0,052;2

-555;0,49;0,095;2

-612;0,372;-0,044;2

-673;0,471;0,028;2

-733;0,471;0,001;2

-786;0,652;-0,073;2

-829;0,399;0,099;2

-866;0,302;-0,05;2

-899;0,607;0,018;2

-928;0,841;0,025;2

-958;0,832;-0,083;2

-984;0,447;0,128;2

-1005;-0,04;-0,009;2

-1020;-0,004;0,018;2

-1033;-0,101;0,039;2

-1049;0,102;-0,112;2

-1062;0,623;0,08;2

-1080;0,762;-0,008;2

-1093;0,85;-0,034;2

-1106;0,761;0,089;2

-1114;0,65;-0,06;2

-1124;0,57;-0,037;2

-1136;-0,007;-0,015;2

-1150;-0,288;-0,043;2

-1162;-0,407;0,061;2

-1173;-0,482;-0,085;2

-1182;-0,668;0,009;2

-1189;-0,961;0,033;2

-1192;-0,812;-0,034;2

-1191;-0,386;0,155;2

-1190;0,145;-0,062;2

-1180;0,511;0,005;2

-1166;0,524;0,076;2

-1136;0,327;-0,074;2

-1100;0,091;0,097;2

-1043;0,464;-0,026;2

-973;0,484;0,045;2

-893;0,176;0,037;2

-819;0,343;-0,057;2

-754;0,556;0,087;2

-704;0,858;-0,012;2

-657;0,937;0,007;2

-614;0,525;0,076;2

-575;0,258;-0,101;2

-543;0,42;0,029;2

-526;0,346;-0,002;2

-537;0,034;-0,041;2

-572;0,092;0,057;2

-626;-0,093;-0,065;2

-691;-0,229;0,006;2

-753;0,033;0,082;2

-813;0,177;-0,087;2

-860;0,338;0,08;2

-896;0,592;-0,04;2

-922;0,999;-0,002;2

-942;0,7;0,051;2

-964;0,717;-0,102;2

-986;0,296;0,075;2

-1013;-0,141;-0,028;2

-1042;-0,043;-0,027;2

-1074;0,034;0,059;2

-1098;0,278;-0,085;2

-1110;0,335;0,058;2

-1110;0,145;-0,014;2

-1106;0,226;-0,032;2

-1103;0,212;0,075;2

-1108;0,292;-0,055;2

-1122;0,295;0,049;2

-1141;0,181;-0,008;2

-1162;0,288;-0,055;2

-1182;0,192;0,109;2

-1200;0,514;-0,072;2

-1207;0,612;0,017;2

-1205;0,41;0,151;2

-1199;0,013;-0,091;2

-1192;-0,057;0,11;2

-1180;0,13;-0,053;2

-1159;0,093;0,008;2

-1128;-0,082;0,04;2

-1093;0,097;-0,095;2

-1053;-0,214;0,06;2

-1015;-0,515;-0,011;2

-970;-0,211;-0,091;2

-920;-0,125;0,087;2

-859;-0,3;-0,064;2

-799;-0,822;0,061;2

-731;-1,028;0,036;2

-672;-0,736;-0,047;2

-614;-0,487;0,104;2

-566;-0,473;-0,087;2

-533;-0,283;0,002;2

-530;-0,555;0,023;2

-555;-0,845;-0,054;2

-599;-0,738;0,109;2

-653;-0,06;-0,087;2

-705;0,291;0,013;2

-757;0,195;0,045;2

-810;0,578;-0,074;2

-862;0,21;0,06;2

-912;-0,137;-0,051;2

-954;0,278;0,017;2

-981;-0,128;0,047;2

-1000;-0,637;-0,069;2

-1020;-0,825;0,095;2

-1032;-1,133;-0,006;2

-1042;-0,733;0,032;2

-1050;-0,76;0,028;2

-1059;-0,52;-0,066;2

-1073;-0,18;0,051;2

-1090;-0,144;0,013;2

-1104;0,203;-0,046;2

-1114;0,567;0,122;2

-1121;0,559;-0,091;2

-1133;0,422;0,044;2

-1144;0,327;0,031;2

-1162;0,479;-0,053;2

-1175;0,528;0,076;2

-1186;0,476;-0,068;2

-1188;0,257;-0,02;2

-1189;-0,449;0,072;2

-1180;-0,696;-0,117;2

-1171;-0,887;0,092;2

-1150;-0,925;-0,046;2

-1125;-1,09;-0,007;2

-1091;-1,139;0,079;2

-1056;-0,684;-0,104;2

-1013;-0,28;0,03;2

-969;0,017;-0,006;2

-918;0,211;-0,03;2

-865;0,095;0,083;2

-799;-0,148;-0,025;2

-733;-0,322;0,044;2

-669;-0,713;-0,011;2

-601;-0,772;-0,041;2

-544;-0,431;0,13;2

-493;-0,122;-0,067;2

-470;0,001;0,01;2

-483;-0,011;0,015;2

-534;0,074;-0,064;2

-604;-0,064;0,057;2

-679;0,04;-0,059;2

-752;0,17;0,008;2

-809;-0,122;0,037;2

-860;-0,162;-0,096;2

-897;-0,143;0,074;2

-927;-0,283;-0,05;2

-947;-0,538;-0,031;2

-966;-0,665;0,044;2

-979;-0,351;-0,089;2

-994;-0,425;0,124;2

-1009;-0,852;0,012;2

-1024;-0,021;-0,04;2

-1042;0,274;0,082;2

-1061;0,068;-0,031;2

-1085;0,06;-0,013;2

-1106;-0,15;-0,001;2

-1126;0,15;-0,068;2

-1136;0,271;0,072;2

-1146;-0,074;-0,098;2

-1152;-0,363;0,02;2

-1159;-0,611;0,016;2

-1164;-0,225;-0,129;2

-1174;-0,138;0,049;2

-1181;-0,129;-0,031;2

-1189;0,633;-0,003;2

-1185;0,986;0,024;2

-1168;1,381;-0,098;2

-1135;1,059;0,069;2

-1096;0,697;-0,008;2

-1048;0,763;-0,011;2

-1006;0,406;0,083;2

-960;0,41;-0,07;2

-909;0,373;0,043;2

-853;-0,414;-0,038;2

-788;-0,62;-0,026;2

-721;-0,678;0,168;2

-647;-1,144;-0,061;2

-571;-1,241;0,01;2

-503;-0,767;0,009;2

-448;-0,43;-0,063;2

-432;-0,485;0,078;2

-455;-0,519;-0,034;2

-516;-0,464;0,064;2

-591;-0,316;0,028;2

-671;-0,138;-0,081;2

-746;-0,008;0,079;2

-805;0,288;-0,041;2

-852;0,49;-0,049;2

-892;0,65;0,056;2

-920;0,941;-0,091;2

-942;0,655;0,002;2

-963;0,759;0,012;2

-979;0,737;-0,034;2

-996;0,726;0,084;2

-1010;1,389;-0,051;2

-1029;1,373;0,016;2

-1049;1,201;-0,037;2

-1074;1,024;-0,066;2

-1096;0,121;0,001;2

-1116;-0,12;-0,076;2

-1131;0,058;-0,008;2

-1144;-0,006;0,03;2

-1152;-0,072;-0,071;2

-1162;-0,252;0,063;2

-1163;-0,22;-0,063;2

-1165;-0,244;-0,002;2

-1164;-0,573;0,059;2

-1165;-0,309;-0,072;2

-1172;0,082;0,073;2

-1180;0,204;-0,013;2

-1181;0,327;-0,006;2

-1174;0,315;0,064;2

-1154;0,428;-0,103;2

-1121;0,336;0,112;2

-1085;-0,002;0,004;2

-1044;0,292;-0,043;2

-999;0,247;0,083;2

-943;0,212;-0,033;2

-880;0,712;0,02;2

-816;0,87;0,029;2

-745;1,153;-0,069;2

-676;1,655;0,123;2

-610;1,707;-0,047;2

-555;1,454;0,059;2

-520;0,975;0,013;2

-519;0,712;-0,078;2

-555;0,291;0,078;2

-616;-0,026;-0,044;2

-684;0,182;0,004;2

-751;0,032;0,055;2

-810;-0,192;-0,073;2

-859;-0,071;0,068;2

-902;-0,204;0,01;2

-928;0,252;-0,031;2

-954;0,242;0,253;2

-973;0,302;-0,056;2

-993;0,457;0,075;2

-1006;0,32;0,03;2

-1025;0,405;-0,047;2

-1041;0,278;0,097;2

-1065;0,064;-0,067;2

-1086;-0,42;0,023;2

-1108;-1,176;0,046;2

-1125;-1,448;-0,064;2

-1142;-1,219;0,092;2

-1156;-0,823;-0,016;2

-1167;0,038;0,024;2

-1177;0,643;0,031;2

-1180;1,051;-0,084;2

-1186;1,091;0,065;2

-1190;1,154;0,197;2

-1194;1,022;0,011;2

-1197;-0,015;0,124;2

-1191;-0,339;-0,083;2

-1167;-0,158;0,079;2

-1135;0,085;-0,004;2

-1093;0,709;0,016;2

-1044;0,671;0,07;2

-991;0,612;-0,054;2

-927;0,192;0,042;2

-858;-0,632;0,033;2

-786;-0,609;-0,038;2

-719;-0,794;0,116;2

-659;-0,738;-0,057;2

-604;-0,33;0,003;2

-558;0,002;0,05;2

-521;0,223;-0,072;2

-513;0,376;0,059;2

-532;0,408;0,004;2

-585;0,331;-0,008;2

-652;-0,11;0,049;2

-724;-0,146;-0,072;2

-784;0,052;0,098;2

-842;0,287;-0,034;2

-890;0,441;-0,03;2

-929;0,367;0,047;2

-954;0,301;-0,081;2

-971;0,01;0,062;2

-985;-0,452;0,024;2

-1005;-0,41;0,044;2

-1028;-0,323;0,041;2

-1050;0,068;-0,05;2

-1070;0,699;0,024;2

-1088;0,684;0,003;2

-1105;0,753;-0,079;2

-1123;0,372;0,098;2

-1141;-0,044;-0,068;2

-1151;0,13;0,013;2

-1156;0,05;0,082;2

-1157;0,099;-0,084;2

-1157;0,004;0,068;2

-1165;-0,013;-0,04;2

-1177;-0,083;-0,02;2

-1187;-0,454;0,025;2

-1196;-0,783;0,056;2

-1204;-0,873;0,085;2

-1213;-0,3;-0,013;2

-1214;0,304;-0,015;2

-1212;0,005;0,092;2

-1198;-0,266;-0,061;2

-1170;-0,173;0,076;2

-1136;-0,273;0,002;2

-1092;-0,821;-0,022;2

-1047;-1,016;0,088;2

-1000;-0,816;-0,048;2

-950;-0,914;0,043;2

-895;-0,911;0,018;2

-836;-0,597;-0,072;2

-775;-0,822;0,078;2

-710;-1,227;-0,073;2

-644;-0,675;0,063;2

-581;-0,481;-0,014;2

-534;-0,219;-0,084;2

-506;0,421;0,078;2

-510;0,547;-0,031;2

-542;0,771;-0,019;2

-600;0,335;0,065;2

-665;-0,322;-0,074;2

-732;-0,729;0,088;2

-786;-0,89;-0,025;2

-836;-0,689;-0,033;2

-876;-0,37;0,094;2

-918;0,082;-0,089;2

-954;0,164;0,063;2

-987;0,271;0,007;2

-1006;0,651;-0,053;2

-1016;0,562;0,082;2

-1023;0,456;-0,063;2

-1034;0,486;0,022;2

-1049;0,328;0,01;2

-1071;0,434;-0,088;2

-1094;0,674;0,09;2

-1113;1,058;-0,023;2

-1129;1,305;-0,009;2

-1140;0,949;0,121;2

-1151;0,961;-0,078;2

-1154;1,017;0,064;2

-1160;1,051;-0,049;2

-1163;1,015;-0,011;2

-1170;0,661;0,075;2

-1179;0,772;-0,129;2

-1183;0,62;0,093;2

-1182;0,087;0,02;2

-1174;-0,14;-0,051;2

-1167;-0,39;0,073;2

-1160;-0,1;-0,056;2

-1155;0,032;0,059;2

-1151;-0,259;0,045;2

-1139;-0,233;-0,109;2

-1118;-0,114;0,136;2

-1092;-0,319;-0,051;2

-1058;0,099;0,019;2

-1011;0,375;0,126;2

-960;0,064;-0,042;2

-894;0,016;0,072;2

-823;-0,186;-0,038;2

-746;-0,849;-0,01;2

-676;-1,295;0,061;2

-609;-0,978;-0,092;2

-556;-0,565;0,074;2

-514;0,051;-0,031;2

-508;1,401;-0,01;2

-528;1,683;0,054;2

-577;0,571;-0,135;2

-639;-0,235;0,056;2

-708;-0,503;-0;2

-764;-0,415;-0,043;2

-815;-0,514;0,088;2

-855;0,024;-0,073;2

-894;1,34;0,116;2

-926;0,609;0,009;2

-957;-0,247;-0,047;2

-982;-0,783;0,092;2

-1006;-1,291;-0,058;2

-1027;-1,348;0,014;2

-1044;-1,389;0,053;2

-1057;-1,228;-0,096;2

-1068;-1,117;0,089;2

-1088;-1,057;-0,043;2

-1105;-1,366;-0,006;2

-1126;-2,026;0,069;2

-1143;-2,233;-0,094;2

-1159;-2,492;0,129;2

-1171;-2,641;-0,037;2

-1175;-2,149;-0,031;2

-1179;-2,04;0,071;2

-1177;-1,695;-0,039;2

-1176;-1,271;0,069;2

-1174;-1,206;0,01;2

-1178;-0,938;0,02;2

-1182;-0,708;0,09;2

-1187;-0,505;-0,072;2

-1192;-0,539;-0,013;2

-1197;-0,739;-0,02;2

-1199;-0,278;-0,041;2

-1190;-0,084;0,077;2

-1167;0,236;-0,038;2

-1136;0,624;-0,052;2

-1101;0,533;0,038;2

-1056;0,649;-0,034;2

-1004;0,731;0,083;2

-940;0,655;-0,02;2

-878;1,197;0,005;2

-813;1,409;0,079;2

-748;1,08;-0,115;2

-681;0,802;0,083;2

-622;0,585;0,006;2

-572;0,168;0,033;2

-546;-0,229;0,075;2

-543;0,044;-0,069;2

-571;0,304;0,055;2

-616;0,128;0,012;2

-671;0,076;-0,044;2

-728;-0,123;0,089;2

-776;0,26;-0,081;2

-819;-0,062;0,015;2

-855;-0,385;0,022;2

-891;-0,195;-0,068;2

-922;-0,366;0,087;2

-950;-0,497;-0,081;2

-970;-0,099;0,026;2

-990;-0,266;0,057;2

-1007;-0,392;-0,058;2

-1026;-0,349;0,073;2

-1045;-0,491;-0,035;2

-1060;-0,119;0,02;2

-1074;0,021;0,052;2

-1089;-0,495;-0,084;2

-1105;-0,502;0,073;2

-1121;-0,225;0,043;2

-1128;0,336;0,008;2

-1135;0,688;0,101;2

-1137;0,701;-0,058;2

-1147;0,677;0,033;2

-1153;0,098;0,022;2

-1162;0,297;-0,069;2

-1168;-0,108;0,094;2

-1170;-0,338;-0,049;2

-1168;0,159;-0,036;2

-1169;0,285;0,017;2

-1167;0,615;-0,06;2

-1157;0,296;0,162;2

-1139;-0,193;-0,027;2

-1109;-0,405;0,012;2

-1075;-0,798;0,054;2

-1031;-0,958;-0,08;2

-981;-1,015;0,089;2

-917;-1,167;-0,072;2

-841;-0,677;-0,04;2

-757;-0,653;0,006;2

-678;-0,778;-0,047;2

-603;-0,436;0,059;2

-539;-0,713;0,037;2

-483;-1,103;-0,064;2

-453;-1,03;0,074;2

-459;-0,359;-0,058;2

-508;0,325;0,013;2

-575;0,198;0,013;2

-647;-0,183;-0,05;2

-712;-0,269;0,074;2

-762;0,007;-0,095;2

-804;0,366;0,003;2

-842;-0,072;0,042;2

-874;-0,15;-0,091;2

-896;0,197;0,074;2

-919;0,089;-0,042;2

-946;0,172;-0,014;2

-972;-0,388;0,083;2

-1002;-0,464;-0,122;2

-1026;-0,188;0,054;2

-1054;-0,23;-0,028;2

-1073;0,012;-0,023;2

-1092;0,235;0,092;2

-1099;0,228;-0,086;2

-1105;0,449;0,026;2

-1103;1,118;0,028;2

-1104;1,149;0,031;2

-1110;0,718;0,073;2

-1126;0,619;-0,059;2

-1134;0,36;0,043;2

-1146;-0,049;0,022;2

-1157;-0,251;-0,056;2

-1166;-0,758;0,063;2

-1174;-0,934;0,11;2

-1177;-1,054;0,037;2

-1169;-0,729;0,061;2

-1151;-0,124;-0,088;2

-1122;0,041;0,068;2

-1086;-0,028;0,009;2

-1039;-0,231;-0,015;2

-987;-0,501;0,077;2

-924;-0,495;-0,084;2

-855;-0,854;0,07;2

-784;-0,867;0,006;2

-712;-0,656;-0,03;2

-640;-0,858;0,102;2

-568;-0,73;-0,07;2

-503;-0,611;0,147;2

-459;-0,702;0,02;2

-446;-0,368;-0,068;2

-478;-0,265;0,14;2

-541;0,096;-0,073;2

-612;0,172;0,012;2

-684;-0,135;0,034;2

-741;0,042;-0,06;2

-784;0,05;0,094;2

-821;-0,182;-0,067;2

-847;-0,255;-0,004;2

-875;-0,411;0,025;2

-903;-0,457;-0,092;2

-932;-0,429;0,029;2

-960;-0,165;0,019;2

-990;0,06;0,002;2

-1012;0,075;0,112;2

-1031;0,07;-0,094;2

-1046;0,056;0,062;2

-1064;-0,354;0,03;2

-1080;-0,134;-0,025;2

-1094;0,259;0,098;2

-1103;0,436;-0,047;2

-1108;0,381;0,031;2

-1112;0,363;0,027;2

-1116;0,596;-0,042;2

-1120;0,45;0,106;2

-1128;0,422;-0,056;2

-1140;0,9;0,004;2

-1152;1,235;0,02;2

-1167;1,285;-0,086;2

-1170;0,773;0,07;2

-1164;0,507;-0,034;2

-1134;1,094;-0,012;2

-1096;1,519;0,092;2

-1051;1,483;-0,084;2

-1007;0,559;0,088;2

-958;0,282;0,007;2

-904;0,548;-0,01;2

-844;0,06;0,088;2

-777;-0,332;-0,101;2

-708;-0,288;0,126;2

-641;-0,601;-0,002;2

-575;-0,429;-0,04;2

-518;-0,426;0,091;2

-475;-0,206;-0,07;2

-461;-0,32;0,04;2

-485;-0,421;-0,014;2

-539;-0,267;-0,072;2

-610;-0,529;0,088;2

-677;-0,565;-0,043;2

-739;-0,45;-0,012;2

-791;-0,379;0,09;2

-838;0,031;-0,101;2

-875;0,152;0,221;2

-908;-0,014;-0,018;2

-940;0,236;-0,019;2

-966;0,048;0,056;2

-988;-0,849;-0,071;2

-1007;-1,491;0,058;2

-1025;-1,548;-0,024;2

-1040;-0,801;-0,034;2

-1058;-0,521;0,076;2

-1072;-0,019;-0,06;2

-1091;0,405;0,007;2

-1102;0,271;0,038;2

-1114;0,231;-0,076;2

-1120;0,448;0,057;2

-1134;0,462;-0,09;2

-1146;0,275;0,016;2

-1157;0,301;0,036;2

-1162;0,638;-0,059;2

-1169;1,122;0,063;2

-1169;1,468;-0,048;2

-1173;1,14;-0,005;2

-1174;0,953;0,058;2

-1173;1,171;-0,013;2

-1171;0,919;0,106;2

-1170;0,629;-0,022;2

-1160;0,773;0,053;2

-1143;0,466;0,049;2

-1117;0,07;-0,09;2

-1081;-0,104;0,04;2

-1034;-0,547;0,021;2

-980;-0,432;-0,039;2

-922;-0,702;0,117;2

-855;-0,596;-0,069;2

-791;-0,524;0,027;2

-724;-1,16;0,002;2

-660;-1,383;-0,059;2

-598;-1,322;0,089;2

-541;-0,997;-0,053;2

-500;-0,494;0,017;2

-476;0,006;0,029;2

-490;-0,11;-0,129;2

-537;-0,612;0,077;2

-607;-0,074;-0,091;2

-678;0,17;0,001;2

-742;0,023;0,06;2

-794;0,76;-0,104;2

-833;1,411;0,081;2

-867;1,594;0,004;2

-901;1,311;0,032;2

-931;1,194;0,059;2

-957;1,554;-0,079;2

-979;1,494;0,061;2

-996;0,915;0,003;2

-1017;0,52;0,092;2

-1037;-0,164;0,1;2

-1058;-0,468;-0,084;2

-1076;-0,578;0,061;2

-1091;-0,817;0;2

-1100;-0,445;-0,037;2

-1112;-0,077;0,086;2

-1121;0,175;-0,048;2

-1130;0,171;-0,003;2

-1134;0,161;0,044;2

-1141;0,953;-0,086;2

-1146;1,357;0,05;2

-1155;0,945;-0,045;2

-1161;0,345;-0,038;2

-1170;-0,46;0,069;2

-1176;-0,677;-0,063;2

-1180;-0,254;0,076;2

-1177;-0,466;-0,021;2

-1162;-0,024;-0,04;2

-1138;-0,096;0,06;2

-1100;0,372;-0,054;2

-1062;0,532;0,021;2

-1017;0,009;-0,006;2

-974;-0,181;-0,07;2

-929;-0,3;0,092;2

-877;-0,145;-0,085;2

-824;0,142;0,032;2

-762;-0,009;-0,004;2

-696;0,15;-0,079;2

-629;-0,069;0,068;2

-567;-0,071;-0,042;2

-517;0,255;0,025;2

-491;0,38;0,023;2

-493;0,592;-0,065;2

-527;0,715;0,097;2

-585;0,457;-0,018;2

-647;0,788;0,009;2

-708;0,396;0,084;2

-762;0,138;-0,1;2

-814;0,109;0,053;2

-856;0,012;-0,006;2

-898;0,285;-0,07;2

-929;0,398;0,099;2

-955;0,556;-0,034;2

-976;0,461;0,046;2

-995;0,284;-0,028;2

-1007;0,572;-0,06;2

-1022;0,178;0,096;2

-1032;0,411;-0,061;2

-1052;1,034;0,025;2

-1074;0,472;-0,004;2

-1096;0,047;-0,083;2

-1113;-0,387;0,073;2

-1126;-0,427;0,003;2

-1139;-0,171;-0,027;2

-1147;-0,328;0,053;2

-1153;0,082;-0,085;2

-1156;0,714;0,055;2

-1161;0,686;-0,003;2

-1163;0,641;-0,012;2

-1172;0,223;0,104;2

-1175;-0,264;-0,07;2

-1178;0,039;0,055;2

-1174;0,104;0,039;2

-1156;0,073;-0,02;2

-1132;0,185;0,103;2

-1093;0,233;-0,096;2

-1056;-0,152;0,033;2

-1006;-0,258;0,002;2

-952;0,058;-0,068;2

-892;0,152;0,094;2

-831;0,031;-0,041;2

-771;0,03;-0,067;2

-707;0,133;0,027;2

-642;0,293;-0,083;2

-582;0,196;0,059;2

-538;0,271;-0,007;2

-512;0,536;-0,023;2

-522;0,484;0,06;2

-555;0,745;-0,122;2

-610;0,76;0,044;2

-671;0,795;0,012;2

-737;0,88;-0,049;2

-799;0,622;0,105;2

-857;0,229;-0,061;2

-903;-0,058;0,047;2

-943;-0,211;-0,005;2

-969;-0,461;-0,045;2

-991;-0,626;0,053;2

-1002;-0,585;-0,087;2

-1012;-0,532;0,015;2

-1022;-0,087;0,015;2

-1034;0,508;-0,096;2

-1052;0,665;0,084;2

-1071;0,256;-0,064;2

-1091;-0,032;-0,004;2

-1112;-0,451;0,051;2

-1134;-0,234;-0,048;2

-1154;-0,026;0,142;2

-1171;-0,335;-0,022;2

-1174;-0,224;0,003;2

-1179;-0,259;0,109;2

-1175;0,048;-0,102;2

-1177;0,812;0,089;2

-1177;1,726;0,029;2

-1180;2,691;-0,073;2

-1184;2,828;0,076;2

-1187;1,996;-0,071;2

-1193;0,9;0,022;2

-1199;0,001;-0,01;2

-1203;-0,162;-0,038;2

-1193;-0,479;0,081;2

-1174;-0,237;0,086;2

-1142;-0,007;0,027;2

-1106;0,381;0,029;2

-1067;0,609;-0,085;2

-1023;-0,153;0,113;2

-968;-0,88;-0,04;2

-905;-0,952;0,013;2

-838;-1,068;0,019;2

-769;-1,346;-0,097;2

-703;-2,114;0,083;2

-635;-2,055;-0,011;2

-571;-1,772;-0,015;2

-523;-1,411;0,07;2

-501;-0,879;-0,078;2

-503;-0,713;0,045;2

-543;-0,271;-0,021;2

-595;0,42;-0,025;2

-661;0,29;0,087;2

-722;0,126;-0,092;2

-780;-0,119;0,039;2

-827;-0,213;0,01;2

-868;-0,026;-0,022;2

-894;-0,024;0,092;2

-916;-0,271;-0,09;2

-931;-0,12;0,01;2

-950;0,027;0,016;2

-967;0,086;-0,115;2

-989;0,18;0,047;2

-1008;0,031;-0,045;2

-1034;0,272;0,021;2

-1058;0,455;0,05;2

-1080;0,33;-0,077;2

-1093;-0,316;0,081;2

-1100;-0,549;-0,008;2

-1100;-0,1;-0,035;2

-1106;-0,55;0,085;2

-1112;-0,638;-0,011;2

-1122;-0,004;0,021;2

-1126;0,277;0,016;2

-1133;0,23;-0,075;2

-1137;-0,058;0,064;2

-1151;0,124;-0,074;2

-1158;0,323;0,009;2

-1166;-0,181;0,011;2

-1172;-0,33;-0,036;2

-1178;0,032;0,063;2

-1185;0,477;-0,043;2

-1188;0,782;0,023;2

-1176;0,83;0,037;2

-1147;0,876;-0,047;2

-1110;0,394;0,092;2

-1071;0,261;-0,013;2

-1036;0,042;0,034;2

-999;-0,411;0,066;2

-961;-0,265;-0,042;2

-906;-0,114;0,069;2

-847;-0,156;0,025;2

-778;-0,211;-0,007;2

-708;-0,575;0,068;2

-634;-0,625;-0,046;2

-565;-0,481;0,044;2

-505;-0,391;0,003;2

-468;-0,043;-0,047;2

-462;-0,035;0,124;2

-493;0,095;-0,056;2

-548;0,087;-0,023;2

-613;0,071;0,029;2

-676;0,182;-0,045;2

-730;0,091;0,106;2

-775;-0,061;-0,029;2

-813;-0,038;-0,023;2

-849;-0,274;0,087;2

-880;-0,296;-0,077;2

-912;-0,177;-0,013;2

-943;0,038;-0,02;2

-967;0,41;-0,028;2

-991;0,798;0,072;2

-1009;1,063;-0,085;2

-1024;0,742;0,048;2

-1033;-0,294;0,005;2

-1045;-0,539;-0,075;2

-1054;-0,654;0,107;2

-1065;-0,301;-0,073;2

-1080;0,335;0,039;2

-1098;0,527;0,019;2

-1115;1,056;-0,109;2

-1133;0,923;0,089;2

-1144;0,771;-0,051;2

-1149;0,767;0,007;2

-1151;0,364;0,026;2

-1152;0,462;-0,064;2

-1152;0,185;0,065;2

-1149;-0,092;-0,02;2

-1144;-0,441;0,005;2

-1139;-0,729;0,062;2

-1139;-0,574;-0,085;2

-1144;-0,199;0,112;2

-1151;-0,314;0,015;2

-1141;-0,163;-0,016;2

-1120;-0,338;0,074;2

-1089;-0,942;-0,055;2

-1056;-1,019;0,061;2

-1021;-0,854;0,008;2

-984;-1,005;-0,037;2

-934;-1,166;0,071;2

-877;-0,958;-0,061;2

-812;-0,522;0,045;2

-750;0,119;0,047;2

-685;0,429;-0,064;2

-625;0,052;0,072;2

-566;-0,856;-0,056;2

-516;-1,063;-0,016;2

-482;-1,184;0,002;2

-478;-0,958;-0,114;2

-512;-0,842;0,064;2

-564;-0,947;-0,012;2

-633;-0,945;0,001;2

-695;-1,164;0,065;2

-755;-1,15;-0,045;2

-800;-0,913;0,078;2

-836;-0,835;-0,013;2

-868;-0,354;-0,001;2

-892;0,031;0,071;2

-920;0,076;-0,054;2

-949;-0,043;0,045;2

-977;-0,225;-0,007;2

-1002;-0,267;-0,04;2

-1024;-0,532;0,193;2

-1036;-0,624;-0,064;2

-1053;-0,242;0,035;2

-1062;-0,172;0,033;2

-1075;0,054;-0,07;2

-1081;0,314;0,112;2

-1092;0,185;-0,058;2

-1100;0,321;0,073;2

-1113;0,373;0,078;2

-1123;0,272;-0,096;2

-1132;0,194;0,09;2

-1137;0,067;-0,021;2

-1143;0,075;0,002;2

-1146;-0,034;0,098;2

-1149;0,376;-0,056;2

-1152;0,638;0,031;2

-1152;0,471;-0,04;2

-1153;0,592;-0,065;2

-1145;0,445;0,038;2

-1125;0,265;-0,078;2

-1095;0,162;-0,01;2

-1055;0,038;0,017;2

-1008;0,182;-0,044;2

-962;0,341;0,08;2

-911;0,587;-0,076;2

-858;0,548;0,015;2

-801;0,287;0,08;2

-745;0,372;-0,131;2

-686;-0,045;0,003;2

-631;-0,042;-0,056;2

-575;0,241;-0,011;2

-520;0,211;0,052;2

-479;0,133;-0,089;2

-462;-0,272;0,063;2

-482;-0,414;0;2

-535;-0,007;-0,007;2

-601;-0,125;0,063;2

-668;-0,372;-0,079;2

-729;-0,445;0,058;2

-780;-0,909;0;2

-825;-1,52;0,084;2

-866;-1,541;0,029;2

-899;-1,003;-0,073;2

-927;-0,594;0,078;2

-952;-0,475;0,031;2

-980;-0,112;-0,054;2

-1002;0,002;0,075;2

-1024;-0,096;-0,086;2

-1036;0,023;-0,011;2

-1051;-0,131;-0,001;2

-1062;-0,217;-0,073;2

-1076;-0,708;0,066;2

-1086;-0,558;-0,024;2

-1099;-0,201;-0,017;2

-1108;0,012;0,01;2

-1120;-0,063;-0,104;2

-1135;0,186;0,075;2

-1152;0,1;-0,014;2

-1165;0,374;-0,018;2

-1173;0,459;0,102;2

-1174;0,429;-0,053;2

-1180;0,474;0,036;2

-1183;0,527;0,013;2

-1186;0,579;-0,053;2

-1190;0,352;0,064;2

-1190;0,37;-0,036;2

-1193;0,392;0,024;2

-1195;0,052;0,024;2

-1200;0,087;0,046;2

-1196;-0,044;0,092;2

-1182;-0,236;-0,051;2

-1159;-0,153;0,014;2

-1126;-0,103;0,066;2

-1091;-0,033;-0,09;2

-1048;-0,002;0,065;2

-996;0,09;-0,03;2

-932;0,321;-0,012;2

-865;0,368;0,071;2

-798;0,399;-0,069;2

-735;0,277;0,051;2

-675;0,484;-0,017;2

-617;0,731;-0,033;2

-568;0,475;0,053;2

-530;0,252;-0,066;2

-525;-0,104;0,054;2

-550;0,021;0,002;2

-601;0,431;-0,063;2

-664;0,214;0,028;2

-734;-0,392;-0,075;2

-799;-0,837;-0,002;2

-856;-1,31;0,017;2

-896;-0,936;-0,077;2

-929;-0,089;0,085;2

-948;0,131;0,005;2

-965;0,571;0,008;2

-981;0,66;0,063;2

-1001;1,07;-0,078;2

-1023;1,207;0,073;2

-1051;0,821;-0,022;2

-1076;0,517;-0,016;2

-1102;0,632;0,074;2

-1121;0,718;-0,078;2

-1131;0,078;0,039;2

-1137;-0,408;0,007;2

-1138;0,111;-0,055;2

-1142;-0,147;0,067;2

-1149;-0,487;-0,068;2

-1158;-0,626;0,027;2

-1164;-0,74;0,01;2

-1170;-0,581;-0,054;2

-1177;-0,839;0,108;2

-1185;-1,066;-0,061;2

-1195;-0,773;-0,032;2

-1200;-0,652;0,009;2

-1205;-0,558;-0,103;2

-1207;-0,72;0,063;2

-1211;-0,209;-0,037;2

-1202;0,076;-0,02;2

-1186;0,502;0,063;2

-1151;0,695;-0,088;2

-1112;0,309;0,065;2

-1066;0,114;-0,016;2

-1017;-0,125;-0,013;2

-967;-0,442;0,063;2

-914;-0,036;-0,046;2

-851;0,522;0,056;2

-789;0,421;0,014;2

-722;0,511;-0,038;2

-657;0,106;0,055;2

-598;0,048;-0,072;2

-545;0,471;0,036;2

-512;0,223;0,013;2

-509;0,38;-0,062;2

-540;0,386;0,041;2

-596;0,835;-0,065;2

-663;1,094;0,01;2

-726;1,247;0,04;2

-778;1,314;-0,054;2

-825;0,798;0,092;2

-869;0,792;-0,014;2

-911;0,972;-0,006;2

-948;0,737;0,061;2

-973;0,973;-0,079;2

-996;0,791;0,067;2

-1014;0,146;-0,019;2

-1033;0,263;-0,039;2

-1050;0,224;0,069;2

-1070;0,635;-0,085;2

-1077;0,751;0,041;2

-1089;0,534;0,01;2

-1098;0,679;-0,048;2

-1110;0,746;0,069;2

-1128;0,636;-0,044;2

-1140;0,458;0,042;2

-1153;0,263;0,026;2

-1163;0,638;-0,103;2

-1175;0,938;0,063;2

-1183;0,833;0,119;2

-1186;0,415;0,032;2

-1183;-0,039;0,065;2

-1177;0,176;-0,074;2

-1173;0,057;0,08;2

-1173;-0,211;-0,014;2

-1168;-0,258;-0,003;2

-1157;-0,155;0,075;2

-1137;0,204;-0,072;2

-1105;0,387;0,068;2

-1076;0,495;0,003;2

-1041;0,522;-0,01;2

-1006;0,133;0,077;2

-957;-0,381;-0,085;2

-908;-0,683;-0,025;2

-845;-0,868;0,027;2

-786;-0,736;-0,061;2

-719;-0,674;0,082;2

-654;-0,811;-0,088;2

-587;-0,382;0,023;2

-532;-0,332;0,046;2

-489;-0,15;-0,078;2

-484;0,165;0,052;2

-512;0,344;-0,024;2

-571;0,504;0,002;2

-640;0,403;-0,031;2

-713;0,631;-0,12;2

-773;0,437;0,066;2

-829;0,301;-0,018;2

-873;0,441;-0,065;2

-912;0,359;0,081;2

-942;0,323;-0,095;2

-964;0,422;0,036;2

-981;0,34;-0,054;2

-994;0,476;0,006;2

-1010;0,388;0,095;2

-1029;0,341;-0,061;2

-1050;0,51;-0,011;2

-1073;0,295;0,009;2

-1093;0,37;-0,069;2

-1113;0,426;0,094;2

-1128;0,562;-0,045;2

-1143;1,158;0,028;2

-1152;0,911;0,026;2

-1159;0,666;0,036;2

-1162;0,201;0,094;2

-1170;-0,209;-0,026;2

-1175;-0,309;-0,012;2

-1182;-0,719;0,053;2

-1188;-0,716;-0,079;2

-1187;-0,069;0,041;2

-1186;0,565;-0,009;2

-1185;0,926;-0,02;2

-1180;-0,162;0,09;2

-1171;-1,319;-0,067;2

-1159;-1,682;0,048;2

-1136;-1,753;0,008;2

-1108;-0,839;-0,038;2

-1067;-0,023;0,07;2

-1025;-0,017;-0,012;2

-972;0,104;0,076;2

-915;0,204;0,007;2

-846;-0,17;-0,055;2

-777;-0,596;0,068;2

-707;-1,142;-0,05;2

-646;-1,214;0,012;2

-592;-1,01;0,045;2

-554;-0,563;-0,082;2

-543;-0,167;0,06;2

-566;-0,113;0,001;2

-621;-0,187;-0,04;2

-690;0,032;0,069;2

-758;0,19;-0,086;2

-813;0,195;0,057;2

-859;0,261;-0,044;2

-895;-0,105;-0,037;2

-928;-0,238;0,095;2

-952;0,381;-0,072;2

-972;0,836;0,031;2

-990;1,322;-0,018;2

-1011;1,703;-0,053;2

-1038;1,242;0,084;2

-1060;0,818;-0,084;2

-1081;0,85;0,01;2

-1097;0,588;0,032;2

-1116;0,116;-0,069;2

-1131;-0,257;0,101;2

-1149;-0,14;-0,037;2

-1159;0,428;0,031;2

-1167;0,159;0,043;2

-1172;-0,397;-0,015;2

-1178;-1,053;0,071;2

-1185;-1,549;-0,014;2

-1191;-1,3;-0,031;2

-1194;-1,122;0,068;2

-1197;-1,043;-0,049;2

-1198;-1,135;0,059;2

-1202;-1,27;-0,002;2

-1203;-0,894;-0,029;2

-1198;-0,71;0,103;2

-1178;-0,574;-0,067;2

-1148;-0,296;0,04;2

-1109;-0,241;-0,01;2

-1062;0,689;-0,032;2

-1009;0,955;0,115;2

-949;0,867;-0,059;2

-887;1,364;-0,001;2

-819;1,627;0,006;2

-756;1,494;-0,041;2

-693;0,977;0,082;2

-629;0,87;0,001;2

-566;0,71;0,007;2

-515;0,305;0,001;2

-487;0,482;-0,114;2

-502;0,184;0,036;2

-552;-0,543;-0,003;2

-622;-0,633;-0,013;2

-694;-0,319;0,122;2

-757;-0,197;-0,068;2

-813;0,14;0,031;2

-853;0,036;-0,026;2

-887;0,387;-0,049;2

-908;0,114;0,05;2

-928;-0,499;-0,076;2

-950;-0,517;0,03;2

-977;-0,415;0,011;2

-1006;0,345;-0,088;2

-1033;0,719;0,089;2

-1053;0,514;-0,045;2

-1070;0,419;-0,003;2

-1086;0,134;0,054;2

-1101;0,061;-0,098;2

-1114;-0,15;0,054;2

-1123;-0,378;-0,01;2

-1130;-0,221;0,014;2

-1138;-0,21;0,068;2

-1148;-0,418;-0,088;2

-1157;-0,68;0,064;2

-1160;-0,863;-0,008;2

-1158;-1,075;-0,058;2

-1163;-1,005;0,043;2

-1175;-0,661;-0,091;2

-1191;-0,802;0,041;2

-1199;-0,919;-0,006;2

-1198;-0,913;-0,07;2

-1177;-0,953;0,046;2

-1147;-0,81;-0,014;2

-1112;-0,528;0,028;2

-1073;-0,493;0,002;2

-1024;0,119;-0,069;2

-972;0,087;0,093;2

-917;-0,206;-0,048;2

-866;-0,071;-0;2

-808;-0,288;0,063;2

-743;-0,15;-0,097;2

-670;-0,025;0,072;2

-594;0,043;-0,058;2

-532;0,665;-0,025;2

-491;0,76;0,061;2

-481;0,365;-0,052;2

-502;0,344;0,048;2

-554;0,668;-0,013;2

-618;0,938;-0,012;2

-688;0,637;0,092;2

-751;0,474;-0,043;2

-802;0,817;-0,02;2

-844;0,703;-0;2

-876;0,342;-0,025;2

-906;-0,122;0,102;2

-930;-0,07;-0,077;2

-955;0,211;-0,003;2

-973;0,181;0,026;2

-994;0,413;-0,058;2

-1015;0,499;0,066;2

-1035;0,508;-0,014;2

-1054;0,594;-0,014;2

-1072;0,406;0,056;2

-1088;0,226;-0,048;2

-1102;-0,229;0,031;2

-1117;-0,56;-0,015;2

-1125;-0,293;-0,025;2

-1136;-0,284;0,105;2

-1143;-0,408;-0,076;2

-1152;-0,62;0,04;2

-1158;-0,797;-0,015;2

-1167;-0,342;-0,061;2

-1172;0,057;0,095;2

-1176;0,652;-0,073;2

-1178;1;0,026;2

-1183;0,965;0,024;2

-1183;0,77;-0,088;2

-1183;-0,206;0,088;2

-1164;-0,641;-0,055;2

-1140;-1,085;-0,022;2

-1106;-0,78;0,012;2

-1069;-0,491;-0,072;2

-1026;-0,73;0,145;2

-978;-0,906;-0,042;2

-921;-0,452;-0,012;2

-857;-0,22;0,077;2

-792;0,083;-0,061;2

-727;0,496;0,14;2

-667;0,84;-0,02;2

-608;1,133;0,004;2

-548;0,671;0,088;2

-507;0,181;-0,079;2

-488;-0,391;0,055;2

-517;-0,756;-0,044;2

-569;-0,399;-0,067;2

-641;-0,266;0,079;2

-710;-0,258;-0,051;2

-776;-0,248;0,006;2

-831;0,016;0,025;2

-875;0,521;-0,081;2

-907;0,318;0,106;2

-929;0,179;-0,119;2

-942;0,171;0,007;2

-960;0,11;0,082;2

-977;0,394;-0,076;2

-1001;0,594;0,088;2

-1024;0,621;-0,026;2

-1045;0,662;0,026;2

-1066;0,86;0,068;2

-1088;0,748;-0,096;2

-1110;0,542;0,094;2

-1130;0,264;0,002;2

-1142;-0,123;-0,03;2

-1148;-0,423;0,094;2

-1154;-0,552;-0,083;2

-1155;-0,38;0,037;2

-1159;-0,521;0,036;2

-1160;-0,312;-0,07;2

-1166;-1,001;0,126;2

-1169;-1,16;-0,049;2

-1181;-0,934;0,035;2

-1190;-0,747;0,025;2

-1196;-0,21;-0,075;2

-1197;0,095;0,223;2

-1192;0,049;0,016;2

-1177;0,536;-0,023;2

-1153;0,99;0,039;2

-1127;1,009;-0,083;2

-1090;0,958;0,115;2

-1055;0,455;-0,026;2

-1010;0,284;-0,015;2

-959;0,417;0,08;2

-902;0,325;-0,095;2

-840;-0,14;0,058;2

-780;-0,683;-0,009;2

-716;-0,756;-0,047;2

-657;-0,816;0,09;2

-598;-0,735;-0,086;2

-550;-0,406;0,025;2

-514;-0,332;0,006;2

-509;0,029;-0,064;2

-532;0,134;0,099;2

-583;0,104;-0,12;2

-643;0,09;-0,007;2

-710;0,021;0,006;2

-771;0,123;-0,07;2

-821;-0,21;0,082;2

-864;-0,374;-0,055;2

-900;0,103;0,003;2

-930;0,317;-0,032;2

-957;0,495;-0,072;2

-980;0,057;0,064;2

-996;-0,832;-0,008;2

-1014;-1,217;-0,042;2

-1030;-1,151;0,06;2

-1053;-0,793;-0,109;2

-1072;-0,752;0,028;2

-1096;-0,765;0,011;2

-1114;-0,299;-0,022;2

-1129;-0,172;0,097;2

-1134;0,132;-0,071;2

-1137;0,506;0,03;2

-1133;0,377;0,031;2

-1133;0,108;-0,075;2

-1136;-0,481;0,089;2

-1146;-0,99;-0,039;2

-1156;-0,871;-0,001;2

-1163;-0,789;0,05;2

-1174;-0,628;-0,13;2

-1178;-0,441;0,056;2

-1185;-0,129;-0,037;2

-1189;-0,231;0,021;2

-1184;-0,145;0,072;2

-1168;0,32;-0,099;2

-1134;0,182;0,061;2

-1097;-0,032;-0,006;2

-1052;-0,066;-0,025;2

-1006;-0,595;0,088;2

-953;-0,978;-0,053;2

-899;-1,248;0,026;2

-842;-1,043;0,018;2

-786;-0,561;-0,06;2

-722;-0,173;0,083;2

-661;0,185;-0,107;2

-592;0,806;0,007;2

-536;0,544;0,011;2

-491;0,677;-0,091;2

-478;0,701;0,021;2

-501;0,243;-0,029;2

-555;-0,036;-0,023;2

-626;-0,346;0,056;2

-703;-0,168;-0,064;2

-767;0,202;0,096;2

-825;0,524;-0,035;2

-867;0,845;-0,021;2

-902;0,681;0,064;2

-930;0,417;-0,077;2

-956;0,449;0,049;2

-976;0,106;-0,002;2

-990;0,134;-0,029;2

-1001;0,313;0,11;2

-1014;0,533;-0,075;2

-1036;0,489;0,176;2

-1064;-0,194;0,01;2

-1096;-0,465;-0,084;2

-1118;-0,967;0,089;2

-1133;-0,626;-0,058;2

-1137;0,095;0,014;2

-1143;0,09;0,036;2

-1149;-0,081;-0,092;2

-1152;0,519;0,067;2

-1160;0,487;-0,047;2

-1164;0,083;-0,006;2

-1171;-0,439;0,076;2

-1175;-0,238;-0,121;2

-1177;-0,266;0,05;2

-1176;-0,033;-0,011;2

-1180;0,476;-0,037;2

-1184;0,559;0,007;2

-1192;0,495;-0,062;2

-1191;0,802;0,052;2

-1174;1,002;0,011;2

-1134;1,175;-0,057;2

-1086;1,185;0,094;2

-1032;1,016;-0,077;2

-975;1,084;0,037;2

-917;1,018;-0,005;2

-857;0,973;-0,076;2

-800;0,82;0,086;2

-745;0,783;-0,031;2

-691;0,97;-0,015;2

-631;1,003;0,083;2

-575;0,664;-0,096;2

-526;0,332;0,044;2

-507;0,251;-0,042;2

-527;-0,029;-0,048;2

-585;-0,213;0,057;2

-652;-0,247;-0,087;2

-720;-0,704;0,052;2

-775;-0,896;-0,009;2

-825;-0,346;-0,043;2

-866;-0,102;0,077;2

-909;0,04;-0,082;2

-937;0,186;0,029;2

-961;-0,063;-0,01;2

-977;0,096;-0,053;2

-991;0,288;0,078;2

-1006;0,359;-0,069;2

-1020;0,687;0,014;2

-1036;0,347;0,095;2

-1052;0,346;-0,046;2

-1076;0,391;0,088;2

-1093;0,501;0,042;2

-1106;0,418;-0,028;2

-1115;0,119;0,045;2

-1119;0,045;-0,088;2

-1126;-0,03;0,085;2

-1131;-0,04;-0,042;2

-1141;0,649;-0,028;2

-1149;0,609;0,039;2

-1159;0,774;-0,061;2

-1163;0,679;0,05;2

-1171;0,113;0,023;2

-1174;0,101;-0,051;2

-1172;-0,351;0,066;2

-1156;-0,574;-0,124;2

-1131;-0,39;0,041;2

-1090;-0,469;0,005;2

-1045;-0,358;-0,026;2

-989;-0,04;0,074;2

-928;0,128;-0,056;2

-860;0,255;0,004;2

-785;0,288;-0,019;2

-708;0,858;-0,067;2

-634;0,913;0,065;2

-574;1,096;-0,053;2

-528;1,041;-0,001;2

-501;0,307;0,056;2

-499;0,216;-0,057;2

-534;0,088;0,005;2

-591;0,015;-0,012;2

-661;0,272;-0,009;2

-729;-0,375;0,059;2

-788;-0,396;-0,059;2

-830;-0,015;0,011;2

-867;-0,145;-0,002;2

-895;-0,16;-0,037;2

-922;0,081;0,051;2

-946;0,094;-0,083;2

-974;0,173;0,03;2

-1000;-0,086;0,013;2

-1021;-0,224;-0,072;2

-1044;-0,312;0,073;2

-1061;0,253;-0,045;2

-1078;0,483;-0,002;2

-1096;0,287;0,085;2

-1112;0,44;-0,098;2

-1120;0,153;0,1;2

-1125;0,26;-0,02;2

-1134;0,69;0,015;2

-1146;0,207;0,056;2

-1160;0,021;-0,089;2

-1173;0,049;0,019;2

-1182;-0,37;-0,005;2

-1185;-0,05;-0,025;2

-1184;0,325;0,077;2

-1180;0,506;-0,04;2

-1177;0,551;0,012;2

-1171;0,567;0,058;2

-1160;0,638;-0,057;2

-1142;0,527;0,061;2

-1119;0,266;-0,054;2

-1088;0;0,014;2

-1053;-0,359;0,026;2

-1002;-0,807;-0,078;2

-945;-1,312;0,085;2

-880;-1,295;-0,051;2

-815;-1,091;-0,015;2

-747;-0,97;0,018;2

-681;-0,636;-0,124;2

-613;-0,223;0,08;2

-553;0,264;-0,012;2

-515;0,378;-0,044;2

-514;0,136;0,098;2

-550;0,469;-0,083;2

-615;1,071;0,043;2

-687;0,675;0,01;2

-756;0,394;-0,023;2

-819;0,561;0,111;2

-867;1,001;-0,067;2

-906;1,054;0,022;2

-935;1,068;0,013;2

-957;1,224;-0,051;2

-978;0,663;0,115;2

-1001;0,574;-0,054;2

-1027;0,538;-0,001;2

-1050;0,09;0,059;2

-1073;0,19;-0,12;2

-1091;0,118;0,122;2

-1110;-0,189;-0,05;2

-1127;-0,297;-0,005;2

-1142;-0,02;0,06;2

-1151;0,129;-0,085;2

-1153;-0,235;0,07;2

-1155;-0,187;-0,022;2

-1156;-0,023;-0,018;2

-1160;-0,158;0,067;2

-1167;0,005;-0,06;2

-1180;-0,074;0,055;2

-1191;-0,195;-0,007;2

-1201;-0,049;-0,04;2

-1204;-0,124;0,095;2

-1206;-0,035;-0,067;2

-1204;0,035;0,04;2

-1204;0,224;0,018;2

-1196;0,756;-0,081;2

-1184;0,605;0,079;2

-1157;0,542;-0,045;2

-1126;0,722;0,026;2

-1088;0,776;-0,024;2

-1052;0,662;-0,094;2

-1004;0,285;0,068;2

-957;0,138;0,012;2

-898;0,248;0,007;2

-842;0,069;0,134;2

-777;-0,249;-0,07;2

-715;-0,522;0,072;2

-649;-0,63;-0,006;2

-589;-0,741;-0,062;2

-542;-0,925;0,055;2

-517;-0,67;-0,087;2

-535;-1,237;0,059;2

-584;-1,992;0,02;2

-652;-1,49;-0,043;2

-722;-1,277;0,085;2

-782;-1,024;-0,073;2

-829;-0,832;0,02;2

-866;-0,643;0,023;2

-898;-0,076;-0,038;2

-920;0,357;0,049;2

-943;0,462;-0,063;2

-959;0,381;0,02;2

-984;0,722;0,053;2

-1012;0,783;-0,132;2

-1039;0,028;0,075;2

-1064;-1,063;0,004;2

-1076;-1,685;-0,004;2

-1087;-1,633;0,094;2

-1097;-0,714;-0,058;2

-1108;-0,118;0,063;2

-1117;0,14;-0,021;2

-1126;0,021;-0,052;2

-1134;-0,099;0,086;2

-1144;-0,036;-0,059;2

-1152;0,076;0,022;2

-1165;-0,048;0,017;2

-1172;0,518;-0,062;2

-1182;1,045;0,081;2

-1194;1,367;-0,021;2

-1206;1,437;0,011;2

-1217;1,384;0,002;2

-1222;1,085;-0,063;2

-1212;0,771;0,06;2

-1190;0,396;-0,037;2

-1159;0,387;-0;2

-1121;0,181;0,055;2

-1087;0,083;-0,096;2

-1050;-0,101;0,089;2

-1014;0,009;-0,051;2

-970;0,133;-0,029;2

-924;-0,04;0,073;2

-867;0,13;-0,159;2

-802;-0,266;0,091;2

-731;-0,428;0,008;2

-653;0,022;-0,034;2

-593;0,296;0,067;2

-555;0,451;-0,081;2

-542;0,311;0,032;2

-559;0,61;0,004;2

-600;0,808;-0,034;2

-656;0,162;0,117;2

-712;0,017;-0,054;2

-769;0,15;0,023;2

-814;-0,069;0,028;2

-861;0,037;-0,053;2

-898;-0,172;0,049;2

-936;-0,216;-0,025;2

-965;-0,189;-0,017;2

-995;-0,681;0,065;2

-1017;-0,935;-0,098;2

-1036;-1,057;0,054;2

-1053;-0,959;-0,016;2

-1067;-0,517;-0,035;2

-1082;-0,458;0,046;2

-1098;-0,525;-0,083;2

-1114;-0,61;0,064;2

-1123;-0,602;-0,003;2

-1129;-0,299;-0,053;2

-1129;0,127;0,128;2

-1135;0,426;-0,042;2

-1139;0,251;0,041;2

-1154;-0,343;0,024;2

-1168;-0,651;-0,05;2

-1182;-0,852;0,087;2

-1192;-0,823;-0,091;2

-1202;-0,314;0,005;2

-1203;0,356;0,044;2

-1201;0,684;-0,077;2

-1192;0,145;0,065;2

-1177;-0,34;0,004;2

-1155;-0,694;0,004;2

-1129;-1,193;0,054;2

-1108;-1,046;-0,066;2

-1075;-0,755;0,071;2

-1040;-0,934;0,019;2

-989;-0,678;-0,001;2

-928;-0,714;0,105;2

-870;-0,455;-0,053;2

-808;-0,248;0,016;2

-749;-0,243;-0;2

-691;0,34;0,032;2

-640;0,04;0,132;2

-593;0,512;-0,034;2

-566;1,568;0,005;2

-556;2,078;0,005;2

-582;2,34;-0,116;2

-630;1,788;0,058;2

-688;0,925;-0,057;2

-746;0,328;-0,007;2

-799;-0,127;0,03;2

-846;0,019;-0,091;2

-884;0,121;0,055;2

-919;0,088;-0,031;2

-942;0,07;-0,019;2

-969;-0,399;0,083;2

-996;-0,199;-0,081;2

-1025;0,19;0,022;2

-1052;0,465;0,027;2

-1075;1,274;-0,013;2

-1087;1,384;0,077;2

-1096;1,516;-0,06;2

-1101;1,064;0,024;2

-1109;0,352;0,019;2

-1118;0,27;-0,025;2

-1130;-0,504;0,074;2

-1138;-0,694;-0,08;2

-1149;-0,224;0,016;2

-1158;-0,448;0,105;2

-1171;-0,475;-0,079;2

-1180;0,023;0,066;2

-1191;0,041;-0,032;2

-1198;-0,173;0,118;2

-1203;-0,633;0,051;2

-1202;-0,85;-0,077;2

-1185;-1,074;0,05;2

-1156;-1,459;-0,022;2

-1122;-1,331;-0,025;2

-1086;-1,442;0,02;2

-1048;-0,961;-0,005;2

-1003;-0,292;0,055;2

-953;0,016;0,021;2

-897;0,163;-0,051;2

-839;-0,604;0,07;2

-777;-0,871;-0,086;2

-716;-0,667;0,036;2

-651;-0,582;-0,016;2

-591;-0,366;-0,011;2

-547;-0,698;0,084;2

-524;-1,021;-0,068;2

-533;-0,632;0,031;2

-570;0,045;0,004;2

-629;0,092;-0,078;2

-693;-0,312;0,061;2

-764;-0,168;-0,02;2

-819;0,513;-0,004;2

-866;0,817;0,07;2

-903;0,734;-0,099;2

-932;0,176;0,061;2

-952;0,005;-0,024;2

-973;0,232;-0,024;2

-990;0,079;0,077;2

-1011;-0,394;0,047;2

-1034;-0,605;0,071;2

-1059;-0,794;0,046;2

-1080;-0,758;-0,058;2

-1096;-1,165;0,101;2

-1098;-1,05;-0,045;2

-1106;-0,445;0,036;2

-1112;-0,226;0,021;2

-1121;0,061;-0,025;2

-1135;0,006;0,022;2

-1143;-0,411;-0,045;2

-1152;-0,653;0,005;2

-1160;-0,394;0,054;2

-1172;-0,019;-0,095;2

-1181;-0,128;0,072;2

-1188;-0,103;-0,014;2

-1187;-0,069;-0,017;2

-1173;-0,52;0,103;2

-1152;-0,399;-0,095;2

-1120;-0,125;0,04;2

-1081;-0,019;0,042;2

-1031;0,16;-0,067;2

-979;0,202;0,072;2

-920;0,569;-0,058;2

-867;0,669;0,016;2

-814;0,385;-0,025;2

-762;0,288;-0,052;2

-707;-0,034;0,082;2

-647;-0,121;-0,079;2

-588;-0,158;0,011;2

-541;-0,369;0,016;2

-521;-0,41;-0,069;2

-535;-0,533;0,061;2

-582;-0,898;-0,005;2

-643;-0,827;-0,016;2

-712;-0,363;0,08;2

-773;0,234;-0,092;2

-829;0,837;0,068;2

-872;1,466;-0,003;2

-911;1,468;-0,016;2

-939;0,458;0,105;2

-964;0,385;-0,072;2

-985;0,555;0,06;2

-1008;0,028;0,005;2

-1028;-0,303;-0,021;2

-1049;-0,385;0,083;2

-1063;-0,726;-0,071;2

-1076;-0,899;0,021;2

-1086;-1,101;-0,002;2

-1101;-1,139;-0,072;2

-1112;-1,022;0,08;2

-1129;-0,756;-0,054;2

-1139;-0,455;0,014;2

-1152;-0,486;0,034;2

-1164;-0,315;-0,082;2

-1172;-0,084;0,087;2

-1179;0,168;-0,026;2

-1176;0,625;-0,011;2

-1169;0,52;0,077;2

-1153;0,27;-0,081;2

-1132;0,125;0,09;2

-1106;0,023;-0,002;2

-1074;-0,129;-0,034;2

-1039;-0,253;0,064;2

-991;0,05;-0,083;2

-943;-0,047;0,053;2

-883;-0,172;0,013;2

-817;0,423;-0,045;2

-747;0,474;0,095;2

-680;0,441;-0,087;2

-609;0,366;0,015;2

-552;0,377;-0,004;2

-500;0,782;-0,084;2

-466;0,523;0,076;2

-457;0,597;-0,071;2

-485;0,624;0,009;2

-547;0,362;0,018;2

-627;0,461;-0,085;2

-702;0,293;0,068;2

-768;0,383;-0,027;2

-817;0,573;-0,009;2

-857;0,414;0,081;2

-887;0,555;-0,074;2

-914;0,146;0,077;2

-938;-0,496;0,009;2

-957;-0,339;-0,023;2

-980;-0,054;0,09;2

-1004;0,479;-0,096;2

-1027;0,866;0,049;2

-1048;0,374;0,026;2

-1065;0,315;-0,062;2

-1077;-0,215;0,091;2

-1093;-0,483;0,006;2

-1104;-0,44;0,004;2

-1122;-0,167;0,052;2

-1134;0,221;-0,075;2

-1145;-0,06;0,057;2

-1155;-0,382;-0,024;2

-1166;-0,393;-0,017;2

-1183;-0,683;0,063;2

-1193;-0,484;-0,08;2

-1200;-0,614;0,048;2

-1198;-0,734;-0,029;2

-1196;-0,466;-0,021;2

-1196;-0,578;0,056;2

-1193;-0,606;-0,093;2

-1180;-0,571;0,08;2

-1158;-0,629;0,004;2

-1124;-0,198;-0,051;2

-1087;-0,097;0,065;2

-1053;0,04;-0,037;2

-1003;-0,19;0,023;2

-948;-0,389;0,038;2

-884;-0,446;-0,081;2

-823;-0,674;0,078;2

-762;-0,76;-0,086;2

-700;-0,547;-0,009;2

-634;-0,611;0,039;2

-578;-0,542;-0,094;2

-530;-0,573;0,104;2

-513;-0,401;-0,031;2

-524;0,189;-0,01;2

-570;0,171;0,024;2

-630;0,008;-0,102;2

-696;-0,023;0,056;2

-765;0,004;0,066;2

-824;-0,143;-0,018;2

-874;-0,528;0,096;2

-914;-0,506;-0,096;2

-940;-0,468;0,029;2

-964;-0,546;-0,001;2

-985;-0,483;-0,035;2

-1005;-0,855;0,085;2

-1027;-1,043;-0,112;2

-1046;-1,036;-0,012;2

-1068;-1,089;0,011;2

-1085;-0,793;-0,072;2

-1103;-0,46;0,081;2

-1119;-0,303;-0,058;2

-1133;-0,093;0,006;2

-1147;-0,402;0,076;2

-1154;-0,114;-0,071;2

-1162;-0,165;0,112;2

-1165;-0,555;-0,015;2

-1171;-0,151;0,015;2

-1174;-0,073;0,072;2

-1178;0,032;-0,125;2

-1180;0,381;0,043;2

-1182;0,477;-0,052;2

-1183;0,374;-0,036;2

-1182;0,188;0,066;2

-1185;0,275;-0,067;2

-1178;0,105;0,081;2

-1169;0,005;0,013;2

-1150;0,726;-0,045;2

-1127;0,986;0,086;2

-1094;0,605;-0,018;2

-1056;0,602;0,012;2

-1009;0,176;0,005;2

-959;0,457;-0,067;2

-904;0,545;0,065;2

-846;0,163;-0,043;2

-782;-0,023;0,078;2

-724;-0,128;0,069;2

-665;0,088;-0,119;2

-619;0,199;0,067;2

-582;0,172;-0,024;2

-565;0,338;-0,016;2

-567;0,488;0,05;2

-600;0,977;-0,064;2

-652;1,524;0,08;2

-715;0,668;-0,015;2

-775;0,136;-0,038;2

-826;0,269;0,081;2

-870;0,649;-0,106;2

-908;0,851;-0,078;2

-944;0,566;0,092;2

-969;0,293;-0,006;2

-988;-0,048;0,075;2

-1001;-0,448;-0,054;2

-1016;-0,506;0,019;2

-1038;-0,543;0,038;2

-1061;-0,089;-0,098;2

-1084;-0,376;0,077;2

-1105;-0,555;-0,024;2

-1124;-0,269;0,031;2

-1138;-0,175;0,057;2

-1149;0,118;-0,081;2

-1152;0,324;0,02;2

-1158;0,546;-0,04;2

-1167;0,948;-0,102;2

-1177;0,76;0,066;2

-1186;0,419;-0,114;2

-1193;0,155;0,044;2

-1192;-0,258;0,011;2

-1194;-0,134;-0,053;2

-1202;-0,324;0,084;2

-1206;-0,198;-0,084;2

-1212;0,164;0,022;2

-1205;-0,142;-0,033;2

-1189;-0,057;-0,095;2

-1162;0,208;0,088;2

-1131;-0,068;-0,036;2

-1095;-0,168;-0,007;2

-1058;-0,329;0,103;2

-1012;-0,357;-0,078;2

-962;-0,143;0,104;2

-903;0,3;-0,048;2

-842;0,638;-0,012;2

-772;0,135;0,066;2

-708;-0,075;-0,055;2

-641;-0,063;0,064;2

-593;-0,555;-0,005;2

-564;-0,504;-0,025;2

-568;-0,453;0,087;2

-596;-0,347;-0,08;2

-647;-0,056;0,028;2

-704;-0,239;0,007;2

-758;0,012;-0,028;2

-810;0,084;0,116;2

-846;0,253;-0,052;2

-884;0,412;0,016;2

-916;0,322;0,057;2

-950;0,584;-0,064;2

-986;0,755;0,095;2

-1012;0,802;-0,018;2

-1035;0,876;0,013;2

-1046;0,622;0,058;2

-1063;0,511;-0,078;2

-1082;0,178;0,078;2

-1107;-0,162;-0,008;2

-1122;0,293;-0,001;2

-1130;0,379;0,052;2

-1128;0,702;-0,053;2

-1130;0,673;0,048;2

-1136;0,064;0,061;2

-1151;-0,31;-0,045;2

-1167;-0,434;0,076;2

-1183;-0,017;-0,054;2

-1191;-0,154;0,051;2

-1198;-0,548;-0,037;2

-1200;-0,09;-0,114;2

-1200;0,152;0,083;2

-1198;0,38;-0,079;2

-1193;0,496;-0,006;2

-1189;0,489;0,029;2

-1188;0,508;-0,09;2

-1179;0,348;-0,004;2

-1162;0,222;-0,02;2

-1133;-0,089;-0,01;2

-1095;-0,435;0,065;2

-1047;0,22;-0,124;2

-993;0,706;0,072;2

-935;0,584;-0,026;2

-873;0,624;-0,018;2

-820;0,367;0,051;2

-768;0,331;-0,088;2

-715;-0,182;-0,009;2

-657;-0,395;0,028;2

-602;-0,157;-0,057;2

-558;-0,25;0,062;2

-543;-0,121;-0,056;2

-567;-0,075;0,012;2

-614;-0,036;0,011;2

-677;0,386;-0,059;2

-735;0,344;0,079;2

-785;0,208;-0,016;2

-825;0,256;-0,01;2

-861;0,287;0,018;2

-898;0,619;-0,082;2

-929;0,085;0,137;2

-958;-0,397;-0,032;2

-980;-0,144;-0,041;2

-1006;-0,204;0,044;2

-1029;0,091;-0,082;2

-1054;0,138;0,073;2

-1069;-0,056;-0,014;2

-1082;-0,097;-0,017;2

-1091;-0,132;0,077;2

-1106;-0,016;-0,067;2

-1123;-0,056;0,072;2

-1141;-0,185;0,004;2

-1148;0,058;-0,064;2

-1150;0,006;0,039;2

-1148;-0,22;-0,07;2

-1147;-0,294;0,009;2

-1148;-0,268;0,049;2

-1151;-0,304;-0,086;2

-1156;-0,344;0,082;2

-1164;-0,306;-0,044;2

-1179;-0,376;0,008;2

-1190;-0,553;0,029;2

-1194;-0,158;-0,054;2

-1181;-0,112;0,079;2

-1162;-0,213;-0,027;2

-1133;0,014;-0;2

-1112;-0,194;0,064;2

-1087;-0,231;-0,076;2

-1057;-0,152;0,026;2

-1018;-0,141;0,07;2

-971;0,002;-0,083;2

-930;-0,161;0,077;2

-887;0,168;-0,073;2

-840;0,409;0,036;2

-780;0,685;0,021;2

-719;1,183;-0,099;2

-664;0,777;0,059;2

-614;0,426;-0,092;2

-575;0,412;-0,032;2

-548;0,347;0,002;2

-545;0,322;-0,071;2

-568;0,713;0,067;2

-614;0,889;-0,038;2

-674;0,679;-0,015;2

-741;0,179;0,059;2

-796;-0,29;-0,071;2

-845;-0,502;0,076;2

-880;-0,487;0,034;2

-904;-0,423;-0,034;2

-923;-0,257;0,086;2

-940;-0,462;-0,066;2

-959;-0,479;0,055;2

-983;0,078;-0,033;2

-1011;0,713;0,063;2

-1037;0,548;0,107;2

-1060;0,23;-0,077;2

-1065;-0,295;0,078;2

-1068;-0,715;0,024;2

-1066;-1,14;-0,072;2

-1075;-1,331;0,078;2

-1088;-0,99;-0,049;2

-1105;-0,748;0,026;2

-1115;-0,758;0,029;2

-1125;-0,071;-0,092;2

-1131;0,651;0,105;2

-1138;0,868;-0,04;2

-1141;0,881;0,056;2

-1142;0,477;0,062;2

-1141;0,123;-0,069;2

-1141;-0,262;0,059;2

-1142;-0,81;-0,016;2

-1128;-0,732;-0,029;2

-1107;-0,626;0,081;2

-1064;-0,44;-0,053;2

-1013;0,043;0,089;2

-958;0,222;-0,013;2

-906;0,54;-0,054;2

-855;0,572;0,066;2

-800;0,827;-0,072;2

-746;0,852;0,027;2

-688;0,645;0,033;2

-635;0,855;-0,078;2

-574;0,298;0,076;2

-518;-0,095;-0,069;2

-469;-0,014;-0,005;2

-452;-0,607;0,214;2

-469;-0,948;-0,078;2

-520;-0,541;0,125;2

-585;-0,35;-0,024;2

-649;0,095;-0,028;2

-707;0,184;0,06;2

-763;0,562;-0,087;2

-806;0,753;0,061;2

-850;1,015;-0,024;2

-881;1,502;-0,033;2

-902;1,524;0,087;2

-919;1,313;-0,081;2

-934;1,034;-0,008;2

-955;1,152;0,014;2

-975;1,329;-0,05;2

-998;0,922;0,086;2

-1017;0,691;-0,028;2

-1036;0,601;0,023;2

-1053;0,466;0,015;2

-1069;0,513;-0,063;2

-1080;0,321;0,067;2

-1091;0,108;-0,042;2

-1098;0,36;-0,006;2

-1108;0,094;0,054;2

-1116;-0,275;0,012;2

-1124;-0,24;0,058;2

-1132;-0,304;-0,036;2

-1136;-0,219;-0,025;2

-1140;-0,807;0,082;2

-1145;-0,991;-0,072;2

-1154;-1,009;0,036;2

-1159;-1,311;-0,01;2

-1166;-0,977;-0,052;2

-1166;-0,678;0,095;2

-1155;-0,397;-0,056;2

-1132;-0,32;0,048;2

-1097;-0,518;0,016;2

-1056;-0,531;-0,042;2

-1008;-0,423;0,072;2

-960;-0,43;-0,072;2

-910;-0,226;0,021;2

-862;0,025;0,058;2

-806;0,258;-0,073;2

-748;0,075;0,077;2

-680;-0,063;-0,031;2

-616;0,066;-0,011;2

-553;0,141;0,037;2

-502;0,642;-0,114;2

-474;0,691;0,058;2

-482;0,38;0,004;2

-523;0,099;-0,002;2

-589;-0,167;0,119;2

-659;-0,318;-0,067;2

-722;-0,56;0,009;2

-770;-0,791;0,054;2

-808;-0,663;-0,063;2

-843;-0,992;0,06;2

-876;-0,562;-0,059;2

-908;-0,295;0,041;2

-940;-0,363;0,067;2

-971;0,009;-0,026;2

-994;-0,059;0,062;2

-1018;-0,069;-0,076;2

-1032;-0,117;-0,033;2

-1045;-0,165;0,04;2

-1057;-0,104;-0,078;2

-1073;-0,167;0,079;2

-1089;-0,292;-0,013;2

-1104;-0,473;-0,009;2

-1117;-0,722;0,062;2

-1132;-0,472;-0,062;2

-1143;-0,584;0,055;2

-1153;-0,678;-0,002;2

-1162;-0,24;-0,064;2

-1162;-0,704;0,059;2

-1162;-1,008;-0,086;2

-1164;-0,798;0,053;2

-1169;-0,509;-0,002;2

-1178;-0,408;-0,097;2

-1181;-0,634;0,133;2

-1171;-0,525;-0,046;2

-1141;-0,308;0,015;2

-1102;0;0,014;2

-1059;0,194;-0,103;2

-1018;0,077;0,066;2

-970;0,327;-0,063;2

-921;0,449;0,025;2

-857;0,448;0,047;2

-792;0,114;-0,034;2

-722;-0,025;0,085;2

-657;-0,634;-0,004;2

-596;-0,757;-0,031;2

-544;-0,534;0,059;2

-500;-0,098;-0,043;2

-474;-0,189;0,052;2

-472;-0,349;0,009;2

-503;-0,228;-0,02;2

-556;-0,245;0,103;2

-620;0,089;-0,079;2

-688;0,127;0,023;2

-746;0,336;0,015;2

-806;0,594;-0,113;2

-859;0,334;0,088;2

-908;0,524;-0,044;2

-945;0,67;0;2

-969;0,735;0,052;2

-979;0,656;-0,093;2

-988;0,853;0,077;2

-999;1,277;-0,046;2

-1021;0,912;-0,005;2

-1048;0,349;0,076;2

-1072;0,201;-0,092;2

-1090;-0,484;0,051;2

-1103;-1,02;0,016;2

-1115;-0,832;-0,012;2

-1126;-0,682;0,058;2

-1140;-0,39;-0,066;2

-1151;-0,029;0,037;2

-1156;-0,008;0,002;2

-1165;0,134;-0,059;2

-1172;0,348;0,115;2

-1185;0,657;-0,025;2

-1190;1,032;-0,001;2

-1197;1,512;0,034;2

-1196;1,744;-0,058;2

-1193;1,246;0,112;2

-1176;0,797;0,02;2

-1154;0,628;0,022;2

-1117;0,526;0,053;2

-1074;0,872;-0,085;2

-1024;1,347;0,075;2

-971;1,16;-0,023;2

-912;0,839;0,042;2

-848;0,427;0,084;2

-783;0,555;-0,076;2

-722;0,32;0,037;2

-660;-0,332;-0,029;2

-599;-0,149;-0,048;2

-549;-0,005;0,041;2

-515;-0,046;-0,042;2

-516;0,745;0,029;2

-554;0,941;0,015;2

-622;0,867;-0,063;2

-690;-0,162;0,077;2

-757;-0,425;-0;2

-812;-0,481;0,068;2

-865;-0,625;0,054;2

-909;-0,271;-0,066;2

-942;-0,111;0,076;2

-962;-0,282;-0,066;2

-982;-0,124;0,018;2

-1006;-0,158;0,047;2

-1033;-0,016;-0,087;2

-1062;0,239;0,049;2

-1084;0,396;-0,025;2

-1099;0,698;-0,005;2

-1111;0,791;0,094;2

-1121;1,021;-0,046;2

-1133;0,974;0,055;2

-1144;0,913;-0,009;2

-1163;0,995;-0,052;2

-1180;0,053;0,064;2

-1193;-0,285;-0,066;2

-1194;0,239;-0,021;2

-1196;-0,062;0,04;2

-1192;-0,202;-0,04;2

-1197;0,34;0,069;2

-1205;0,393;-0,05;2

-1214;0,302;-0,007;2

-1217;-0,184;0,013;2

-1219;-0,3;-0,091;2

-1216;-0,271;0,068;2

-1203;-0,51;-0,035;2

-1174;-0,6;-0,001;2

-1136;-0,581;0,085;2

-1087;-0,24;-0,038;2

-1040;-0,078;0,135;2

-988;0,198;-0,017;2

-932;0,606;0,006;2

-877;0,773;0,074;2

-811;1,071;-0,125;2

-747;1,127;0,032;2

-680;1,159;0,02;2

-618;1,431;-0,064;2

-565;1,411;0,206;2

-523;1,309;-0,088;2

-496;1,481;0,024;2

-498;1,088;0,035;2

-528;0,852;-0,089;2

-587;0,675;0,072;2

-656;0,593;-0,067;2

-727;0,75;-0,053;2

-788;0,34;0,028;2

-837;0,14;-0,073;2

-883;-0,528;0,063;2

-916;-1,048;-0,042;2

-942;-0,642;-0,036;2

-961;-0,572;0,096;2

-973;-0,67;-0,08;2

-985;-1,187;0,125;2

-1006;-1,439;-0,004;2

-1030;-0,762;-0,02;2

-1051;-0,85;0,096;2

-1074;-0,943;-0,083;2

-1085;-0,672;0,04;2

-1101;-0,9;0,036;2

-1110;-0,755;-0,055;2

-1122;-0,587;0,063;2

-1128;-0,498;-0,016;2

-1139;-0,263;-0,011;2

-1148;-0,243;0,022;2

-1157;-0,365;-0,121;2

-1159;-0,486;0,09;2

-1162;-0,809;-0,002;2

-1163;-1,003;0,007;2

-1176;-1,181;0,084;2

-1188;-1,418;-0,096;2

-1198;-1,338;0,053;2

-1190;-1,154;0,049;2

-1167;-1,268;-0,016;2

-1128;-1,085;0,075;2

-1084;-0,487;-0,072;2

-1033;-0,309;0,037;2

-975;-0,199;0,017;2

-914;-0,253;-0,036;2

-860;-0,937;0,09;2

-810;-0,953;-0,061;2

-761;-0,33;0,032;2

-704;-0,605;-0,005;2

-638;-0,625;-0,096;2

-575;-0,459;0,115;2

-515;-0,286;-0,068;2

-479;-0,224;0,013;2

-482;-0,125;0,003;2

-528;-0,161;-0,069;2

-603;-0,442;0,085;2

-688;-0,036;-0,032;2

-767;0,242;0,041;2

-825;-0,228;0,081;2

-869;-0,058;-0,073;2

-902;0,531;0,054;2

-928;0,66;-0,009;2

-954;0,387;-0,033;2

-974;-0,243;0,042;2

-998;-0,611;-0,056;2

-1014;-0,418;0,07;2

-1032;-0,6;0,013;2

-1047;-0,268;-0,05;2

-1067;0,226;0,074;2

-1084;0,263;-0,054;2

-1103;0,594;-0,003;2

-1121;0,539;0,046;2

-1135;0,83;-0,029;2

-1149;0,557;0,113;2

-1156;0,479;-0,042;2

-1161;0,163;0,006;2

-1164;-0,138;-0,016;2

-1168;0,19;-0,073;2

-1170;-0,106;0,066;2

-1179;-0,3;-0,001;2

-1186;0,131;-0,013;2

-1196;0,199;0,092;2

-1186;0,267;-0,076;2

-1160;0,345;0,057;2

-1119;0,409;0,033;2

-1078;0,607;-0,079;2

-1034;0,477;0,077;2

-989;0,081;-0,047;2

-939;-0,04;0,025;2

-876;0,113;0,016;2

-814;0,35;-0,048;2

-749;0,139;0,087;2

-683;0,366;-0,058;2

-610;0,533;-0,003;2

-546;0,531;0,006;2

-498;0,817;-0,067;2

-477;0,559;0,053;2

-503;0,321;0;2

-553;0,406;-0;2

-626;-0,062;0,038;2

-702;-0,363;-0,071;2

-766;-0,293;0,072;2

-820;0,076;-0,054;2

-858;0,272;-0,008;2

-892;0,066;0,015;2

-926;0,064;-0,102;2

-957;-0,245;0,015;2

-986;-0,241;0,005;2

-1010;0,127;-0,053;2

-1026;0,113;0,072;2

-1043;0,199;-0,051;2

-1055;-0,102;0,01;2

-1074;-0,295;0,035;2

-1089;-0,126;-0,031;2

-1105;0,053;0,096;2

-1123;-0,008;-0,049;2

-1139;0,307;0,03;2

-1153;0,268;0,024;2

-1164;0,144;-0,139;2

-1172;0,021;0,078;2

-1178;-0,22;-0,033;2

-1186;0,095;-0,003;2

-1193;-0,268;0,063;2

-1204;0,136;-0,087;2

-1208;0,539;0,064;2

-1205;0,662;0,043;2

-1194;0,806;-0,015;2

-1190;0,77;0,087;2

-1187;0,787;-0,046;2

-1185;0,953;0,051;2

-1170;0,922;0,001;2

-1144;1,175;-0,076;2

-1103;1,036;0,089;2

-1053;1,038;-0,079;2

-1002;1,1;0,01;2

-941;1,193;0,015;2

-873;1,102;-0,024;2

-796;0,815;0,077;2

-721;0,818;-0,07;2

-654;0,777;-0,013;2

-596;0,524;0,072;2

-548;0,38;-0,079;2

-504;0,146;0,089;2

-486;0,117;0,001;2

-496;-0,104;-0,001;2

-543;-0,575;0,081;2

-611;-0,714;-0,089;2

-686;-1,086;0,034;2

-751;-1,014;-0,025;2

-808;-0,432;-0,082;2

-855;-0,287;0,094;2

-897;0,278;-0,086;2

-926;0,907;0,014;2

-948;1,128;-0,011;2

-966;1,004;-0,07;2

-985;0,716;0,066;2

-1011;0,597;-0,054;2

-1039;0,436;0,043;2

-1061;0,099;0,024;2

-1075;0,478;-0,041;2

-1092;0,168;0,043;2

-1108;-0,062;-0,022;2

-1130;-0,042;0,004;2

-1149;-0,493;0,06;2

-1167;-0,661;-0,101;2

-1173;-1,126;0,069;2

-1182;-0,985;-0,011;2

-1190;-0,5;-0,026;2

-1201;-0,227;0,101;2

-1212;-0,321;-0,037;2

-1220;-0,101;0,062;2

-1224;0,034;-0,008;2

-1228;0,298;-0,009;2

-1224;0,08;0,115;2

-1208;0,041;-0,066;2

-1179;-0,232;0,034;2

-1142;-0,877;0,009;2

-1105;-0,868;-0,152;2

-1060;-0,682;0,089;2

-1007;-0,677;-0,063;2

-945;-0,716;-0,043;2

-872;-0,77;0,052;2

-802;-0,573;-0,085;2

-735;-0,745;0,057;2

-675;-1,041;-0,034;2

-613;-1,064;-0,029;2

-559;-0,692;0,072;2

-512;-0,406;-0,089;2

-489;-0,54;0,086;2

-509;-0,677;-0,012;2

-564;-0,398;0,038;2

-635;-0,335;0,13;2

-708;-0,246;-0,052;2

-776;0,062;0,032;2

-831;-0,141;-0,014;2

-880;0,272;-0,033;2

-912;-0,158;0,073;2

-941;-0,224;-0,054;2

-962;0,207;-0,004;2

-985;0,325;0,022;2

-1008;0,229;-0,094;2

-1035;-0,051;0,05;2

-1052;-0,343;-0,029;2

-1074;-0,502;0,008;2

-1091;-0,354;0,049;2

-1110;-0,135;-0,034;2

-1130;-0,091;0,059;2

-1148;0,014;-0,025;2

-1161;0,127;-0,017;2

-1172;-0,014;0,088;2

-1179;-0,207;-0,076;2

-1187;-0,226;0,056;2

-1194;-0,075;0,013;2

-1199;0,306;-0,028;2

-1205;0,224;0,084;2

-1207;0,143;-0,077;2

-1206;0,21;-0,005;2

-1189;0,025;0,031;2

-1157;-0,244;-0,054;2

-1115;-0,08;0,088;2

-1064;0,004;0;2

-1014;0,113;-0,051;2

-956;0,233;0,01;2

-894;0,369;-0,057;2

-823;0,381;0,061;2

-749;0,502;-0,059;2

-676;0,944;-0,012;2

-611;0,956;0,067;2

-550;0,871;-0,094;2

-501;0,744;0,067;2

-477;0,887;-0,003;2

-487;1,02;-0,009;2

-530;0,729;0,054;2

-593;0,645;-0,063;2

-663;0,623;0,068;2

-728;0,329;0,014;2

-784;0,504;-0,039;2

-828;0,247;0,081;2

-869;0,136;-0,068;2

-906;0,217;0,034;2

-944;-0,116;0,1;2

-978;-0,192;-0,075;2

-1009;-0,45;0,052;2

-1028;-0,413;-0,03;2

-1044;-0,403;0,005;2

-1057;-0,377;0,052;2

-1072;-0,558;-0,015;2

-1085;-0,255;0,064;2

-1096;0,273;0,081;2

-1110;0,315;-0,013;2

-1126;0,473;0,065;2

-1142;0,808;-0,072;2

-1166;1,113;0,101;2

-1184;1,064;0,002;2

-1198;0,915;-0,02;2

-1204;0,725;0,104;2

-1211;0,482;-0,048;2

-1216;0,169;0,093;2

-1216;0,099;0,009;2

-1215;0,025;-0,075;2

-1212;-0,173;0,083;2

-1211;0,022;-0,014;2

-1205;0,219;0,012;2

-1196;0,088;0,082;2

-1173;0,238;-0,092;2

-1142;0,285;0,086;2

-1104;0,201;-0,04;2

-1056;0,226;0,002;2

-1005;0,114;0,062;2

-943;0,171;-0,048;2

-875;0,161;0,081;2

-806;0,169;-0,021;2

-734;0,329;-0,015;2

-669;0,272;0,083;2

-609;0,326;-0,114;2

-550;0,672;0,045;2

-500;0,374;0,01;2

-465;0,341;-0,056;2

-470;-0,002;-0,004;2

-512;-0,137;-0,115;2

-580;-0,229;0,014;2

-655;-0,444;0,011;2

-726;-0,383;-0,032;2

-788;-0,699;0,135;2

-845;-0,662;-0,085;2

-891;-0,484;-0,024;2

-931;-0,526;0,059;2

-959;-0,224;-0,065;2

-987;-0,398;0,082;2

-1005;-0,226;-0,029;2

-1027;0,224;0,007;2

-1044;-0,023;0,025;2

-1064;0,438;-0,112;2

-1081;0,301;0,042;2

-1103;-0,103;0,036;2

-1124;-0,018;-0,047;2

-1144;0,053;0,076;2

-1160;-0,056;-0,098;2

-1171;-0,212;0,087;2

-1185;-0,097;0,019;2

-1192;0,113;-0,043;2

-1203;0,082;0,028;2

-1208;0,074;-0,057;2

-1209;-0,1;0,019;2

-1210;-0,177;0,022;2

-1212;-0,199;-0,062;2

-1216;-0,231;0,089;2

-1218;0,104;-0,042;2

-1205;0,301;0,007;2

-1184;-0,066;0,048;2

-1155;0,284;-0,098;2

-1120;0,655;0,121;2

-1081;0,228;-0,027;2

-1030;-0,086;-0,032;2

-971;-0,182;0,098;2

-906;-0,144;-0,103;2

-844;0,005;0,051;2

-778;0,02;-0,002;2

-710;0,065;-0,063;2

-641;-0,065;0,084;2

-577;0,132;-0,064;2

-517;-0,152;0,054;2

-483;-0,555;-0,036;2

-478;-0,19;-0,061;2

-519;-0,011;0,073;2

-585;-0,063;-0,075;2

-666;-0,25;0,014;2

-740;-0,368;-0,032;2

-802;-0,202;-0,074;2

-852;0,005;0,066;2

-894;-0,052;-0,035;2

-924;-0,29;-0,015;2

-951;-0,561;0,065;2

-971;-0,428;-0,091;2

-997;0,197;0,116;2

-1020;0,395;-0,013;2

-1049;0,552;-0,017;2

-1080;0,607;0,057;2

-1103;0,766;-0,063;2

-1126;0,498;0,048;2

-1140;0,46;-0,002;2

-1152;0,768;-0,026;2

-1162;0,578;0,077;2

-1171;0,442;-0,057;2

-1180;0,707;0,04;2

-1182;0,788;0,038;2

-1185;0,902;-0,039;2

-1187;0,687;0,101;2

-1196;0,415;-0,067;2

-1204;0,314;-0,017;2

-1219;-0,035;0,017;2

-1225;-0,065;-0,12;2

-1228;-0,013;0,094;2

-1228;0,337;-0,036;2

-1232;0,806;-0,013;2

-1233;0,674;0,081;2

-1230;0,697;-0,112;2

-1211;0,706;0,066;2

-1187;0,446;-0,001;2

-1156;0,622;-0,029;2

-1121;0,428;0,086;2

-1084;0,172;-0,071;2

-1036;0,094;0,023;2

-990;-0,258;0,063;2

-946;-0,494;-0,051;2

-902;-0,47;0,097;2

-853;-0,248;-0,064;2

-790;0,027;0,024;2

-721;-0,112;0,011;2

-655;0,062;-0,095;2

-594;0,075;0,051;2

-545;0,151;-0,049;2

-505;0,417;0,004;2

-488;0,283;0,038;2

-506;0,46;-0,039;2

-555;0,437;0,082;2

-626;0,4;-0,026;2

-704;0,238;0,006;2

-769;0,078;0,08;2

-825;0,069;-0,079;2

-870;-0,238;0,071;2

-916;-0,369;0,034;2

-953;-0,608;-0,037;2

-976;-1,009;0,094;2

-996;-0,9;-0,072;2

-1007;-0,945;-0,003;2

-1025;-1,034;0,041;2

-1048;-0,309;-0,066;2

-1076;-0,273;0,08;2

-1099;0,017;-0,062;2

-1116;0,105;0,002;2

-1129;-0,176;-0,084;2

-1145;-0,001;-0,078;2

-1156;0,071;0,104;2

-1164;-0,516;-0,042;2

-1175;-0,559;0,002;2

-1180;-0,517;0,046;2

-1190;-0,497;-0,062;2

-1194;-0,626;0,055;2

-1197;-0,58;0,016;2

-1199;-0,087;-0,035;2

-1207;-0,564;0,078;2

-1212;-0,655;-0,077;2

-1221;-0,88;0,054;2

-1220;-0,759;-0,033;2

-1222;-0,266;-0,018;2

-1218;-0,283;0,079;2

-1214;-0,026;-0,052;2

-1198;0,261;0,02;2

-1174;0,004;-0;2

-1142;0,176;-0,063;2

-1108;-0,263;0,052;2

-1070;-0,363;0,004;2

-1040;-0,181;0,016;2

-998;-0,328;0,049;2

-951;-0,094;-0,128;2

-897;-0,003;0,073;2

-842;-0,102;-0,02;2

-784;0,043;0,014;2

-725;0,219;0,03;2

-662;0,03;-0,068;2

-598;0,065;0,042;2

-540;0,129;0,006;2

-495;0,175;-0,05;2

-478;0,398;0,102;2

-494;0,73;-0,064;2

-543;0,681;0,01;2

-612;0,344;0,005;2

-680;0,66;-0,057;2

-741;0,757;0,089;2

-789;0,492;-0,056;2

-835;0,474;0,06;2

-874;0,411;-0,024;2

-917;0,593;-0,06;2

-950;0,056;0,082;2

-981;-0,408;-0,082;2

-1005;-0,281;-0,008;2

-1031;-0,177;0,032;2

-1053;-0,212;-0,041;2

-1075;-0,257;0,034;2

-1093;-0,024;-0,017;2

-1107;0,209;-0,027;2

-1115;0,09;0,072;2

-1123;0,003;-0,049;2

-1132;0,119;0,053;2

-1142;-0,283;-0,013;2

-1157;-0,308;-0,048;2

-1169;-0,261;0,129;2

-1182;-0,726;-0,068;2

-1184;-0,874;0,025;2

-1187;-0,712;0,014;2

-1186;-0,86;-0,046;2

-1190;-1,079;0,075;2

-1191;-0,484;-0,045;2

-1193;-0,063;0,026;2

-1181;-0,525;0,045;2

-1161;-0,368;-0,059;2

-1139;-0,236;0,056;2

-1112;-0,243;-0,013;2

-1072;-0,237;-0,011;2

-1026;-0,499;0,095;2

-972;-0,683;-0,068;2

-918;-0,508;0,049;2

-867;-0,255;-0,006;2

-812;-0,148;0,009;2

-755;-0,371;0,075;2

-690;-0,106;-0,003;2

-628;-0,42;0,006;2

-573;-0,756;-0,023;2

-533;-0,647;-0,08;2

-519;-0,7;0,099;2

-532;-0,341;-0,061;2

-581;-0,03;0,039;2

-644;-0,047;0,036;2

-714;0,301;-0,061;2

-778;0,172;0,156;2

-833;-0,008;-0,037;2

-876;-0,078;0,03;2

-912;-0,293;0,05;2

-936;-0,117;-0,068;2

-963;0,075;0,047;2

-980;-0,532;-0,044;2

-999;-0,304;-0,027;2

-1012;-0,452;0,095;2

-1030;-0,521;-0,08;2

-1047;-0,472;0,062;2

-1068;-0,744;0,005;2

-1086;-0,513;-0,02;2

-1103;-0,265;0,046;2

-1114;-0,099;-0,101;2

-1128;-0,084;0,017;2

-1139;0,119;-0,001;2

-1148;0,439;-0,039;2

-1156;0,204;0,074;2

-1159;0,367;-0,086;2

-1164;0,429;0,023;2

-1171;0,558;0,028;2

-1178;0,812;-0,078;2

-1186;0,491;0,071;2

-1188;0,386;-0,032;2

-1185;0,627;0,055;2

-1185;0,405;0,111;2

-1186;0,475;-0,095;2

-1196;0,404;0,042;2

-1204;0,56;-0,02;2

-1204;0,523;-0,017;2

-1195;0,4;0,106;2

-1170;0,29;-0,065;2

-1141;0,39;0,057;2

-1102;0,23;0,009;2

-1065;0,079;-0,032;2

-1028;0,016;0,087;2

-992;0,163;-0,075;2

-954;0,383;-0,041;2

-911;0,316;0,034;2

-859;0,331;-0,043;2

-804;0,256;0,102;2

-751;0,127;-0,025;2

-693;0,081;0,031;2

-638;0,136;0,022;2

-581;0,181;-0,089;2

-528;0,196;0,072;2

-494;-0,003;-0,04;2

-492;-0,443;-0,003;2

-520;-0,437;0,076;2

-575;-0,728;-0,072;2

-640;-0,75;0,054;2

-709;-0,615;0,02;2

-770;-0,135;-0,019;2

-832;-0,196;0,086;2

-878;-0,572;0,016;2

-920;-0,522;0,027;2

-946;-0,002;0,018;2

-970;0,38;-0,078;2

-989;0,101;0,087;2

-1012;0,314;-0,097;2

-1032;0,418;0,003;2

-1052;0,317;0,015;2

-1064;0,421;-0,051;2

-1080;0,348;0,071;2

-1095;0,165;-0,038;2

-1109;0,097;-0,007;2

-1128;0,106;0,071;2

-1142;0,339;-0,079;2

-1155;0,328;0,073;2

-1168;0,504;-0,082;2

-1183;0,43;-0,012;2

-1194;0,135;0,063;2

-1198;0,374;-0,087;2

-1200;0,016;0,13;2

-1194;-0,337;0,029;2

-1196;-0,423;-0,07;2

-1199;-0,586;0,091;2

-1202;-0,176;-0,042;2

-1203;0,323;0,047;2

-1206;0,259;0,007;2

-1206;0,435;-0,082;2

-1211;0,24;0,067;2

-1210;0,405;-0,065;2

-1202;0,341;0,049;2

-1177;-0,008;-0,006;2

-1142;-0,038;-0,071;2

-1102;-0,278;0,074;2

-1060;-0,213;-0,021;2

-1019;-0,273;-0,062;2

-975;-0,365;0,041;2

-932;-0,235;-0,075;2

-880;-0,165;0,065;2

-822;-0,071;-0,044;2

-760;-0,048;0,063;2

-701;0,073;0,078;2

-641;0,155;0,047;2

-590;0,002;0,031;2

-544;-0,182;0,022;2

-522;-0,249;-0,041;2

-524;-0,296;0,083;2

-557;-0,133;-0,048;2

-607;-0,199;-0,031;2

-670;-0,383;0,06;2

-728;-0,112;-0,047;2

-786;-0,136;0,09;2

-833;-0,076;-0,116;2

-873;0,161;0,004;2

-910;0,01;0,089;2

-941;-0,144;-0,084;2

-966;-0,227;0,07;2

-986;-0,061;-0,053;2

-1008;0,01;0,018;2

-1026;-0,061;0,068;2

-1051;0,06;-0,109;2

-1072;-0,079;0,065;2

-1092;-0,247;-0;2

-1106;-0,434;0,045;2

-1112;-0,512;0,07;2

-1118;-0,107;0,005;2

-1117;0,194;0,011;2

-1122;-0,355;0,019;2

-1127;-0,799;-0,059;2

-1142;-0,873;0,1;2

-1160;-0,367;-0,055;2

-1177;-0,12;0,001;2

-1191;-1,44;0,025;2

-1200;-1,807;-0,074;2

-1197;-1,86;0,078;2

-1198;-1,898;-0,064;2

-1192;-1,854;-0,047;2

-1190;-1,329;0,043;2

-1180;-0,735;-0,07;2

-1161;-0,598;0,082;2

-1133;-0,799;-0,022;2

-1101;-0,586;-0,019;2

-1064;-0,222;0,066;2

-1032;-0,091;-0,103;2

-993;-0,352;0,066;2

-958;-0,103;-0,031;2

-914;0,542;0,058;2

-874;0,735;0,084;2

-826;1,033;-0,06;2

-770;0,884;0,051;2

-707;0,491;0,003;2

-639;0,673;-0,003;2

-567;0,243;0,076;2

-516;-0,081;-0,057;2

-496;0,107;0,019;2

-515;-0,103;0,022;2

-570;-0,174;-0,092;2

-636;-0,216;0,066;2

-712;-0,277;-0,018;2

-778;-0,091;-0,023;2

-834;-0,072;0,095;2

-877;0,044;-0,106;2

-908;0,194;0,047;2

-933;0,438;-0,073;2

-952;0,175;-0,073;2

-978;-0,49;0,074;2

-998;-0,525;-0,08;2

-1017;-0,268;0,043;2

-1031;-0,087;0,013;2

-1047;0,343;-0,057;2

-1061;0,308;0,085;2

-1080;0,089;-0,066;2

-1094;0,33;0,039;2

-1113;0,304;0,041;2

-1129;0,161;-0,051;2

-1146;0,156;0,069;2

-1159;0,424;-0,046;2

-1166;0,813;0,007;2

-1168;0,813;0,161;2

-1173;0,884;-0,032;2

-1174;0,907;0,036;2

-1177;0,732;0,017;2

-1177;0,637;0,004;2

-1177;0,433;0,067;2

-1173;0,461;-0,104;2

-1170;0,32;0,057;2

-1159;-0,179;0,014;2

-1142;-0,324;0,026;2

-1118;-0,28;0,081;2

-1091;-0,434;-0,074;2

-1059;-0,085;0,026;2

-1023;0,148;-0,005;2

-979;0,048;0;2

-928;0,191;0,092;2

-866;0,459;-0,081;2

-804;0,655;0,022;2

-741;0,913;0,086;2

-682;1,13;-0,072;2

-628;1,077;0,11;2

-572;0,765;-0,033;2

-529;0,646;-0,048;2

-506;0,635;0,039;2

-520;1,038;-0,068;2

-564;0,805;0,045;2

-624;0,724;-0,029;2

-694;0,991;-0,051;2

-760;0,886;0,104;2

-820;0,689;-0,075;2

-863;0,614;0,134;2

-898;0,741;-0;2

-916;0,742;-0,035;2

-934;0,492;0,109;2

-952;0,33;-0,115;2

-971;0,258;0,024;2

-993;0,046;-0,011;2

-1017;0,136;-0,039;2

-1036;-0,154;0,07;2

-1056;-0,306;-0,049;2

-1073;-0,22;0,013;2

-1089;-0,191;0,045;2

-1108;-0,318;0,047;2

-1127;-1,087;0,07;2

-1149;-1,341;-0,024;2

-1158;-1,436;-0,011;2

-1159;-1,6;0,046;2

-1151;-1,294;-0,115;2

-1144;-0,845;0,053;2

-1138;-0,608;-0,013;2

-1143;-0,179;0,029;2

-1151;-0,34;0,088;2

-1167;-0,295;-0,065;2

-1178;0,077;0,048;2

-1191;0,059;-0,029;2

-1189;0,265;-0,055;2

-1176;0,278;0,102;2

-1148;0,507;0,07;2

-1118;0,352;0,025;2

-1086;-0,032;0,064;2

-1052;0,063;-0,046;2

-1014;0,035;0,077;2

-968;-0,022;-0,058;2

-921;-0,365;0,009;2

-869;-0,963;0,037;2

-816;-0,941;-0,101;2

-763;-0,703;0,057;2

-709;-0,928;-0,05;2

-658;-1,411;-0,003;2

-610;-1,852;0,084;2

-571;-1,787;-0,087;2

-543;-0,953;0,065;2

-539;-0,084;-0,068;2

-557;0,334;-0,008;2

-602;0,242;0,088;2

-660;0,498;-0,055;2

-725;0,733;0,034;2

-780;0,862;0,008;2

-830;0,929;-0,043;2

-868;0,794;0,071;2

-898;0,58;-0,022;2

-923;0,162;0,027;2

-946;-0,184;0,01;2

-964;-0,341;-0,092;2

-988;-0,357;0,052;2

-1006;-0,308;-0,022;2

-1029;-0,004;0,022;2

-1052;-0,453;0,03;2

-1075;-0,459;-0,107;2

-1094;-0,218;0,067;2

-1109;-0,441;-0,007;2

-1117;-0,317;-0,008;2

-1123;-0,205;0,069;2

-1123;-0,143;-0,051;2

-1126;-0,228;0,042;2

-1130;-0,197;0,009;2

-1135;0,106;-0,049;2

-1140;-0,101;0,071;2

-1147;-0,16;-0,076;2

-1158;-0,118;0,01;2

-1164;-0,059;0,032;2

-1173;0,256;0,018;2

-1177;0,078;0,094;2

-1182;0,041;-0,026;2

-1180;0,152;-0,013;2

-1168;-0,133;0,052;2

-1144;0,095;-0,088;2

-1105;0,494;0,076;2

-1069;0,547;-0,012;2

-1026;0,42;0,001;2

-989;0,222;0,051;2

-948;0,232;-0,057;2

-906;0,088;0,08;2

-864;0,137;0,007;2

-815;0,416;-0,014;2

-757;-0,086;0,066;2

-695;-0,282;-0,065;2

-627;0,022;0,023;2

-566;0,306;0,024;2

-518;0,628;-0,041;2

-498;0,267;0,043;2

-511;0,504;-0,07;2

-554;0,815;0,081;2

-618;0,371;0,003;2

-684;0,399;-0,147;2

-750;0,753;-0,027;2

-804;1,066;-0,058;2

-851;0,96;0,009;2

-892;0,374;-0,194;2

-929;0,554;-0,055;2

-961;0,643;-0,005;2

-987;0,396;-0,008;2

-1004;0,084;-0,005;2

-1018;-0,054;-0,027;2

-1031;0,036;-0,056;2

-1049;-0,199;0,062;2

-1065;-0,456;0,006;2

-1084;-0,724;-0,04;2

-1095;-1,165;0,116;2

-1110;-1,415;-0,049;2

-1124;-1,113;0,103;2

-1145;-1,078;0,039;2

-1163;-1,034;-0,065;2

-1175;-0,981;0,069;2

-1182;-0,581;0,066;2

-1185;-0,609;0,046;2

-1193;-0,484;0,128;2

-1197;-0,378;-0,088;2

-1204;-0,295;0,088;2

-1206;-0,075;-0,073;2

-1203;-0,116;-0,031;2

-1190;-0,077;0,046;2

-1174;-0,001;-0,054;2

-1152;0,083;0,083;2

-1130;0,146;-0,01;2

-1101;0,021;-0,022;2

-1071;-0,122;0,073;2

-1033;-0,2;-0,056;2

-993;-0,193;-0,02;2

-950;-0,286;0,009;2

-904;0,013;-0,03;2

-850;0,014;0,098;2

-790;0,066;-0,084;2

-729;0,24;0,009;2

-676;0,229;0,01;2

-624;0,336;-0,05;2

-582;0,003;0,093;2

-557;-0,306;-0,082;2

-562;0,096;-0;2

-599;-0,223;0,03;2

-658;0,04;-0,091;2

-729;0,512;0,158;2

-794;0,489;-0,036;2

-853;0,332;0,057;2

-898;0,705;0,029;2

-934;1,338;-0,092;2

-957;0,734;0,056;2

-980;0,488;0,021;2

-996;0,58;-0,02;2

-1015;0,372;0,091;2

-1035;0,702;-0,079;2

-1054;0,49;0,043;2

-1074;0,247;0,03;2

-1097;0,17;-0,027;2

-1118;0,273;0,15;2

-1139;0,454;-0,071;2

-1154;0,091;0,056;2

-1162;-0,271;0,016;2

-1168;-0,275;-0,093;2

-1166;-0,223;0,173;2

-1165;-0,021;-0,041;2

-1158;0,046;0,041;2

-1159;-0,072;0,027;2

-1164;0,382;-0,091;2

-1171;0,405;0,099;2

-1180;0,081;-0,013;2

-1190;0,162;-0,035;2

-1196;-0,207;0,064;2

-1199;-0,481;-0,067;2

-1200;-0,708;0,043;2

-1200;-0,873;0,01;2

-1194;-0,842;-0,036;2

-1179;-0,812;0,048;2

-1155;-0,3;-0,072;2

-1121;0,11;0,043;2

-1090;-0,064;0,024;2

-1055;0,378;-0,017;2

-1019;0,124;0,091;2

-977;0,161;-0,059;2

-926;0,414;0,016;2

-867;0,267;0,027;2

-811;0,354;0,017;2

-758;0,099;0,092;2

-712;0,248;-0,051;2

-665;0,198;0,003;2

-619;-0,363;0,061;2

-584;-0,565;-0,055;2

-580;-0,91;0,097;2

-602;-0,985;-0,013;2

-651;-0,7;-0,021;2

-707;-0,587;0,091;2

-768;-0,28;-0,06;2

-819;-0,106;0,04;2

-872;-0,172;0,007;2

-917;-0,187;-0,144;2

-952;-0,303;0,069;2

-986;-0,106;-0,04;2

-1010;0,208;0,015;2

-1034;0,254;0,019;2

-1053;0,208;-0,069;2

-1070;-0,152;0,065;2

-1087;-0,062;-0,091;2

-1101;0,086;-0,034;2

-1114;-0,17;0,052;2

-1127;0,072;-0,091;2

-1137;-0,068;0,065;2

-1145;-0,232;-0,032;2

-1150;-0,169;0,013;2

-1160;-0,24;0,074;2

-1176;-0,285;-0,047;2

-1190;-0,363;0,09;2

-1207;-0,381;-0,01;2

-1211;-0,336;-0,045;2

-1218;-0,337;0,064;2

-1217;-0,139;-0,038;2

-1216;-0,154;0,031;2

-1214;-0,227;-0,016;2

-1214;0,017;-0,054;2

-1211;0,322;0,073;2

-1211;0,195;-0,076;2

-1199;-0,092;0,021;2

-1174;-0,182;0,01;2

-1138;0,144;-0,077;2

-1094;0,178;0,038;2

-1049;-0,036;-0,023;2

-1006;0,142;-0,001;2

-960;0,348;0,061;2

-914;0,276;-0,077;2

-864;0,225;-0,021;2

-809;-0,194;-0,063;2

-751;-0,123;0,06;2

-690;0,037;0,063;2

-633;-0,061;-0,07;2

-580;-0,745;0,057;2

-549;-1,389;0,007;2

-546;-1,109;0,029;2

-576;-1,103;0,083;2

-629;-1,142;0,029;2

-691;-1,164;0,027;2

-753;-0,739;-0,008;2

-805;-0,177;-0,071;2

-848;0,12;0,08;2

-883;0,185;-0,067;2

-915;0,313;-0,018;2

-939;0,4;0,038;2

-961;0,364;-0,081;2

-980;0,052;0,096;2

-1000;0,053;-0,057;2

-1021;0,099;-0,11;2

-1044;-0,124;0,054;2

-1063;0,242;-0,074;2

-1079;0,688;0,071;2

-1091;0,752;-0,019;2

-1104;0,811;-0,039;2

-1118;0,685;0,067;2

-1130;0,831;-0,06;2

-1141;0,851;0,11;2

-1144;0,35;-0,005;2

-1153;0,387;-0,048;2

-1162;0,187;0,097;2

-1172;0,125;-0,034;2

-1175;-0,146;0,039;2

-1176;-0,484;0,039;2

-1175;-0,053;-0,065;2

-1181;-0,275;0,104;2

-1182;-0,64;-0,072;2

-1190;-0,634;0,056;2

-1188;-0,817;0,035;2

-1179;-0,311;-0,137;2

-1160;-0,239;0,066;2

-1133;-0,384;0,021;2

-1099;0,292;-0,025;2

-1062;0,597;0,056;2

-1023;0,692;-0,056;2

-977;0,19;0,046;2

-935;0,451;0,011;2

-888;0,12;-0,021;2

-841;0,027;0,082;2

-789;0,22;-0,061;2

-740;0,058;0,078;2

-688;-0,064;0,022;2

-641;0,127;-0,029;2

-604;-0,027;0,065;2

-579;-0,237;-0,082;2

-586;-0,112;-0,001;2

-612;0,006;-0,001;2

-660;-0,214;-0,046;2

-713;-0,876;0,074;2

-767;-0,77;-0,041;2

-814;-0,65;-0,025;2

-862;-0,924;0,046;2

-904;-0,397;-0,063;2

-939;-0,148;0,068;2

-970;-0,176;0;2

-996;-0,022;-0,039;2

-1021;-0,112;0,066;2

-1041;-0,028;-0,044;2

-1060;0,109;0,03;2

-1074;0,08;0,106;2

-1090;0,072;-0,038;2

-1108;-0,107;0,098;2

-1127;-0,164;-0,055;2

-1145;-0,163;0,029;2

-1156;-0,486;0,041;2

-1156;-0,654;-0,082;2

-1157;-0,698;0,101;2

-1154;-0,236;-0,007;2

-1162;-0,085;-0,059;2

-1168;0,035;0,018;2

-1179;0,394;-0,019;2

-1187;0,18;0,07;2

-1194;0,172;0,008;2

-1197;0,498;-0,024;2

-1203;0,284;0,121;2

-1200;-0,051;-0,068;2

-1194;-0,293;0,059;2

-1181;0,036;-0,011;2

-1159;-0,056;-0,036;2

-1138;-0,898;0,13;2

-1114;-0,574;-0,086;2

-1087;-0,379;0,056;2

-1052;-0,699;0,024;2

-1013;-0,434;-0,037;2

-969;-0,158;0,108;2

-920;-0,023;-0,078;2

-868;0,631;0,067;2

-810;0,678;0,023;2

-750;0,858;-0,067;2

-690;0,775;0,106;2

-644;0,576;-0,052;2

-603;0,534;0,032;2

-584;-0,058;0,047;2

-581;-0,267;-0,085;2

-610;-0,331;0,07;2

-655;-0,611;-0,034;2

-711;-0,441;0,017;2

-769;-0,081;0,052;2

-825;0,125;-0,078;2

-874;0,377;0,054;2

-914;0,469;-0,037;2

-946;0,451;-0,029;2

-969;0,207;0,061;2

-988;0,317;-0,055;2

-1006;0,417;0,023;2

-1027;-0,001;-0,013;2

-1044;0,201;-0,063;2

-1066;0,267;0,067;2

-1082;0,334;-0,05;2

-1095;0,419;0,011;2

-1103;0,347;0,013;2

-1109;0,088;-0,14;2

-1111;-0,514;0,057;2

-1122;-0,797;-0,029;2

-1131;-0,609;-0,007;2

-1147;-0,512;0,053;2

-1155;-0,375;-0,068;2

-1156;-0,167;0,065;2

-1162;-0,194;-0,001;2

-1166;-0,296;-0,01;2

-1177;-0,254;0,049;2

-1182;-0,141;-0,06;2

-1183;-0,123;0,072;2

-1173;-0,277;-0,016;2

-1150;0,113;-0,062;2

-1121;-0,181;0,099;2

-1085;-0,349;-0,051;2

-1046;0,345;0,007;2

-1003;0,86;0,079;2

-960;0,803;-0,101;2

-914;0,542;0,016;2

-867;0,783;-0,059;2

-806;0,963;0,014;2

-749;0,791;0,036;2

-690;0,495;-0,116;2

-639;0,316;0,063;2

-599;0,331;-0,041;2

-576;0,035;-0,036;2

-574;-0,411;0,033;2

-600;-0,25;-0,081;2

-643;-0,384;0,05;2

-697;-0,39;-0,002;2

-751;0,353;0,001;2

-802;0,462;0,082;2

-847;0,82;-0,056;2

-888;0,764;0,036;2

-922;0,242;0,01;2

-952;0,187;-0,046;2

-976;0,077;0,075;2

-993;-0,109;-0,116;2

-1012;-0,223;0,007;2

-1031;-0,669;0,006;2

-1053;-0,658;-0,09;2

-1069;-0,364;-0,002;2

-1085;-0,383;-0,038;2

-1093;-0,243;-0,014;2

-1100;0,049;0,083;2

-1102;0,158;-0,07;2

-1111;0,085;0,083;2

-1118;0,161;-0,019;2

-1129;0,034;0,032;2

-1141;-0,2;0,066;2

-1151;0,153;-0,044;2

-1159;0,215;0,027;2

-1166;-0,167;-0,004;2

-1172;0,24;-0,03;2

-1176;0,122;0,077;2

-1183;-0,087;-0,091;2

-1182;0,026;0,049;2

-1186;0,202;0,023;2

-1175;0,256;-0,056;2

-1153;0,252;0,083;2

-1125;0,242;-0,051;2

-1096;0,175;0,037;2

-1070;0,089;-0,071;2

-1044;0,438;-0,065;2

-1011;0,314;0,085;2

-974;0,186;-0,03;2

-930;0,287;0,009;2

-879;-0,065;0,084;2

-827;-0,574;-0,092;2

-769;-1,147;0,074;2

-712;-1,513;0,023;2

-654;-1,146;-0,026;2

-607;-1,204;0,048;2

-574;-1,097;-0,085;2

-566;-0,897;0,041;2

-581;-0,844;-0,004;2

-620;-0,556;-0,04;2

-670;-0,497;0,076;2

-728;-0,393;-0,065;2

-784;-0,545;0,116;2

-840;-0,773;0,007;2

-884;-0,671;-0,127;2

-917;-0,607;0,059;2

-941;-0,542;-0,036;2

-962;-0,295;-0,043;2

-978;-0,193;0,024;2

-994;-0,051;-0,075;2

-1007;-0,21;0,094;2

-1028;-0,246;-0,026;2

-1046;-0,494;-0,04;2

-1066;-0,657;0,054;2

-1085;-0,668;-0,051;2

-1098;-0,744;0,066;2

-1111;-0,726;0,017;2

-1119;-0,521;-0,03;2

-1130;-0,578;0,072;2

-1140;-0,51;-0,1;2

-1150;-0,369;0,04;2

-1160;0,106;0,025;2

-1166;0,6;0,045;2

-1168;0,452;0,057;2

-1161;0,466;-0,062;2

-1150;0,493;0,004;2

-1132;0,314;0,011;2

-1113;0,464;-0,088;2

-1087;0,303;0,018;2

-1058;-0,191;0,082;2

-1019;-0,604;0,051;2

-981;-1,113;-0,149;2

-942;-0,714;-0,144;2

-907;-0,472;0,062;2

-867;-0,48;-0,007;2

-823;-0,364;0,029;2

-766;-0,467;0,115;2

-708;-0,161;-0,083;2

-647;0,003;0,082;2

-601;-0,009;-0,027;2

-571;0,168;-0,038;2

-571;0,163;0,12;2

-602;0,294;-0,07;2

-657;-0,045;0,047;2

-724;-0,503;0,038;2

-792;-0,423;-0,069;2

-842;-0,228;0,027;2

-879;-0,355;-0,056;2

-911;-0,413;0,069;2

-940;-0,745;0,016;2

-967;-0,482;-0,054;2

-988;-0,411;0,078;2

-1004;-0,355;-0,036;2

-1012;-0,074;0,009;2

-1028;-0,287;0,084;2

-1046;-0,463;-0,028;2

-1069;-0,126;0,066;2

-1087;-0,219;0,04;2

-1096;-0,25;-0,115;2

-1108;-0,087;0,065;2

-1119;0,207;-0,071;2

-1136;0,326;0,028;2

-1143;0,017;-0,004;2

-1150;0,123;-0,094;2

-1151;0,073;0,042;2

-1156;0,09;-0,079;2

-1158;0,296;0,026;2

-1166;0,442;0,014;2

-1169;0,565;-0,061;2

-1169;0,251;0,058;2

-1165;0,408;-0,061;2

-1168;0,24;-0,024;2

-1180;-0,087;-0,009;2

-1198;0,051;-0,057;2

-1207;0,221;0,086;2

-1198;0,158;-0,025;2

-1166;0,122;-0,02;2

-1127;0,198;0,074;2

-1093;0,691;-0,073;2

-1064;0,83;0,048;2

-1041;0,58;0,014;2

-1009;0,719;0,014;2

-966;0,785;0,06;2

-922;0,749;-0,056;2

-877;0,966;0,023;2

-826;0,799;-0,016;2

-770;0,55;-0,046;2

-710;0,559;0,219;2

-650;0,768;-0,043;2

-593;0,524;0,026;2

-550;-0,103;-0,009;2

-531;-0,166;-0,06;2

-545;0,271;0,076;2

-580;0,498;-0,016;2

-638;0,635;0,002;2

-702;0,111;0,004;2

-766;-0,044;-0,08;2

-822;-0,13;0,073;2

-858;-0,284;-0,02;2

-890;-0,135;-0,034;2

-910;-0,405;0,072;2

-932;-0,113;-0,059;2

-953;0,305;0,018;2

-974;0,298;0,034;2

-995;0,438;-0,013;2

-1017;-0,079;0,101;2

-1036;-0,225;-0,079;2

-1055;0,148;0,017;2

-1072;0,199;0,016;2

-1084;0,491;-0,07;2

-1095;0,64;0,121;2

-1101;0,588;-0,036;2

-1109;0,198;-0,013;2

-1119;-0,434;0,032;2

-1135;-0,237;-0,087;2

-1146;-0,161;0,049;2

-1155;-0,279;-0,034;2

-1154;-0,218;-0,028;2

-1154;-0,275;0,088;2

-1154;0,313;-0,094;2

-1160;0,362;0,07;2

-1163;0,508;-0,008;2

-1167;0,877;-0,026;2

-1160;0,614;0,204;2

-1156;0,526;-0,044;2

-1155;0,659;0,046;2

-1162;0,428;0,16;2

-1174;0,42;-0,068;2

-1181;-0,1;0,091;2

-1161;-0,26;-0,079;2

-1130;-0,142;0,005;2

-1090;-0,156;0,04;2

-1048;-0,233;-0,035;2

-1007;-0,3;0,093;2

-963;-0,431;-0,086;2

-918;-0,282;-0,026;2

-873;-0,328;0,061;2

-833;-0,491;-0,025;2

-787;-0,231;0,058;2

-737;0,068;-0,02;2

-684;0,345;-0,011;2

-634;0,291;0,047;2

-603;0,33;-0,083;2

-594;0,824;0,079;2

-614;0,82;-0,005;2

-650;0,435;-0,058;2

-696;0,124;0,059;2

-740;0,286;-0,02;2

-788;0,483;0,036;2

-828;0,545;-0,067;2

-870;0,657;-0,069;2

-910;0,628;0,088;2

-942;0,772;-0,059;2

-966;0,739;0,038;2

-983;0,242;0,042;2

-995;0,468;-0,066;2

-1005;0,286;0,078;2

-1018;0,28;-0,007;2

-1031;0,651;-0,004;2

-1048;0,332;0,061;2

-1060;0,113;-0,164;2

-1077;0,022;0,06;2

-1088;0,255;0,039;2

-1103;0,233;0,007;2

-1113;0,167;0,074;2

-1121;0,444;-0,075;2

-1125;0,356;0,054;2

-1131;0,192;0,044;2

-1133;0,175;-0,052;2

-1136;0,143;0,087;2

-1136;-0,07;-0,06;2

-1136;0,088;0,01;2

-1132;-0,011;0,033;2

-1122;0,036;-0,064;2

-1106;0,123;0,092;2

-1080;0,232;0,055;2

-1051;0,164;0,023;2

-1016;0,092;0,055;2

-975;0,123;-0,079;2

-928;0,127;0,082;2

-874;0,082;-0,005;2

-825;0,112;-0,01;2

-773;0,318;0,075;2

-727;-0,019;-0,05;2

-684;-0,328;0,072;2

-640;-0,517;0,006;2

-596;-0,033;-0,051;2

-570;-0,199;0,083;2

-576;0,014;-0,097;2

-611;0,179;0,01;2

-671;-0,176;-0,101;2

-735;-0,459;-0,048;2

-797;-0,414;0,08;2

-848;-0,135;-0,063;2

-886;-0,136;0,013;2

-913;-0,123;0,081;2

-935;0,26;-0,057;2

-952;0,301;0,078;2

-971;0,359;-0,058;2

-986;0,393;-0,012;2

-1005;0,415;0,096;2

-1020;0,352;-0,141;2

-1038;0,149;0,083;2

-1057;0,156;0,015;2

-1076;0,298;-0,012;2

-1094;0,016;0,016;2

-1105;-0,166;-0,078;2

-1113;-0,083;0,042;2

-1119;-0,189;0,012;2

-1129;-0,46;-0,047;2

-1136;-1,038;0,093;2

-1142;-0,803;-0,064;2

-1145;-0,136;0,031;2

-1155;0,132;0,003;2

-1159;0,432;-0,076;2

-1163;0,646;0,067;2

-1156;0,549;-0,079;2

-1130;0,634;0,008;2

-1098;0,691;0,041;2

-1059;0,314;-0,034;2

-1021;-0,167;0,09;2

-978;0,062;-0,045;2

-930;0,009;-0,008;2

-878;-0,204;0,052;2

-820;-0,281;-0,076;2

-765;-0,315;0,074;2

-711;-0,054;-0,027;2

-659;0,38;-0,013;2

-611;0,629;0,057;2

-572;0,827;-0,072;2

-545;0,808;0,079;2

-553;0,776;0,036;2

-584;1,003;-0,107;2

-635;0,865;0,083;2

-689;0,472;-0,07;2

-739;0,175;0,057;2

-784;-0,015;-0,01;2

-834;0,057;-0,066;2

-880;-0,278;0,058;2

-922;-0,174;0,046;2

-951;-0,129;-0,008;2

-974;-0,318;0,051;2

-987;-0,297;-0,173;2

-1000;-0,289;0,113;2

-1012;-0,46;-0,034;2

-1031;-0,777;-0,022;2

-1052;-1,495;0,077;2

-1077;-1,457;-0,083;2

-1099;-1,119;0,083;2

-1124;-0,777;-0,008;2

-1137;-0,213;-0,019;2

-1142;-0,286;0,08;2

-1149;-0,098;-0,091;2

-1154;0,466;0,061;2

-1161;0,806;0,008;2

-1163;0,731;-0,039;2

-1165;0,171;0,092;2

-1165;-0,08;-0,013;2

-1170;-0,068;0,005;2

-1176;-0,314;0,026;2

-1186;-0,4;-0,077;2

-1192;-0,493;0,069;2

-1193;-0,647;-0,005;2

-1187;-0,51;-0,003;2

-1165;-0,561;0,033;2

-1134;-0,618;-0,097;2

-1096;-0,318;0,126;2

-1055;0,083;-0,118;2

-1005;0,226;0,011;2

-960;0,024;0,072;2

-912;0,236;-0,057;2

-866;0,201;0,048;2

-812;0,585;0,037;2

-760;0,757;-0,045;2

-710;0,474;0,102;2

-669;0,228;-0,126;2

-634;-0,016;0,025;2

-612;-0,65;0,1;2

-607;-0,351;-0,021;2

-626;0,099;0,098;2

-664;0,657;-0,016;2

-711;0,843;0,005;2

-762;0,513;0,028;2

-806;0,718;-0,101;2

-842;0,328;0,053;2

-879;0,271;-0,034;2

-912;0,436;-0,056;2

-942;0,037;0,078;2

-967;-0,081;-0,06;2

-994;-0,082;0,073;2

-1020;-0,207;-0,059;2

-1050;0,043;0,028;2

-1080;0,168;0,085;2

-1102;0,22;-0,077;2

-1113;0,006;0,031;2

-1115;-0,317;0,005;2

-1114;-0,295;-0,047;2

-1108;-0,71;0,1;2

-1111;-0,602;-0,083;2

-1108;-0,334;0,053;2

-1113;-0,514;0,027;2

-1123;-0,063;-0,022;2

-1141;0,132;0,054;2

-1162;-0,039;-0,078;2

-1172;0,274;-0,001;2

-1173;0,493;0,061;2

-1160;0,502;-0,048;2

-1146;0,334;0,065;2

-1123;0,458;-0,012;2

-1103;0,249;-0,009;2

-1077;0,267;0,054;2

-1047;0,236;-0,085;2

-1003;-0,298;0,076;2

-956;0,066;0,031;2

-902;0,236;-0,036;2

-847;-0,315;0,066;2

-790;-0,509;-0,082;2

-733;-0,233;0,032;2

-679;-0,277;-0,008;2

-627;0,238;-0,008;2

-580;0,558;0,089;2

-547;0,863;-0,053;2

-542;0,578;0,015;2

-566;0,161;0,018;2

-619;0,39;-0,06;2

-684;0,346;0,093;2

-748;0,511;-0,029;2

-803;0,694;-0,015;2

-849;0,845;0,088;2

-880;1,182;-0,081;2

-905;0,925;0,043;2

-923;0,601;-0,011;2

-947;0,741;-0,009;2

-970;0,386;0,09;2

-999;-0,015;-0,091;2

-1025;-0,028;0,052;2

-1049;-0,031;0,01;2

-1066;-0,081;-0,075;2

-1078;-0,264;0,101;2

-1083;-0,133;-0,047;2

-1087;0,187;0,019;2

-1094;0,169;0,129;2

-1105;0,014;-0,056;2

-1123;0,05;0,088;2

-1137;0,154;-0,075;2

-1148;-0,01;-0,016;2

-1158;-0,729;0,029;2

-1162;-0,971;-0,078;2

-1165;-0,607;0,08;2

-1166;-0,555;-0,011;2

-1169;-0,009;-0,02;2

-1165;0,34;0,089;2

-1156;0,363;-0,102;2

-1138;0,221;0,072;2

-1110;-0,093;-0,005;2

-1078;0,025;-0,041;2

-1044;0,099;0,076;2

-1005;0,31;-0,063;2

-968;0,551;0,033;2

-921;-0,187;0,005;2

-878;-0,242;-0,051;2

-829;0,216;0,095;2

-786;0,078;-0,056;2

-732;0,076;0,021;2

-680;0,183;0,07;2

-630;0,419;-0,098;2

-603;0,469;0,041;2

-601;0,232;-0,048;2

-631;-0,104;-0,006;2

-679;-0,267;0,021;2

-735;-0,149;-0,098;2

-784;-0,236;0,07;2

-834;-0,229;-0,029;2

-875;0,068;-0,017;2

-912;0,069;0,07;2

-939;0,052;-0,078;2

-964;0,12;0,018;2

-980;0,211;-0,016;2

-1001;0,576;-0,014;2

-1027;0,544;0,072;2

-1050;0,49;-0,098;2

-1072;0,11;0,021;2

-1082;0,049;0,026;2

-1090;0,335;-0,015;2

-1100;0,18;0,109;2

-1110;0,699;-0,071;2

-1126;0,722;-0,004;2

-1140;0,705;0,025;2

-1150;0,875;-0,076;2

-1153;0,649;0,071;2

-1162;0,557;-0,034;2

-1166;0,808;-0,034;2

-1168;0,948;0,066;2

-1164;1,092;-0,117;2

-1164;0,986;0,033;2

-1167;0,627;-0,044;2

-1177;0,704;-0,039;2

-1166;0,654;0,091;2

-1143;0,498;-0,049;2

-1107;0,43;0,057;2

-1066;0,482;-0,013;2

-1021;0,792;-0,068;2

-981;0,482;0,094;2

-935;0,21;-0,054;2

-889;0,162;0,044;2

-844;-0,15;0,034;2

-798;-0,722;-0,014;2

-749;-1,014;0,049;2

-701;-0,801;-0,032;2

-652;-0,88;0,027;2

-607;-1,198;0,044;2

-580;-0,887;-0,067;2

-590;-0,408;0,042;2

-630;-0,19;-0,03;2

-690;-0,229;-0,03;2

-759;-0,227;0,064;2

-818;0,047;-0,121;2

-864;0,293;0,065;2

-898;0,419;0,014;2

-926;0,599;-0,01;2

-946;0,264;0,087;2

-964;0,043;-0,1;2

-979;-0,264;0,03;2

-998;-0,359;0,02;2

-1017;-0,011;-0,073;2

-1040;-0,303;0,077;2

-1059;-0,105;-0,113;2

-1072;0,32;-0,005;2

-1088;0,433;0,024;2

-1097;0,467;-0,085;2

-1111;0,206;0,088;2

-1124;-0,111;-0,028;2

-1137;-0,186;-0,015;2

-1148;-1,015;0,01;2

-1159;-1,073;-0,095;2

-1162;-0,679;0,073;2

-1162;-0,651;-0,012;2

-1159;-0,363;-0,008;2

-1163;-0,607;0,118;2

-1167;-0,745;-0,072;2

-1175;-0,481;0,064;2

-1181;-0,385;0,022;2

-1175;-0,129;-0,04;2

-1162;-0,138;0,095;2

-1135;-0,388;-0,076;2

-1109;-0,188;0,016;2

-1081;-0,19;-0,025;2

-1045;-0,172;-0,044;2

-1006;-0,042;0,09;2

-963;0,021;-0,021;2

-911;-0,003;-0,005;2

-864;-0,031;0,043;2

-811;0,16;-0,076;2

-764;-0,147;0,057;2

-717;0,183;-0,018;2

-677;0,46;0,011;2

-638;0,01;0,077;2

-611;-0,111;-0,079;2

-598;-0,087;0,018;2

-616;-0,429;-0,02;2

-649;-0,411;-0,025;2

-699;-0,885;0,118;2

-755;-0,702;-0,066;2

-811;-0,341;0,046;2

-866;-0,32;0,004;2

-906;-0,071;-0,03;2

-942;0,037;0,076;2

-964;0,287;-0,052;2

-978;0,676;0,026;2

-990;0,461;0,024;2

-1001;0,571;-0,129;2

-1018;0,565;0,045;2

-1040;0,201;-0,06;2

-1062;0,469;0,004;2

-1083;0,513;0,058;2

-1099;0,319;-0,075;2

-1105;0,427;0,094;2

-1114;0,492;-0,02;2

-1116;0,758;-0,015;2

-1120;0,642;0,05;2

-1121;0,844;-0,079;2

-1128;0,703;0,036;2

-1129;0,563;-0,032;2

-1136;0,848;-0,044;2

-1144;0,662;0,075;2

-1154;0,104;-0,1;2

-1160;0,038;0,06;2

-1167;-0,118;0,008;2

-1167;0,235;-0,11;2

-1159;-0,011;0,081;2

-1140;-0,243;-0,048;2

-1113;-0,173;-0,019;2

-1081;-0,263;0,015;2

-1052;-0,014;-0,084;2

-1020;-0,012;0,076;2

-994;0,005;-0,055;2

-963;-0,055;-0,025;2

-933;-0,029;0,078;2

-892;0,115;-0,154;2

-847;-0,33;0,055;2

-792;-0,538;-0,001;2

-742;-0,165;-0,015;2

-693;-0,325;0,082;2

-651;-0,159;-0,039;2

-625;-0,093;0,052;2

-620;-0,016;0;2

-642;-0,089;-0,062;2

-681;-0,357;0,084;2

-737;-0,074;-0,07;2

-791;0,121;-0,005;2

-842;0,133;0,033;2

-885;0,108;0,001;2

-917;-0,112;0,078;2

-946;-0,434;-0,041;2

-969;-0,423;0,023;2

-995;-0,377;0,048;2

-1015;-0,263;-0,077;2

-1035;-0,131;0,072;2

-1047;0,16;-0,046;2

-1060;0,362;0,005;2

-1074;0,396;0,048;2

-1088;0,373;-0,069;2

-1101;0,372;0,084;2

-1113;0,51;0,012;2

-1121;0,549;-0,015;2

-1132;0,315;0,078;2

-1148;0,226;-0,099;2

-1160;0,189;0,035;2

-1175;0,447;0,019;2

-1182;0,707;0,002;2

-1184;0,746;0,105;2

-1181;0,559;-0,032;2

-1181;0,573;-0,005;2

-1176;0,207;0,055;2

-1171;-0,05;-0,083;2

-1151;-0,056;0,078;2

-1127;-0,371;0,01;2

-1093;-0,766;-0,009;2

-1064;-1,244;0,021;2

-1033;-1,066;-0,079;2

-999;-0,663;0,062;2

-951;-0,169;0,01;2

-894;-0,013;-0,028;2

-831;0,151;0,071;2

-769;0,778;-0,065;2

-712;0,711;0,044;2

-659;0,233;0,008;2

-623;0,16;-0,05;2

-607;0,08;0,063;2

-626;-0,126;-0,063;2

-669;-0,147;0,045;2

-720;-0,418;-0,004;2

-776;-0,372;-0,044;2

-816;-0,342;0,063;2

-851;-0,53;-0,046;2

-883;-0,51;-0,01;2

-914;-0,756;-0,006;2

-943;-0,608;-0,071;2

-970;-0,525;0,067;2

-992;-0,573;-0,109;2

-1012;-0,222;0,005;2

-1030;-0,386;0,083;2

-1046;-0,285;-0,101;2

-1059;0,067;0,065;2

-1070;0,067;-0,022;2

-1080;0,026;-0,023;2

-1092;-0,156;0,09;2

-1108;-0,294;-0,071;2

-1113;-0,515;0,045;2

-1123;-0,339;0,008;2

-1126;-0,314;-0,022;2

-1132;-0,687;0,085;2

-1136;-0,725;-0,06;2

-1148;-0,685;0,04;2

-1154;-0,89;0,024;2

-1159;-0,506;-0,09;2

-1147;-0,481;0,085;2

-1122;-0,373;-0,044;2

-1086;-0,023;0,016;2

-1045;-0,036;0,049;2

-1000;0,202;-0,086;2

-957;0,343;0,075;2

-911;0,343;0,007;2

-865;0,62;-0,005;2

-817;0,199;0,078;2

-766;-0,094;-0,087;2

-714;-0,471;0,053;2

-665;-0,705;-0,007;2

-624;-0,605;-0,031;2

-603;-0,203;0,121;2

-612;0,08;-0,056;2

-650;0,171;0,014;2

-697;0,386;0,012;2

-749;0,429;-0,041;2

-792;-0,034;0,082;2

-828;-0,024;-0,048;2

-862;-0,214;0,022;2

-897;-0,367;-0,001;2

-930;-0,124;-0,052;2

-961;-0,332;0,082;2

-985;-0,151;-0,018;2

-1001;0,274;-0,012;2

-1017;-0,136;0,191;2

-1032;0,277;-0,067;2

-1044;0,146;0,089;2

-1054;0,173;-0,018;2

-1065;0,534;-0,009;2

-1076;0,662;0,066;2

-1093;0,725;-0,044;2

-1103;0,849;0,045;2

-1115;0,755;0,008;2

-1120;0,57;-0,069;2

-1126;0,093;0,089;2

-1130;0,189;-0,067;2

-1141;0,094;0,032;2

-1149;0,341;-0,072;2

-1159;0,442;-0,08;2

-1161;0,398;0,091;2

-1160;0,069;-0,037;2

-1159;-0,315;0,004;2

-1146;-0,67;0,056;2

-1119;-0,885;-0,045;2

-1088;-0,742;0,038;2

-1050;-0,251;-0,032;2

-1013;0,276;-0,007;2

-983;0,066;0,062;2

-951;-0,045;-0,082;2

-915;-0,162;0,061;2

-872;-0,373;0,07;2

-822;-0,22;-0,038;2

-764;-0,205;0,098;2

-705;-0,318;-0,078;2

-651;-0,566;0,066;2

-606;-0,783;0;2

-588;-0,114;-0,046;2

-599;-0,409;0,105;2

-642;-0,54;-0,089;2

-701;-0,532;0,011;2

-764;-0,757;0,025;2

-816;-0,399;-0,066;2

-859;-0,561;0,092;2

-892;-0,556;-0,05;2

-917;-0,337;0,002;2

-940;-0,37;0,062;2

-958;-0,25;-0,058;2

-979;0,072;0,066;2

-995;0,106;-0,027;2

-1015;-0,155;0,002;2

-1033;-0,315;0,052;2

-1050;0,105;-0,097;2

-1062;0,25;0,012;2

-1072;0,279;0,02;2

-1080;0,426;-0,036;2

-1091;0,071;0,096;2

-1098;-0,181;-0,04;2

-1108;-0,087;0,036;2

-1113;0,134;0,01;2

-1119;0,135;-0,075;2

-1120;0,039;0,1;2

-1127;0,179;-0,055;2

-1130;0,307;0,006;2

-1135;0,334;0,029;2

-1134;0,535;-0,078;2

-1135;0,547;0,061;2

-1140;0,519;-0,021;2

-1138;0,349;-0,02;2

-1137;0,091;0,085;2

-1121;0,172;-0,069;2

-1096;0,267;0,076;2

-1065;-0,061;-0,032;2

-1029;-0,158;-0,025;2

-988;0,179;0,076;2

-943;0,394;-0,065;2

-897;0,072;-0,093;2

-850;-0,231;-0,018;2

-805;0,195;-0,042;2

-760;-0,227;0,097;2

-712;-0,723;-0,034;2

-668;-1;-0,001;2

-633;-0,958;0,035;2

-616;-0,46;-0,075;2

-624;-0,466;0,099;2

-656;-0,302;-0,021;2

-696;0,204;0,011;2

-749;0,158;0,038;2

-798;0,145;-0,059;2

-846;0,287;0,076;2

-885;0,139;-0,032;2

-916;0,183;-0,021;2

-938;0,379;0,07;2

-958;0,652;-0,072;2

-974;0,633;0,057;2

-985;0,518;-0;2

-994;0,659;-0,049;2

-1005;0,538;0,093;2

-1015;0,795;-0,059;2

-1031;0,501;0,028;2

-1053;-0,046;-0,008;2

-1070;0,112;-0,069;2

-1090;0,165;0,084;2

-1106;0,134;-0,055;2

-1119;0,398;0,003;2

-1129;0,305;0,01;2

-1132;0,14;-0,05;2

-1126;-0,143;0,072;2

-1124;0,143;-0,092;2

-1124;-0,234;-0,022;2

-1131;-0,702;0,068;2

-1144;-0,203;-0,084;2

-1150;-0,246;0,085;2

-1140;0,072;-0,001;2

-1115;0,413;-0,017;2

-1083;0,461;0,091;2

-1044;0,267;-0,068;2

-1006;0,057;0,064;2

-971;0,158;-0,001;2

-925;0,011;-0,034;2

-880;-0,291;0,08;2

-829;-0,332;-0,102;2

-779;-0,544;0,043;2

-730;-0,956;0,011;2

-687;-0,834;-0,089;2

-655;-0,837;0,074;2

-644;-0,592;-0,065;2

-656;-0,032;-0;2

-695;-0,045;0,045;2

-739;-0,287;-0,077;2

-789;-0,708;0,07;2

-829;-0,764;-0,053;2

-861;-0,472;-0,003;2

-887;-0,313;0,081;2

-914;0,073;-0,093;2

-941;0,365;0,065;2

-972;0,458;-0,015;2

-998;0,561;-0,024;2

-1023;0,44;0,065;2

-1045;0,147;-0,086;2

-1063;0,114;0,048;2

-1080;-0,141;0,005;2

-1089;-0,022;-0,059;2

-1098;-0,002;0,085;2

-1101;0,019;-0,04;2

-1104;0,198;-0,099;2

-1107;0,08;0,021;2

-1114;0,042;-0,056;2

-1121;-0,345;0,052;2

-1135;-0,585;-0,034;2

-1143;-0,123;0,013;2

-1152;-0,015;0,064;2

-1153;0,293;-0,044;2

-1152;0,693;0,071;2

-1142;0,724;0,073;2

-1114;1,045;0,002;2

-1084;0,971;0,067;2

-1046;1,103;-0,083;2

-1008;0,896;0,083;2

-975;0,607;-0,045;2

-942;0,736;-0,032;2

-905;0,731;0,069;2

-861;0,653;-0,061;2

-808;0,641;0,04;2

-749;0,64;0,025;2

-696;0,395;-0,052;2

-646;0,152;0,107;2

-616;-0,051;-0,058;2

-606;-0,29;-0,017;2

-628;-0,378;0,018;2

-673;-0,003;-0,075;2

-729;0,363;0,103;2

-788;0,569;-0,027;2

-842;0,361;0,01;2

-881;-0,315;0,046;2

-912;-0,403;-0,101;2

-938;-0,551;0,072;2

-963;-0,693;-0,007;2

-981;-0,177;-0,037;2

-1001;-0,209;0,079;2

-1016;-0,459;-0,072;2

-1035;-0,746;0,04;2

-1053;-0,945;-0,019;2

-1069;-0,874;-0,068;2

-1081;-0,633;0,106;2

-1092;-0,334;-0,076;2

-1103;0,13;0,02;2

-1112;0,106;0,034;2

-1122;0,262;-0,089;2

-1128;0,309;0,099;2

-1130;0,045;-0,065;2

-1124;0,239;0,005;2

-1122;0,557;0,055;2

-1126;0,732;-0,108;2

-1137;0,462;0,097;2

-1145;0,046;-0,032;2

-1141;0,421;0,029;2

-1122;0,089;0,037;2

-1087;-0,392;-0,026;2

-1050;-0,719;0,081;2

-1007;-0,632;-0,005;2

-966;-0,253;-0,023;2

-918;-0,266;0,088;2

-870;-0,217;-0,031;2

-814;-0,112;0,013;2

-759;-0,137;0,024;2

-708;0,048;-0,041;2

-659;0,021;0,088;2

-613;0,146;-0,082;2

-582;0,062;0,015;2

-580;0,057;0,025;2

-617;0,473;-0,001;2

-677;0,205;0,063;2

-748;0,331;-0,028;2

-810;0,453;-0,013;2

-855;0,047;-0,002;2

-880;0,251;-0,081;2

-900;0,681;0,077;2

-917;1,074;-0,037;2

-938;0,745;0,017;2

-965;0,222;0,055;2

-995;0,114;-0,1;2

-1024;0,079;0,067;2

-1048;-0,091;0,025;2

-1066;0,031;-0,007;2

-1078;-0,254;0,08;2

-1092;0,384;-0,09;2

-1103;0,48;0,027;2

-1114;0,193;0,038;2

-1124;0,687;-0,062;2

-1132;0,703;0,104;2

-1136;0,444;-0,056;2

-1144;0,54;0,011;2

-1147;0,224;0,049;2

-1154;0,334;-0,082;2

-1155;0,495;0,07;2

-1161;-0,317;-0,033;2

-1168;-1,084;0,111;2

-1174;-1,536;0,009;2

-1176;-1,322;-0,089;2

-1165;-0,762;0,056;2

-1145;-0,243;-0,001;2

-1118;0,011;-0,012;2

-1093;0,084;0,049;2

-1065;0,669;-0,06;2

-1037;0,801;0,038;2

-1003;0,595;-0,002;2

-967;1,028;-0,047;2

-920;1,072;0,089;2

-872;0,574;-0,036;2

-823;0,262;0,089;2

-769;0,258;0,045;2

-720;0,286;-0,085;2

-678;-0,047;0,059;2

-651;-0,251;-0,042;2

-644;0,18;0,01;2

-663;0,244;0,029;2

-699;0,12;-0,032;2

-751;-0,19;0,052;2

-801;-0,735;-0,017;2

-854;-0,472;-0,014;2

-899;-0,595;0,034;2

-939;-0,279;-0,073;2

-968;-0,198;0,156;2

-996;-0,415;0,023;2

-1014;-0,012;0,037;2

-1029;0,351;0,096;2

-1036;0,501;-0,062;2

-1048;0,37;0,046;2

-1060;0,186;0,016;2

-1084;0,391;-0,073;2

-1103;-0,038;0,097;2

-1124;0,13;-0,072;2

-1137;0,392;0,01;2

-1147;0,313;0,032;2

-1152;0,67;-0,04;2

-1155;0,319;0,168;2

-1157;-0,233;-0,02;2

-1161;0,135;-0,019;2

-1171;-0,014;0,053;2

-1183;0,089;-0,082;2

-1196;0,022;0,075;2

-1211;-0,13;-0,015;2

-1207;-0,247;-0,038;2

-1186;-0,534;0,075;2

-1151;-0,465;-0,072;2

-1115;-0,388;0,069;2

-1072;-0,417;0,003;2

-1039;-0,267;-0,036;2

-1006;-0,219;0,146;2

-975;-0,4;-0,035;2

-934;-0,067;0,057;2

-887;-0,293;-0,022;2

-834;-0,264;-0,076;2

-788;-0,047;0,078;2

-755;-0,087;-0,077;2

-755;-0,018;0,024;2

-783;-0,363;0,056;2

-828;0,064;-0,076;2

-874;-0,138;0,095;2

-915;-0,562;-0,036;2

-945;-0,05;-0,011;2

-970;-0,138;0,067;2

-995;-0,076;-0,092;2

-1017;0,107;0,066;2

-1037;0,119;0,01;2

-1057;0,294;-0,033;2

-1080;0,19;0,065;2

-1096;0,288;-0,051;2

-1117;0,511;0,036;2

-1126;0,615;-0,007;2

-1131;0,902;-0,048;2

-1131;0,978;0,091;2

-1136;0,972;-0,068;2

-1147;0,521;0,045;2

-1163;0,498;0,008;2

-1177;0,807;-0,055;2

-1178;0,703;0,107;2

-1161;0,792;0,019;2

-1127;0,452;0,015;2

-1092;0,101;-0,02;2

-1051;0,161;-0,086;2

-1009;0,113;0,074;2

-972;-0,108;-0,016;2

-936;0,213;-0,011;2

-906;0,334;0,078;2

-870;0,016;-0,079;2

-835;-0,114;-0,025;2

-789;-0,389;-0,015;2

-739;-0,24;-0,049;2

-691;0,044;0,052;2

-656;0,084;-0,07;2

-634;0,612;0,027;2

-642;0,27;0,013;2

-673;0,086;-0,06;2

-729;-0,023;0,085;2

-789;-0,209;-0,069;2

-849;-0,437;0,015;2

-894;-0,304;0,042;2

-930;-0,466;-0,083;2

-957;-0,717;0,055;2

-985;-0,616;-0,052;2

-1004;-0,475;-0,004;2

-1027;-0,481;0,045;2

-1044;-0,083;-0,113;2

-1057;-0,094;0,069;2

-1069;-0,235;-0,014;2

-1081;-0,003;0,019;2

-1092;-0,158;0,078;2

-1112;-0,348;-0,041;2

-1132;-0,33;0,091;2

-1152;-0,224;0,015;2

-1164;-0,08;-0,001;2

-1177;0,069;0,088;2

-1182;0,502;-0,063;2

-1188;0,651;0,041;2

-1185;0,253;0,072;2

-1169;0,478;-0,079;2

-1144;0,358;0,07;2

-1112;0,068;-0,039;2

-1079;0,134;0,01;2

-1042;-0,026;0,042;2

-1003;-0,108;0,148;2

-958;-0,263;0,083;2

-911;-0,069;-0,047;2

-868;0,072;0,043;2

-820;-0,183;0,118;2

-774;0,324;-0,083;2

-737;0,665;0,063;2

-725;0,437;-0,01;2

-734;0,513;-0,04;2

-765;0,188;0,082;2

-803;0,181;-0,083;2

-846;0,167;0,029;2

-888;0,003;0,023;2

-930;-0,061;-0,053;2

-969;-0,484;0,165;2

-1000;-0,826;-0,077;2

-1022;-0,736;-0,016;2

-1038;-0,731;0,121;2

-1047;-0,219;-0,085;2

-1060;0,113;0,111;2

-1074;0,246;-0,009;2

-1091;0,263;-0,015;2

-1109;0,085;0,053;2

-1128;0,333;-0,092;2

-1141;-0,083;0,066;2

-1149;0,045;0,02;2

-1147;0,183;-0,006;2

-1141;-0,289;0,063;2

-1138;-0,441;-0,074;2

-1141;-0,675;0,028;2

-1155;-0,524;-0,047;2

-1170;-0,883;-0,04;2

-1190;-1,072;0,091;2

-1195;-0,703;-0,049;2

-1185;-0,601;0,014;2

-1153;-0,608;0,035;2

-1107;-0,414;-0,055;2

-1056;-0,442;0,1;2

-1010;-0,05;-0,088;2

-963;0,004;-0,015;2

-920;-0,167;0,052;2

-877;-0,086;-0,054;2

-824;-0,672;0,083;2

-763;-0,777;-0,058;2

-698;-0,505;-0,056;2

-645;-0,37;0,071;2

-610;0,331;-0,08;2

-606;0,38;0,05;2

-632;-0,009;-0,027;2

-677;0,189;-0,013;2

-738;0,086;0,085;2

-793;-0,122;-0,066;2

-836;-0,034;0,072;2

-871;-0,187;-0,012;2

-899;-0,101;-0,049;2

-921;-0,193;0,2;2

-950;-0,083;-0,095;2

-977;0,253;0,133;2

-1011;-0,013;0,054;2

-1034;-0,035;-0,055;2

-1051;0,014;0,07;2

-1063;0,347;-0,049;2

-1074;0,442;-0,075;2

-1083;0,237;0,051;2

-1096;0,338;-0,106;2

-1105;0,203;0,072;2

-1118;0,226;-0,012;2

-1131;0,415;-0,032;2

-1148;0,304;0,073;2

-1158;0,408;-0,07;2

-1161;0,031;0,093;2

-1158;-0,212;-0,03;2

-1153;-0,162;-0,038;2

-1152;-0,338;0,064;2

-1151;-0,392;-0,075;2

-1143;-0,324;0,069;2

-1126;-0,313;0,019;2

-1107;-0,384;-0,063;2

-1081;-0,604;0,103;2

-1049;-0,065;-0,021;2

-1008;0,274;0,029;2

-956;-0,453;0,045;2

-901;-0,219;-0,075;2

-844;-0,344;0,085;2

-800;-0,191;-0,039;2

-758;-0,448;0,007;2

-720;-0,785;0,067;2

-690;-0,624;-0,082;2

-680;-0,306;0,058;2

-694;-0,017;-0,02;2

-727;-0,337;-0,027;2

-773;-0,162;0,07;2

-816;0,19;-0,061;2

-858;0,322;0,045;2

-893;0,4;-0,002;2

-930;0,585;-0,051;2

-964;0,329;0,061;2

-997;0,358;-0,06;2

-1019;0,397;0,026;2

-1038;0,286;0,117;2

-1050;0,023;0,006;2

-1059;-0,145;0,102;2

-1068;-0,068;-0,041;2

-1082;0,204;0,046;2

-1095;0,262;0,04;2

-1109;0,041;-0,09;2

-1125;0,176;0,088;2

-1138;-0,043;-0,001;2

-1149;0,155;-0,017;2

-1156;0,388;0,085;2

-1160;0,526;-0,098;2

-1162;0,098;0,079;2

-1168;-0,081;-0,013;2

-1172;-0,096;-0,022;2

-1182;-0,212;0,072;2

-1191;-0,245;-0,062;2

-1189;-0,323;0,028;2

-1176;-0,418;0,008;2

-1144;-0,013;-0,043;2

-1103;-0,26;0,073;2

-1051;-0,463;-0,065;2

-1003;-0,382;0,019;2

-957;-0,695;0,023;2

-920;-0,599;-0,065;2

-882;-0,682;0,047;2

-845;-0,776;-0,041;2

-810;-0,44;0,008;2

-767;-0,453;0,124;2

-725;-0,45;-0,103;2

-673;-0,134;0,142;2

-626;-0,214;-0,018;2

-592;-0,055;-0,01;2

-593;-0,14;0,065;2

-623;-0,325;-0,049;2

-680;-0,068;0,05;2

-744;-0,124;-0,033;2

-804;-0,573;-0,024;2

-854;-0,656;0,058;2

-898;-0,058;-0,076;2

-933;0,07;0,016;2

-958;-0,216;0,025;2

-982;0,14;0,052;2

-1002;-0,168;0,078;2

-1019;-0,609;-0,068;2

-1036;-0,425;-0,013;2

-1057;-0,149;0,042;2

-1077;0,387;-0,085;2

-1096;-0,085;0,071;2

-1109;-0,492;-0,04;2

-1123;-0,259;0,002;2

-1131;0,174;0,134;2

-1144;0,307;-0,046;2

-1156;0,152;0,063;2

-1169;0,112;-0,006;2

-1174;0,405;-0,029;2

-1183;0,069;0,066;2

-1189;-0,198;-0,079;2

-1192;0,045;0,046;2

-1193;-0,043;0,016;2

-1198;-0,154;-0,049;2

-1198;-0,139;0,1;2

-1183;-0,242;-0,028;2

-1161;-0,492;0,016;2

-1124;-0,518;0,018;2

-1088;-0,274;-0,104;2

-1048;-0,624;0,07;2

-1006;-0,673;-0,058;2

-966;-0,299;0,025;2

-917;-0,364;0,125;2

-870;-0,371;-0,083;2

-822;-0,262;0,069;2

-774;-0,138;-0,036;2

-725;0,18;-0,005;2

-682;-0,188;0,062;2

-634;0,061;-0,06;2

-590;0,386;0,063;2

-557;0,498;-0,002;2

-552;0,485;-0,052;2

-577;0,322;0,074;2

-633;0,499;-0,077;2

-699;0,555;0,047;2

-764;-0,095;0,001;2

-816;-0,008;-0,071;2

-862;0,015;0,057;2

-896;0,026;-0,071;2

-927;0,05;0,018;2

-947;0,125;0,011;2

-965;0,528;0,013;2

-981;0,615;0,018;2

-1001;0,852;-0,05;2

-1023;1,119;0,002;2

-1053;0,781;0,068;2

-1077;0,792;-0,066;2

-1102;0,838;0,037;2

-1120;0,106;-0,012;3

-1140;-0,154;-0,013;3

-1152;0,156;0,124;3

-1162;0,332;-0,036;3

-1168;0,131;0,165;3

-1172;-0,555;0,012;3

-1177;-0,67;-0,073;3

-1176;-0,616;0,083;3

-1177;-0,889;-0,069;3

-1178;-0,767;0,016;3

-1181;-0,468;0,017;3

-1183;-0,257;-0,079;3

-1176;-0,358;0,033;3

-1155;-0,322;-0,003;3

-1120;-0,119;0,014;3

-1082;0,086;0,041;3

-1037;0,435;-0,086;3

-991;0,5;0,076;3

-937;0,314;0,067;3

-883;0,3;-0,038;3

-822;0,11;0,012;3

-766;0,133;-0,072;3

-712;0,316;0,046;3

-665;0,1;0,002;3

-617;0,42;-0,017;3

-576;0,098;0,086;3

-550;-0,36;-0,072;3

-560;-0,137;0,018;3

-598;-0,355;-0;3

-657;-0,85;-0,069;3

-721;-0,809;0,083;3

-778;-0,59;-0,053;3

-829;-0,553;0,063;3

-877;-1,101;0,016;3

-916;-0,467;-0,06;3

-952;-0,31;0,032;3

-980;-0,473;-0,038;3

-1004;-0,477;-0,007;3

-1026;-0,458;0,054;3

-1041;0,125;-0,082;3

-1057;0,308;0,092;3

-1074;0,371;-0,029;3

-1089;0,833;-0,001;3

-1105;0,455;0,078;3

-1123;0,517;-0,092;3

-1143;0,882;0,074;3

-1162;0,68;-0,036;3

-1166;0,345;-0,064;3

-1163;0,203;-0,014;3

-1142;0,187;-0,067;3

-1116;0,298;0,04;3

-1086;0,319;-0,04;3

-1051;0,091;-0,083;3

-1012;-0,125;0,112;3

-965;-0,203;-0,042;3

-922;-0,3;0,031;3

-881;-0,1;0,047;3

-840;0,21;-0,077;3

-795;-0,108;0,045;3

-754;-0,056;-0,03;3

-718;0,13;-0,005;3

-709;0,035;0,09;3

-717;-0,016;-0,093;3

-746;-0,166;0,08;3

-784;-0,024;0,037;3

-828;0,122;-0,016;3

-873;-0,082;0,054;3

-917;-0,073;-0,078;3

-954;-0,198;0,043;3

-989;-0,341;0,007;3

-1019;-0,063;-0,048;3

-1046;-0,053;0,082;3

-1069;0,066;-0,063;3

-1084;0,104;0,156;3

-1099;-0,153;0,027;3

-1110;-0,202;-0,056;3

-1117;-0,055;0,059;3

-1110;-0,15;-0,077;3

-1088;0,02;0,003;3

-1051;0,082;0,061;3

-1010;0,077;-0,074;3

-965;-0,228;0,07;3

-927;-0,167;-0,04;3

-884;-0,112;0,005;3

-838;0,072;0,085;3

-787;-0,159;-0,091;3

-745;-0,208;0,064;3

-712;0,219;-0,005;3

-707;0,502;-0,022;3

-729;0,362;0,083;3

-770;0,236;-0,043;3

-821;0,025;0,027;3

-872;0,108;0,004;3

-921;0,432;-0,044;3

-959;0,354;0,095;3

-993;0,656;-0,071;3

-1013;0,616;0,007;3

-1031;0,494;0,017;3

-1044;0,492;-0,051;3

-1063;0,27;0,063;3

-1079;0,163;-0,084;3

-1095;-0,099;-0,018;3

-1110;-0,189;0,056;3

-1122;-0,2;-0,083;3

-1137;-0,218;0,032;3

-1152;-0,32;0,008;3

-1168;-0,463;0,021;3

-1179;-0,321;0,076;3

-1185;-0,424;-0,06;3

-1188;-0,478;0,033;3

-1191;-0,467;-0,003;3

-1194;-0,433;-0,036;3

-1198;-0,327;0,08;3

-1197;-0,206;-0,001;3

-1196;-0,203;0,032;3

-1199;0,121;0;3

-1211;0,389;-0,128;3

-1223;0,472;0,094;3

-1232;0,443;-0,041;3

-1223;0,617;0,017;3

-1199;0,606;0,045;3

-1159;0,819;-0,089;3

-1115;0,951;0,086;3

-1065;0,578;-0,018;3

-1018;0,197;-0,008;3

-965;-0,108;0,028;3

-916;0,099;-0,064;3

-863;-0,093;0,072;3

-814;-0,607;0,007;3

-762;-0,506;0,081;3

-710;-0,796;0,11;3

-662;-0,668;-0,091;3

-620;-0,184;0,027;3

-602;0,006;0,017;3

-607;0,164;-0,021;3

-643;0,453;0,094;3

-696;0,479;-0,062;3

-754;0,604;0,039;3

-812;0,575;0,013;3

-862;0,713;-0,043;3

-901;0,462;0,059;3

-930;0,473;-0,047;3

-954;0,724;-0,017;3

-971;0,638;0,037;3

-995;0,558;-0,089;3

-1015;0,309;0,085;3

-1038;0,055;-0,002;3

-1049;0,264;-0,012;3

-1053;0,04;0,075;3

-1036;-0,274;-0,073;3

-1005;-0,505;0,044;3

-965;-0,791;0,002;3

-923;-0,695;-0,031;3

-877;-0,882;0,094;3

-830;-0,804;-0,061;3

-775;-0,731;0,002;3

-720;-1,057;0,002;3

-669;-0,822;-0,08;3

-628;-0,455;0,084;3

-610;-0,418;-0,059;3

-631;0,253;-0,024;3

-682;0,518;0,041;3

-750;0,359;-0,106;3

-818;0,273;0,047;3

-876;0,763;-0,035;3

-920;0,991;0,014;3

-957;0,652;0,151;3

-987;0,426;0,019;3

-1010;0,063;0,099;3

-1034;0,056;-0,015;3

-1053;0,462;-0,011;3

-1076;0,311;0,079;3

-1099;0,192;-0,07;3

-1118;0,191;0,046;3

-1136;0,12;0,015;3

-1154;0,153;-0,036;3

-1174;0,517;0,097;3

-1188;-0,007;-0,054;3

-1199;-0,348;0,043;3

-1201;0,147;0,072;3

-1205;0,257;-0,065;3

-1208;0,083;0,16;3

-1217;-0,125;-0,05;3

-1222;0,062;0,016;3

-1227;0,007;0,035;3

-1227;0,273;-0,079;3

-1234;0,343;0,066;3

-1238;0,389;-0,018;3

-1243;0,388;-0,016;3

-1249;-0,118;0,094;3

-1246;0,088;-0,081;3

-1244;0,609;0,022;3

-1235;0,297;0,033;3

-1216;0,093;-0,037;3

-1188;0,04;0,09;3

-1158;0,208;-0,066;3

-1118;0,308;0,027;3

-1076;0,395;0,059;3

-1036;0,505;-0,05;3

-995;0,268;0,122;3

-955;0,195;-0,042;3

-918;0,295;-0,012;3

-884;-0,064;0,033;3

-852;0,238;0,003;3

-820;-0,088;0,083;3

-790;-0,308;-0,042;3

-758;0,123;-0,017;3

-726;0,167;0,048;3

-692;0,543;-0,072;3

-669;0,247;0,062;3

-659;0,054;-0,008;3

-672;0,419;-0,026;3

-704;0,233;0,102;3

-751;0,333;-0,004;3

-798;-0,019;0,056;3

-838;-0,307;0,023;3

-877;-0,486;-0,014;3

-907;-0,379;0,09;3

-940;-0,049;-0,055;3

-969;-0,302;0,006;3

-994;-0,47;0,041;3

-1012;-0,317;-0,06;3

-1023;-0,538;0,087;3

-1023;-0,61;-0,061;3

-1010;-0,295;0,017;3

-982;-0,41;0,065;3

-942;-0,339;-0,069;3

-903;-0,604;0,053;3

-861;-0,467;-0,004;3

-814;-0,012;0,011;3

-758;-0,087;0,068;3

-701;0,047;-0,092;3

-654;0,429;0,033;3

-636;0,434;-0,016;3

-646;0,753;-0,024;3

-686;0,557;0,077;3

-741;0,939;-0,097;3

-802;1,046;0,049;3

-852;0,406;0,007;3

-901;0,538;-0,052;3

-937;0,392;0,093;3

-971;0,338;-0,031;3

-998;0,467;0,037;3

-1021;0,383;0,036;3

-1041;0,266;0,003;3

-1062;0,297;0,086;3

-1077;-0,005;-0,047;3

-1092;-0,133;-0,015;3

-1105;-0,143;0,048;3

-1113;0,126;-0,078;3

-1125;0,118;0,079;3

-1134;-0,042;-0,037;3

-1145;-0,015;-0,017;3

-1157;-0,236;0,067;3

-1170;-0,058;-0,072;3

-1180;-0,222;0,076;3

-1185;-0,112;-0,012;3

-1187;0,019;-0,046;3

-1176;-0,251;0,067;3

-1157;-0,144;-0,052;3

-1124;-0,024;0,04;3

-1085;-0,052;0,022;3

-1036;0,416;-0,066;3

-987;0,443;0,07;3

-930;0,27;-0,079;3

-878;0,18;0,035;3

-820;-0,057;0,064;3

-764;0,139;-0,033;3

-708;0,156;0,012;3

-658;0,264;0,054;3

-619;0,245;-0,024;3

-604;-0,187;0,049;3

-615;0,306;-0,097;3

-658;0,278;0,049;3

-714;0,086;0,053;3

-776;0,445;-0,02;3

-831;-0,084;0,097;3

-875;-0,066;-0,084;3

-910;0,092;0,028;3

-938;0,322;0,016;3

-965;0,644;-0,046;3

-991;0,53;0,069;3

-1018;0,484;-0,078;3

-1042;0,48;0,016;3

-1065;0,367;0,012;3

-1082;0,472;-0,083;3

-1100;0,716;0,053;3

-1113;0,737;-0,067;3

-1127;0,497;0,004;3

-1136;0,503;0,042;3

-1147;0,601;-0,08;3

-1157;0,533;0,083;3

-1169;0,337;-0,016;3

-1178;0,348;-0,018;3

-1187;0,012;0,113;3

-1196;0,1;-0,066;3

-1203;-0,203;0,061;3

-1210;-0,448;-0,004;3

-1203;-0,209;-0,058;3

-1189;-0,076;0,095;3

-1158;-0,229;-0,067;3

-1130;0,118;0,039;3

-1098;0,195;0,008;3

-1065;0,477;-0,036;3

-1028;0,753;0,096;3

-984;0,656;-0,055;3

-938;0,179;0,03;3

-890;-0,421;0,041;3

-839;-0,29;-0,077;3

-786;-0,716;0,075;3

-735;-0,389;-0,042;3

-689;-0,245;0,004;3

-659;-0,478;0,074;3

-651;-0,595;-0,046;3

-675;-0,41;0,075;3

-719;-0,37;-0,043;3

-773;-0,079;-0,01;3

-826;-0,229;0,018;3

-876;-0,01;-0,092;3

-915;0,144;0,166;3

-947;0,172;0,003;3

-965;0,602;-0,044;3

-982;0,672;0,049;3

-999;0,559;-0,089;3

-1023;0,773;0,035;3

-1048;0,455;0,018;3

-1073;0,318;-0,067;3

-1094;0,226;0,077;3

-1119;0,123;0,02;3

-1134;-0,054;0,01;3

-1152;0,271;0,023;3

-1160;0,306;-0,09;3

-1166;0,194;0,017;3

-1167;0,276;-0,04;3

-1166;0,609;-0,018;3

-1171;0,551;0,052;3

-1179;0,852;-0,062;3

-1188;0,574;0,05;3

-1190;0,342;0,004;3

-1190;0,336;-0,004;3

-1189;0,267;0,046;3

-1178;0,348;-0,076;3

-1158;0,277;0,059;3

-1129;0,17;-0,002;3

-1093;0,314;-0,12;3

-1052;-0,112;0,089;3

-1010;-0,01;-0,067;3

-959;0,301;0,013;3

-908;0,07;0,026;3

-847;-0,037;-0,05;3

-784;-0,04;0,077;3

-722;-0,107;-0,05;3

-668;-0,115;0,023;3

-623;-0,237;0,05;3

-591;-0,586;-0,08;3

-581;-0,526;0,081;3

-604;-0,075;0,001;3

-648;0,176;-0,045;3

-712;0,098;0,089;3

-774;0,164;-0,063;3

-831;0,293;0,096;3

-872;0,031;0,002;3

-906;0,161;-0,024;3

-932;0,177;0,094;3

-957;0,051;-0,092;3

-972;0,022;0,052;3

-988;0,047;0,015;3

-999;0,345;-0,066;3

-1016;-0,12;0,102;3

-1034;-0,492;-0,122;3

-1054;-0,189;0,009;3

-1072;-0,221;0,063;3

-1088;-0,298;-0,067;3

-1102;-0,474;0,068;3

-1113;-0,415;-0,013;3

-1117;-0,215;-0,011;3

-1118;-0,513;0,054;3

-1122;-0,25;-0,039;3

-1123;-0,204;0,066;3

-1128;-0,537;-0,014;3

-1134;-0,081;-0,013;3

-1138;-0,09;0,045;3

-1137;-0,196;-0,066;3

-1123;-0,209;0,05;3

-1103;-0,227;0,023;3

-1073;-0,108;-0,008;3

-1044;-0,029;0,092;3

-1013;0,409;-0,091;3

-979;0,125;0,03;3

-936;-0,27;0,061;3

-889;-0,041;-0,08;3

-834;-0,058;0,081;3

-781;-0,33;-0,021;3

-722;-0,188;0;3

-671;0,243;-0,033;3

-630;0,5;-0,155;3

-604;0,698;0,048;3

-601;0,817;-0,048;3

-627;1,121;-0,032;3

-673;0,956;0,06;3

-726;0,532;-0,09;3

-781;0,6;0,053;3

-828;0,591;0,017;3

-865;0,186;-0,015;3

-893;-0,197;0,083;3

-919;0,117;-0,06;3

-941;0,572;0,025;3

-967;0,359;0,008;3

-988;0,401;-0,07;3

-1010;0,054;0,101;3

-1027;0,073;-0,074;3

-1044;-0,035;0,001;3

-1058;-0,216;-0,002;3

-1076;0,197;-0,072;3

-1088;0,07;0,078;3

-1101;-0,143;-0,046;3

-1113;0,187;-0,002;3

-1121;0,283;0,073;3

-1132;0,217;-0,082;3

-1133;0,476;0,202;3

-1131;0,849;0,03;3

-1128;0,688;-0,048;3

-1128;0,042;0,075;3

-1135;0,328;-0,018;3

-1136;0,19;0,049;3

-1128;-0,415;-0,017;3

-1107;-0,044;-0,045;3

-1080;0,071;0,047;3

-1044;-0,035;-0,042;3

-1006;-0,368;0,021;3

-956;-0,427;0,001;3

-905;-0,579;-0,061;3

-850;-0,919;0,055;3

-803;-0,929;-0,006;3

-755;-1,115;-0,007;3

-709;-1,175;0,035;3

-662;-0,733;-0,074;3

-617;-0,481;0,054;3

-584;-0,289;-0,028;3

-582;0,107;-0,011;3

-612;0,071;0,038;3

-669;0,145;-0,097;3

-735;0,021;0,074;3

-799;0,319;-0,006;3

-850;0,681;-0,047;3

-892;0,766;0,061;3

-929;1,133;-0,07;3

-958;1,197;0,025;3

-980;1,338;0,001;3

-997;1,145;-0,061;3

-1012;0,838;0,049;3

-1028;0,88;-0,04;3

-1048;0,464;0,03;3

-1061;0,283;0,016;3

-1081;0,279;-0,094;3

-1094;0,247;0,075;3

-1112;0,084;-0,044;3

-1128;-0,176;-0,101;3

-1142;-0,618;0,037;3

-1154;0,035;-0,074;3

-1161;0,146;0,054;3

-1167;-0,044;-0,026;3

-1164;0,003;-0,004;3

-1161;-0,376;0,081;3

-1158;-0,635;-0,079;3

-1157;-0,338;0,069;3

-1154;-0,362;0,016;3

-1152;-0,548;-0,008;3

-1146;-0,726;0,088;3

-1136;-0,437;-0,03;3

-1127;-0,232;0,031;3

-1114;-0,254;0,007;3

-1092;-0,326;-0,051;3

-1060;-0,523;0,089;3

-1018;-0,486;-0,069;3

-970;-0,361;0,013;3

-918;-0,37;0,025;3

-867;-0,163;-0,115;3

-818;-0,416;0,072;3

-766;-0,463;0,009;3

-718;-0,927;-0,007;3

-674;-1,317;0,056;3

-639;-0,846;-0,093;3

-610;-0,547;0,052;3

-604;-0,705;-0,025;3

-620;-0,53;-0,024;3

-663;-0,609;0,061;3

-718;-0,757;-0,069;3

-778;-0,442;0,074;3

-828;-0,23;-0,007;3

-870;-0,105;-0,049;3

-900;-0,126;0,09;3

-932;-0,134;-0,106;3

-957;0,134;0,029;3

-982;0,158;0,086;3

-1007;0,219;-0,057;3

-1031;0,185;0,103;3

-1048;0,289;-0,078;3

-1058;0,501;0,013;3

-1070;0,255;0,034;3

-1077;0,292;-0,07;3

-1084;0,033;0,079;3

-1091;0,049;-0,045;3

-1097;0,296;-0,006;3

-1108;-0,172;0,071;3

-1116;-0,072;-0,085;3

-1130;-0,098;0,065;3

-1137;-0,574;-0,024;3

-1143;-0,133;-0,036;3

-1144;0,043;0,06;3

-1154;0,208;-0,005;3

-1162;0,512;0,054;3

-1168;0,321;0,059;3

-1162;0,217;-0,051;3

-1145;-0,096;0,088;3

-1119;-0,149;-0,056;3

-1091;0,24;0,006;3

-1059;0,036;0,021;3

-1020;0,213;-0,047;3

-980;-0,161;0,094;3

-933;-0,468;-0,04;3

-888;0,163;-0,002;3

-841;-0,176;0,08;3

-794;-0,193;-0,078;3

-745;-0,184;0,101;3

-696;-0,192;-0,026;3

-649;0,268;-0,014;3

-623;0,464;0,063;3

-622;0,371;-0,075;3

-656;0,191;0,067;3

-704;0,307;-0,012;3

-767;0,124;-0,041;3

-823;-0,36;0,074;3

-872;-0,712;-0,061;3

-910;-0,718;0,03;3

-944;-0,739;0,018;3

-969;-0,64;-0,064;3

-992;-0,416;0,082;3

-1013;-0,641;-0,054;3

-1031;-0,528;0,011;3

-1054;-0,607;0,004;3

-1071;-0,506;-0,074;3

-1090;-0,263;0,082;3

-1106;-0,184;-0,058;3

-1120;-0,233;-0,065;3

-1131;-0,217;0,05;3

-1139;-0;-0,068;3

-1148;0,198;0,082;3

-1150;0,232;-0,012;3

-1154;0,583;-0,025;3

-1158;0,383;0,087;3

-1167;0,465;-0,04;3

-1174;0,51;0,04;3

-1183;0,421;0,014;3

-1187;0,309;-0,074;3

-1192;-0,132;0,081;3

-1183;-0,142;-0,075;3

-1164;-0,3;-0,029;3

-1137;-0,532;-0,01;3

-1103;-0,278;-0,047;3

-1071;-0,105;0,067;3

-1040;-0,004;-0,025;3

-1005;0,136;0,013;3

-963;-0,259;0,04;3

-919;0,013;-0,052;3

-872;-0,104;0,062;3

-823;-0,222;-0,046;3

-767;0,13;-0,013;3

-716;0,382;0,097;3

-673;0,556;-0,167;3

-654;0,193;0,055;3

-659;0,272;-0,02;3

-690;0,555;-0,021;3

-732;0,569;0,057;3

-783;0,701;-0,069;3

-827;0,298;0,016;3

-871;0,272;-0,009;3

-909;0,094;-0,076;3

-946;-0,085;0,106;3

-980;-0,052;-0,054;3

-1004;0,159;0,005;3

-1030;0,1;0,002;3

-1049;0,039;-0,071;3

-1070;-0,069;0,071;3

-1089;-0,113;-0,042;3

-1102;-0,089;0,001;3

-1115;-0,266;0,058;3

-1124;-0,182;-0,095;3

-1134;0,004;0,051;3

-1143;0,188;-0,027;3

-1153;0,211;-0,058;3

-1158;0,173;0,069;3

-1164;0,331;-0,094;3

-1171;0,182;0,031;3

-1183;-0,002;-0,023;3

-1192;0,205;-0,019;3

-1202;-0,267;0,076;3

-1200;-0,252;-0,087;3

-1189;-0,052;0,011;3

-1165;-0,212;0,013;3

-1134;0,181;-0,075;3

-1095;0,121;0,114;3

-1052;0,159;-0,037;3

-1005;0,034;0,012;3

-961;0,076;0,021;3

-916;0,205;-0,072;3

-873;0,083;0,054;3

-826;-0,049;-0,031;3

-778;-0,171;-0,009;3

-731;-0,596;0,07;3

-690;-0,478;-0,087;3

-657;-0,39;0,026;3

-644;-0,603;0,024;3

-655;-0,489;-0,02;3

-693;-0,258;0,063;3

-745;-0,11;-0,073;3

-797;-0,077;0,004;3

-849;0,028;-0,004;3

-886;-0,047;-0,038;3

-922;-0,022;0,098;3

-951;-0,024;-0,07;3

-979;-0,134;0,019;3

-1004;0,075;-0,004;3

-1025;0,448;-0,079;3

-1047;0,617;0,087;3

-1065;0,646;-0,063;3

-1084;1,017;0;3

-1095;0,76;0,035;3

-1108;0,587;-0,069;3

-1118;0,503;0,061;3

-1128;0,424;-0,048;3

-1137;0,571;-0,004;3

-1148;-0,175;0,075;3

-1154;-0,192;0,025;3

-1162;0,129;0,067;3

-1166;0,249;0,026;3

-1158;0,255;-0,004;3

-1145;0,128;0,061;3

-1122;0,36;-0,08;3

-1093;0,464;0,038;3

-1059;0,476;0,019;3

-1026;0,329;-0,055;3

-988;0,049;0,08;3

-954;0,175;-0,058;3

-908;0,083;0,009;3

-859;-0,373;0,03;3

-801;-0,062;-0,146;3

-750;-0,33;0,086;3

-706;-0,352;-0,078;3

-681;0,13;0,007;3

-680;-0,191;0,028;3

-705;-0,123;-0,096;3

-747;-0,127;0,082;3

-802;-0,09;-0,019;3

-853;-0,075;0,039;3

-901;-0,415;0,079;3

-936;-0,455;-0,085;3

-966;-0,9;0,054;3

-993;-0,723;-0,023;3

-1017;-0,249;-0,049;3

-1040;-0,766;0,077;3

-1060;-0,925;-0,084;3

-1076;-0,697;0,012;3

-1088;-0,886;-0,004;3

-1104;-0,478;-0,061;3

-1118;-0,599;0,082;3

-1130;-0,521;-0,03;3

-1140;-0,09;0,055;3

-1144;-0,014;0,053;3

-1153;0,124;-0,096;3

-1160;0,109;0,08;3

-1167;0,092;0,1;3

-1163;0,285;0,018;3

-1149;0,039;0,073;3

-1122;0,158;-0,09;3

-1092;0,219;0,066;3

-1058;0,084;0,005;3

-1023;0,02;-0,04;3

-982;-0,2;0,036;3

-940;-0,2;-0,052;3

-893;-0,327;0,054;3

-850;-0,304;0,044;3

-806;0,12;-0,048;3

-762;-0,089;0,047;3

-716;-0,226;0,022;3

-672;-0,177;0,025;3

-640;-0,42;0,027;3

-632;0,329;-0,079;3

-654;0,095;0,06;3

-696;-0,06;-0,041;3

-752;0,009;-0,002;3

-806;-0,404;0,022;3

-857;0,054;-0,098;3

-897;0,285;0,096;3

-936;0,224;-0,028;3

-966;0,43;-0,005;3

-994;0,594;0,064;3

-1016;0,493;-0,095;3

-1038;0,599;0,058;3

-1056;0,734;0,005;3

-1076;0,635;-0,033;3

-1090;0,46;0,076;3

-1107;0,43;-0,068;3

-1121;0,212;0,049;3

-1132;-0,129;-0,01;3

-1144;-0,205;-0,015;3

-1151;-0,129;0,102;3

-1160;-0,197;-0,072;3

-1168;0,013;0,029;3

-1178;0,014;0,001;3

-1183;0,061;-0,059;3

-1181;-0,329;0,064;3

-1175;-0,585;-0,045;3

-1160;-0,019;0,005;3

-1144;0,054;0,061;3

-1119;0,179;-0,039;3

-1092;0,196;0,074;3

-1057;0,019;-0,013;3

-1024;0,22;-0,023;3

-991;-0,038;0,064;3

-965;0,127;-0,063;3

-934;0,293;0,042;3

-907;0,253;0,056;3

-884;0,102;-0,041;3

-872;0,083;0,071;3

-864;0,201;-0,047;3

-862;0,381;0,028;3

-862;0,205;0,036;3

-878;0,084;-0,071;3

-899;-0,24;0,08;3

-927;0,038;-0,034;3

-959;0,05;-0,027;3

-989;-0,581;0,078;3

-1014;-0,185;-0,057;3

-1037;-0,482;0,06;3

-1063;-0,937;-0,046;3

-1086;-0,944;-0,004;3

-1105;-0,772;0,095;3

-1115;-0,299;-0,083;3

-1121;0,045;0,164;3

-1123;0,023;-0,004;3

-1130;0,012;0,009;3

-1133;-0,11;0,076;3

-1146;0,314;-0,088;3

-1151;0,271;0,038;3

-1148;0,137;0,009;3

-1134;0,38;-0,027;3

-1109;0,183;0,113;3

-1080;0,036;-0,089;3

-1045;-0,05;0,022;3

-1006;-0,006;0,03;3

-963;0,244;-0,055;3

-919;-0,127;0,094;3

-874;-0,24;-0,042;3

-828;-0,222;-0,01;3

-783;-0,521;0,058;3

-740;-0,299;-0,098;3

-706;-0,2;0,058;3

-688;-0,386;-0,054;3

-699;-0,422;-0,043;3

-732;-0,651;0,078;3

-782;-0,208;-0,119;3

-830;0,289;0,069;3

-871;-0,02;-0,018;3

-901;0,051;-0,064;3

-924;-0,156;0,045;3

-937;-0,129;-0,074;3

-951;-0,202;0,016;3

-964;-0,087;0,029;3

-985;0,063;-0,047;3

-1007;0,251;0,105;3

-1026;0,467;-0,064;3

-1042;0,339;0,011;3

-1060;0,064;-0,005;3

-1075;0,052;-0,066;3

-1091;-0,186;0,066;3

-1103;-0,299;-0,049;3

-1117;0,186;0,039;3

-1130;0,214;0,068;3

-1144;0,095;-0,081;3

-1155;0,224;0,039;3

-1165;0,075;-0,05;3

-1171;-0,049;-0,007;3

-1178;-0,235;0,091;3

-1180;0,15;-0,058;3

-1174;0,116;0,173;3

-1160;-0,328;0,051;3

-1134;-0,03;-0,055;3

-1101;-0,276;0,053;3

-1066;-0,083;-0,043;3

-1034;-0,226;0,012;3

-1005;-0,609;0,063;3

-968;-0,683;-0,08;3

-930;-0,358;0,068;3

-882;0,066;-0,045;3

-829;-0,029;0,007;3

-775;0,118;0,053;3

-720;0,38;-0,119;3

-667;0,374;0,077;3

-633;-0,131;-0,057;3

-626;0,099;-0,021;3

-654;0,196;0,056;3

-703;0,32;-0,072;3

-762;0,449;0,079;3

-814;0,724;-0,031;3

-859;0,616;-0,023;3

-900;0,678;0,118;3

-937;0,824;-0,052;3

-970;0,596;0,001;3

-994;0,264;0,007;3

-1014;0,443;-0,003;3

-1031;-0,002;0,092;3

-1047;-0,713;-0,139;3

-1063;-0,538;0,026;3

-1081;-0,859;0,029;3

-1092;-0,68;-0,062;3

-1100;-0,475;0,118;3

-1111;-0,153;-0,05;3

-1123;-0,026;0,011;3

-1135;-0,145;0,029;3

-1144;0,229;-0,076;3

-1151;0,009;0,064;3

-1154;0,511;-0,046;3

-1158;1,005;0,023;3

-1169;0,62;0,066;3

-1168;0,676;-0,066;3

-1157;0,709;0,052;3

-1130;0,473;0,018;3

-1096;0,383;-0,05;3

-1065;0,288;0,091;3

-1029;-0,083;-0,08;3

-993;-0,048;0,04;3

-952;-0,103;-0,001;3

-907;0,21;-0,065;3

-856;0,339;0,082;3

-805;-0,12;-0,082;3

-749;-0,369;0,015;3

-701;-0,32;0,049;3

-657;-0,508;-0,089;3

-635;-0,738;0,071;3

-644;-0,873;0,013;3

-682;-0,636;-0,014;3

-732;-0,647;0,014;3

-786;-0,551;-0,075;3

-835;-0,393;0,052;3

-878;-0,422;-0,038;3

-916;-0,034;-0,025;3

-950;0,375;0,079;3

-976;0,604;-0,083;3

-996;0,422;0,024;3

-1016;0,003;0,019;3

-1034;0,145;-0,055;3

-1051;0,091;0,069;3

-1068;-0,05;-0,076;3

-1081;0,169;0,03;3

-1091;0,198;0,041;3

-1101;0,607;-0,092;3

-1109;0,298;0,117;3

-1118;0,041;-0,041;3

-1127;0,152;-0,017;3

-1138;-0,153;0,053;3

-1145;-0,338;-0,033;3

-1154;0,094;0,077;3

-1159;-0,423;0,014;3

-1160;-0,704;0,002;3

-1146;-0,885;0,075;3

-1124;-0,892;-0,104;3

-1095;-0,759;0,052;3

-1066;-0,635;0,005;3

-1030;-0,32;-0,029;3

-998;-0,537;0,08;3

-963;-0,352;-0,059;3

-919;-0,134;0,042;3

-874;-0,035;0,024;3

-820;0,61;-0,028;3

-770;0,625;0,1;3

-718;0,214;0,015;3

-673;0,454;0,002;3

-635;0,346;-0,015;3

-613;-0,205;-0,074;3

-612;-0,475;0,067;3

-640;-0,485;-0,052;3

-687;-0,535;0,002;3

-742;-0,702;0,027;3

-796;-0,352;-0,056;3

-842;0,042;0,056;3

-882;-0,303;-0,015;3

-916;-0,154;-0,063;3

-951;-0,222;0,078;3

-979;-0,116;-0,058;3

-1005;0,075;-0,051;3

-1024;0,085;0,009;3

-1039;0,169;-0,079;3

-1049;-0,072;0,085;3

-1064;-0,574;-0,078;3

-1075;-0,808;0,021;3

-1091;-0,501;0,053;3

-1102;-0,114;-0,072;3

-1114;0,017;0,08;3

-1125;0,547;-0,06;3

-1139;0,596;-0,028;3

-1151;0,45;0,045;3

-1163;0,573;-0,112;3

-1165;0,278;0,052;3

-1168;0,135;-0,022;3

-1166;0,208;-0,016;3

-1171;-0,035;0,066;3

-1174;-0,052;-0,072;3

-1172;-0,244;0,052;3

-1172;-0,151;0,008;3

-1168;-0,168;-0,023;3

-1164;-0,257;0,087;3

-1144;0,117;-0,016;3

-1114;-0,391;0,003;3

-1078;-0,574;0,009;3

-1031;-0,282;-0,06;3

-986;-0,501;0,098;3

-938;-0,239;-0,144;3

-892;-0,044;0,01;3

-838;-0,241;0,02;3

-784;0,087;-0,104;3

-726;0,077;0,064;3

-675;0,171;-0,056;3

-627;0,024;0;3

-594;-0,154;0,035;3

-579;0,273;-0,083;3

-596;0,105;0,037;3

-635;-0,301;0,008;3

-689;-0,342;-0,035;3

-744;-0,265;0,089;3

-797;-0,483;-0,086;3

-840;-0,707;0,072;3

-884;-0,315;-0,007;3

-916;-0,011;-0,044;3

-944;-0,317;0,071;3

-965;-0,398;-0,007;3

-984;-0,325;0,016;3

-999;-0,331;0,007;3

-1018;0,064;-0,081;3

-1033;0,268;-0,038;3

-1052;-0,096;-0,023;3

-1064;-0,069;0,02;3

-1077;-0,101;0,065;3

-1091;-0,015;-0,078;3

-1102;0,127;0,085;3

-1112;0,122;-0,044;3

-1118;0,594;-0,008;3

-1126;0,627;0,06;3

-1132;0,455;-0,102;3

-1139;0,102;0,057;3

-1143;-0,047;0,005;3

-1135;0,09;-0,058;3

-1116;-0,147;0,098;3

-1088;-0,38;-0,074;3

-1057;-0,514;0,044;3

-1018;-0,527;-0,029;3

-979;-0,034;-0,067;3

-928;-0,011;0,092;3

-879;0,086;-0,064;3

-828;0,3;0,011;3

-778;-0,213;0,043;3

-731;-0,101;-0,064;3

-686;-0,044;0,065;3

-642;-0,042;-0,068;3

-605;-0,316;-0,017;3

-590;-0,453;0,046;3

-604;-0,032;-0,089;3

-650;-0,162;0,045;3

-704;-0,279;-0,032;3

-765;-0,427;-0,005;3

-819;-0,452;0,067;3

-870;0,081;-0,062;3

-911;0,281;0,075;3

-950;0,006;0,018;3

-976;-0,184;-0,067;3

-1000;0,161;0,086;3

-1021;0,388;-0,055;3

-1041;0,229;0,092;3

-1057;0,042;0,019;3

-1074;0,402;-0,057;3

-1086;0,148;0,096;3

-1101;0,261;-0,075;3

-1116;-0,078;-0,021;3

-1130;-0,417;0,017;3

-1143;-0,206;-0,085;3

-1150;-0,603;0,071;3

-1158;-0,798;-0,029;3

-1162;-0,289;0,012;3

-1171;-0,383;0,084;3

-1178;-0,291;-0,092;3

-1174;-0,086;0,053;3

-1156;-0,038;-0,019;3

-1127;0,162;-0,011;3

-1091;0,007;0,091;3

-1055;0,143;-0,065;3

-1024;0,122;0,053;3

-990;-0,334;0,006;3

-954;-0,05;-0,014;3

-910;0,223;0,064;3

-869;0,233;-0,05;3

-822;0,347;0,029;3

-772;0,439;0,024;3

-720;0,865;-0,003;3

-678;0,609;0,076;3

-657;0,522;-0,054;3

-666;0,544;-0,009;3

-702;0,312;0,037;3

-753;0,11;-0,011;3

-809;-0,174;0,078;3

-857;-0,196;-0,023;3

-898;-0,077;-0,018;3

-933;-0,25;0,075;3

-966;0,046;-0,078;3

-995;0,226;0,042;3

-1024;0,107;-0,007;3

-1049;0,191;-0,028;3

-1073;0,232;0,073;3

-1090;0,35;-0,064;3

-1108;0,144;-0,003;3

-1121;0,033;0,023;3

-1137;-0,151;-0,065;3

-1148;-0,564;0,072;3

-1159;-0,531;-0,074;3

-1166;-0,159;0,035;3

-1174;-0,269;0,047;3

-1180;0,139;-0,052;3

-1190;-0,166;0,075;3

-1194;-0,218;0,028;3

-1198;0,168;0,019;3

-1196;-0,473;0,052;3

-1189;-0,377;-0,081;3

-1174;-0,398;0,085;3

-1153;-0,729;0,014;3

-1126;-0,164;-0,01;3

-1087;-0,266;0,058;3

-1044;-0,005;-0,073;3

-997;0,159;0,045;3

-950;0,19;0,006;3

-900;0,783;-0,019;3

-844;0,67;0,079;3

-786;0,317;-0,05;3

-731;0,199;-0;3

-688;-0,228;0,018;3

-654;-0,118;-0,072;3

-649;-0,129;0,18;3

-665;-0,491;-0,055;3

-710;-0,384;-0;3

-763;-0,395;0,038;3

-822;-0,336;-0,048;3

-871;-0,302;0,073;3

-916;-0,301;-0,027;3

-947;-0,327;-0,009;3

-978;-0,337;0,144;3

-1001;-0,04;-0,067;3

-1028;0,196;0,074;3

-1047;-0,057;-0,011;3

-1065;0,013;-0,033;3

-1076;-0,298;0,073;3

-1090;-0,354;-0,105;3

-1104;-0,44;0,067;3

-1121;-0,32;-0,008;3

-1135;0,176;-0,011;3

-1149;0,205;0,091;3

-1157;-0,18;-0,043;3

-1166;-0,183;0,004;3

-1174;-0,073;0,02;3

-1181;-0,42;-0,075;3

-1186;-0,396;0,076;3

-1179;0,023;-0,022;3

-1161;0,307;-0,016;3

-1132;0,34;0,049;3

-1104;0,817;-0,054;3

-1067;0,832;0,065;3

-1027;0,638;0,009;3

-982;0,416;0;3

-924;0,003;0,111;3

-870;-0,349;-0,08;3

-817;-0,208;0,184;3

-764;-0,327;0,02;3

-716;0,102;-0,025;3

-673;0,37;0,046;3

-646;0,455;-0,07;3

-649;0,286;0,083;3

-684;0,158;-0,002;3

-731;0,176;-0,094;3

-786;0,33;0,078;3

-832;0,403;-0,037;3

-874;0,361;0,018;3

-910;-0,357;0,021;3

-944;-0,636;-0,115;3

-975;-0,785;0,08;3

-1005;-0,473;-0,046;3

-1027;-0,089;-0,01;3

-1046;-0,072;0,07;3

-1065;0,188;-0,134;3

-1079;-0,117;0,068;3

-1095;0,05;-0,029;3

-1111;0,181;-0,015;3

-1129;-0,045;0,056;3

-1143;0,172;-0,079;3

-1158;-0,124;0,047;3

-1167;-0,401;-0,009;3

-1179;0,294;0,089;3

-1186;0,598;0,081;3

-1184;0,201;-0,052;3

-1161;-0,058;0,032;3

-1136;0,052;0,024;3

-1101;0,398;-0,058;3

-1069;0,298;0,064;3

-1043;0,003;-0,033;3

-1025;-0,241;-0,022;3

-1008;-0,479;0,02;3

-995;-0,132;-0,113;3

-993;-0,06;0,088;3

-996;0,014;-0,037;3

-1011;0,253;-0,036;3

-1030;0,226;0,05;3

-1048;0,127;-0,095;3

-1065;0,297;0,056;3

-1078;0,125;-0,013;3

-1092;0,156;-0,036;3

-1105;0,334;0,092;3

-1117;0,169;-0,074;3

-1128;0,281;0,045;3

-1141;0,492;0,013;3

-1151;0,257;-0,048;3

-1164;-0,169;0,085;3

-1172;-0,278;-0,13;3

-1173;0,125;0,024;3

-1156;0,219;0,034;3

-1119;0,405;-0,076;3

-1073;0,491;0,067;3

-1028;0,404;-0,063;3

-985;0,233;0,014;3

-942;-0,219;0,053;3

-905;-0,05;-0,09;3

-869;0,419;0,141;3

-832;0,112;-0,015;3

-782;-0,085;-0,034;3

-727;0,056;0,093;3

-675;0,017;-0,055;3

-647;0,095;0,057;3

-646;0,058;0,041;3

-680;-0,1;-0,042;3

-730;-0,139;0,065;3

-790;0,031;-0,042;3

-843;-0,266;0,026;3

-893;-0,667;-0,021;3

-929;-0,63;-0,052;3

-959;-0,474;0,093;3

-986;-0,194;-0,041;3

-1007;0,036;-0,016;3

-1030;-0,336;-0,002;3

-1050;-0,322;-0,082;3

-1072;-0,222;0,085;3

-1087;-0,3;-0,013;3

-1104;-0,256;-0,032;3

-1119;-0,128;0,065;3

-1129;-0,159;-0,069;3

-1142;0,252;0,079;3

-1147;0,287;-0,014;3

-1154;-0,242;-0,045;3

-1161;-0,437;0,148;3

-1169;0,095;-0,058;3

-1166;-0,013;0,076;3

-1150;0,089;0,026;3

-1115;0,278;-0,083;3

-1078;-0,405;0,099;3

-1038;-0,386;-0,067;3

-997;-0,426;0,017;3

-948;-0,47;0,043;3

-896;-0,453;-0,071;3

-841;-0,32;0,072;3

-790;-0,116;-0,056;3

-741;-0,005;-0,039;3

-696;0,043;0,062;3

-662;0,291;-0,071;3

-655;0,046;0,069;3

-682;-0,14;-0,011;3

-726;0,277;-0,033;3

-785;-0,004;0,058;3

-842;-0,037;-0,065;3

-889;0,025;0,073;3

-929;0,141;-0,003;3

-964;0,255;-0,028;3

-990;-0,574;0,07;3

-1016;-0,436;-0,044;3

-1034;0,096;-0,013;3

-1058;0,224;0,013;3

-1076;0,471;-0,086;3

-1094;0,315;0,093;3

-1110;0,605;-0,088;3

-1124;0,437;0,002;3

-1133;-0,244;0,042;3

-1142;-0,198;-0,067;3

-1154;-0,109;0,043;3

-1165;-0,035;-0,021;3

-1180;-0,045;-0,006;3

-1193;-0;0,045;3

-1202;0,077;-0,057;3

-1208;0,142;0,073;3

-1200;0,282;-0,099;3

-1182;0,277;-0,049;3

-1152;0,066;0,077;3

-1118;0,02;-0,073;3

-1073;0,061;0,046;3

-1030;-0,219;-0,014;3

-979;0,046;-0,029;3

-931;0,083;0,093;3

-877;0,074;-0,073;3

-826;0,032;0,038;3

-774;-0,114;0,025;3

-730;-0,248;-0,073;3

-694;-0,077;0,135;3

-679;0,324;-0,022;3

-689;0,264;-0,012;3

-724;0,333;0,041;3

-775;0,266;-0,079;3

-828;0,239;0,086;3

-881;0,583;-0,024;3

-924;0,491;-0,036;3

-961;0,756;0,068;3

-990;0,752;-0,074;3

-1014;0,087;0,061;3

-1037;-0,631;0,011;3

-1062;-0,845;-0,028;3

-1083;-0,658;0,094;3

-1108;-0,476;-0,075;3

-1122;-0,224;0,051;3

-1138;0,189;0,029;3

-1148;0,591;-0,039;3

-1162;0,491;0,074;3

-1170;0,285;-0,066;3

-1177;0,18;0,022;3

-1185;0,04;0,016;3

-1191;0,196;-0,082;3

-1200;0,196;0,068;3

-1207;0,734;-0,049;3

-1214;1,057;0,071;3

-1218;0,712;0,048;3

-1217;0,712;-0,08;3

-1205;0,592;0,06;3

-1178;0,419;-0,02;3

-1146;0,587;-0,019;3

-1106;0,276;0,034;3

-1066;0,462;-0,105;3

-1016;0,104;0,045;3

-969;-0,134;-0,012;3

-919;0,052;-0,017;3

-874;-0,195;0,083;3

-830;-0,334;-0,077;3

-787;-0,143;0,026;3

-734;-0,628;0,011;3

-680;-0,692;-0,032;3

-627;-0,725;0,094;3

-599;-0,328;-0,056;3

-607;-0,1;0,001;3

-651;-0,368;0,034;3

-706;-0,169;-0,077;3

-767;0,074;0,087;3

-818;-0,027;-0,055;3

-864;-0,223;-0,002;3

-904;-0,034;0,061;3

-945;0,355;-0,096;3

-975;0,166;0,062;3

-1002;0,352;0,07;3

-1026;0,46;-0,024;3

-1049;0,04;0,086;3

-1068;-0,258;-0,07;3

-1084;0,007;0,054;3

-1096;0,029;0,004;3

-1110;-0,084;-0,018;3

-1125;-0,084;0,095;3

-1142;-0,166;-0,07;3

-1159;-0,105;0,022;3

-1167;0,165;0,026;3

-1173;0,485;-0,151;3

-1176;0,173;0,114;3

-1184;0,131;-0,049;3

-1196;0,4;0,049;3

-1206;0,2;0,037;3

-1209;0,302;-0,094;3

-1191;0,046;0,08;3

-1163;-0,078;0,038;3

-1128;-0,089;0,011;3

-1099;-0,352;0,045;3

-1064;-0,339;-0,078;3

-1029;-0,159;0,066;3

-984;-0,105;-0,004;3

-941;0,194;-0,013;3

-893;-0,015;0,106;3

-845;0,096;-0,072;3

-795;-0,184;0,027;3

-745;-0,21;0,022;3

-694;0,345;-0,063;3

-658;0,656;0,082;3

-644;0,403;-0,07;3

-665;0,514;0,003;3

-707;0,669;0,003;3

-764;0,672;-0,063;3

-818;0,655;0,043;3

-869;0,804;-0,143;3

-913;0,591;0,001;3

-951;0,302;0,04;3

-975;0,19;-0,062;3

-996;-0,16;0,089;3

-1015;-0,216;-0,015;3

-1036;0,118;-0,028;3

-1056;-0,005;0,088;3

-1073;-0,049;-0,06;3

-1089;-0,211;0,059;3

-1103;-0,3;0,013;3

-1118;-0,101;-0,054;3

-1134;-0,188;0,055;3

-1147;-0,547;-0,061;3

-1154;-0,622;0,015;3

-1159;-0,815;0,01;3

-1159;-0,406;-0,066;3

-1163;-0,413;0,077;3

-1168;-0,696;-0,049;3

-1172;-0,516;0,012;3

-1181;-0,597;0,052;3

-1184;-0,632;-0,106;3

-1190;-0,5;0,052;3

-1180;-0,287;-0,029;3

-1162;0,295;-0,045;3

-1134;0,275;-0,004;3

-1100;0,437;-0,076;3

-1060;-0,007;0,064;3

-1015;-0,394;0,007;3

-964;-0,12;-0,029;3

-915;-0,014;0,076;3

-859;0,062;-0,042;3

-807;-0,1;0,094;3

-756;-0,354;-0,01;3

-708;-0,065;-0,03;3

-671;-0,111;0,066;3

-656;0,138;-0,065;3

-672;0,484;0,023;3

-709;0,123;0,08;3

-758;0,066;-0,073;3

-807;0,025;0,054;3

-855;0,016;-0,063;3

-897;-0,018;-0,036;3

-937;-0,072;0,063;3

-970;-0,277;-0,082;3

-999;-0,54;0,055;3

-1020;-0,39;-0,04;3

-1043;-0,037;0,004;3

-1061;0,037;0,067;3

-1083;0,238;-0,088;3

-1100;-0,151;0,041;3

-1117;-0,073;-0,019;3

-1128;0,69;0,007;3

-1143;0,627;0,103;3

-1149;0,831;-0,092;3

-1159;0,814;0,027;3

-1165;0,444;0,005;3

-1172;0,789;-0,053;3

-1180;0,32;0,085;3

-1186;0,159;-0,077;3

-1191;-0,267;-0,028;3

-1192;-0,699;0,024;3

-1190;-0,484;0,054;3

-1183;-0,227;0,087;3

-1170;-0,044;-0,026;3

-1148;-0,029;-0,016;3

-1121;0,17;0,072;3

-1092;0,55;-0,135;3

-1051;0,477;0,058;3

-1001;0,495;-0,014;3

-949;0,523;-0,042;3

-900;0,187;0,097;3

-850;0,016;-0,061;3

-805;-0,151;0,071;3

-754;-0,541;-0,01;3

-704;-0,432;-0,048;3

-662;-0,502;0,054;3

-640;-0,577;-0,054;3

-644;-0,613;0,004;3

-680;-0,874;-0,017;3

-727;-0,352;-0,067;3

-783;-0,451;0,089;3

-830;-0,584;-0,058;3

-873;-0,288;0,029;3

-908;-0,258;0,023;3

-939;-0,41;-0,085;3

-966;-0,215;0,06;3

-993;-0,096;-0,016;3

-1018;-0,061;0,018;3

-1042;-0,039;0,086;3

-1060;0,457;-0,12;3

-1076;0,631;0,06;3

-1085;-0,067;0,013;3

-1094;-0,147;0,006;3

-1105;-0,226;0,084;3

-1118;-0,462;-0,08;3

-1132;-0,181;0,048;3

-1146;0,231;0,009;3

-1161;0,26;-0,061;3

-1170;0,158;0,096;3

-1182;0,296;-0,073;3

-1187;0,659;0,019;3

-1189;0,058;0,02;3

-1190;-0,024;-0,071;3

-1188;-0,205;0,077;3

-1179;-0,051;-0,033;3

-1157;0,332;-0,046;3

-1135;-0,077;0,066;3

-1102;-0,159;-0,056;3

-1065;0,086;0,078;3

-1019;0,13;-0,023;3

-969;0,519;-0,009;3

-914;0,295;-0,011;3

-856;0,274;-0,067;3

-797;0,469;0,045;3

-737;0,68;-0,027;3

-679;0,351;-0,08;3

-633;-0,152;0,087;3

-610;-0,097;-0,06;3

-610;-0,051;0,01;3

-646;-0,009;-0,005;3

-701;0,248;-0,05;3

-757;-0,002;0,085;3

-805;-0,252;-0,047;3

-846;-0,38;-0,007;3

-880;-0,604;0,037;3

-911;-0,545;-0,078;3

-939;-0,521;0,064;3

-968;-0,629;-0,02;3

-990;-0,18;0,005;3

-1010;0,34;0,08;3

-1028;0,654;-0,095;3

-1047;0,443;0,052;3

-1060;0,176;-0,019;3

-1074;0,452;0,001;3

-1086;0,582;0,071;3

-1099;0,634;-0,087;3

-1112;0,927;0,031;3

-1126;0,88;-0,007;3

-1135;0,845;-0,062;3

-1138;0,915;0,091;3

-1147;0,466;-0,074;3

-1151;0,3;-0,015;3

-1162;0,348;0,04;3

-1174;0,296;-0,044;3

-1173;0,147;0,07;3

-1163;0,103;-0,055;3

-1142;0,446;0,008;3

-1125;0,788;0,034;3

-1106;0,466;-0,195;3

-1091;0,21;0,07;3

-1074;0,585;-0,032;3

-1056;0,204;-0,034;3

-1034;-0,46;0,066;3

-1013;-0,46;-0,066;3

-982;-0,788;0,064;3

-941;-0,698;0,007;3

-885;-0,299;-0,059;3

-829;-0,349;0,084;3

-772;-0,445;-0,056;3

-719;-0,593;0,023;3

-672;-0,55;0,052;3

-637;-0,232;-0,049;3

-605;-0,324;0,099;3

-594;-0,468;-0,015;3

-611;-0,668;0,006;3

-658;-0,426;0,024;3

-717;-0,599;-0,089;3

-782;-0,732;0,034;3

-835;-0,515;-0,023;3

-881;-0,078;-0,002;3

-917;-0,204;0,055;3

-950;-0,345;-0,137;3

-978;-0,043;0,094;3

-1004;-0,219;-0,038;3

-1026;-0,023;-0,03;3

-1047;0,11;0,015;3

-1063;0,043;-0,063;3

-1072;0,005;0,016;3

-1079;-0,198;0,015;3

-1078;-0,497;-0,052;3

-1080;-0,661;0,108;3

-1083;-0,275;-0,053;3

-1078;-0,033;0,039;3

-1064;-0,407;0,03;3

-1044;-0,213;-0,063;3

-1023;-0,557;0,009;3

-994;-1,039;-0,012;3

-960;-0,575;0,011;3

-918;-0,588;0,043;3

-880;-0,586;-0,086;3

-846;-0,382;0,059;3

-817;-0,206;-0,081;3

-782;0,104;0,008;3

-748;0,055;0,099;3

-731;-0,348;-0,079;3

-748;0,026;0,079;3

-791;0,115;-0,028;3

-846;-0,405;0,062;3

-901;-0,664;0,121;3

-942;-0,289;-0,058;3

-979;-0,207;-0,209;3

-1009;0,293;-0,001;3

-1040;0,99;-0,047;3

-1062;-0,02;0,069;3

-1086;-0,495;-0,072;3

-1103;-0,214;0,027;3

-1116;0,183;0,048;3

-1129;0,411;-0,061;3

-1145;0,254;0,076;3

-1153;0,11;-0,054;3

-1166;0,413;-0,018;3

-1179;0,205;0,006;3

-1194;0,125;-0,092;3

-1205;-0,121;0,079;3

-1218;-0,093;-0,017;3

-1225;0,067;0,02;3

-1226;-0,384;0,08;3

-1229;-0,145;-0,133;3

-1227;0,15;0,055;3

-1220;-0,174;-0,022;3

-1198;0,014;-0,034;3

-1170;-0,096;0,062;3

-1136;-0,553;-0,107;3

-1102;-0,3;0,054;3

-1065;-0,277;0,029;3

-1024;-0,056;-0,08;3

-980;-0,083;0,092;3

-927;0,175;-0,043;3

-878;0,299;-0,001;3

-830;0,027;0,029;3

-785;0,76;-0,088;3

-742;0,54;0,067;3

-707;0,479;-0,043;3

-691;0,831;-0,009;3

-706;0,521;0,084;3

-748;0,577;-0,079;3

-802;0,551;0,071;3

-854;0,527;0,001;3

-896;0,437;-0,018;3

-930;0,068;0,056;3

-961;0,103;-0,072;3

-991;-0,199;-0,003;3

-1013;-0,32;-0,017;3

-1035;-0,24;-0,061;3

-1053;-0,218;0,097;3

-1071;-0,178;-0,059;3

-1088;-0,163;0,105;3

-1108;-0,256;0,009;3

-1120;0,196;-0,05;3

-1133;0,123;0,078;3

-1140;0,082;-0,046;3

-1153;0,171;-0,016;3

-1160;0,101;0,055;3

-1172;0,047;-0,091;3

-1177;0,135;0,052;3

-1179;-0,007;-0,003;3

-1180;0,268;-0,011;3

-1183;0,41;0,088;3

-1183;0,214;0,006;3

-1170;0,147;0,058;3

-1148;0,095;-0,003;3

-1111;0,742;-0,045;3

-1072;0,596;0,08;3

-1023;0,534;-0,07;3

-971;0,523;0,038;3

-918;0,223;0,033;3

-862;0,563;-0,087;3

-815;0,16;0,078;3

-767;0,453;-0,063;3

-719;0,801;-0,004;3

-677;0,67;0,094;3

-643;0,45;-0,055;3

-635;0,308;0,127;3

-656;0,133;-0,049;3

-699;0,375;-0,004;3

-753;0,419;0,104;3

-807;0,45;-0,087;3

-856;0,402;0,089;3

-900;0,248;0,011;3

-937;0,262;-0,016;3

-968;0,45;0,069;3

-988;0,79;-0,054;3

-1009;0,556;0,026;3

-1027;0,503;-0,089;3

-1049;0,283;-0,046;3

-1068;0,166;0,098;3

-1085;0,014;-0,068;3

-1100;-0,076;0,026;3

-1120;-0,349;0,033;3

-1138;-0,433;-0,071;3

-1156;-0,278;0,079;3

-1167;0,045;-0,048;3

-1175;-0,052;0,016;3

-1180;-0,2;0,043;3

-1187;-0,023;-0,084;3

-1193;-0,428;0,123;3

-1202;-0,435;-0,039;3

-1211;-0,133;-0,008;3

-1215;-0,217;0,087;3

-1204;0,178;-0,081;3

-1180;-0,021;0,129;3

-1149;-0,299;-0,011;3

-1110;-0,054;-0,023;3

-1070;0,258;0,075;3

-1026;0,045;-0,072;3

-985;-0,063;0,034;3

-952;0,006;0,01;3

-917;0,304;-0,06;3

-890;-0,079;0,091;3

-860;-0,072;-0,07;3

-834;0,434;0,034;3

-817;0,286;0,04;3

-814;0,361;-0,077;3

-820;0,21;0,048;3

-836;0,231;-0,04;3

-862;0,538;0,005;3

-895;0,062;0,086;3

-929;0,037;-0,063;3

-964;0,501;0,062;3

-998;0,518;-0,023;3

-1024;0,423;-0,067;3

-1052;0,625;0,087;3

-1069;1,193;-0,072;3

-1085;0,983;0,062;3

-1095;0,538;0,001;3

-1106;0,351;-0,045;3

-1116;-0,064;0,082;3

-1134;0,018;-0,081;3

-1149;0,126;0,046;3

-1159;0,023;0,034;3

-1160;-0,125;-0,036;3

-1166;-0,069;0,07;3

-1172;0,043;0,037;3

-1185;0,05;0,012;3

-1196;0,412;0,041;3

-1207;0,609;-0,09;3

-1201;0,637;0,07;3

-1178;0,603;-0,058;3

-1145;0,999;0,013;3

-1118;0,61;0,064;3

-1094;0,322;-0,075;3

-1071;0,195;0,086;3

-1045;-0,588;-0,175;3

-1007;-0,452;-0,024;3

-967;-0,582;0,13;3

-919;-0,463;-0,067;3

-872;-0,186;0,085;3

-824;-0,358;0,005;3

-767;-0,2;-0,019;3

-707;-0,639;0,062;3

-654;-0,626;-0,049;3

-630;-0,458;0,022;3

-641;-0,802;-0,006;3

-684;-1,141;-0,059;3

-735;-1,105;0,085;3

-790;-0,82;-0,064;3

-837;-0,326;-0,009;3

-879;-0,404;0,014;3

-914;-0,549;-0,077;3

-946;0,051;0,076;3

-972;-0,305;-0,021;3

-995;-0,508;-0,025;3

-1014;-0,279;0,108;3

-1032;-0,387;-0,1;3

-1051;-0,213;0,096;3

-1067;0,068;0,033;3

-1082;0,014;-0,023;3

-1094;-0,214;0,077;3

-1105;-0,437;-0,076;3

-1113;-0,167;-0,054;3

-1124;-0,273;-0,016;3

-1128;-0,28;-0,009;3

-1136;-0,302;0,127;3

-1148;0,073;-0,052;3

-1160;0,46;0,005;3

-1168;0,564;0,025;3

-1171;0,605;-0,061;3

-1159;0,415;0,078;3

-1146;0,231;-0,05;3

-1132;-0,168;-0,006;3

-1122;-0,067;0,068;3

-1103;0,092;-0,008;3

-1074;-0,363;0,114;3

-1036;-0,23;-0,014;3

-987;-0,015;-0,022;3

-933;0,16;0,087;3

-864;-0,06;-0,058;3

-795;-0,057;0,071;3

-736;-0,241;0,03;3

-689;-0,241;-0,054;3

-658;-0,396;0,098;3

-640;-0,032;-0,036;3

-643;0,195;0,021;3

-663;0,127;0,003;3

-701;0,153;-0,036;3

-745;0,296;0,111;3

-794;0,604;-0,05;3

-844;0,27;-0,005;3

-901;0,062;0,032;3

-951;0,427;-0,076;3

-993;0,683;0,071;3

-1018;0,489;-0,03;3

-1026;0,428;-0,001;3

-1033;0,019;0,087;3

-1048;0,309;-0,05;3

-1074;0,165;0,056;3

-1101;-0,22;-0,041;3

-1122;-0,206;-0,029;3

-1132;-0,077;0,079;3

-1136;-0,123;-0,045;3

-1138;-0,28;-0,008;3

-1147;-0,342;0,008;3

-1158;-0,233;-0,054;3

-1173;-0,849;0,113;3

-1184;-0,762;-0,061;3

-1196;-0,62;0,031;3

-1199;-0,849;0,021;3

-1189;-0,61;-0,114;3

-1157;-0,28;0,151;3

-1120;-0,362;-0,038;3

-1078;0,021;-0,002;3

-1043;0,031;0,028;3

-1005;0,265;-0,128;3

-962;0,285;0,091;3

-910;0,223;-0,123;3

-847;0,319;-0,016;3

-789;0,243;0,056;3

-736;0,201;-0,078;3

-688;0,185;0,082;3

-646;0,404;0,016;3

-614;0,384;-0,125;3

-607;0,016;0,121;3

-627;0,125;-0,065;3

-674;0,276;0,034;3

-728;-0,034;0,006;3

-793;-0,134;-0,055;3

-853;-0,339;0,103;3

-911;-0,164;-0,046;3

-954;0,231;-0,008;3

-983;-0,235;0,02;3

-997;-0,063;-0,089;3

-1007;-0,337;0,057;3

-1022;-0,233;-0,038;3

-1042;-0,005;0,038;3

-1067;-0,394;0,059;3

-1088;-0,118;-0,19;3

-1109;0,179;0,053;3

-1127;0,308;-0,003;3

-1144;0,171;0,002;3

-1154;-0,23;0,087;3

-1165;0,207;-0,059;3

-1171;0,233;0,073;3

-1182;0,131;-0,071;3

-1191;0,474;-0,049;3

-1199;0,404;0,112;3

-1203;0,578;-0,151;3

-1209;0,617;0,033;3

-1208;0,459;0,038;3

-1202;0,365;-0,09;3

-1184;0,156;0,067;3

-1164;0,175;-0,043;3

-1137;0,142;-0,017;3

-1107;-0,136;0,012;3

-1067;-0,134;-0,094;3

-1024;-0,297;0,077;3

-984;-0,379;0,016;3

-937;0,165;-0,024;3

-893;-0,084;0,161;3

-851;-0,438;-0,103;3

-806;-0,179;0,035;3

-773;0,278;0,009;3

-751;0,552;-0,024;3

-743;-0,138;0,093;3

-738;-0,118;-0,061;3

-748;0,134;0,018;3

-772;-0,064;0,009;3

-815;0,293;-0,049;3

-863;0,141;0,122;3

-911;-0,211;-0,051;3

-946;-0,083;0,008;3

-974;-0,443;0,02;3

-997;-0,153;-0,111;3

-1020;-0,533;0,087;3

-1043;-0,498;-0,097;3

-1067;-0,361;0;3

-1089;-0,498;0,055;3

-1109;-0,128;-0,083;3

-1129;-0,049;0,025;3

-1143;0,117;-0,013;3

-1152;0,403;-0,024;3

-1156;0,341;0,092;3

-1163;0,21;-0,081;3

-1165;0,084;0,019;3

-1169;-0,237;-0,007;3

-1174;-0,126;-0,052;3

-1175;-0,072;0,092;3

-1165;-0,141;-0,057;3

-1154;-0,197;-0;3

-1130;0,018;0,025;3

-1102;0,039;-0,085;3

-1067;0,253;0,092;3

-1032;-0,127;0,002;3

-994;-0,36;-0,01;3

-958;-0,935;0,052;3

-911;-0,981;-0,016;3

-857;-0,491;0,032;3

-808;-0,163;-0,1;3

-767;-0,155;0,034;3

-748;-0,439;0,081;3

-755;-0,039;-0,077;3

-790;0,605;0,027;3

-826;0,535;0,032;3

-848;0,244;-0,005;3

-850;-0,391;0,07;3

-837;-0,376;-0,052;3

-822;-0,497;0,023;3

-797;-0,652;0,098;3

-775;-0,477;-0,03;3

-757;-0,367;0,161;3

-762;-0,252;-0,048;3

-793;0,062;0,026;3

-847;0,034;0,016;3

-898;-0,079;-0,132;3

-948;0,03;0,076;3

-986;-0,024;-0,042;3

-1021;-0,148;-0,008;3

-1046;-0,22;0,087;3

-1051;-0,509;-0,075;3

-1029;-1,457;0,047;3

-998;-1,471;-0,042;3

-965;-0,584;0,007;3

-922;-0,198;0,081;3

-876;-0,198;-0,092;3

-838;-0,167;0,009;3

-815;-0,131;-0,056;3

-822;-0,23;-0,04;3

-863;-0,436;0,1;3

-918;-0,226;-0,088;3

-974;-0,292;0,068;3

-1016;-0,477;0,011;3

-1045;-0,145;-0,081;3

-1068;-0,375;0,165;3

-1097;-0,388;0,033;3

-1124;0,096;-0,027;3

-1150;0,089;0,033;3

-1164;0,188;-0,139;3

-1175;0,264;0,098;3

-1187;0,331;0,015;3

-1199;0,675;0,005;3

-1218;0,661;0,027;3

-1227;0,649;-0,101;3

-1226;0,417;0,083;3

-1198;-0,225;-0,017;3

-1155;-0,387;-0,049;3

-1104;-0,323;0,093;3

-1054;-0,096;-0,081;3

-1012;0,095;0,027;3

-972;-0,303;0,022;3

-941;-0,25;-0,089;3

-925;-0,063;0,083;3

-935;-0,233;-0,063;3

-960;-0,569;0,01;3

-994;-0,834;-0,107;3

-1022;-0,242;-0,208;3

-1049;-0,045;0,082;3

-1072;-0,187;-0,014;3

-1100;0,125;0,124;3

-1128;-0,216;-0,009;3

-1154;-0,038;-0,036;3

-1169;0,173;0,079;3

-1180;0,045;-0,048;3

-1184;0,065;-0,009;3

-1192;0,087;0,053;3

-1197;0,711;-0,101;3

-1210;0,523;0,047;3

-1213;0,45;-0,016;3

-1206;0,629;-0,135;3

-1183;0,284;0,07;3

-1157;0,579;-0,068;3

-1123;0,731;-0,028;3

-1084;0,032;0,218;3

-1031;0,717;-0,103;3

-977;0,193;0,076;3

-925;0,107;-0,054;3

-886;-0,004;-0,003;3

-868;-0,436;0,038;3

-874;0,037;-0,047;3

-896;0,164;0,056;3

-925;0,163;-0,031;3

-960;0,261;0,024;3

-991;0,096;0,069;3

-1020;0,605;-0,013;3

-1050;0,883;0,076;3

-1073;0,36;-0,022;3

-1090;0,28;-0,028;3

-1109;0,136;0,064;3

-1126;-0,056;-0,042;3

-1137;-0,156;0,065;3

-1148;-0,109;0,018;3

-1151;-0,096;-0,051;3

-1156;-0,4;0,042;3

-1157;-0,31;-0,039;3

-1167;0,11;0,028;3

-1178;0,212;0,024;3

-1188;0,523;-0,056;3

-1197;0,592;0,088;3

-1202;0,415;-0,041;3

-1195;0,223;-0,003;3

-1170;0,044;0,091;3

-1131;0,321;-0,056;3

-1086;0,266;0,155;3

-1038;0,354;0,007;3

-987;0,435;0,009;3

-936;0,032;0,055;3

-876;0,426;-0,075;3

-820;0,185;0,063;3

-764;0,227;0,088;3

-714;0,55;-0,003;3

-673;0,082;0,075;3

-647;-0,182;-0,096;3

-636;0,152;0,036;3

-654;0,249;0,031;3

-690;0,338;-0,062;3

-745;0,352;0,082;3

-799;0,466;-0,055;3

-848;0,206;0,033;3

-886;0,116;0,028;3

-922;-0,111;-0,089;3

-954;-0,146;0,05;3

-985;-0,389;-0,056;3

-1010;-0,465;0,002;3

-1030;-0,576;-0,03;3

-1050;-0,049;-0,064;3

-1065;0,168;0,121;3

-1081;-0,098;-0,037;3

-1097;0,121;-0,007;3

-1114;0,305;0,069;3

-1125;0,327;-0,058;3

-1129;0,247;0,052;3

-1136;0,152;-0,002;3

-1144;0,468;-0,035;3

-1157;0,51;0,175;3

-1168;0,203;-0,055;3

-1160;0,29;0,039;3

-1128;0,187;0,007;3

-1089;0,029;-0,087;3

-1044;0,26;0,071;3

-999;0,219;-0,076;3

-946;-0,134;0,002;3

-900;-0,042;0,047;3

-852;0,223;-0,063;3

-812;0,182;0,072;3

-764;0,111;-0,051;3

-721;0,31;0,001;3

-680;0,602;0,047;3

-660;0,339;-0,068;3

-662;0,16;0,129;3

-701;-0,207;0,081;3

-751;-0,068;0,005;3

-808;-0,287;0,07;3

-857;-0,252;-0,066;3

-901;-0,591;0,074;3

-938;-0,291;-0,011;3

-974;0,211;-0,029;3

-1005;0,07;0,103;3

-1034;-0,005;-0,015;3

-1062;0,149;0,019;3

-1090;-0,062;0,009;3

-1116;0,11;0,103;3

-1129;0,152;0,009;3

-1137;0,419;-0,074;3

-1135;0,382;0,036;3

-1143;0,37;0,058;3

-1153;0,579;-0,097;3

-1166;0,263;0,091;3

-1184;0,329;0,019;3

-1196;0,558;0,011;3

-1214;0,441;0,068;3

-1222;0,573;-0,083;3

-1221;0,47;0,191;3

-1205;0,162;0,029;3

-1178;-0,222;0,016;3

-1141;-0,387;0,089;3

-1105;-0,269;-0,046;3

-1065;-0,174;0,025;3

-1025;-0,184;0,009;3

-982;-0,442;-0,043;3

-942;-0,108;0,086;3

-906;0,153;-0,074;3

-863;-0,045;0,016;3

-823;-0,185;0,084;3

-784;0,184;-0,022;3

-754;0,12;0,057;3

-739;0,071;-0,044;3

-749;0,391;-0,008;3

-781;0,465;-0,048;3

-832;0,683;-0,064;3

-878;0,155;0,103;3

-922;-0,03;-0,011;3

-955;0,382;-0;3

-986;0,45;0,069;3

-1015;0,791;-0,066;3

-1047;0,968;0,017;3

-1071;1,224;0,019;3

-1096;1,182;0,039;3

-1113;0,661;0,025;3

-1127;0,506;-0,083;3

-1134;0,277;-0,001;3

-1143;0,203;0,163;3

-1151;0,014;-0,093;3

-1158;-0,186;0,057;3

-1166;-0,147;0,001;3

-1173;-0,112;0,066;3

-1184;-0,296;0,043;3

-1190;-0,234;-0,085;3

-1187;-0,361;0,08;3

-1169;-0,178;-0,038;3

-1142;-0,044;0,058;3

-1111;-0,053;0,252;3

-1072;-0,126;-0,082;3

-1026;-0,006;0,111;3

-974;0,168;0,13;3

-923;0,281;-0,061;3

-869;0,213;0,508;3

-821;0,168;-0,021;3

-763;-0,209;0,012;3

-708;-0,184;0,004;3

-656;0,489;-0,039;3

-623;0,49;0,196;3

-605;0,404;-0,054;3

-626;0,442;0,038;3

-670;-0,072;0,004;3

-732;0,242;-0,069;3

-795;0,384;0,071;3

-851;0,049;-0,035;3

-895;0,535;-0,013;3

-930;0,85;0,113;3

-961;0,721;-0,081;3

-989;0,272;0,198;3

-1020;0,267;0,046;3

-1050;0,157;-0,031;3

-1072;-0,116;0,047;3

-1092;-0,02;0,095;3

-1106;-0,051;0,118;3

-1125;-0,495;-0,023;3

-1142;-0,276;-0,022;3

-1157;-0,376;0,076;3

-1164;-0,163;-0,046;3

-1173;0,315;0,074;3

-1178;0,275;0,001;3

-1190;0,208;-0,058;3

-1205;-0,103;0,086;3

-1206;-0,487;-0,088;3

-1199;-0,146;-0;3

-1180;0,011;0,029;3

-1156;-0,229;-0,154;3

-1126;-0,403;0,045;3

-1089;-0,245;-0,04;3

-1045;-0,102;-0,001;3

-992;-0,002;0,048;3

-949;0,519;-0,109;3

-919;0,299;0,041;3

-908;0,63;0,047;3

-911;0,35;-0,027;3

-931;-0,025;0,118;3

-961;-0,234;0,055;3

-1000;-0,159;0,032;3

-1033;0,174;-0,047;3

-1064;0,363;-0,095;3

-1083;0,209;0,313;3

-1100;-0,01;-0,072;3

-1117;-0,136;0,059;3

-1139;-0,07;0,031;3

-1156;0,026;-0,004;3

-1172;-0,028;0,098;3

-1181;0,006;-0,012;3

-1189;0,297;-0,027;3

-1198;0,115;0,029;3

-1213;0,213;-0,153;3

-1223;0,302;0,184;3

-1227;0,33;-0,054;3

-1228;0,367;0,143;3

-1220;0,108;0,021;3

-1198;0,275;-0,062;3

-1170;0,331;0,074;3

-1131;-0,116;-0,011;3

-1086;-0,392;-0,005;3

-1033;-0,346;0,208;3

-979;-0,424;-0,045;3

-926;-0,33;-0,068;3

-875;-0,251;0,064;3

-827;-0,154;-0,047;3

-778;-0,149;0,06;3

-739;-0,026;-0,065;3

-711;-0,155;-0,005;3

-708;-0,369;0,145;3

-728;-0,199;-0,074;3

-767;-0,191;0,034;3

-822;-0,164;0,024;3

-871;-0,139;-0,009;3

-919;0,012;0,024;3

-953;-0,35;-0,073;3

-986;-0,204;0,083;3

-1010;-0,259;0,013;3

-1034;-0,596;-0,007;3

-1057;-0,292;0,178;3

-1078;-0,534;-0,058;3

-1100;-0,398;0,048;3

-1119;-0,229;-0,006;3

-1133;-0,208;-0,052;3

-1147;0,031;0,106;3

-1159;0,086;-0,072;3

-1170;0,434;0,027;3

-1181;0,046;0,015;3

-1187;0,114;-0,074;3

-1198;0,61;0,089;3

-1205;0,628;-0,121;3

-1211;0,684;-0,02;3

-1208;0,512;0,069;3

-1202;0,284;-0,061;3

-1178;0,107;0,075;3

-1154;0,029;-0,016;3

-1121;0,072;-0,012;3

-1091;0,004;0,094;3

-1052;-0,139;-0,062;3

-1015;-0,01;0,079;3

-973;-0,152;-0,009;3

-932;0,034;-0,018;3

-883;-0,283;0,07;3

-831;-0,15;-0,053;3

-791;0,292;0,025;3

-777;-0,003;0,09;3

-787;0,267;-0,048;3

-819;0,477;0,077;3

-862;0,535;-0,057;3

-897;0,97;0,044;3

-934;0,475;0,052;3

-966;0,373;-0,065;3

-995;0,319;0,017;3

-1023;-0,035;-0,049;3

-1042;-0,072;-0,004;3

-1062;-0,28;0,06;3

-1080;0,056;-0,078;3

-1103;0,023;0,084;3

-1119;-0,181;0,052;3

-1133;-0,019;-0,008;3

-1139;-0,059;0,082;3

-1144;-0,048;-0,171;3

-1153;0,047;0,056;3

-1162;0,201;0,003;3

-1169;0,325;-0,049;3

-1169;0,333;-0,97;3

-1157;0,274;-0,064;3

-1135;-0,118;-0,009;3

-1116;-0,154;-0,018;3

-1089;0,031;-0,001;3

-1062;-0,139;0,128;3

-1029;-0,16;-0,11;3

-994;-0,07;0,136;3

-941;0,16;0,03;3

-884;0,381;-0,081;3

-825;0,19;0,041;3

-776;0,476;-0,042;3

-739;0,613;0,008;3

-728;0,117;0,161;3

-740;0,274;-0,096;3

-780;0,116;-0,05;3

-830;-0,1;-0,032;3

-877;0,038;-0,005;3

-918;-0,105;0,161;3

-953;-0,036;-0,067;3

-984;0,125;0,052;3

-1011;-0,009;0,018;3

-1036;-0,151;-0,023;3

-1056;-0,34;0,112;3

-1075;-0,358;-0,072;3

-1088;-0,137;0,052;3

-1100;-0,149;0,043;3

-1111;-0,13;-0,074;3

-1120;-0,206;0,089;3

-1133;0,167;-0;3

-1144;0,197;-0,046;3

-1158;0,088;0,044;3

-1167;0,213;-0,079;3

-1165;0,025;0,086;3

-1146;-0,101;-0,059;3

-1110;0,195;-0,017;3

-1068;-0,181;0,06;3

-1018;-0,053;-0,084;3

-972;-0,046;0,069;3

-928;-0,329;-0,039;3

-884;-0,094;-0,015;3

-838;-0,038;0,099;3

-793;-0,085;-0,113;3

-746;0,187;0,024;3

-707;-0,127;0,002;3

-668;-0,017;-0,067;3

-652;0,189;0,085;3

-660;0,312;-0,051;3

-696;0,031;0,059;3

-745;0,209;0,016;3

-800;0,461;-0,073;3

-850;0,331;0,088;3

-897;0,45;-0,049;3

-936;0,393;0,002;3

-970;0,275;0,059;3

-998;0,025;-0,079;3

-1027;0,128;-0,039;3

-1056;-0,15;-0,03;3

-1079;-0,118;-0,01;3

-1098;-0,22;0,096;3

-1108;-0,048;-0,062;3

-1118;0,001;0,065;3

-1129;-0,056;0,024;3

-1146;0,112;-0,052;3

-1160;-0,186;0,101;3

-1174;-0,197;-0,016;3

-1181;-0,069;0,022;3

-1187;-0,154;-0,041;3

-1195;0,192;-0,036;3

-1201;-0,044;0,084;3

-1210;0,277;-0,066;3

-1210;0,595;0,003;3

-1204;0,218;0,044;3

-1179;0,296;-0,134;3

-1142;0,41;0,065;3

-1097;0,166;-0,043;3

-1044;0,139;0,046;3

-984;-0,353;0,061;3

-924;0,044;-0,008;3

-864;0,205;0,078;3

-812;-0,33;-0,008;3

-761;0,163;-0,002;3

-709;-0,005;0,088;3

-654;-0,432;-0,082;3

-594;-0,246;0,021;3

-548;0,164;0,035;3

-520;-0,176;-0,012;3

-529;-0,591;0,08;3

-569;-0,354;-0,085;3

-636;0,003;0,022;3

-708;0,121;-0,012;3

-778;0,214;-0,054;3

-836;0,317;0,085;3

-886;0,666;0,035;3

-921;0,236;-0,001;3

-955;0,2;0,047;3

-978;0,038;-0,066;3

-1004;-0,118;0,073;3

-1027;-0,603;-0,016;3

-1052;-0,253;-0,029;3

-1072;-0,219;0,059;3

-1091;-0,114;-0,047;3

-1105;-0,27;0,042;3

-1125;-0,322;0,002;3

-1142;-0,225;-0,03;3

-1158;-0,305;0,088;3

-1172;0,168;0,05;3

-1174;0,08;0,052;3

-1174;-0,161;0,04;3

-1175;0,318;-0,062;3

-1180;0,097;0,084;3

-1188;0,287;-0,082;3

-1190;0,23;0,01;3

-1181;-0,289;0,008;3

-1153;-0,049;-0,016;3

-1119;0,227;0,083;3

-1080;-0,02;-0,046;3

-1045;0,073;-0,006;3

-1002;0,081;0,065;3

-963;-0,138;0,037;3

-914;-0,503;0,09;3

-864;-0,397;-0,003;3

-809;-0,043;-0,032;3

-754;-0,187;0,069;3

-697;-0,206;-0,058;3

-637;-0,183;0,036;3

-574;0,02;0,013;3

-521;0,1;-0,055;3

-475;0,07;0,077;3

-451;0;-0,064;3

-463;0,104;0,04;3

-511;0,436;0,022;3

-584;0,547;-0,031;3

-668;-0,03;0,089;3

-744;-0,235;-0,04;3

-811;0,232;-0,009;3

-865;0,258;0,058;3

-906;0,597;-0,078;3

-937;0,383;0,064;3

-960;0,255;-0,006;3

-984;0,128;-0,043;3

-1006;-0,36;0,066;3

-1030;-0,087;-0,058;3

-1052;-0,335;0,059;3

-1078;-0,398;0,009;3

-1107;-0,148;-0,04;3

-1138;-0,005;0,08;3

-1159;0,262;-0,06;3

-1175;0,42;0,063;3

-1181;0,434;0,026;3

-1184;0,761;-0,039;3

-1191;0,609;-0,037;3

-1194;0,426;-0,048;3

-1192;0,385;0,009;3

-1172;0,148;0,043;3

-1144;0,351;-0,06;3

-1118;0,088;0,071;3

-1095;-0,136;-0,048;3

-1067;0,065;-0,068;3

-1028;-0,097;0,057;3

-975;0,071;-0,09;3

-911;-0,091;0,042;3

-846;-0,254;-0,03;3

-778;0,327;-0,027;3

-723;0,296;0,088;3

-690;0,568;-0,035;3

-696;0,781;0,074;3

-732;0,702;-0,024;3

-790;0,689;-0,065;3

-849;0,215;0,107;3

-901;0,359;-0,071;3

-941;0,47;-0,009;3

-976;0,013;0,012;3

-1003;0,229;-0,077;3

-1032;0,232;0,022;3

-1051;0,069;-0,064;3

-1071;-0,007;0,012;3

-1085;-0,236;0,03;3

-1104;-0,345;-0,01;3

-1126;-0,418;0,016;3

-1151;-0,05;-0,027;3

-1170;0,417;-0,006;3

-1187;0,167;0,033;3

-1194;0,275;-0,07;3

-1198;0,29;0,044;3

-1195;-0,002;-0,011;3

-1181;0,011;-0,018;3

-1152;0,224;0,103;3

-1115;-0,149;-0,063;3

-1082;-0,472;0,084;3

-1044;-0,43;-0,034;3

-1006;-0,288;-0,109;3

-962;-0,497;0,085;3

-911;-0,31;-0,057;3

-857;-0,216;0,021;3

-794;-0,659;0,032;3

-731;-0,495;0,063;3

-661;-0,317;0,114;3

-595;-0,441;-0,006;3

-534;-0,498;0,014;3

-488;-0,151;0,045;3

-470;-0,349;-0,088;3

-498;-0,497;0,122;3

-562;-0,241;-0,012;3

-651;-0,468;-0,022;3

-734;-0,648;0,121;3

-808;-0,342;-0,074;3

-860;-0,157;0,064;3

-898;0,008;-0,052;3

-925;0,183;-0,044;3

-954;-0,294;0,07;3

-980;-0,32;-0,063;3

-1008;-0,144;0,062;3

-1034;-0,248;0,053;3

-1054;-0,099;0,006;3

-1074;-0,01;0,099;3

-1090;-0,351;0,02;3

-1110;-0,208;-0,013;3

-1130;-0,191;0,04;3

-1147;0,573;-0,089;3

-1161;0,693;0,09;3

-1174;0,383;0,067;3

-1184;0,585;0,036;3

-1194;0,442;0,052;3

-1192;0,121;-0,077;3

-1183;-0,161;0,078;3

-1155;-0,09;-0,012;3

-1124;-0,097;-0,018;3

-1089;-0,202;0,084;3

-1044;0,32;-0,087;3

-990;0,121;0,045;3

-923;-0,307;0,031;3

-856;0,314;-0,047;3

-788;0,199;0,082;3

-723;0,304;-0,076;3

-660;0,467;0,034;3

-596;0,319;0,01;3

-536;0,423;-0,077;3

-502;0,512;0,1;3

-512;0,462;-0,059;3

-562;0,421;-0,022;3

-639;0,194;0,011;3

-716;0,115;-0,076;3

-782;0,588;0,076;3

-834;0,386;-0,018;3

-882;-0,11;-0,022;3

-921;-0,159;0,141;3

-958;-0,24;-0,119;3

-987;-0,085;0,064;3

-1018;-0,313;0,005;3

-1044;-0,379;-0,028;3

-1073;-0,09;0,123;3

-1096;0,196;-0,037;3

-1116;0,295;0,063;3

-1130;0,155;-0,018;3

-1140;0,269;-0,051;3

-1147;0,449;0,096;3

-1158;0,369;-0,058;3

-1169;0,501;0,014;3

-1190;0,248;0,199;3

-1206;0,036;-0,022;3

-1223;-0,337;0,091;3

-1236;-0,133;-0,047;3

-1247;0,045;0,008;3

-1247;0,136;0,077;3

-1233;-0,11;-0,083;3

-1197;-0,336;0,068;3

-1149;-0,152;-0,005;3

-1098;0,085;-0,034;3

-1053;-0,02;0,001;3

-1012;0,169;-0,063;3

-969;0,027;0,014;3

-926;-0,084;-0,094;3

-872;0,275;-0,029;3

-823;0,326;0,122;3

-775;0,098;-0,077;3

-729;0,306;0,014;3

-685;-0,082;0,019;3

-641;0,233;-0,064;3

-607;0,264;0,116;3

-594;-0,131;-0,066;3

-613;-0,037;0,018;3

-657;-0,008;0,028;3

-719;0,287;-0,071;3

-778;0,058;0,073;3

-833;0,06;-0,07;3

-881;0,498;-0,005;3

-927;0,48;0,083;3

-962;0,58;-0,076;3

-994;0,416;0,063;3

-1020;0,159;-0,016;3

-1039;0,194;-0,017;3

-1057;0,153;0,122;3

-1072;0,465;-0,026;3

-1092;0,155;0,048;3

-1116;-0,283;0,019;3

-1140;0,082;-0,034;3

-1160;0,127;0,121;3

-1183;0,248;-0,014;3

-1198;0,475;-0,022;3

-1211;0,064;0,024;3

-1215;0,417;-0,067;3

-1220;0,871;0,065;3

-1210;0,734;-0,09;3

-1188;0,264;0,003;3

-1156;0,582;0,083;3

-1123;0,617;-0,104;3

-1080;0,057;0,118;3

-1031;-0,029;-0,049;3

-975;0,232;-0,004;3

-921;-0,181;-0,004;3

-870;0,049;-0,056;3

-812;0,007;0,057;3

-762;-0,176;0,001;3

-706;-0,449;-0,026;3

-668;-0,369;0,124;3

-666;-0,182;-0,077;3

-692;-0,088;0,008;3

-747;0,082;0,009;3

-804;-0,025;-0,082;3

-860;0,133;0,116;3

-906;0,502;-0,057;3

-952;0,613;-0,006;3

-989;0,705;0,03;3

-1020;0,602;-0,078;3

-1039;0,133;0,097;3

-1059;-0,571;-0,057;3

-1074;-0,305;-0,029;3

-1091;-0,418;0,053;3

-1107;0,263;-0,053;3

-1120;0,449;0,067;3

-1133;0,244;-0,009;3

-1134;0,452;-0,013;3

-1116;0,197;0,095;3

-1079;-0,082;-0,029;3

-1035;-0,448;-0,005;3

-988;-0,161;-0,005;3

-954;0,23;-0,053;3

-936;-0,158;0,122;3

-940;-0,116;-0,069;3

-956;0,051;-0,006;3

-986;0,013;0,01;3

-1017;0,008;-0,07;3

-1051;0,054;0,092;3

-1079;-0,15;-0,06;3

-1108;-0,434;0,141;3

-1130;-0,084;0,06;3

-1146;0,23;-0,011;3

-1160;-0,111;0,085;3

-1169;-0,239;0,001;3

-1172;-0,117;0,014;3

-1174;-0,419;0,077;3

-1185;-0,358;-0,075;3

-1195;-0,292;0,075;3

-1206;-0,226;0,004;3

-1210;-0,113;-0,028;3

-1190;0,005;0,098;3

-1157;0,155;-0,312;3

-1112;0,275;0,037;3

-1070;0,366;0,005;3

-1021;0,31;-0,041;3

-968;0,426;0,088;3

-908;0,477;-0,065;3

-846;0,377;0,019;3

-780;0,253;0,017;3

-720;0,226;-0,158;3

-662;0,337;0,094;3

-601;0,364;-0,029;3

-546;0,391;0,005;3

-519;0,223;0,048;3

-540;-0,184;-0,069;3

-596;0,315;0,07;3

-677;0,187;0,065;3

-749;0,076;0,002;3

-812;0,072;0,065;3

-861;0,108;-0,081;3

-905;0,046;0,063;3

-941;-0,243;-0,001;3

-974;-0,272;-0,038;3

-996;-0,296;-0,008;3

-1021;-0,059;-0,082;3

-1045;0,202;0,031;3

-1069;0,112;0,052;3

-1091;0,244;-0,139;3

-1112;0,096;0,079;3

-1125;-0,075;-0,083;3

-1142;0,197;-0,007;3

-1156;0,166;0,044;3

-1170;0,205;-0,077;3

-1178;0,019;0,077;3

-1184;-0,11;-0,039;3

-1191;-0,409;-0,03;3

-1194;-0,402;-0,013;3

-1203;-0,322;-0,093;3

-1212;-0,085;0,113;3

-1218;-0,111;-0,047;3

-1207;0,139;-0,011;3

-1175;-0,009;0,014;3

-1138;0,061;-0,06;3

-1088;0,068;0,051;3

-1036;-0,195;0,01;3

-980;0,107;-0,052;3

-920;0,273;0,094;3

-854;0,114;-0,069;3

-788;0,344;0,022;3

-720;0,206;0,027;3

-653;0,463;-0,06;3

-593;0,237;0,078;3

-565;-0,012;0,034;3

-580;0,316;0,006;3

-631;-0,35;0,057;3

-699;-0,598;-0,134;3

-765;-0,715;0,058;3

-824;-0,578;-0,032;3

-873;-0,101;-0,016;3

-914;0,111;0,077;3

-949;0,459;-0,095;3

-980;0,557;0,122;3

-1005;0,63;0,089;3

-1028;0,773;-0,029;3

-1047;0,512;0,086;3

-1069;0,698;-0,08;3

-1083;0,832;-0,051;3

-1100;0,167;0,009;3

-1097;0,293;-0,048;3

-1079;0,069;0,161;3

-1037;-0,003;-0,041;3

-990;-0,043;0,024;3

-939;-0,49;0,036;3

-890;-0,316;-0,036;3

-847;-0,502;0,071;3

-833;-0,331;-0,021;3

-840;0,015;0,019;3

-872;0,355;0,13;3

-911;0,491;-0,093;3

-951;0,552;0,085;3

-987;0,332;-0,011;3

-1017;0,255;-0,014;3

-1045;0,062;0,065;3

-1069;0,107;-0,047;3

-1091;0,242;0,046;3

-1106;-0,395;0;3

-1124;-0,069;0,004;3

-1138;-0,123;0,092;3

-1150;-0,468;-0,055;3

-1161;-0,516;0,059;3

-1173;-0,678;0,017;3

-1182;-0,471;0,061;3

-1178;-0,384;0,086;3

-1155;-0,135;-0,053;3

-1114;0,206;-0,006;3

-1072;-0,272;0,064;3

-1028;0,066;-0,082;3

-979;0,174;0,09;3

-927;0,026;-0,015;3

-873;0,055;-0,019;3

-814;-0,142;0,072;3

-756;0,25;-0,06;3

-696;-0,148;0,062;3

-641;-0,286;0,012;3

-592;-0,102;-0,053;3

-568;-0,536;0,043;3

-583;-0,246;-0,048;3

-638;0,095;0,01;3

-709;0,075;0,014;3

-786;0,007;-0,027;3

-852;-0,221;0,067;3

-908;-0,07;-0,051;3

-950;-0,064;0,022;3

-989;0,126;0,022;3

-1018;0,503;-0,089;3

-1047;0,341;0,074;3

-1065;0,396;0,051;3

-1078;0,652;0,045;3

-1072;0,019;0,055;3

-1046;0,394;-0,144;3

-1009;0,364;0,108;3

-959;-0,046;-0,013;3

-907;0,283;-0,016;3

-851;-0,008;0,058;3

-802;0,125;-0,108;3

-763;-0,03;0,044;3

-738;-0,111;-0,006;3

-734;0,387;-0,099;3

-755;0,421;0,059;3

-798;0,379;-0,036;3

-854;0,341;0,029;3

-913;0,67;-0,005;3

-964;0,618;-0,07;3

-1008;0,459;0,035;3

-1043;0,68;-0,039;3

-1077;0,535;-0,01;3

-1105;0,409;0,036;3

-1132;0,787;-0,096;3

-1149;0,437;0,118;3

-1166;-0,027;-0,043;3

-1178;-0,041;-0,012;3

-1195;-0,233;0,13;3

-1206;-0,389;-0,066;3

-1218;-0,737;0,072;3

-1226;-0,657;-0,041;3

-1236;-0,551;-0,021;3

-1247;-0,488;0,042;3

-1262;-0,112;-0,044;3

-1275;-0,172;0,043;3

-1280;-0,353;-0,005;3

-1273;0,027;-0,043;3

-1253;-0,137;0,099;3

-1218;-0,101;-0,056;3

-1176;0,333;0,007;3

-1138;0,181;0,036;3

-1097;0,09;-0,087;3

-1051;0,046;0,035;3

-998;0,058;-0,036;3

-943;0,206;0,005;3

-886;-0,015;0,083;3

-836;0,458;-0,081;3

-790;0,309;0,03;3

-762;0,463;-0,04;3

-756;0,582;0,005;3

-776;-0,026;0,06;3

-815;-0,066;-0,083;3

-858;0,717;0,036;3

-902;-0,148;-0,022;3

-932;-0,329;-0,013;3

-961;-0,272;0,066;3

-981;-0,457;-0,065;3

-999;-0,289;0,038;3

-1019;-0,261;0,02;3

-1037;0,127;-0,044;3

-1052;-0,233;0,114;3

-1070;-0,229;-0,057;3

-1085;0,139;-0,017;3

-1111;-0,202;0,056;3

-1139;-0,348;-0,073;3

-1169;-0,258;0,103;3

-1198;-0,42;-0,029;3

-1220;-0,276;-0,001;3

-1228;-0,041;0,1;3

-1227;-0,372;-0,079;3

-1209;-0,427;0,086;3

-1176;-0,183;-0,005;3

-1136;-0,054;-0,008;3

-1096;-0,228;0,094;3

-1062;-0,044;-0,067;3

-1029;0,474;0,065;3

-998;0,129;0,012;3

-955;0,036;-0,052;3

-908;-0,027;0,164;3

-868;-0,275;-0,057;3

-842;-0,381;0,037;3

-830;-0,503;0,033;3

-815;-0,549;-0,067;3

-785;-0,285;0,083;3

-758;-0,252;-0,049;3

-751;-0,361;-0,033;3

-778;-0,191;0,02;3

-825;-0,014;-0,103;3

-884;-0,128;0,069;3

-940;0,425;-0,056;3

-988;0,995;0,01;3

-1034;0,726;-0,018;3

-1068;0,417;-0,081;3

-1102;0,418;0,143;3

-1124;0,293;0,004;3

-1142;0,291;0,007;3

-1153;-0,119;0,072;3

-1163;0,105;-0,079;3

-1171;0,52;-0,001;3

-1185;0,507;-0,056;3

-1194;0,651;-0,059;3

-1205;0,232;0,121;3

-1216;0,091;-0,057;3

-1223;-0,031;0,014;3

-1217;-0,306;0,015;3

-1197;-0,155;-0,084;3

-1169;0,019;0,122;3

-1132;-0,148;-0,033;3

-1085;-0,321;0,009;3

-1033;-0,158;0,077;3

-981;0,011;-0,073;3

-925;0,051;0,059;3

-885;0,291;-0,025;3

-864;0,713;-0,022;3

-872;0,743;0,1;3

-897;0,411;-0,04;3

-937;0,229;0,192;3

-978;-0,02;0,029;3

-1015;0,396;-0,057;3

-1043;0,043;0,103;3

-1070;0,223;-0,072;3

-1089;-0,302;0,065;3

-1101;-0,156;0,025;3

-1098;-0,572;-0,065;3

-1081;-0,838;0,082;3

-1056;-0,569;-0,049;3

-1028;-0,62;-0,005;3

-994;-0,342;0,047;3

-959;0,402;-0,087;3

-934;0,282;0,055;3

-920;0,116;-0,031;3

-931;0,221;-0,024;3

-957;0,541;0,063;3

-994;0,719;-0,087;3

-1028;0,568;0,058;3

-1062;0,384;-0,03;3

-1092;0,478;-0,019;3

-1117;0,457;0,089;3

-1137;0,29;-0,069;3

-1147;0,123;0,06;3

-1157;0,245;-0,015;3

-1162;0,29;-0,05;3

-1173;-0,299;0,087;3

-1186;-0,563;-0,087;3

-1202;-0,077;0,023;3

-1210;0,246;0,014;3

-1220;0,465;-0,039;3

-1225;0,105;0,059;3

-1236;0,046;-0,036;3

-1242;0,304;0,013;3

-1248;0,147;0,051;3

-1250;0,198;-0,07;3

-1252;0,217;0,063;3

-1252;-0,001;-0,02;3

-1256;0,153;-0,054;3

-1255;-0,147;0,09;3

-1259;-0,372;-0,086;3

-1262;-0,184;0,051;3

-1267;0,122;0,021;3

-1268;0,302;-0,027;3

-1254;0,168;0,071;3

-1226;0,205;-0,017;3

-1191;0,409;0,039;3

-1150;0,142;0,004;3

-1112;0,566;-0,061;3

-1076;0,326;0,061;3

-1035;-0,168;-0,064;3

-988;-0,192;0,075;3

-933;-0,39;0,066;3

-879;-0,131;-0,069;3

-822;-0,298;0,074;3

-760;-0,135;-0,059;3

-705;-0,018;-0,006;3

-648;-0,18;0,069;3

-609;-0,04;-0,061;3

-604;-0,108;0,054;3

-645;0,201;0,002;3

-710;0,215;-0,024;3

-779;0,12;0,078;3

-841;0,019;-0,146;3

-886;-0,369;0,161;3

-920;-0,604;0,015;3

-947;-0,487;-0,061;3

-970;-0,326;0,082;3

-989;-0,569;-0,069;3

-1012;-0,53;0,008;3

-1033;-0,477;0,007;3

-1060;0,047;-0,077;3

-1076;0,059;0,098;3

-1072;-0,404;-0,054;3

-1038;-0,269;0,005;3

-993;-0,09;0,05;3

-939;0,102;-0,082;3

-882;0,298;-0,007;3

-827;-0,122;-0,016;3

-779;-0,125;-0,004;3

-734;-0,233;0,085;3

-697;-0,004;-0,079;3

-669;0,21;-0;3

-665;-0,009;0,051;3

-691;-0,119;-0,013;3

-738;-0,146;0,082;3

-795;0,042;-0,06;3

-849;0,031;0,058;3

-896;0,005;0,018;3

-937;0,43;-0,069;3

-976;0,293;0,086;3

-1010;0,621;-0,056;3

-1042;0,29;0,04;3

-1067;-0,118;0,035;3

-1089;0,021;-0,075;3

-1106;-0,173;0,072;3

-1117;-0,174;-0,025;3

-1133;-0,29;0,007;3

-1147;-0,466;0,045;3

-1157;-0,439;-0,077;3

-1148;-0,433;0,068;3

-1128;-0,274;-0,011;3

-1101;-0,118;0,088;3

-1070;-0,186;0,093;3

-1027;-0,008;-0,075;3

-968;-0,046;0,085;3

-898;-0,298;0,016;3

-826;0,042;-0,005;3

-772;-0,321;0,088;3

-746;-0,32;-0,06;3

-764;-0,39;-0,001;3

-805;-0,089;0,005;3

-858;-0,118;-0,074;3

-909;-0,179;0,09;3

-954;-0,562;-0,067;3

-995;-0,681;0,003;3

-1032;-0,138;0,032;3

-1059;0,175;-0,078;3

-1085;-0,047;0,083;3

-1104;0,418;-0,046;3

-1117;0,603;0,066;3

-1095;0,142;0,06;3

-1042;0,088;-0,064;3

-972;0,237;0,077;3

-903;0,131;-0,002;3

-834;0,172;0,002;3

-781;-0,118;0,08;3

-748;-0,117;0,094;3

-750;-0,137;0,01;3

-784;-0,17;0,019;3

-835;0,007;-0,061;3

-887;-0,156;0,054;3

-934;0,038;-0,051;3

-976;0,249;0,04;3

-1007;-0,058;0,006;3

-1037;0,219;-0,089;3

-1065;0,176;0,056;3

-1092;0,022;-0,028;3

-1112;0,06;0,01;3

-1129;-0,15;0,01;3

-1146;-0,157;-0,103;3

-1162;-0,087;0,026;3

-1178;0,141;-0,032;3

-1182;0,27;-0,015;3

-1164;0,123;0,073;3

-1115;0,018;-0,089;3

-1056;-0,355;0,059;3

-986;-0,41;0,032;3

-919;0,061;-0,034;3

-865;-0,135;0,092;3

-843;0,086;-0,053;3

-850;0,43;0,036;3

-885;0,027;0,106;3

-932;0,178;-0,058;3

-980;0,013;0,094;3

-1019;-0,208;-0,048;3

-1054;-0,201;0,016;3

-1083;-0,205;-0,017;3

-1083;-0,288;-0,06;3

-1043;-0,229;0,07;3

-970;-0,008;-0,051;3

-880;0,117;-0,015;3

-783;-0,278;0,067;3

-694;-0,38;-0,087;3

-633;-0,472;0,044;3

-627;-0,481;-0,012;3

-666;-0,117;-0,025;3

-733;-0,396;0,065;3

-801;-0,127;-0,057;3

-865;-0,172;0,046;3

-912;-0,325;0,015;3

-954;0,046;-0,05;3

-989;0,066;0,098;3

-1022;-0,146;-0,045;3

-1041;-0,105;0,047;3

-1037;-0,117;0,013;3

-999;0,42;-0,09;3

-943;0,226;0,002;3

-887;-0,051;-0,06;3

-831;0,274;-0,022;3

-790;0,149;0,061;3

-752;0,04;-0,098;3

-722;-0,016;0,119;3

-706;-0,236;-0,057;3

-721;-0,151;-0,02;3

-768;-0,089;0,073;3

-830;-0,229;-0,077;3

-895;-0,242;0,177;3

-948;-0,326;-0,03;3

-994;0,106;-0,084;3

-1034;0,213;0,1;3

-1067;0,254;-0,077;3

-1093;0,098;0,011;3

-1120;0,006;0,099;3

-1135;0,11;-0,054;3

-1156;0,533;0,139;3

-1175;0,673;-0,358;3

-1198;0,344;0,019;3

-1215;0,191;-0,025;3

-1227;0,382;-0,033;3

-1226;0,029;0,059;3

-1213;0,217;-0,03;3

-1183;0,127;0,305;3

-1147;-0,275;0,064;3

-1100;-0,183;-0,084;3

-1047;-0,294;0,085;3

-986;-0,262;-0,015;3

-922;-0,192;-0,019;3

-859;-0,177;0,052;3

-801;-0,363;-0,07;3

-752;-0,339;0,114;3

-722;-0,083;-0,025;3

-727;0,328;-0,041;3

-760;0,534;0,085;3

-811;0,664;-0,093;3

-852;0,54;0,033;3

-890;-0,029;-0,01;3

-920;-0,444;-0,071;3

-949;-0,128;0,081;3

-975;-0,282;-0,047;3

-998;-0,402;0,024;3

-1022;-0,395;0,05;3

-1040;-0,227;-0,137;3

-1060;-0,397;0,079;3

-1081;-0,427;-0,044;3

-1104;-0,478;0,047;3

-1119;-0,505;0,119;3

-1111;-0,231;-0,069;3

-1078;-0,353;0,086;3

-1030;-0,469;-0,025;3

-975;-0,251;-0,036;3

-918;-0,155;0,07;3

-870;-0,095;-0,079;3

-844;0,093;0,024;3

-850;0,115;-0,042;3

-884;0,048;-0,071;3

-937;-0,358;0,128;3

-990;-0,29;-0,058;3

-1037;-0,364;0,003;3

-1075;-0,199;0,03;3

-1109;-0,095;-0,031;3

-1134;-0,182;0,061;3

-1159;-0,108;-0,064;3

-1174;0,086;0,024;3

-1166;0,03;0,039;3

-1128;-0,127;-0,057;3

-1072;-0,187;0,077;3

-996;0,132;-0,05;3

-914;0,23;0,104;3

-832;0,162;0,079;3

-770;0,291;-0,084;3

-742;0,092;0,05;3

-766;-0,22;-0,002;3

-816;0,17;-0,045;3

-880;0,009;0,04;3

-938;-0,291;-0,059;3

-985;-0,512;-0,017;3

-1027;-0,665;-0,01;3

-1058;-0,412;-0,094;3

-1085;-0,473;0,086;3

-1105;-0,385;-0,061;3

-1120;-0,421;0,004;3

-1108;-0,418;0,025;3

-1073;-0,181;-0,065;3

-1017;0,36;0,103;3

-952;0,786;-0,041;3

-885;0,877;-0,008;3

-816;0,56;0,054;3

-771;0,238;-0,155;3

-760;0,132;0,031;3

-790;0,272;-0,026;3

-834;0,616;-0,018;3

-884;0,206;0,076;3

-932;0,317;-0,052;3

-976;-0,03;0,067;3

-1015;-0,075;0,081;3

-1048;0,117;-0,034;3

-1075;-0,296;0,08;3

-1102;-0,459;-0,05;3

-1125;-0,227;-0,004;3

-1146;-0,309;0,048;3

-1164;0,109;-0,07;3

-1177;-0,246;0,102;3

-1190;-0,132;-0,126;3

-1201;0,661;0,004;3

-1204;0,324;0,028;3

-1181;0,25;-0,067;3

-1135;0,387;0,091;3

-1077;0,397;-0,024;3

-1016;0,089;0,031;3

-947;0,126;0,04;3

-879;0,163;-0,067;3

-810;0,069;0,068;3

-757;-0,026;0,018;3

-712;0,015;-0,016;3

-692;-0,006;0,085;3

-697;-0,057;-0,076;3

-732;0,286;0,033;3

-783;0,2;-0,014;3

-838;0,195;-0,043;3

-891;-0,033;0,096;3

-937;0,019;-0,061;3

-979;-0,047;-0,012;3

-1015;-0,11;0,045;3

-1048;0,254;-0,07;3

-1077;0,105;0,067;3

-1099;-0,015;0,027;3

-1118;0,362;0,091;3

-1109;-0,119;0,069;3

-1075;0,143;-0,063;3

-1015;0,336;0,059;3

-953;-0,029;-0,003;3

-897;0,354;-0,034;3

-860;-0,006;0,087;3

-850;-0,252;-0,089;3

-870;-0,116;0,071;3

-906;-0,23;-0,016;3

-950;0,179;-0,042;3

-991;-0,011;0,081;3

-1026;-0,136;-0,072;3

-1062;-0,314;-0,015;3

-1090;-0,427;-0,002;3

-1122;-0,022;-0,076;3

-1144;0,544;0,035;3

-1162;0,601;-0,067;3

-1172;0,492;-0,006;3

-1181;0,489;0,027;3

-1190;0,327;-0,099;3

-1208;0,509;0,062;3

-1220;0,379;-0,036;3

-1220;0,68;0,001;3

-1188;0,574;0,061;3

-1132;0,417;-0,089;3

-1068;0,228;0,083;3

-1001;-0,003;-0,017;3

-932;-0,067;-0,002;3

-857;0,01;0,05;3

-784;0,476;-0,069;3

-712;0,844;0,047;3

-649;0,372;0,018;3

-604;0,651;-0,025;3

-586;0,258;0,126;3

-604;-0,067;-0,05;3

-653;0,207;0,016;3

-720;0,166;0,014;3

-785;0,277;-0,086;3

-847;0,102;0,081;3

-897;0,209;-0,076;3

-940;0,352;0,001;3

-973;0,413;0,058;3

-1002;0,52;-0,09;3

-1028;0,213;0,073;3

-1049;-0,036;-0,031;3

-1069;0,211;0,002;3

-1090;-0,059;0,071;3

-1116;0,107;-0,087;3

-1140;-0,171;0,054;3

-1163;-0,268;0,011;3

-1173;-0,155;-0,086;3

-1172;-0,482;0,109;3

-1146;-0,251;-0,073;3

-1100;-0,071;0,025;3

-1036;0,275;0,006;3

-971;0,312;-0,076;3

-902;-0,154;0,076;3

-846;0,167;-0,05;3

-814;0,236;-0,014;3

-816;0,223;0,028;3

-843;0,134;-0,171;3

-891;-0,098;0,052;3

-937;-0,203;-0,027;3

-979;0,306;-0,011;3

-1020;0,225;0,065;3

-1054;0,015;-0,066;3

-1087;0,178;0,066;3

-1111;-0,177;-0,003;3

-1132;-0,255;-0,025;3

-1141;-0,47;0,053;3

-1131;0,077;-0,043;3

-1084;-0,096;0,052;3

-1017;-0,207;-0,002;3

-948;0,033;-0,063;3

-893;-0,088;0,091;3

-866;-0,046;0,024;3

-875;-0,034;0,023;3

-910;0,034;0,011;3

-953;0,172;-0,068;3

-998;-0,219;0,087;3

-1035;-0,326;-0,034;3

-1065;0,365;0,04;3

-1088;0,399;0,04;3

-1107;-0,126;-0,094;3

-1122;0,052;0,072;3

-1140;0,09;-0,023;3

-1153;0,194;0,021;3

-1144;0,314;0,056;3

-1104;0,251;-0,086;3

-1047;0,173;0,055;3

-980;-0,198;0,001;3

-902;-0,126;-0,031;3

-830;-0,207;0,067;3

-772;-0,095;-0,074;3

-751;0,227;0,039;3

-760;0,651;-0,007;3

-801;0,488;-0,099;3

-849;0,202;0,082;3

-898;0,533;-0,024;3

-940;0,715;0,03;3

-980;0,224;0,054;3

-1012;0,304;-0,078;3

-1045;0,296;0,099;3

-1070;0,089;-0,062;3

-1091;0,175;-0,002;3

-1109;0,263;0,019;3

-1125;0,319;-0,064;3

-1131;0,251;0,099;3

-1119;0,056;0,005;3

-1082;0,146;-0,027;3

-1037;-0,074;0,041;3

-982;0,046;-0,07;3

-926;-0,124;0,061;3

-861;-0,702;-0,082;3

-799;-0,483;-0,081;3

-753;-0,13;0,062;3

-729;0,069;-0,038;3

-745;0,079;0,028;3

-783;0,291;-0,013;3

-836;0,624;-0,062;3

-889;0,275;0,082;3

-936;0,044;-0,074;3

-972;-0,109;0,006;3

-998;-0,039;0,085;3

-995;-0,023;-0,074;3

-960;0,16;0,047;3

-906;-0,13;-0,087;3

-838;-0,013;-0;3

-767;0,163;0,063;3

-704;0,195;-0,086;3

-683;0,124;0,079;3

-702;0,114;-0,012;3

-752;0,074;-0,019;3

-809;0,255;0,084;3

-866;0,884;-0,067;3

-915;1,14;0,045;3

-971;0,535;0,036;3

-1017;0,681;-0,053;3

-1053;0,459;0,112;3

-1045;0,128;0,017;3

-987;0,223;0,011;3

-900;-0,122;-0;3

-817;-0,076;-0,071;3

-755;-0,165;0,069;3

-740;-0,184;-0,041;3

-760;-0,001;-0,033;3

-810;-0,258;0,031;3

-862;-0,386;-0,075;3

-913;-0,209;0,071;3

-958;-0,232;0,028;3

-998;-0,018;-0;3

-1034;-0,096;0,039;3

-1072;-0,105;-0,1;3

-1100;0,104;0,086;3

-1100;0,394;0,023;3

-1055;0,274;-0,06;3

-978;-0,012;0,087;3

-880;0,025;-0,039;3

-784;0,025;0,036;3

-724;-0,179;0,032;3

-712;0,07;-0,064;3

-749;-0,128;0,085;3

-817;0,114;-0,06;3

-888;0,246;0,122;3

-948;-0,461;0,043;3

-992;-0,281;-0,028;3

-1028;-0,019;0,09;3

-1064;-0,096;-0,043;3

-1099;-0,144;0,014;3

-1125;-0,04;0,086;3

-1149;-0,045;-0,116;3

-1162;0,178;0,043;3

-1178;0,236;-0,015;3

-1176;0,442;0,028;3

-1154;0,311;0,078;3

-1102;0,485;-0,073;3

-1043;0,469;0,028;3

-976;0,331;-0,001;3

-907;0,532;-0,032;3

-836;0,191;0,089;3

-766;0,207;-0,051;3

-689;0,024;-0,007;3

-645;-0,191;0,01;3

-646;-0,157;-0,071;3

-690;-0,445;0,078;3

-758;-0,223;-0,069;3

-829;0,071;-0,027;3

-888;-0,221;0,06;3

-942;-0,135;-0,006;3

-988;0,016;0,059;3

-1030;-0,135;-0,056;3

-1065;0,08;-0,007;3

-1096;-0,085;0,052;3

-1118;-0,244;-0,079;3

-1134;-0,133;0,086;3

-1150;-0,425;-0,001;3

-1167;0,173;-0,009;3

-1188;0,042;0,072;3

-1193;-0,136;-0,049;3

-1177;-0,189;0,032;3

-1141;-0,604;0,015;3

-1092;-0,643;-0,103;3

-1043;-0,497;0,088;3

-985;-0,251;-0,046;3

-926;0,117;0,002;3

-865;0,214;0,017;3

-807;0,422;-0,062;3

-752;0,313;0,04;3

-719;0,287;-0,033;3

-701;0,306;0,007;3

-714;0,276;0,063;3

-749;0,177;-0,093;3

-806;0,462;0,067;3

-861;0,081;-0,002;3

-918;-0,486;-0,01;3

-966;-0,322;0,076;3

-1011;-0,031;-0,078;3

-1046;-0,267;0,066;3

-1078;-0,513;0,023;3

-1099;-0,142;-0,025;3

-1114;-0,103;0,083;3

-1127;0,013;-0,065;3

-1116;0,104;0,063;3

-1082;0,228;0,01;3

-1028;0,39;-0,055;3

-963;0,483;0,09;3

-886;0,391;-0,072;3

-807;0,378;-0,014;3

-745;0,171;0,049;3

-723;0,461;-0,082;3

-737;0,156;0,067;3

-781;-0,076;-0,013;3

-841;0,276;0,009;3

-902;0,062;0,101;3

-958;-0,07;-0,081;3

-1004;-0,107;0,075;3

-1040;-0,061;-0,046;3

-1069;-0,002;-0,032;3

-1093;-0,26;0,024;3

-1098;-0,298;-0,035;3

-1066;-0,126;0,047;3

-1003;-0,03;0,07;3

-925;0,037;-0,05;3

-833;0,165;0,089;3

-749;0,622;0,015;3

-684;0,705;0,013;3

-668;0,486;0,024;3

-699;0,306;-0,089;3

-768;0,414;0,11;3

-846;0,346;-0,032;3

-919;0,167;-0,015;3

-979;0,231;0,04;3

-1029;0,374;-0,061;3

-1066;0,174;0,13;3

-1091;0,141;-0,025;3

-1115;0,074;-0,011;3

-1128;0,023;0,092;3

-1127;0,277;-0,072;3

-1090;0,006;0,082;3

-1034;-0,156;-0,075;3

-967;0,05;-0,003;3

-897;-0,135;0,089;3

-823;-0,053;-0,07;3

-768;-0,108;-0,055;3

-746;-0,122;0,035;3

-772;0,054;0,013;3

-823;0,121;0,076;3

-890;0,18;-0,085;3

-949;0,229;-0,041;3

-1000;0,184;0,019;3

-1039;0,043;-0,093;3

-1077;0,205;0,06;3

-1106;0,365;-0,037;3

-1134;0,393;-0,003;3

-1158;0,599;0,044;3

-1182;0,533;-0,038;3

-1202;0,702;0,09;3

-1223;0,691;-0,034;3

-1242;0,638;-0,014;3

-1257;0,514;0,075;3

-1270;0,571;-0,065;3

-1270;0,52;0,038;3

-1277;0,359;0,022;3

-1283;0,049;-0,087;3

-1298;-0,193;0,088;3

-1313;0,1;-0,079;3

-1320;0,095;0,03;3

-1321;-0,216;-0,004;3

-1319;-0,338;-0,126;3

-1323;-0,07;0,111;3

-1326;0,125;-0,059;3

-1331;0,061;0,015;3

-1319;0,06;0,081;3

-1280;0,287;-0,088;3

-1211;0,474;0,091;3

-1133;0,164;-0,043;3

-1048;0,1;0,009;3

-968;0,051;0,069;3

-892;0,186;-0,056;3

-836;0,155;0,061;3

-809;0,137;0,012;3

-824;0,346;-0,011;3

-860;0,136;0,074;3

-906;0,066;-0,049;3

-943;-0,056;0,004;3

-982;-0,103;0,015;3

-1015;0,005;-0,066;3

-1048;0,079;0,12;3

-1075;0,061;-0,077;3

-1099;0,122;0,014;3

-1116;0,083;0,028;3

-1123;0,314;-0,077;3

-1100;0,454;0,068;3

-1053;0,368;-0,022;3

-994;0,054;-0,019;3

-943;-0,104;0,036;3

-889;-0,163;-0,069;3

-845;-0,236;0,061;3

-824;-0,403;-0,039;3

-830;-0;0,043;3

-866;-0,074;0,106;3

-912;-0,522;-0,091;3

-959;-1,024;0,053;3

-996;-0,987;0,019;3

-1031;-0,585;-0,042;3

-1062;-0,823;0,201;3

-1086;-0,669;-0,057;3

-1098;-0,49;0,052;3

-1073;-0,329;0,017;3

-1012;-0,059;-0,074;3

-930;-0,152;0,113;3

-847;-0,027;-0,076;3

-759;-0,016;-0,024;3

-685;-0,286;0,007;3

-627;-0,139;-0,067;3

-608;-0,073;0,109;3

-636;-0,305;0,004;3

-703;-0,184;-0,015;3

-777;-0,458;0,064;3

-844;-0,373;-0,1;3

-896;-0,375;0,08;3

-940;-0,514;0,022;3

-973;-0,207;-0,015;3

-1006;-0,345;0,08;3

-1033;-0,184;-0,034;3

-1058;-0,035;0,081;3

-1074;-0,079;0,008;3

-1093;-0,166;-0,06;3

-1095;-0,263;0,087;3

-1069;0,024;-0,049;3

-1021;0,049;0,186;3

-956;0,068;0,059;3

-877;0,391;-0,078;3

-810;0,468;0,066;3

-768;0,296;-0,054;3

-766;0,377;-0,004;3

-791;0,154;0,095;3

-834;0,09;-0,053;3

-882;-0,158;0,052;3

-927;-0,248;-0,009;3

-973;-0,14;0,034;3

-1014;-0,195;0,024;3

-1050;0,039;-0,081;3

-1077;0,341;0,049;3

-1102;0,139;0,001;3

-1119;0,075;-0,029;3

-1124;-0,067;0,116;3

-1099;-0,141;-0,052;3

-1054;0,054;0,037;3

-1000;0,139;0,003;3

-944;0,098;-0,077;3

-904;0,066;0,069;3

-889;-0,044;-0,055;3

-902;-0,483;0,009;3

-932;-0,442;0,033;3

-967;-0,062;-0,123;3

-1002;-0,42;0,07;3

-1037;-0,345;-0,028;3

-1063;0,17;-0,002;3

-1095;-0,083;0,103;3

-1117;-0,082;-0,086;3

-1136;0,424;0,081;3

-1141;0,355;-0;3

-1116;0,186;-0,034;3

-1058;-0,15;0,082;3

-994;0,071;-0,074;3

-932;0,406;0,135;3

-875;0,405;-0,026;3

-819;0,133;-0,048;3

-791;-0,124;0,036;3

-790;-0,238;-0,079;3

-822;-0,074;0,009;3

-868;-0,1;0,005;3

-917;-0,234;-0,069;3

-959;-0,661;0,096;3

-999;-0,323;-0,05;3

-1034;-0,117;-0,011;3

-1066;-0,565;0,033;3

-1087;-0,228;-0,07;3

-1083;-0,191;0,057;3

-1049;-0,371;-0,03;3

-990;0,086;-0,008;3

-932;-0,353;0,066;3

-873;-0,325;-0,089;3

-809;-0,07;0,066;3

-760;0,122;0,001;3

-743;0,211;0,029;3

-771;-0,082;0,089;3

-822;0,171;-0,087;3

-882;0,45;0,035;3

-933;0,258;0,012;3

-975;0,154;-0,078;3

-1008;0,282;0,199;3

-1044;0,168;-0,055;3

-1067;0,015;0,048;3

-1089;-0,008;0,022;3

-1104;0,115;-0,091;3

-1120;0,09;0,093;3

-1139;0,234;-0,051;3

-1158;-0,001;0,011;3

-1178;-0,458;0,066;3

-1176;-0,35;-0,069;3

-1144;-0,078;0,078;3

-1085;-0,186;0,003;3

-1016;-0,122;-0,038;3

-933;-0,6;0,061;3

-848;-0,519;-0,07;3

-756;-0,358;0,06;3

-680;-0,408;-0,003;3

-625;-0,248;-0,046;3

-619;-0,51;0,073;3

-651;-0,226;-0,016;3

-714;-0,057;0,057;3

-779;-0,1;0,007;3

-838;0,257;-0,053;3

-885;0,44;0,076;3

-930;0,097;-0,054;3

-963;0,221;0,053;3

-995;0,057;0,058;3

-1020;0,127;-0,082;3

-1039;0,213;0,091;3

-1062;-0,126;-0,014;3

-1085;0,212;-0,001;3

-1111;0,083;0,04;3

-1135;-0,147;-0,124;3

-1150;-0,274;0,054;3

-1153;-0,081;0,011;3

-1127;-0,191;-0,053;3

-1081;-0,479;0,054;3

-1022;-0,454;-0,063;3

-967;-0,573;0,032;3

-910;-0,576;-0,002;3

-850;-0,285;-0,052;3

-780;-0,248;0,099;3

-713;0,002;-0,076;3

-658;0,343;0,006;3

-641;0,391;0,01;3

-658;0,313;-0,013;3

-698;0,177;0,084;3

-741;-0,15;-0,047;3

-788;-0,07;-0,001;3

-829;-0,246;0,065;3

-873;-0,239;-0,124;3

-911;-0,08;0,08;3

-941;-0,06;-0,009;3

-966;0,141;-0,01;3

-988;-0,353;0,039;3

-998;-0,486;-0,053;3

-971;-0,111;0,11;3

-911;-0,041;0,018;3

-844;0,085;-0,049;3

-797;-0,32;0,105;3

-785;0,302;-0,055;3

-810;0,499;-0,02;3

-859;0,078;-0,046;3

-915;-0,106;-0,075;3

-964;-0,197;0,076;3

-1011;0,066;-0,056;3

-1045;-0,264;0,064;3

-1077;-0,578;0,033;3

-1100;-0,069;-0,101;3

-1120;0,088;0,066;3

-1136;0,028;-0,034;3

-1152;-0,059;-0,013;3

-1156;-0,253;0,05;3

-1125;-0,171;-0,075;3

-1074;-0,228;0,052;3

-1003;-0,111;0,063;3

-929;-0,106;0,025;3

-853;0,219;0,076;3

-782;0,084;-0,069;3

-710;0,221;0,015;3

-655;-0,076;0,022;3

-636;0,42;-0,034;3

-660;0,2;0,079;3

-717;-0,087;-0,074;3

-786;-0,09;0,016;3

-854;-0,086;0,024;3

-915;-0,092;-0,045;3

-966;-0,163;0,049;3

-1008;-0,452;-0,047;3

-1048;0,038;-0,011;3

-1077;0,123;0,022;3

-1105;0,202;-0,061;3

-1124;0,072;0,063;3

-1139;-0,192;-0,019;3

-1147;0,186;-0,005;3

-1159;0,151;0,041;3

-1173;0,084;-0,095;3

-1194;-0,063;0,072;3

-1212;0,143;-0,011;3

-1230;0,322;-0,015;3

-1239;-0,046;0,195;3

-1249;-0,084;-0,065;3

-1252;0,002;0,053;3

-1253;0,189;0,021;3

-1235;0,347;-0,057;3

-1199;0,101;0,081;3

-1149;-0,09;-0,061;3

-1103;-0,009;0,02;3

-1050;-0,092;0,036;3

-999;-0,183;-0,039;3

-948;-0,387;0,075;3

-901;-0,327;-0,028;3

-857;-0,025;-0,016;3

-806;-0,419;-0,018;3

-759;-0,137;-0,081;3

-713;-0,281;0,08;3

-670;-0,687;-0,019;3

-641;0,334;0,007;3

-639;0,306;0,065;3

-673;0,153;-0,051;3

-728;0,076;0,045;3

-794;-0,068;-0,014;3

-856;0,041;-0,042;3

-914;-0,009;0,099;3

-961;0,031;-0,048;3

-1001;0,063;0,035;3

-1030;0,119;-0,069;3

-1054;0,656;-0,071;3

-1075;0,388;0,105;3

-1095;0,243;-0,069;3

-1122;0,285;-0,042;3

-1144;0,083;0,04;3

-1163;0,258;-0,058;3

-1177;-0,194;0,057;3

-1192;-0,225;-0,046;3

-1200;0,07;-0,014;3

-1208;0,09;0,076;3

-1213;0,247;-0,071;3

-1221;0,19;0,07;3

-1221;-0,148;0,002;3

-1221;0,166;-0,083;3

-1206;0,262;0,079;3

-1186;0,194;-0,12;3

-1154;-0,093;0,079;3

-1124;0,121;0,002;3

-1086;0,632;-0,035;3

-1044;0,43;0,095;3

-996;0,124;-0,077;3

-944;0,316;0,001;3

-894;0,306;0,035;3

-843;0,217;-0,079;3

-798;0,362;0,067;3

-757;0,31;-0,049;3

-732;0,055;0,03;3

-731;-0,255;0,047;3

-752;-0,302;-0,083;3

-794;-0,033;0,107;3

-845;0,088;-0,01;3

-900;-0,351;-0;3

-949;-0,399;0,06;3

-992;0,045;-0,074;3

-1026;-0,16;0,051;3

-1057;-0,05;0,014;3

-1082;0,077;-0,045;3

-1100;0,1;0,086;3

-1112;0,122;-0,03;3

-1128;-0,021;0,03;3

-1145;-0,065;-0,027;3

-1164;0,46;-0,078;3

-1183;0,014;0,034;3

-1195;-0,103;-0,059;3

-1203;0,013;0,011;3

-1194;-0,072;0,019;3

-1172;0,167;-0,073;3

-1136;-0,299;0,082;3

-1097;0,089;0,015;3

-1059;0,251;0,031;3

-1029;-0,118;0,069;3

-1003;0,235;-0,064;3

-977;0,109;0,069;3

-940;-0,094;-0,03;3

-890;0,036;-0,066;3

-822;-0,085;0,087;3

-754;-0,417;-0,062;3

-688;-0,051;0,036;3

-644;-0,237;-0,038;3

-627;-0,331;-0,046;3

-652;-0,5;0,088;3

-701;-0,191;-0,072;3

-764;0,095;0,049;3

-821;-0,784;0;3

-867;-0,793;-0,059;3

-909;-0,376;0,079;3

-949;-0,093;-0,016;3

-991;-0,01;0,11;3

-1032;-0,49;0,048;3

-1066;-0,456;-0,102;3

-1090;-0,036;0,033;3

-1103;0,308;-0,028;3

-1112;0,22;-0,037;3

-1118;0,054;0,06;3

-1132;-0,034;-0,043;3

-1147;-0,323;0,077;3

-1167;-0,404;-0,051;3

-1183;-0,29;-0,021;3

-1199;-0,1;0,076;3

-1211;-0,116;-0,078;3

-1221;-0,125;0,062;3

-1227;-0,622;0,027;3

-1232;-0,264;-0,059;3

-1226;-0,307;0,101;3

-1214;-0,197;-0,065;3

-1194;-0,17;0,003;3

-1167;-0,439;0,031;3

-1128;-0,146;-0,078;3

-1090;-0,137;0,081;3

-1053;-0,212;-0,043;3

-1016;-0,143;-0,004;3

-981;-0,036;0,085;3

-944;-0,099;-0,052;3

-901;-0,11;0,093;3

-857;-0,608;-0,053;3

-809;-0,47;0,009;3

-755;-0,43;0,063;3

-704;-0,779;-0,079;3

-669;-0,822;0,056;3

-660;-0,58;-0,02;3

-682;-0,519;-0,021;3

-733;-0,436;0,068;3

-792;-0,392;-0,079;3

-851;-0,553;0,037;3

-903;-0,523;0,008;3

-950;-0,06;-0,056;3

-989;0,099;0,131;3

-1028;0,255;-0,051;3

-1062;0,279;-0,006;3

-1091;0,102;0,032;3

-1120;0,193;-0,072;3

-1138;-0,182;0,096;3

-1159;-0,282;-0,025;3

-1174;-0,062;-0,003;3

-1189;0,039;0,086;3

-1204;-0,496;-0,066;3

-1218;-0,701;0,067;3

-1233;-0,396;-0,011;3

-1244;-0,054;0,008;3

-1261;0,03;0,133;3

-1267;0,054;-0,063;3

-1274;0,104;0,048;3

-1280;-0,179;-0,026;3

-1287;0,393;-0,046;3

-1289;0,254;0,08;3

-1288;0,251;-0,058;3

-1269;0,263;0,021;3

-1248;0,18;0,035;3

-1224;0,08;-0,046;3

-1203;-0,063;0,101;3

-1180;-0,082;-0,057;3

-1157;-0,199;-0,015;3

-1128;-0,261;0,054;3

-1099;-0,074;-0,076;3

-1063;0,122;0,063;3

-1024;-0,345;-0,035;3

-976;-0,331;-0,007;3

-923;-0,394;0,068;3

-866;-0,431;-0,03;3

-812;-0,293;0,022;3

-771;-0,147;-0,01;3

-730;-0,415;-0,032;3

-695;-0,254;0,077;3

-670;0,002;-0,089;3

-672;-0,179;0,032;3

-702;-0,175;0,006;3

-754;-0,408;-0,058;3

-811;-0,863;0,076;3

-868;-0,787;-0,053;3

-915;-0,662;-0,004;3

-957;-0,376;0,024;3

-994;0,225;-0,076;3

-1021;0,32;0,099;3

-1049;0,022;-0,041;3

-1068;0,28;-0,069;3

-1092;0,241;0,092;3

-1115;0,453;-0,095;3

-1141;0,356;0,201;3

-1162;0,17;-0,014;3

-1178;0,15;-0,017;3

-1187;0,087;0,087;3

-1194;0,147;-0,126;3

-1204;0,358;0,092;3

-1214;0,142;0,028;3

-1226;0,398;-0,027;3

-1236;0,367;0,087;3

-1248;-0,093;-0,071;3

-1251;-0,049;0,026;3

-1248;-0,149;0,014;3

-1234;-0,128;-0,05;3

-1210;-0,074;0,004;3

-1183;-0,021;-0,059;3

-1152;0,199;-0,016;3

-1121;0,051;0,046;3

-1087;0,044;-0,08;3

-1040;0,211;0,048;3

-989;0,045;-0,042;3

-933;-0,009;-0,008;3

-876;-0,113;0,066;3

-821;-0,461;-0,081;3

-774;-0,419;0,039;3

-735;-0,381;-0,001;3

-707;-0,689;-0,029;3

-689;-0,907;0,078;3

-696;-0,65;-0,099;3

-714;-0,809;0,04;3

-752;-1,096;-0,01;3

-798;-0,696;-0,037;3

-850;-0,788;0,101;3

-898;-0,334;-0,054;3

-942;0,136;-0,002;3

-981;-0,281;0,029;3

-1011;-0,283;-0,087;3

-1042;-0,049;0,111;3

-1071;-0,218;-0,021;3

-1097;-0,543;0,035;3

-1118;-0,667;0,043;3

-1135;-0,366;-0,076;3

-1149;-0,193;0,068;3

-1164;-0,282;0,03;3

-1179;-0,017;-0,009;3

-1190;-0,128;0,079;3

-1197;0,084;-0,048;3

-1200;-0,063;0,029;3

-1208;-0,208;-0,008;3

-1215;0,178;-0,04;3

-1227;0,019;0,078;3

-1235;0,064;-0,068;3

-1245;-0,04;0,108;3

-1249;-0,213;0,015;3

-1259;0,064;-0,051;3

-1260;0,05;0,092;3

-1262;-0,25;-0,066;3

-1252;-0,005;0,019;3

-1229;-0,134;0,032;3

-1193;0,198;-0,097;3

-1157;0,068;0,063;3

-1119;-0,645;-0,028;3

-1082;-0,005;-0,004;3

-1036;-0,232;0,061;3

-988;0,019;-0,075;3

-937;-0,001;0,04;3

-890;-0,521;-0,021;3

-839;-0,665;0,001;3

-786;-0,77;0,064;3

-731;-0,245;-0,057;3

-682;-0,074;0,03;3

-647;-0,48;-0,002;3

-629;-0,008;-0,043;3

-642;-0,193;0,07;3

-670;-0,06;-0,058;3

-723;0,164;0,021;3

-783;-0,048;0,057;3

-845;0,318;-0,07;3

-901;0,411;0,061;3

-940;0,311;-0,081;3

-974;0,525;0,022;3

-1000;0,313;0,035;3

-1031;0,328;-0,077;3

-1058;0,398;0,063;3

-1082;0,28;-0,005;3

-1100;0,623;-0,031;3

-1117;0,368;0,037;3

-1130;0,238;-0,083;3

-1143;0,221;0,066;3

-1154;0,254;0,001;3

-1170;0,304;-0,018;3

-1186;0,059;0,07;3

-1203;0,224;-0,065;3

-1219;0,219;0,046;3

-1226;-0,047;0,002;3

-1230;0,033;-0,045;3

-1222;-0,562;0,152;3

-1204;-0,47;-0,038;3

-1177;-0,024;0,022;1

-1148;-0,112;0,051;1

-1113;-0,31;-0,075;1

-1076;-0,313;0,103;1

-1033;-0,389;-0,01;1

-998;-0,325;0,085;1

-960;-0,164;0,04;1

-927;-0,04;-0,083;1

-892;-0,221;0,064;1

-854;-0,337;-0,024;1

-810;-0,023;0,006;1

-767;-0,175;0,157;1

-713;-0,012;-0,044;1

-656;0,103;0,064;1

-603;-0,062;-0,033;1

-569;-0,026;0,01;1

-573;0;0,128;1

-613;0,066;-0,104;1

-682;-0,1;-0,043;1

-750;-0,215;0,066;1

-815;0,062;-0,074;1

-872;0,135;0,09;1

-919;0,044;-0,057;1

-961;-0,051;0,013;1

-990;-0,055;0,052;1

-1014;0,285;-0,07;1

-1034;0,244;0,111;1

-1060;0,176;-0,016;1

-1082;0,503;-0,032;1

-1111;0,121;0,064;1

-1128;-0,239;-0,09;1

-1146;-0,369;0,129;1

-1156;-0,674;0,019;1

-1173;-0,53;-0,016;1

-1188;-0,42;0,07;1

-1203;-0,395;-0,052;1

-1213;-0,128;-0,06;1

-1225;-0,18;0,02;1

-1231;-0,113;-0,051;1

-1239;-0,168;0,094;1

-1242;-0,262;-0,064;1

-1249;-0,325;0,039;1

-1250;-0,148;0,036;1

-1254;0,212;-0,02;1

-1256;0,378;0,171;1

-1254;-0,025;0,018;1

-1235;0,046;-0,002;1

-1207;0,504;0,054;1

-1169;0,528;-0,182;1

-1128;0,467;0,061;1

-1084;0,43;-0,028;1

-1031;0,371;-0,018;1

-967;0,074;0,074;1

-893;0,105;-0,094;1

-813;0,293;0,061;1

-729;0,601;0,005;1

-648;0,608;-0,057;1

-568;0,09;0,095;1

-511;-0,086;-0,081;1

-486;-0,02;0,029;1

-501;-0,058;0,005;1

-543;0,119;-0,003;1

-605;0,254;0,085;1

-672;0,068;-0,097;1

-734;0,093;-0,003;1

-796;0,313;0,028;1

-847;0,367;-0,081;1

-893;0,142;0,063;1

-931;0,167;-0,037;1

-965;0,072;0,006;1

-990;-0,063;0,047;1

-1018;0,022;-0,069;1

-1046;0,245;0,052;1

-1073;0,243;-0,005;1

-1093;0,382;-0,01;1

-1116;0,239;0,06;1

-1132;0,282;-0,023;1

-1149;0,1;0,078;1

-1163;-0,159;0,016;1

-1172;0,143;-0,045;1

-1184;0,206;0,094;1

-1195;0,101;-0,065;1

-1203;0,101;0,026;1

-1202;0,069;0,061;1

-1190;0,113;-0,038;1

-1163;-0,2;0,074;1

-1127;-0,323;-0,025;1

-1079;-0,137;-0,014;1

-1024;-0,149;0,055;1

-955;-0,045;-0,096;1

-883;0,09;0,08;1

-805;0,347;-0,004;1

-723;0,505;-0,026;1

-634;0,226;0,066;1

-541;0,212;-0,093;1

-446;0,146;0,089;1

-375;0,131;0,017;1

-346;0,281;0,042;1

-372;0,182;0,238;1

-438;-0,21;0,506;1

-521;-0,161;0,033;1

-603;-0,107;0,473;1

-667;0,457;0,358;1

-723;0,133;0,18;1

-766;-0,264;-0,29;1

-801;-0,312;0,326;1

-833;-0,455;0,204;1

-860;-0,015;0,15;1

-878;-0,103;-0,227;1

-892;-0,153;0,599;1

-900;0,035;0,255;1

-906;-0,323;0,078;1

-910;-0,462;0,772;1

-912;-0,263;-0,041;1

-916;-0,476;0,024;1

-922;-0,402;-0,041;1

-929;-0,271;0,157;1

-918;-0,096;0,018;1

-893;-0,219;0,056;1

-852;-0,301;-0,005;1

-802;-0,104;-0,072;1

-740;-0,477;0,459;1

-672;-0,461;-0,419;1

-588;-0,367;-0,012;1

-499;-0,828;-0,069;1

-401;-0,413;-0,128;1

-302;0,123;0,159;1

-198;0,173;-0,028;1

-119;-0,022;0,02;1

-83;-0,037;0,008;1

-108;0,249;0,067;1

-183;0,142;0,163;1

-281;-0,013;0,12;1

-374;0,155;-0,07;1

-456;-0,071;0,06;1

-513;0,173;-0,262;1

-552;0,505;0,036;1

-578;0,412;-0,098;1

-587;0,628;-0,035;1

-597;0,354;0,069;1

-602;0,17;-0,053;1

-615;0,054;0,063;1

-633;-0,053;-0,013;1

-655;0,24;-0,003;1

-676;-0,112;0,09;1

-698;-0,085;-0,097;1

-724;-0,201;0,041;1

-751;-0,375;0,066;1

-787;-0,176;-0,113;1

-823;-0,325;0,055;1

-864;-0,064;-0,022;1

-897;-0,124;-0,106;1

-929;-0,305;0,088;1

-958;-0,16;-0,081;1

-988;-0,312;0,067;1

-1017;-0,439;-0,043;1

-1046;-0,183;0,04;1

-1072;-0,479;0,042;1

-1101;-0,593;-0,71;1

-1131;-0,454;0,161;1

-1164;-0,506;0,495;1

-1191;-0,589;-0,105;1

-1211;-0,49;0,057;1

-1212;-0,226;-0,164;1

-1194;-0,162;-0,513;1

-1163;-0,414;-0,122;1

-1118;-0,105;-0,013;1

-1061;0,141;0,239;1

-998;0,213;0,103;1

-928;0,324;-0,754;1

-849;0,294;-0,004;1

-765;0,33;-0,052;1

-671;0,496;0,125;1

-572;0,662;0,002;1

-470;0,792;0,031;1

-376;0,744;0,073;1

-311;0,686;-0,054;1

-310;0,64;0,11;1

-378;0,645;0,02;1

-485;0,516;-0,01;1

-591;0,039;0,043;1

-688;0,46;-0,001;1

-766;0,82;-0,008;1

-835;0,423;-0,062;1

-894;0,363;-0,056;1

-936;0,404;0,096;1

-975;0,3;-0,082;1

-1003;0,077;0,002;1

-1030;-0,073;-0,154;1

-1062;0,191;0,003;1

-1090;0,29;0,045;1

-1118;0,192;-0,08;1

-1149;0,213;0,092;1

-1177;-0,154;0,009;1

-1204;-0,314;-0,086;1

-1226;-0,685;0,098;1

-1245;-0,874;0,003;1

-1261;-0,609;-0,082;1

-1276;-0,937;0,09;1

-1295;-0,947;-0,092;1

-1309;-0,438;0,102;1

-1325;-0,508;0,018;1

-1336;-0,125;0,003;1

-1351;0,107;0,094;1

-1365;0,135;0,014;1

-1374;0,183;-0,008;1

-1366;-0,016;-0,069;1

-1348;0,083;-0,07;1

-1314;0,191;0,058;1

-1273;0,127;-0,152;1

-1222;0,166;0,054;1

-1167;-0,066;0,023;1

-1105;0,106;0,009;1

-1049;0,273;0,056;1

-986;-0,176;-0,025;1

-927;-0,007;0,005;1

-858;0,104;0,067;1

-784;0,216;-0,054;1

-697;0,157;0,041;1

-612;0,156;-0,044;1

-543;0,308;-0,032;1

-512;0,389;0,047;1

-533;0,878;-0,037;1

-591;0,893;0,092;1

-672;0,46;0,03;1

-749;0,484;-0,028;1

-819;0,527;-0,039;1

-875;0,387;-0,075;1

-921;0,233;0,031;1

-958;0,334;-0,025;1

-991;0,128;-0,113;1

-1022;-0,18;0,068;1

-1043;-0,083;0,025;1

-1069;0,119;0,022;1

-1096;0,09;0,063;1

-1126;0,343;-0,141;1

-1152;0,121;0,089;1

-1175;0,015;-0,01;1

-1191;0,569;-0,007;1

-1208;0,66;0,096;1

-1221;0,525;-0,092;1

-1237;0,558;0,05;1

-1250;0,412;-0,08;1

-1263;0,477;0,12;1

-1271;0,281;0,085;1

-1284;0,441;-0,114;1

-1295;0,531;0,05;1

-1307;0,164;-0,115;1

-1314;0,132;-0,043;1

-1310;-0,005;0,172;1

-1286;-0,352;-0,038;1

-1256;-0,488;0,059;1

-1214;-0,764;0,043;1

-1175;-0,647;-0,072;1

-1131;-0,529;0,059;1

-1087;-0,458;-0,019;1

-1036;-0,28;-0,007;1

-988;-0,38;0,039;1

-935;-0,291;-0,145;1

-879;-0,363;0,117;1

-819;-0,578;-0,011;1

-753;-0,561;-0,037;1

-678;-0,505;0,104;1

-599;-0,466;-0,081;1

-537;-0,117;0,038;1

-503;-0,009;-0,009;1

-514;0,385;-0,044;1

-558;0,374;0,092;1

-621;0,145;-0,096;1

-698;0,559;0,031;1

-770;0,507;0,012;1

-835;0,517;-0,055;1

-887;0,459;0,094;1

-931;0,501;-0,055;1

-966;0,932;-0,011;1

-1003;0,719;0,024;1

-1036;0,616;-0,082;1

-1075;0,546;0,111;1

-1109;0,48;-0,032;1

-1139;0,422;-0,06;1

-1164;0,347;0,04;1

-1188;0,54;-0,094;1

-1205;0,557;0,059;1

-1226;0,323;-0,008;1

-1245;0,482;-0,01;1

-1265;0,261;0,078;1

-1281;0,054;-0,107;1

-1301;-0,03;-0,025;1

-1314;-0,298;0,014;1

-1328;-0,105;-0,05;1

-1339;0,028;0,084;1

-1346;0,275;-0,066;1

-1350;0,119;0,003;1

-1354;-0,169;0,021;1

-1345;0,345;-0,045;1

-1330;0,667;0,101;1

-1301;0,506;-0,067;1

-1269;0,532;0,024;1

-1228;0,217;0,021;1

-1182;0,282;-0,096;1

-1135;0,361;0,078;1

-1082;0,329;-0,046;1

-1024;0,466;0,022;1

-962;0,233;0,041;1

-894;0,466;-0,089;1

-814;0,513;0,066;1

-732;0,469;0,011;1

-647;0,561;0,07;1

-575;0,542;0,096;1

-529;0,446;-0,065;1

-523;0,508;0,045;1

-563;0,607;-0,025;1

-627;0,763;-0,038;1

-701;0,623;0,067;1

-766;0,68;-0,027;1

-828;0,69;0,017;1

-881;0,606;0,039;1

-933;0,808;-0,054;1

-979;0,277;0,089;1

-1025;0,012;-0,047;1

-1062;0,295;-0,038;1

-1094;0,006;0,043;1

-1118;0,136;-0,079;1

-1142;0,295;0,072;1

-1162;0,133;-0,06;1

-1183;0,184;-0,037;1

-1201;-0,115;0,07;1

-1219;0,012;-0,078;1

-1238;0,303;0,045;1

-1257;0,127;0,004;1

-1272;0,154;-0,056;1

-1288;0,42;0,066;1

-1292;0,227;-0,081;1

-1298;-0,016;0,059;1

-1308;-0,026;0,012;1

-1321;0,301;-0,055;1

-1331;0,297;0,055;1

-1340;0,19;-0,031;1

-1340;0,261;-0,058;1

-1334;0,071;0,02;1

-1314;0,428;-0,186;1

-1280;0,311;0,081;1

-1241;-0,06;0,02;1

-1189;0,169;-0,028;1

-1139;-0,004;0,017;1

-1084;-0,001;-0,099;1

-1030;0,131;0,079;1

-971;0,07;0,024;1

-905;0,05;-0,03;1

-830;-0,024;0,068;1

-747;-0,093;-0,044;1

-666;-0,13;0,038;1

-580;-0,312;0,048;1

-507;-0,135;-0,005;1

-463;-0,256;-0,218;1

-468;-0,234;-0,088;1

-517;-0,304;0,12;1

-598;-0,406;0,139;1

-682;-0,169;0,147;1

-760;-0,385;0,089;1

-825;-0,215;0,132;1

-880;0,353;0,129;1

-927;0,248;0,09;1

-968;0,18;0,027;1

-1000;0,181;0,184;1

-1033;-0,055;0,028;1

-1059;0,368;0,059;1

-1083;0,117;0,123;1

-1104;-0,053;-0,055;1

-1127;-0;0,06;1

-1149;0,06;0,116;1

-1172;0,287;-0,065;1

-1194;0,146;0,118;1

-1215;0,063;-0,049;1

-1234;0,206;0,138;1

-1259;0,142;0,017;1

-1275;-0,061;0,078;1

-1272;-0,034;0,027;1

-1246;0,311;0,244;1

-1192;0,406;-0,244;1

-1125;0,5;-0,646;1

-1044;0,378;-0,056;1

-953;-0,008;-1,521;1

-854;0,109;0,454;1

-756;0,041;-0,86;1

-654;-0,083;-0,442;1

-547;-0,115;-0,099;1

-434;0,02;0,579;1

-314;0,153;-1,067;1

-194;0,166;-0,924;1

-92;-0,086;0,383;1

-51;0,013;-1,139;1

-92;0,492;0,88;1

-198;0,568;0,852;1

-308;0,429;0,581;1

-404;0,291;-1,676;1

-465;0,337;0,483;1

-492;0,546;1,199;1

-494;0,171;0,557;1

-489;-0,139;0,496;1

-483;-0,015;0,036;1

-480;-0,076;0,257;1

-472;-0,071;1,146;1

-459;-0,3;-0,557;1

-443;-0,278;-0,473;1

-433;-0,074;-0,614;1

-428;0,133;0,873;1

-426;-0,215;1,157;1

-421;-0,465;-0,277;1

-412;-0,298;-0,482;1

-407;-0,774;0,45;1

-406;-0,812;-0,083;1

-408;-0,233;-0,162;1

-415;-0,97;-0,09;1

-429;-0,906;-0,605;1

-439;-0,558;0,205;1

-456;-0,973;0,088;1

-471;-0,847;-0,209;1

-486;-1,041;0,097;1

-500;-1,172;-0,837;1

-518;-0,577;-0,125;1

-541;-0,157;-0,005;1

-577;-0,245;-0,073;1

-615;-0,553;-0,273;1

-656;-0,368;0,412;1

-690;0,107;-0,494;1

-723;-0,216;-0,09;1

-753;-0,342;-0,109;1

-779;-0,177;-0,161;1

-813;-0,297;-0,357;1

-844;-0,201;0,159;1

-874;-0,345;1,388;1

-892;-0,436;0,17;1

-897;-0,361;-0,31;1

-892;-0,132;-0,02;1

-878;-0,085;-0,406;1

-854;-0,15;0,59;1

-821;-0,075;0,456;1

-784;-0,225;0,079;1

-739;-0,29;0,002;1

-695;-0,074;-0,007;1

-642;-0,264;-0,317;1

-584;-0,226;-0,099;1

-514;-0,079;-0,087;1

-433;-0,217;-0,522;1

-339;0,214;-0,244;1

-243;0,142;0,008;1

-152;-0,034;-1,384;1

-112;-0,063;0,052;1

-156;-0,151;-0,392;1

-281;0,146;0,595;1

-429;-0,126;0,278;1

-577;-0,235;0,039;1

-694;-0,259;-0,79;1

-791;0,168;0,935;1

-874;0,397;0,026;1

-951;0,204;0,407;1

-1028;0,143;-0,524;1

-1104;0,296;-1,393;1

-1163;0,126;1,006;1

-1208;0,306;0,141;1

-1239;-0,096;-0,521;1

-1269;-0,405;-0,742;1

-1301;-0,417;-0,086;1

-1331;-0,173;0,145;1

-1343;-0,11;-0,172;1

-1346;-0,201;1,785;1

-1351;-0,372;-0,315;1

-1357;-0,231;0,058;1

-1370;-0,336;0,173;1

-1388;-0,143;-0,655;1

-1417;-0,172;1,939;1

-1447;-0,571;-0,652;1

-1473;-0,422;1,745;1

-1499;-0,214;0,768;1

-1520;-0,254;0,597;1

-1531;-0,604;-0,098;1

-1516;-0,342;0,736;1

-1478;-0,416;0,396;1

-1409;-0,365;0,09;1

-1324;-0,525;0,738;1

-1224;-0,439;0,446;1

-1125;-0,461;0,362;1

-1025;-0,181;-1,799;1

-915;-0,208;-0,121;1

-776;-0,179;-0,185;1

-626;-0,056;0,064;1

-477;0,001;0,12;1

-351;-0,061;0,381;1

-298;0,055;-0,216;1

-360;-0,104;-0,362;1

-516;0,218;0,183;1

-686;0,211;-0,013;1

-823;0,016;0,1;1

-920;0,156;-0,172;1

-988;0,094;-0,03;1

-1034;-0,053;1,368;1

-1072;-0,03;-0,039;1

-1103;-0,156;-0,815;1

-1136;0,007;1,55;1

-1168;-0,447;0,686;1

-1210;-0,545;0,417;1

-1248;-0,339;-0,135;1

-1287;-0,477;0,811;1

-1322;-0,013;-0;1

-1356;0,062;-0,184;1

-1383;0,185;-0,133;1

-1412;0,036;0,762;1

-1437;-0,299;1,51;1

-1456;0,164;0,217;1

-1482;0,369;-0,154;1

-1503;0,379;-0,576;1

-1522;0,039;0,624;1

-1531;-0,444;0,028;1

-1519;0,036;-0,327;1

-1482;0,285;-1,17;1

-1421;0,026;0,188;1

-1349;-0,082;-0,238;1

-1266;-0,114;0,151;1

-1181;0,282;-0,189;1

-1088;0,082;-0,393;1

-993;-0,102;-0,052;1

-888;0,054;-0,484;1

-775;0,136;0,155;1

-634;0,083;0,685;1

-488;-0,06;-0,253;1

-358;-0,063;0,196;1

-305;0,179;-0,157;1

-385;0,115;2,161;1

-545;0,177;-0,02;1

-713;0,144;0,049;1

-848;-0,179;2,513;1

-941;0,104;-0,461;1

-1002;0,26;-0,077;1

-1046;-0,094;2,066;1

-1070;0,204;0,07;1

-1093;0,255;-0,098;1

-1115;0,452;-0,578;1

-1136;0,592;-0,19;1

-1160;0,37;0,024;1

-1186;0,446;0,128;1

-1204;0,47;1,816;1

-1228;0,676;0,522;1

-1251;0,674;-0,228;1

-1279;0,482;-0,264;1

-1304;0,529;0,123;1

-1333;0,311;0,097;1

-1360;0,13;0,845;1

-1386;0,216;-0,371;1

-1415;-0,089;0,167;1

-1438;-0,072;-0,496;1

-1456;-0,051;-1,788;1

-1460;-0,09;1,752;1

-1457;0,129;-0,175;1

-1436;-0,134;-0,254;1

-1398;-0,061;0,143;1

-1349;-0,378;0,012;1

-1282;-0,535;0,137;1

-1200;-0,488;0,729;1

-1107;-0,7;-0,081;1

-1009;-0,553;-0,627;1

-898;-0,5;-0,327;1

-781;-0,317;-0,198;1

-644;-0,352;1,125;1

-501;-0,485;0,108;1

-370;-0,446;-0,435;1

-303;-0,609;0,121;1

-324;-0,623;-0,138;1

-424;-0,259;-0,198;1

-550;-0,238;-0,035;1

-667;-0,302;-0,224;1

-756;-0,208;-0,355;1

-828;-0,124;0,047;1

-881;-0,378;0,056;1

-931;-0,288;1,203;1

-971;-0,138;-0,027;1

-1015;-0,228;0,025;1

-1051;-0,026;0,071;1

-1084;0,181;-0,072;1

-1111;0,049;0,117;1

-1136;0,119;-0,107;1

-1159;0,269;-0,038;1

-1191;0,195;0,054;1

-1217;0,275;0,012;1

-1244;0,088;0,047;1

-1267;-0,144;-0,084;1

-1289;0,289;-0,034;1

-1309;0,482;0,016;1

-1330;0,178;-0,014;1

-1344;0,017;0,02;1

-1355;0,141;0,011;1

-1360;0,357;-0,097;1

-1363;0,309;0,067;1

-1372;0,469;0,049;1

-1378;0,408;-0,018;1

-1385;0,274;0,116;1

-1378;0,206;-0,045;1

-1356;-0,002;0,048;1

-1321;-0,018;-0,068;1

-1273;0,397;-0,066;1

-1223;0,315;-0,004;1

-1163;0,332;-0,049;1

-1104;0,481;0,049;1

-1035;0,358;0,011;1

-962;0,062;-0,061;1

-880;0,083;0,05;1

-792;0,333;-0,081;1

-696;0,614;0,018;1

-606;0,544;-0,078;1

-534;0,279;-0,223;1

-504;-0,409;0,101;1

-524;-0,405;-0,049;1

-585;0,192;-0,031;1

-661;0,566;0,044;1

-739;0,587;-0,094;1

-807;0,484;0,026;1

-867;0,731;-0,022;1

-916;0,662;0,009;1

-959;0,527;0,046;1

-996;0,849;-0,144;1

-1030;0,969;0,003;1

-1052;0,741;-0,047;1

-1079;0,618;-0,083;1

-1105;0,641;0,112;1

-1133;0,8;-0,071;1

-1162;0,531;0,034;1

-1194;0,173;0,069;1

-1216;0,276;-0,085;1

-1238;-0,019;0,057;1

-1253;0,197;-0,044;1

-1268;0,356;0,026;1

-1283;0,099;0,048;1

-1298;0,093;-0,104;1

-1314;-0,014;0,108;1

-1327;0,391;-0,053;1

-1339;0,656;-0,001;1

-1348;0,167;-0,058;1

-1348;-0,101;-0,139;1

-1339;-0,251;0,09;1

-1310;-0,56;0,052;1

-1275;-0,334;-0,082;2

-1235;-0,122;0,104;2

-1196;0,187;-0,018;2

-1151;0,097;0,084;2

-1110;-0,245;-0,119;2

-1054;-0,113;-0,022;2

-994;-0,082;0,076;2

-925;0,159;-0,197;2

-845;0,378;0,011;2

-761;0,419;0,013;2

-674;0,393;-0,012;2

-595;0,261;0,076;2

-548;0,26;-0,104;2

-545;0,386;0,02;2

-581;0,314;0,013;2

-640;0,431;-0,004;2

-700;0,33;0,049;2

-766;0,421;-0,072;2

-824;0,722;0,013;2

-880;0,52;0,152;2

-929;0,32;0,004;2

-966;0,332;0,072;2

-998;0,21;0,01;2

-1027;0,26;-0,023;2

-1054;-0,017;0,084;2

-1085;0,02;-0,018;2

-1110;0,264;0,028;2

-1138;0,323;0,005;2

-1162;0,303;-0,132;2

-1190;-0,18;0,097;2

-1221;-0,307;-0,048;2

-1245;-0;-0,006;2

-1269;-0,068;0,033;2

-1286;0,087;-0,051;2

-1298;-0,152;0,119;2

-1309;-0,253;-0,044;2

-1322;0,131;0,018;2

-1335;0,348;0,093;2

-1343;0,352;-0,051;2

-1352;0,291;0,134;2

-1353;0,48;0,015;2

-1360;0,609;-0,047;2

-1363;0,445;0,124;2

-1366;0,718;-0,116;2

-1354;0,589;0,128;2

-1331;0,511;0,054;2

-1291;0,65;0,015;2

-1246;0,483;0,132;2

-1192;0,529;0,012;2

-1139;0,264;0,063;2

-1081;-0,028;0,043;2

-1022;0,343;-0,051;2

-958;0,358;0,072;2

-889;0,385;-0,136;2

-814;0,578;0,048;2

-734;0,151;0,063;2

-656;0,046;-0,069;2

-594;-0,314;0,016;2

-561;-0,758;-0,014;2

-565;-0,466;-0,02;2

-602;-0,254;-0,052;2

-657;-0,062;-0,09;2

-717;-0,071;0,071;2

-776;-0,25;-0,023;2

-828;-0,158;-0,022;2

-872;-0,388;-0,062;2

-917;-0,217;-0,074;2

-954;0,318;0,117;2

-995;0,051;0,026;2

-1028;-0,112;-0,01;2

-1059;0,167;0,071;2

-1084;0,461;-0,008;2

-1110;0,587;0,088;2

-1137;0,177;0,031;2

-1164;0,527;-0,035;2

-1189;0,36;0,152;2

-1208;0,094;-0,077;2

-1227;0,269;-0,064;2

-1242;0,179;-0,007;2

-1254;0,265;-0,135;2

-1269;0,11;0,071;2

-1282;-0,185;-0,03;2

-1297;0,099;0,014;2

-1310;0,178;0,063;2

-1324;-0,118;-0,039;2

-1334;-0,473;-0,021;2

-1339;-0,405;-0,035;2

-1336;-0,207;-0,076;2

-1336;-0,21;0,157;2

-1332;0,264;-0,103;2

-1328;0,196;0,028;2

-1311;-0,087;-0,004;2

-1283;0,269;0,043;2

-1240;-0,074;0,05;2

-1191;-0,043;-0,034;2

-1139;0,422;0,063;2

-1084;0,177;0,018;2

-1020;0,587;-0,065;2

-951;0,554;0,158;2

-875;0,343;-0,123;2

-797;0,576;-0,029;2

-721;0,577;0,049;2

-649;0,651;-0,013;2

-583;0,799;0,127;2

-534;0,272;-0,006;2

-515;0,633;-0,007;2

-532;0,587;0,03;2

-582;0,355;-0,045;2

-643;0,125;0,067;2

-705;0,182;0,025;2

-759;0,399;-0,028;2

-808;-0,006;0,075;2

-847;0,214;-0,067;2

-885;0,687;-0,095;2

-920;0,652;0,011;2

-953;0,579;-0,033;2

-985;0,386;0,103;2

-1013;0,436;-0,076;2

-1046;0,419;0,028;2

-1072;0,105;0,051;2

-1100;0,323;-0,126;2

-1126;0,22;0,067;2

-1150;0,21;-0,018;2

-1170;0,082;0,027;2

-1191;-0,151;0,062;2

-1208;0,007;-0,126;2

-1230;-0,109;-0,006;2

-1249;-0,247;-0,049;2

-1269;0,081;-0,065;2

-1283;0,028;0,086;2

-1294;0,102;-0,038;2

-1306;0,194;0,084;2

-1312;0,068;-0,044;2

-1318;0,294;0,003;2

-1320;0,307;0,066;2

-1323;0,276;-0,134;2

-1324;0,201;0,024;2

-1327;0,013;-0,043;2

-1325;0,267;-0,023;2

-1319;0,139;0,091;2

-1299;-0,08;0;2

-1268;-0,138;-0,005;2

-1228;-0,223;0,043;2

-1186;-0,284;-0,094;2

-1137;-0,511;0,097;2

-1089;-0,795;-0,036;2

-1029;-0,646;0,001;2

-969;-0,468;0,015;2

-901;-0,403;-0,042;2

-836;-0,663;0,043;2

-765;-0,842;-0,04;2

-695;-0,261;-0,036;2

-624;-0,183;0,092;2

-566;-0,009;-0,122;2

-544;0,404;0,093;2

-560;0,534;-0,001;2

-608;0,809;0,02;2

-664;0,939;0,037;2

-718;0,872;-0,056;2

-768;0,976;0,023;2

-816;0,734;0,054;2

-860;0,624;-0,137;2

-901;0,523;0,038;2

-938;0,636;-0,14;2

-964;0,778;0,031;2

-988;0,795;-0,1;2

-1010;0,862;-0,134;2

-1035;0,552;0,03;2

-1058;0,544;-0,008;2

-1078;0,892;-0,04;2

-1098;0,865;0,109;2

-1123;0,804;-0,048;2

-1147;0,691;-0,007;2

-1173;0,44;-0,045;2

-1192;0,204;0,051;2

-1208;0,128;0,101;2

-1222;0,428;-0,071;2

-1237;0,338;0,098;2

-1251;0,206;0,001;2

-1262;0,383;0,018;2

-1273;0,154;0,092;2

-1276;-0,432;-0,044;2

-1283;-0,476;-0,003;2

-1286;-0,454;0,02;2

-1290;-0,313;-0,061;2

-1297;-0,524;0,161;2

-1302;-0,775;-0,001;2

-1302;-0,438;0,007;2

-1296;-0,704;0,019;2

-1277;-0,562;-0,027;2

-1253;-0,304;0,056;2

-1212;-0,527;-0,014;2

-1167;-0,391;-0,036;2

-1114;-0,698;0,133;2

-1059;-0,59;-0,055;2

-1005;-0,348;-0,004;2

-949;-0,292;0,062;2

-896;-0,051;0,015;2

-840;-0,293;0,061;2

-781;-0,467;-0,051;2

-711;-0,269;0,077;2

-638;-0,336;-0,035;2

-568;0,124;-0,056;2

-517;0,056;-0,059;2

-499;-0,021;-0,141;2

-520;0,088;-0,037;2

-571;0,063;0,061;2

-629;0,415;-0,173;2

-691;0,313;0,081;2

-746;0,12;-0,001;2

-805;0,084;-0,084;2

-858;-0,008;0,061;2

-903;0,117;-0,052;2

-937;0,184;0,023;2

-967;0,462;-0,026;2

-987;0,69;-0,006;2

-1013;0,8;-0,042;2

-1035;0,999;-0,074;2

-1059;0,956;-0,105;2

-1080;0,741;0,065;2

-1103;0,798;-0,14;2

-1125;0,652;0,053;2

-1150;0,087;-0,044;2

-1166;0,272;0,03;2

-1185;0,212;-0,069;2

-1200;-0,023;-0,083;2

-1220;-0,174;0,103;2

-1238;0,091;-0,053;2

-1253;0,154;0,016;2

-1261;0,158;0,11;2

-1267;0,281;0,012;2

-1270;0,108;0,097;2

-1278;-0,078;-0,135;2

-1286;-0,266;-0,062;2

-1299;-1,007;0,04;2

-1307;-0,761;0,041;2

-1309;-0,621;-0,026;2

-1313;-0,68;-0,003;2

-1305;-0,593;0,011;2

-1292;-0,789;0,101;2

-1267;-0,573;-0,077;2

-1232;-0,48;-0,073;2

-1197;-0,338;-0,068;2

-1157;-0,115;0,018;2

-1118;-0,212;-0,03;2

-1077;-0,293;-0,02;2

-1031;-0,35;0,061;2

-973;-0,266;0,096;2

-915;0,064;-0,22;2

-843;-0,033;0,063;2

-774;-0,195;-0,007;2

-695;-0,272;-0,065;2

-623;-0,321;0,077;2

-559;-0,269;-0,016;2

-522;-0,421;0,076;2

-520;-0,359;0,032;2

-550;-0,346;-0,046;2

-600;-0,711;0,109;2

-658;-0,843;-0,1;2

-716;-0,982;0,057;2

-779;-0,785;0,027;2

-834;-0,052;-0,058;2

-876;0,119;0,097;2

-914;0,132;-0,086;2

-939;0,259;0,069;2

-964;0,396;0,025;2

-986;0,591;-0,046;2

-1009;0,038;0,058;2

-1030;-0,043;-0,069;2

-1056;0,256;0,021;2

-1079;0,133;0,181;2

-1104;-0,004;-0,058;2

-1123;0,267;0,069;2

-1144;0,312;-0,037;2

-1157;0,001;-0,105;2

-1174;-0,256;0,075;2

-1191;-0,05;-0,066;2

-1208;-0,286;0,05;2

-1221;-0,432;-0,024;2

-1230;-0,098;-0,056;2

-1243;-0,207;0,083;2

-1250;-0,279;-0,1;2

-1261;-0,549;0,032;2

-1271;-0,835;0,072;2

-1282;-0,983;-0,15;2

-1295;-0,942;0,101;2

-1304;-1,02;-0,005;2

-1317;-1,107;0,037;2

-1320;-1,426;-0,027;2

-1316;-0,957;0,006;2

-1298;-0,936;-0,008;2

-1272;-0,662;-0,037;2

-1239;-0,327;-0,007;2

-1202;-0,223;0,033;2

-1159;0,036;-0,068;2

-1115;-0,045;0,125;2

-1064;-0,203;-0,076;2

-1006;0,025;-0,12;2

-944;-0,036;0,063;2

-874;-0,142;-0,206;2

-791;-0,059;0,13;2

-706;0,009;0,004;2

-622;0,104;-0,031;2

-540;-0,011;0,164;2

-475;0,055;-0,082;2

-435;-0,177;0,069;2

-444;-0,433;0,02;2

-500;-0,253;-0,072;2

-585;-0,574;0,16;2

-676;-0,503;-0,061;2

-761;0,15;-0,046;2

-829;0,283;0,023;2

-890;0,256;-0,03;2

-936;0,331;0,122;2

-979;0,414;-0,059;2

-1010;0,53;0,026;2

-1041;0,236;0,089;2

-1070;0,047;-0,082;2

-1094;0,164;0,051;2

-1122;-0,096;0,032;2

-1148;-0,421;-0,02;2

-1173;-0,561;0,055;2

-1196;-0,375;-0,057;2

-1212;-0,485;0,055;2

-1221;-0,519;-0,03;2

-1228;-0,629;-0,112;2

-1238;-0,843;0,105;2

-1250;-1,133;-0,042;2

-1263;-1,326;0,061;2

-1264;-1,428;-0,007;2

-1257;-1,13;-0,063;2

-1231;-0,91;0,109;2

-1198;-0,983;-0,025;2

-1153;-0,705;-0,036;2

-1102;-1,113;0,041;2

-1037;-0,755;-0,066;2

-967;-0,529;0,085;2

-897;-0,31;0,056;2

-821;-0,399;-0,042;2

-736;-0,431;0,13;2

-645;0,008;-0,069;2

-549;-0,043;0,044;2

-459;0,085;-0,034;2

-382;-0,046;-0,019;2

-336;-0,374;0,042;2

-349;-0,339;-0,109;2

-421;-0,216;-0,024;2

-527;-0,445;0,019;2

-633;-0,308;-0,489;2

-730;-0,426;0,215;2

-805;-0,299;-0,333;2

-867;0,022;0,148;2

-915;-0,223;0,014;2

-960;-0,248;0,075;2

-995;-0,058;0,263;2

-1030;0,071;-0,026;2

-1061;0,033;-0,313;2

-1094;-0,05;0,253;2

-1127;-0,14;0,021;2

-1154;-0,166;0,137;2

-1176;-0,354;-0,425;2

-1191;-0,499;-0,061;2

-1210;-0,437;0,141;2

-1226;-0,215;0,089;2

-1244;-0,198;-0,33;2

-1258;-0,481;0,019;2

-1258;-0,166;-0,103;2

-1236;-0,523;0,19;2

-1192;-0,368;0,019;2

-1140;-0,439;0,174;2

-1077;-0,571;0,016;2

-1007;-0,177;0,144;2

-920;-0,36;0,115;2

-830;-0,552;-0,061;2

-733;-0,52;0,08;2

-636;-0,764;0,074;2

-528;-0,535;-0,069;2

-424;-0,581;0,117;2

-326;-0,284;-0,015;2

-249;0,102;0,018;2

-219;-0,284;-0,029;2

-274;-0,102;-0,142;2

-391;-0,16;0,156;2

-523;-0,274;0,024;2

-636;-0,204;0,141;2

-726;-0,622;0,187;2

-795;-0,496;-0,079;2

-851;-0,274;0,047;2

-896;-0,421;-0,205;2

-934;-0,301;-0,003;2

-974;-0,383;-0,593;2

-1010;-0,396;-0,018;2

-1050;-0,31;0,053;2

-1085;-0,297;0,065;2

-1116;-0,459;-0,087;2

-1143;-0,314;0,127;2

-1169;-0,458;-0,012;2

-1192;-0,21;0,074;2

-1217;-0,273;0,07;2

-1235;-0,303;-0,032;2

-1252;-0,152;0,043;2

-1267;-0,215;-0,002;2

-1283;-0,263;0,008;2

-1302;-0,585;0,09;2

-1308;-0,586;-0,066;2

-1300;-0,651;0,016;2

-1268;-0,77;0,037;2

-1224;-0,524;-0,093;2

-1170;-0,425;0,078;2

-1103;-0,4;-0,1;2

-1032;-0,345;0,071;2

-952;-0,612;0,046;2

-863;-0,848;-0,055;2

-762;-0,99;-0,002;2

-657;-1,145;-0,006;2

-542;-1,058;-0,068;2

-437;-0,935;0,055;2

-349;-0,087;-0,098;2

-308;-0,421;0,051;2

-334;-1,169;-0,047;2

-420;-1,332;-0,006;2

-532;-0,868;0,066;2

-646;-0,366;-0,051;2

-742;-0,287;0,081;2

-825;-0,068;-0,027;2

-883;0,6;-0,055;2

-934;0,965;0,103;2

-976;0,941;-0,048;2

-1018;1,06;-0,022;2

-1054;1,367;-0,038;2

-1088;1,538;-0,053;2

-1118;1,063;0,131;2

-1147;0,571;-0,088;2

-1172;0,46;0,042;2

-1199;0,149;0,031;2

-1223;0,244;-0,07;2

-1247;0,029;0,05;2

-1267;-0,092;-0,057;2

-1290;0,069;0,005;2

-1311;-0,036;0,084;2

-1325;0,024;-0,078;2

-1341;-0,073;0,039;2

-1348;-0,288;-0,1;2

-1358;-0,393;-0,015;2

-1363;-0,576;0,141;2

-1364;-0,577;-0,103;2

-1354;-0,493;0,034;2

-1324;-0,4;0,061;2

-1284;-0,202;-0,115;2

-1236;-0,454;0,078;2

-1188;-0,537;-0,044;2

-1137;-0,401;0,04;2

-1086;-0,395;-0,026;2

-1025;-0,226;-0,061;2

-956;-0,285;0,133;2

-875;-0,321;-0,139;2

-793;-0,149;0,006;2

-704;-0,214;0,037;2

-615;-0,217;-0,124;2

-531;-0,023;0,081;2

-477;0,161;0,01;2

-469;0,479;-0,085;2

-514;0,166;0,072;2

-590;0,086;-0,094;2

-682;0,051;0,074;2

-771;-0,155;-0,047;2

-851;0,104;-0,08;2

-917;0,168;0,063;2

-970;0,246;-0,083;2

-1011;0,24;-0,037;2

-1046;0,383;-0,027;2

-1074;0,556;0,014;2

-1101;0,206;0,072;2

-1127;0,166;-0,05;2

-1150;0,262;-0,003;2

-1178;0,132;0,018;2

-1205;-0,509;0,024;2

-1231;-0,56;0,105;2

-1262;-0,319;-0,026;2

-1284;-0,379;-0,007;2

-1299;-0,658;0,047;2

-1312;-0,84;-0,135;2

-1321;-0,826;0,089;2

-1332;-0,622;-0,016;2

-1341;-0,324;-0,088;2

-1354;-0,267;0,069;2

-1363;-0,058;-0,07;2

-1368;0,052;0,025;2

-1369;0,166;0,031;2

-1351;0,182;0,007;2

-1326;-0,394;0,105;2

-1292;-0,211;-0,051;2

-1249;0,019;0,011;2

-1197;0,131;0,005;2

-1138;0,044;-0,071;2

-1075;-0,014;0,079;2

-1010;0,342;-0,059;2

-936;0,167;0,011;2

-859;-0,338;-0,041;2

-774;0,011;-0,046;2

-688;-0,549;0,085;2

-612;-0,847;-0,038;2

-550;-0,949;0,029;2

-517;-1,246;0,071;2

-521;-1,052;-0,068;2

-563;-0,846;0,042;2

-631;-0,867;-0,03;2

-713;-0,608;-0,032;2

-794;-0,628;0,076;2

-870;-0,757;-0,025;2

-935;-0,524;0,009;2

-992;-0,217;0,034;2

-1033;0,133;-0,03;2

-1066;0,183;0,079;2

-1087;0,467;-0,007;2

-1110;0,616;0,041;2

-1128;0,275;0,041;2

-1153;0,503;0,003;2

-1175;0,396;0,086;2

-1203;0,416;-0,065;2

-1226;0,194;0,013;2

-1251;-0,253;0,029;2

-1271;-0,099;-0,069;2

-1283;-0,149;0,061;2

-1296;0,199;-0,034;2

-1307;0,08;-0,042;2

-1321;-0,516;0,03;2

-1333;-0,475;-0,078;2

-1339;-0,355;0,04;2

-1327;-0,566;-0,006;2

-1294;-0,461;0,01;2

-1250;-0,628;0,041;2

-1202;-0,426;-0,118;2

-1154;-0,567;0,108;2

-1102;-0,704;0,054;2

-1051;-0,362;-0,057;2

-986;-0,493;0,086;2

-918;-0,431;-0,094;2

-838;-0,457;0,035;2

-755;-0,66;0,047;2

-664;-0,17;-0,096;2

-579;-0,146;0,085;2

-505;0,049;-0,052;2

-465;0,07;-0,026;2

-480;-0,162;0,062;2

-541;0,144;-0,12;2

-627;-0,21;0,11;2

-712;-0,287;-0,011;2

-785;0,041;-0,006;2

-846;-0,027;0,042;2

-906;0,105;-0,046;2

-958;0,374;0,032;2

-1008;0,34;-0,047;2

-1045;0,777;0,019;2

-1074;0,764;0,07;2

-1095;0,567;-0,002;2

-1116;0,358;0,043;2

-1132;-0,034;-0,022;2

-1155;0,189;-0,088;2

-1175;-0,098;0,111;2

-1200;-0,203;-0,077;2

-1226;0,289;-0,021;2

-1252;-0,174;0,033;2

-1272;0,063;-0,081;2

-1287;0,077;0,087;2

-1295;-0,112;-0,06;2

-1302;0,159;0,052;2

-1310;0,357;0,051;2

-1308;0,454;-0,076;2

-1295;0,393;0,041;2

-1268;0,209;0,027;2

-1228;0,559;-0,047;2

-1183;0,443;0,044;2

-1127;0,548;-0,129;2

-1071;0,331;0,033;2

-1002;0,018;-0,013;2

-939;0,324;0,047;2

-867;0,337;0,034;2

-798;0,352;-0,061;2

-722;0,029;0,056;2

-643;-0,137;-0,005;2

-561;0,145;-0,07;2

-492;-0,06;0,071;2

-451;-0,274;-0,072;2

-466;-0,222;-0,039;2

-526;-0,631;0,027;2

-612;-1,226;-0,041;2

-707;-1,403;0,102;2

-789;-1,18;-0,067;2

-861;-0,967;-0,043;2

-915;-0,915;0,113;2

-962;-0,684;-0,064;2

-999;-0,558;0,046;2

-1031;-0,175;-0,03;2

-1060;0,154;-0,029;2

-1090;0,038;0,127;2

-1115;0,467;0,004;2

-1141;0,559;0,06;2

-1155;0,367;0,051;2

-1173;0,206;-0,082;2

-1186;-0,322;0,085;2

-1201;-0,097;-0,067;2

-1218;0,128;0,024;2

-1237;-0,126;0,012;2

-1254;0,209;-0,083;2

-1266;-0,004;0,126;2

-1276;-0,122;-0,036;2

-1280;-0,037;-0,027;2

-1285;-0,018;-0,047;2

-1288;-0,121;-0,073;2

-1295;0,199;0,031;2

-1292;0,121;0,043;2

-1274;-0,095;-0,013;2

-1240;-0,145;0,113;2

-1191;-0,083;-0,072;2

-1139;-0,301;0,087;2

-1078;-0,354;-0,038;2

-1018;-0,176;0,024;2

-951;-0,199;0,056;2

-882;0,231;-0,114;2

-814;0,137;0,036;2

-746;-0,248;0,005;2

-674;-0,146;-0,134;2

-604;-0,347;0,075;2

-533;-0,391;-0,108;2

-475;-0,555;0,004;2

-440;-0,569;0,004;2

-447;-0,296;-0,041;2

-502;-0,485;0,076;2

-579;-0,436;0,003;2

-665;-0,312;-0,009;2

-742;-0,286;0,016;2

-807;-0,079;-0,066;2

-867;-0,038;0,064;2

-918;0,141;-0,063;2

-966;0,008;0,018;2

-1004;-0,152;0,097;2

-1034;-0,149;-0,007;2

-1064;0,233;0,081;2

-1088;0,26;0,022;2

-1112;0,603;-0,017;2

-1129;0,679;0,082;2

-1145;0,708;-0,017;2

-1160;0,787;0,005;2

-1183;0,396;0,048;2

-1203;0,633;0,009;2

-1226;0,632;0,182;2

-1242;0,201;-0,089;2

-1254;0,407;-0,017;2

-1270;0,219;0,034;2

-1279;0,334;-0,09;2

-1287;0,219;0,133;2

-1284;-0,079;-0,003;2

-1270;-0,189;0,002;2

-1245;-0,701;0,096;2

-1211;-0,466;-0,106;2

-1174;-0,204;0,113;2

-1127;-0,159;-0,014;2

-1074;-0,5;0,04;2

-1014;-0,953;0,089;2

-949;-0,464;-0,118;2

-880;-0,026;0,079;2

-812;-0,092;0,018;2

-733;-0,242;-0,025;2

-657;0,095;0,074;2

-577;0,266;-0,063;2

-510;0,037;0,004;2

-463;0,069;-0,019;2

-455;-0,022;-0,056;2

-491;-0,023;0,035;2

-565;0,236;0,073;2

-651;0,122;0,004;2

-734;-0,162;0,006;2

-805;0,213;-0,077;2

-865;0,46;0,063;2

-913;0,275;0,02;2

-956;0,209;0;2

-994;0,161;0,057;2

-1027;0,182;0,042;2

-1056;0,216;0,068;2

-1080;0,222;-0,003;2

-1102;0,195;-0,032;2

-1126;-0,075;0,206;2

-1149;-0,129;-0,07;2

-1169;-0,201;0,068;2

-1186;-0,246;0,025;2

-1201;0,049;-0,065;2

-1220;0,163;0,06;2

-1235;0,073;-0,033;2

-1245;0,151;-0,009;2

-1258;0,22;0,029;2

-1265;0,029;-0,06;2

-1279;-0,382;0,082;2

-1291;0,016;-0,034;2

-1300;0,381;0,112;2

-1302;0,287;0,027;2

-1288;0,217;-0,09;2

-1258;0,136;0,115;2

-1217;0,153;-0,13;2

-1172;0,044;-0,021;2

-1121;-0,186;0,087;2

-1067;-0,101;-0,095;2

-1006;-0,019;0,068;2

-946;-0,256;0,039;2

-874;-0,212;0,014;2

-803;-0,17;0,085;2

-722;-0,257;0,041;2

-636;-0,2;0,035;2

-559;-0,007;0,029;2

-498;0,372;-0,02;2

-464;0,777;0,095;2

-473;0,635;0,038;2

-519;0,89;0,09;2

-590;1,149;0,047;2

-666;0,773;-0,05;2

-739;-0,213;0,065;2

-808;-1,178;-0,021;2

-862;-1,137;0,041;2

-907;-0,696;0,127;2

-945;-0,266;-0,045;2

-982;-0,155;0,085;2

-1013;-0,023;-0,047;2

-1042;-0,107;-0,001;2

-1066;-0,149;0,096;2

-1091;-0,139;-0,112;2

-1113;0,032;0,045;2

-1139;0,311;-0,033;2

-1164;0,386;-0,078;2

-1187;-0,08;0,061;2

-1201;0,107;-0,041;2

-1209;0,517;0,094;2

-1221;0,185;0,032;2

-1231;0,259;-0,107;2

-1245;0,125;0,119;2

-1258;-0,175;-0,088;2

-1267;-0,545;-0,014;2

-1273;-1,167;0,055;2

-1271;-1,023;-0,147;2

-1262;-0,609;0,056;2

-1235;-0,402;0,016;2

-1197;0,121;0,032;2

-1151;0,047;0,078;2

-1104;0,088;-0,038;2

-1054;0,243;0,029;2

-1003;0,255;0,028;2

-939;0,236;-0,053;2

-868;-0,068;-0,008;2

-784;-0,555;-0,092;2

-700;-0,699;0,008;2

-612;-1,036;-0,038;2

-534;-1,086;-0,015;2

-472;-0,921;0,044;2

-453;-0,541;-0,055;2

-486;-0,003;0,022;2

-562;0,188;0,052;2

-652;0,303;-0,054;2

-740;0,215;-0,03;2

-813;0,242;-0,091;2

-880;0,435;0,019;2

-935;0,54;0,103;2

-982;0,759;-0,041;2

-1016;0,453;0,033;2

-1042;0,03;0,003;2

-1065;-0,074;-0,014;2

-1088;-0,73;0,117;2

-1111;-0,986;-0,052;2

-1135;-0,936;0,057;2

-1160;-0,649;0,074;2

-1182;-0,224;-0,049;2

-1206;-0,698;0,094;2

-1228;-0,601;-0,04;2

-1246;-0,398;0,041;2

-1261;-0,829;0,006;2

-1272;-0,522;-0,068;2

-1282;-0,442;0,062;2

-1292;-0,102;-0,037;2

-1301;-0,138;0,011;2

-1307;-0,513;0,063;2

-1313;0,23;-0,081;2

-1311;-0,069;0,04;2

-1302;-0,42;-0,032;2

-1276;-0,121;-0,02;2

-1239;0,159;0,096;2

-1190;0,383;-0,086;2

-1134;0,201;0,052;2

-1076;0,449;0,002;2

-1017;0,53;0,048;2

-954;0,491;0,078;2

-883;0,805;-0,104;2

-804;0,63;0,053;2

-715;-0,006;0,034;2

-634;-0,346;-0,118;2

-555;-0,084;0,05;2

-495;-0,095;-0,073;2

-461;-0,45;-0,002;2

-476;-0,443;-0,2;2

-533;0,004;-0,079;2

-621;-0,31;0,064;2

-711;-0,495;-0,074;2

-797;-0,138;0,05;2

-861;0,134;0,157;2

-914;0,356;-0,047;2

-958;0,517;0,08;2

-999;0,512;-0,015;2

-1030;0,527;-0,005;2

-1056;0,155;0,037;2

-1076;-0,011;-0,103;2

-1097;0,133;-0,001;2

-1118;-0,021;-0,003;2

-1142;-0,034;0,026;2

-1163;0,018;0,09;2

-1180;0,085;-0,103;2

-1199;0,375;0,061;2

-1212;0,398;0,014;2

-1231;0,767;-0,069;2

-1251;0,712;0,084;2

-1264;0,125;-0,071;2

-1279;-0,247;-0,027;2

-1285;-0,141;0,006;2

-1293;0,268;-0,084;2

-1292;0,201;0,056;2

-1280;-0,108;-0,055;2

-1252;-0,071;-0,052;2

-1216;-0,193;0,043;2

-1171;0,044;-0,025;2

-1125;-0,33;-0,01;2

-1074;-0,791;0,022;2

-1021;-0,566;-0,011;2

-957;-0,235;0,051;2

-891;0,289;-0,105;2

-812;0,327;0,066;2

-730;-0,018;-0,009;2

-644;0,289;-0,098;2

-565;0,214;0,056;2

-506;0,177;-0,075;2

-480;0,089;-0,03;2

-496;0,187;0,033;2

-551;0,674;-0,054;2

-627;0,364;0,07;2

-712;-0,211;-0,035;2

-791;-0,124;-0,019;2

-861;-0,271;0,01;2

-916;-0,645;-0,054;2

-961;-0,659;0,035;2

-997;-0,211;-0,034;2

-1030;0,357;-0,005;2

-1057;0,249;0,059;2

-1081;0,259;-0,074;2

-1103;0,34;-0,011;2

-1121;0,482;-0,041;2

-1145;0,773;-0,074;2

-1166;0,65;0,058;2

-1192;0,285;-0,048;2

-1212;0,451;0,028;2

-1229;-0,191;-0,008;2

-1243;-0,624;-0,009;2

-1254;-0,316;0,076;2

-1269;0,082;-0,082;2

-1278;-0,045;0,036;2

-1289;-0,224;0,031;2

-1292;0,009;-0,1;2

-1300;-0,082;0,117;2

-1304;0,018;-0,142;2

-1301;0,055;-0,03;2

-1285;-0,073;0,091;2

-1259;0,132;-0,022;2

-1218;0,205;0,205;2

-1176;0,358;-0,2;2

-1129;0,383;-0,052;2

-1077;-0,142;0,24;2

-1013;-0,652;0,516;2

-938;-0,753;0,238;2

-853;-0,375;-0,081;2

-764;-0,192;-0,108;2

-676;-0,615;0,091;2

-591;-0,78;-0,069;2

-516;-1,186;-0,084;2

-454;-1,28;-0,034;2

-433;-0,712;-0,216;2

-463;-0,281;0,087;2

-534;-0,124;-0,073;2

-618;0,164;0,016;2

-704;-0,264;-0,18;2

-771;-0,144;-0,088;2

-834;0,086;0,069;2

-890;-0,05;-0,042;2

-941;-0,057;-0,07;2

-983;-0,338;0,024;2

-1022;-0,544;-0,047;2

-1050;-0,183;0,081;2

-1080;-0,083;0,014;2

-1109;0,477;0,016;2

-1138;0,062;0,06;2

-1162;-0,186;-0,063;2

-1179;-0,085;0,028;2

-1195;-0,266;0,029;2

-1207;-0,494;-0,071;2

-1226;-0,752;0,038;2

-1241;-0,69;-0,087;2

-1260;-0,707;-0,035;2

-1274;-0,851;0,029;2

-1283;-0,531;-0,063;2

-1285;-0,389;0,092;2

-1263;-0,716;-0,036;2

-1227;-0,309;0,038;2

-1182;-0,467;0,067;2

-1132;-0,321;-0,07;2

-1080;-0,478;0,072;2

-1026;-0,352;0,024;2

-967;0,028;-0,013;2

-906;-0,235;0,084;2

-840;-0,347;-0,137;2

-771;-0,194;0,062;2

-691;-0,049;-0,001;2

-609;-0,106;-0,06;2

-528;-0,242;0,098;2

-469;-0,216;-0,142;2

-444;-0,267;0,026;2

-475;-0,208;0,02;2

-535;-0,047;-0,059;2

-617;0,067;0,078;2

-698;0,307;-0,08;2

-776;0,26;0,011;2

-841;0,092;-0,011;2

-898;0,213;-0,06;2

-942;0,413;0,063;2

-981;0,364;-0,054;2

-1014;0,571;-0,014;2

-1048;0,715;0,068;2

-1077;0,54;-0,103;2

-1107;0,212;0,077;2

-1132;0,348;-0,047;2

-1155;0,465;0,033;2

-1179;0,143;0,028;2

-1198;0,331;-0,098;2

-1220;0,573;-0,015;2

-1238;0,426;-0,025;2

-1256;-0,087;-0,047;2

-1275;-0,546;0,08;2

-1286;-0,562;-0,066;2

-1297;-0,06;0,003;2

-1307;0,1;0,047;2

-1319;0,359;-0,04;2

-1327;0,121;0,061;2

-1335;0,241;-0,049;2

-1336;0,559;0,012;2

-1343;0,202;0,019;2

-1334;0,468;-0,073;2

-1313;0,215;0,075;2

-1275;-0,253;-0,001;2

-1232;-0,031;-0,007;2

-1187;-0,193;0,11;2

-1142;-0,29;-0,059;2

-1094;-0,591;0,034;2

-1038;-0,356;-0,015;2

-968;0,174;0,001;2

-892;0,324;0,047;2

-812;0,483;-0,074;2

-726;0,278;0,105;2

-641;-0,153;0,05;2

-566;-0,422;-0,055;2

-517;-0,568;0,102;2

-506;-0,195;-0,054;2

-538;0,187;0,028;2

-606;0,089;0,021;2

-688;0,384;-0,056;2

-769;0,285;0,07;2

-842;-0,07;-0,039;2

-899;0,01;-0,115;2

-947;0,033;0,042;2

-983;0,306;-0,078;2

-1015;0,458;0,205;2

-1042;0,11;-0,054;2

-1070;0,251;0,035;2

-1096;0,36;0,138;2

-1123;0,553;-0,063;2

-1152;0,733;0,119;2

-1181;0,791;-0,002;2

-1206;0,583;-0,036;2

-1225;0,328;0,071;2

-1242;0,461;-0,042;2

-1253;0,269;0,055;2

-1268;0,302;0,091;2

-1284;0,536;-0,074;2

-1297;0,11;0,09;2

-1312;0,196;-0,074;2

-1320;0,246;-0,008;2

-1326;-0,042;0,053;2

-1324;0,337;-0,1;2

-1309;0,217;0,089;2

-1284;0,085;0,017;2

-1248;0,284;-0,047;2

-1212;0,083;0,092;2

-1173;-0,132;-0,108;2

-1125;-0,173;0,085;2

-1073;-0,145;-0,022;2

-1010;0,074;-0,023;2

-943;-0,146;0,098;2

-870;0,002;-0,081;2

-792;0,198;0,005;2

-714;0,656;-0,019;2

-642;1,041;-0,042;2

-572;0,777;0,081;2

-526;0,853;-0,067;2

-513;0,85;0,014;2

-548;0,811;0,004;2

-611;1,16;-0,086;2

-694;0,692;0,019;2

-772;0,319;-0,094;2

-846;-0,054;0,022;2

-907;-0,457;0,039;2

-959;-0,507;-0,05;2

-998;-0,732;0,09;2

-1030;-0,818;-0,008;2

-1056;-0,494;0,005;2

-1079;-0,346;0,1;2

-1104;-0,583;-0,072;2

-1125;-1,102;0,11;2

-1148;-1,267;-0,101;2

-1165;-0,997;-0,075;2

-1186;-0,45;0,249;2

-1211;-0,248;-0,058;2

-1232;-0,333;0,087;2

-1258;-0,317;-0,026;2

-1281;0,067;-0,069;2

-1300;-0,016;0,071;2

-1314;0,05;-0,096;2

-1322;0,329;0,053;2

-1319;0,233;0,003;2

-1317;0,641;-0,108;2

-1312;0,267;0,054;2

-1309;-0,253;-0,036;2

-1302;-0,237;-0,034;2

-1291;-0,453;0,048;2

-1270;-0,659;-0,093;2

-1241;-0,75;0,026;2

-1204;-0,561;-0,016;2

-1159;-0,239;-0,013;2

-1110;-0,186;0,103;2

-1056;0,03;-0,076;2

-992;0,299;0,086;2

-923;0,059;-0,002;2

-844;0,208;-0,02;2

-757;0,335;0,157;2

-672;0,547;-0,074;2

-594;0,957;0,086;2

-532;0,674;0,133;2

-501;0,443;-0,085;2

-512;0,131;0,097;2

-567;-0,138;-0,067;2

-648;-0,352;-0,089;2

-733;-0,345;0,028;2

-813;-0,114;-0,09;2

-878;-0,101;0,089;2

-933;-0,184;0,016;2

-974;-0,101;0,001;2

-1012;-0,145;0,107;2

-1043;0,474;-0,109;2

-1074;0,394;0,062;2

-1106;0,459;0,044;2

-1128;0,849;0,003;2

-1149;0,456;0,087;2

-1168;0,068;-0,073;2

-1187;0,053;0,015;2

-1205;-0,022;0,025;2

-1228;-0,131;-0,044;2

-1248;-0,322;0,106;2

-1269;-0,278;-0,04;2

-1285;-0,041;-0,08;2

-1297;-0,181;0,071;2

-1305;-0,493;-0,071;2

-1315;-0,624;0,077;2

-1315;-0,177;-0,034;2

-1308;0,399;0,019;2

-1287;0,726;0,038;2

-1256;0,97;-0,093;2

-1223;0,767;0,101;2

-1178;0,189;-0,034;2

-1129;0,255;0,011;2

-1072;0,338;0,103;2

-1014;0,251;-0,08;2

-945;0,066;0,052;2

-879;-0,292;0,028;2

-803;-0,423;-0,039;2

-727;-0,529;0,121;2

-650;-0,245;-0,109;2

-582;-0,087;0,058;2

-524;-0,41;-0,034;2

-505;-0,185;-0,116;2

-528;-0,076;0,084;2

-589;0,436;-0,079;2

-664;0,717;-0,003;2

-752;0,349;0,023;2

-832;0,532;-0,05;2

-908;0,571;0,094;2

-966;0,197;-0,035;2

-1008;0,272;0,034;2

-1041;0,434;0,09;2

-1062;0,704;-0,147;2

-1085;0,793;0,063;2

-1103;0,585;-0,055;2

-1122;0,878;-0,031;2

-1145;0,661;0,073;2

-1166;0,441;-0,017;2

-1191;0,087;0,045;2

-1210;-0,198;-0,027;2

-1232;-0,309;-0,113;2

-1249;-0,766;0,141;2

-1263;-0,723;-0,06;2

-1273;-0,08;-0,005;2

-1286;0,255;0,02;2

-1295;0,672;-0,07;2

-1306;0,551;0,118;2

-1314;0,102;-0,067;2

-1322;-0,3;-0,007;2

-1320;-0,518;0,065;2

-1304;-0,643;-0,068;2

-1277;-0,798;0,026;2

-1237;-0,307;0,043;2

-1194;0,279;0,027;2

-1148;0,324;0,157;2

-1105;0,404;-0,111;2

-1054;0,27;0,077;2

-997;0,145;-0,018;2

-929;0,603;-0,037;2

-858;0,324;0,091;2

-779;-0,114;-0,081;2

-706;-0,086;0,052;2

-632;-0,351;-0,005;2

-564;-0,052;-0,069;2

-509;-0,451;0,056;2

-494;-0,998;-0,105;2

-522;-0,714;-0;2

-588;-0,621;0,014;2

-668;-0,276;-0,062;2

-755;-0,103;0,059;2

-833;-0,127;-0,048;2

-894;-0,02;-0,027;2

-949;-0,215;0,071;2

-992;-0,343;-0,128;2

-1029;-0,571;0,07;2

-1059;-0,811;-0,022;2

-1081;-0,785;-0,027;2

-1104;-1,203;0,021;2

-1124;-0,609;-0,038;2

-1151;-0,629;-0,002;2

-1176;-1,025;-0,018;2

-1200;0,06;-0,097;2

-1216;0,078;0,059;2

-1227;-0,134;-0,048;2

-1234;-0,211;0,04;2

-1242;-0,503;0,002;2

-1250;-0,477;-0,072;2

-1267;-0,46;0,086;2

-1280;-0,31;-0,035;2

-1294;-0,255;-0,022;2

-1306;-0,387;0,008;2

-1310;0,178;-0,066;2

-1297;0,374;0,108;2

-1268;0,37;-0,031;2

-1220;0,454;0,024;2

-1167;0,499;0,056;2

-1112;0,676;-0,036;2

-1063;0,547;0,012;2

-1017;0,41;-0,038;2

-967;0,231;-0,019;2

-909;-0,478;-0;2

-839;-0,228;-0,17;2

-766;-0,281;0,073;2

-688;-0,387;0,038;2

-610;-0,088;-0,075;2

-543;-0,054;0,126;2

-499;0,026;-0,053;2

-489;0,413;0,068;2

-533;0,335;0,004;2

-606;0,149;-0,055;2

-693;-0,104;0,107;2

-771;-0,503;-0,032;2

-837;-0,36;-0,014;2

-886;-0,419;0,131;2

-930;-0,663;-0,028;2

-972;-0,461;0,077;2

-1009;-0,196;0,007;2

-1042;0,164;-0,057;2

-1067;0,181;0,07;2

-1090;0,228;-0,034;2

-1113;0,229;0,055;2

-1138;0,187;-0,009;2

-1160;0,431;-0,01;2

-1182;0,019;0,114;2

-1198;-0,255;-0,101;2

-1214;-0,193;0,044;2

-1234;-0,552;-0,037;2

-1252;0,021;-0,062;2

-1265;-0,196;0,026;2

-1275;-0,278;-0,081;2

-1281;-0,092;-0,011;2

-1288;-0,355;0,039;2

-1294;-0,325;-0,089;2

-1292;-0,523;0,051;2

-1277;-0,3;-0,015;2

-1251;-0,089;0,021;2

-1217;-0,188;0,027;2

-1180;0,171;-0,072;2

-1137;0,075;0,037;2

-1089;-0,193;-0,033;2

-1032;0,133;-0,057;2

-968;0,313;-0,006;2

-900;0,433;-0,117;2

-825;0,97;0,022;2

-751;1,328;0,044;2

-674;1,277;-0,058;2

-607;1,454;0,123;2

-552;1,221;-0,072;2

-531;0,938;0,036;2

-554;0,403;0,02;2

-617;0,452;-0,096;2

-694;-0,024;0,052;2

-779;0,285;-0,044;2

-845;0,142;0,008;2

-905;-0,169;0,054;2

-952;0,378;-0,083;2

-994;0,095;0,043;2

-1032;0,225;-0,032;2

-1064;0,606;0,022;2

-1094;0,465;0,078;2

-1117;0,875;-0,099;2

-1141;0,73;0,1;2

-1166;0,169;-0,023;2

-1187;0,169;-0,072;2

-1209;-0,006;0,087;2

-1227;-0,137;-0,101;2

-1244;0,016;0,067;2

-1257;0,237;-0,015;2

-1271;0,138;-0,045;2

-1277;0,308;0,114;2

-1285;0,41;-0,055;2

-1289;0,278;0,014;2

-1302;0,238;-0,01;2

-1310;0,251;-0,053;2

-1320;0,278;0,215;2

-1325;0,251;0,005;2

-1318;0,699;0,002;2

-1293;0,384;0,005;2

-1255;0,481;-0,074;2

-1206;0,252;0,12;2

-1152;0,178;-0,009;2

-1096;0,238;-0,078;2

-1038;-0,217;0,061;2

-972;-0,036;-0,068;2

-904;0,011;0,046;2

-835;-0,137;-0,017;2

-763;0,273;-0,087;2

-694;0,024;0,102;2

-629;0,031;-0,102;2

-569;0,414;0,05;2

-523;0,507;0,047;2

-514;0,662;-0,058;2

-543;0,554;0,091;2

-605;1,004;-0,037;2

-677;0,731;0,035;2

-752;0,111;0,108;2

-819;0,539;-0,119;2

-874;0,485;0,063;2

-929;0,367;-0,032;2

-971;0,532;-0,042;2

-1007;-0,029;0,071;2

-1033;0,291;-0,075;2

-1058;0,411;0,066;2

-1082;0,394;-0,024;2

-1108;0,493;-0,017;2

-1132;0,32;0,153;2

-1154;0,293;-0,086;2

-1170;0,513;0,03;2

-1186;0,181;0,068;2

-1200;-0,134;-0,034;2

-1217;0,184;0,032;2

-1239;0,197;0,045;2

-1256;0,014;0,053;2

-1268;-0,106;0,005;2

-1275;0,093;-0,083;2

-1281;0,087;0,088;2

-1281;-0,101;-0,057;2

-1275;0,011;0,028;2

-1256;-0,177;0,036;2

-1231;0,125;-0,109;2

-1202;0,233;0,031;2

-1168;0,25;-0,048;2

-1129;-0,038;-0,023;2

-1083;-0,016;0,089;2

-1028;-0,351;-0,117;2

-969;-0,191;0,124;2

-906;-0,186;0,01;2

-831;-0,097;0,018;2

-748;0,028;0,1;2

-666;0,477;-0,036;2

-583;0,341;0,023;2

-524;0,583;0,014;2

-494;0,991;-0,079;2

-508;0,947;0,068;2

-560;0,977;-0,047;2

-640;1,112;-0,009;2

-724;0,785;-0,074;2

-804;0,522;-0,093;2

-870;0,621;0,06;2

-918;0,338;-0,015;2

-961;0,009;-0,012;2

-997;-0,476;0,079;2

-1032;-0,305;-0,041;2

-1061;-0,076;0,026;2

-1091;-0,467;-0,03;2

-1114;-0,162;-0,056;2

-1140;-0,158;0,035;2

-1162;-0,306;-0,062;2

-1181;-0,083;0,144;2

-1197;-0,686;0,03;2

-1216;-0,823;-0,09;2

-1231;-0,733;0,064;2

-1245;-0,424;-0,054;2

-1260;-0,669;0,036;2

-1271;-0,504;0,051;2

-1280;-0,306;-0,025;2

-1286;-0,619;0,027;2

-1293;-0,709;-0,04;2

-1305;-0,625;0,027;2

-1312;-1,012;-0;2

-1308;-0,649;-0,106;2

-1292;-0,508;0,059;2

-1258;-0,699;-0,042;2

-1217;-0,548;-0,003;2

-1169;-0,614;0,059;2

-1114;-0,563;-0,037;2

-1051;-0,587;0,081;2

-984;-0,635;-0,031;2

-910;-0,075;-0,037;2

-836;-0,264;0,066;2

-763;0,066;-0,103;2

-691;0,407;0,052;2

-625;0,344;-0,039;2

-570;0,449;-0,061;2

-523;-0,117;0,055;2

-501;-0,233;-0,036;2

-518;0,019;0,003;2

-573;-0,044;0,062;2

-648;-0,13;-0,055;2

-731;-0,187;0,122;2

-807;-0,137;-0,041;2

-869;0,092;-0,002;2

-925;0,184;0,068;2

-966;0,889;-0,069;2

-1006;0,853;0,056;2

-1039;0,372;-0,056;2

-1067;0,781;-0,011;2

-1092;0,882;0,089;2

-1112;0,863;-0,065;2

-1131;0,345;0,072;2

-1151;0,016;0,037;2

-1174;-0,138;-0,029;2

-1192;-0,333;0,048;2

-1210;-0,246;-0,054;2

-1222;-0,109;0,025;2

-1238;-0,889;0,018;2

-1253;-1,21;-0,08;2

-1270;-0,889;0,159;2

-1279;-0,766;-0,08;2

-1291;-0,529;0,042;2

-1293;-0,479;0,036;2

-1296;-0,14;-0,054;2

-1290;-0,273;0,077;2

-1275;-0,397;-0,089;2

-1246;-0,177;-0,039;2

-1210;-0,621;0,059;2

-1163;-0,298;-0,071;2

-1112;-0,038;0,05;2

-1054;0,242;0,009;2

-993;0,231;0,004;2

-927;-0,216;0,061;2

-860;0,109;-0,035;2

-790;0,183;0,043;2

-718;0,41;-0,019;2

-649;0,577;-0,056;2

-580;0,285;0,117;2

-532;0,252;-0,061;2

-512;0,416;0,024;2

-534;0,282;-0,045;2

-591;0,421;-0,059;2

-668;0,289;0,004;2

-749;0,307;-0,051;2

-819;0,405;0,017;2

-883;0,212;0,018;2

-932;0,294;-0,068;2

-973;0,469;0,046;2

-1008;0,815;-0,019;2

-1038;0,83;0,011;2

-1068;0,727;0,063;2

-1094;0,776;-0,061;2

-1121;0,311;0,12;2

-1140;0,183;-0,022;2

-1162;0,047;-0,04;2

-1180;0,088;0,075;2

-1198;0,057;0,036;2

-1214;0,24;0,045;2

-1232;0,025;-0,005;2

-1244;0,145;-0,047;2

-1258;0,26;0,089;2

-1268;0,335;-0,068;2

-1282;0,263;0,01;2

-1293;0,089;0,021;2

-1300;0,256;-0,094;2

-1301;0,172;0,088;2

-1301;-0,177;0,009;2

-1301;-0,206;-0;2

-1305;-0,237;0,08;2

-1300;-0,047;-0,039;2

-1281;-0,114;0,094;2

-1244;-0,083;0,011;2

-1200;-0;-0,016;2

-1152;0,035;0,069;2

-1097;0,044;-0,073;2

-1035;-0,598;0,041;2

-973;-0,714;-0,053;2

-904;-0,425;-0,031;2

-835;-0,75;0,086;2

-771;-0,464;-0,08;2

-703;-0,667;-0,047;2

-640;-0,743;0,016;2

-582;-0,407;-0,081;2

-549;-0,576;0,041;2

-549;-0,433;-0,058;2

-587;0,28;-0,01;2

-659;0,209;0,068;2

-737;-0,106;-0,057;2

-815;0,271;0,054;2

-882;0,33;-0,048;2

-938;0,038;0,034;2

-976;-0,323;0,065;2

-1010;-0,116;-0,005;2

-1035;-0,296;0,105;2

-1066;-0,653;-0,032;2

-1092;-0,009;-0,045;2

-1125;0,359;0,04;2

-1150;0,502;-0,03;2

-1174;0,392;0,02;2

-1192;-0,255;0,043;2

-1207;-0,136;-0,081;2

-1221;-0,087;0,061;2

-1230;-0,285;-0,066;2

-1238;0,058;0,065;2

-1250;0,19;0,068;2

-1262;0,249;-0,037;2

-1276;0,357;0,066;2

-1294;0,261;-0,017;2

-1306;0,197;-0,012;2

-1317;-0,019;0,074;2

-1313;0,058;-0,031;2

-1294;0,299;0,056;2

-1261;0,276;-0,027;2

-1219;0,264;-0,06;2

-1172;0,418;0,029;2

-1125;0,774;-0,099;2

-1073;0,725;0,062;2

-1018;0,4;-0,006;2

-955;0,41;-0,028;2

-894;-0,236;0,047;2

-821;-0,346;-0,035;2

-745;-0,481;0,031;2

-666;-0,714;-0,003;2

-591;-0,193;-0,063;2

-535;-0,093;0,083;2

-516;-0,292;-0,105;2

-541;-0,171;0,015;2

-602;-0,228;0,027;2

-682;0,049;-0,065;2

-760;-0,383;0,039;2

-829;-0,557;-0,026;2

-884;-0,078;0,044;2

-934;0,171;-0,038;2

-975;0,477;-0,045;2

-1014;0,502;0,055;2

-1046;0,161;0,034;2

-1070;0,268;-0,016;2

-1090;0,312;0,115;2

-1111;0,219;-0,071;2

-1131;-0,161;0,045;2

-1151;-0,401;0,038;2

-1172;0,099;-0,11;2

-1194;-0,188;0,02;2

-1217;-0,375;-0,069;2

-1235;0,013;0,04;2

-1251;0,386;0,003;2

-1260;0,276;-0,073;2

-1264;-0,121;0,095;2

-1268;-0,182;-0,047;2

-1271;-0,125;0,003;2

-1278;-0,367;0,054;2

-1289;-0,262;-0,127;2

-1299;-0,132;0,092;2

-1306;-0,208;-0,052;2

-1303;0,16;0,156;2

-1288;-0,217;0,118;2

-1263;-0,405;-0,1;2

-1232;0,159;0,05;2

-1187;-0,015;0,038;2

-1138;-0,387;-0,075;2

-1077;-0,378;0,088;2

-1019;0,125;-0,012;2

-954;0,33;0,043;2

-888;0,017;0,036;2

-820;-0,196;-0,08;2

-749;-0,308;0,094;2

-686;-0,369;-0,041;2

-621;-0,372;0,006;2

-570;-0,317;0,038;2

-537;0,231;-0,048;2

-541;0,276;0,088;2

-588;0,23;0,002;2

-670;0,46;0,076;2

-758;0,264;0,091;2

-832;0,427;-0,057;2

-889;0,666;0,035;2

-933;0,339;-0,02;2

-974;0,641;-0,031;2

-1014;0,158;0,073;2

-1051;0,001;-0,052;2

-1084;0,095;-0,031;2

-1111;-0,007;0,004;2

-1133;-0,105;-0,044;2

-1156;-0,089;0,113;2

-1172;0,072;-0,035;2

-1190;0,313;0,003;2

-1202;0,511;0,036;2

-1219;0,897;-0,086;2

-1233;0,736;0,081;2

-1254;0,977;-0,078;2

-1267;1,038;-0,043;2

-1280;0,659;-0,011;2

-1292;0,649;-0,077;2

-1301;0,708;0,062;2

-1308;0,405;-0,13;2

-1310;0,445;-0,023;2

-1299;0,179;0,066;2

-1281;0,163;-0,076;2

-1249;0,116;0,052;2

-1215;-0,116;0,002;2

-1172;-0,056;0,011;2

-1125;-0,297;0,081;2

-1068;-0,237;-0,05;2

-1010;-0,121;0,162;2

-951;-0,143;0,004;2

-890;-0,269;-0,087;2

-825;-0,44;0,076;2

-750;-0,383;-0,058;2

-675;-0,838;-0,011;2

-605;-1,481;0,024;2

-558;-1,125;-0,048;2

-543;-0,778;0,065;2

-562;-0,538;-0;2

-617;-0,138;-0,01;2

-688;-0,065;0,082;2

-767;0,267;-0,073;2

-837;0,07;0,073;2

-896;0,075;0,008;2

-940;0,492;-0,009;2

-982;0,251;0,033;2

-1021;-0,002;-0,03;2

-1055;-0,335;0,087;2

-1084;-0,157;0,019;2

-1108;0,075;-0,045;2

-1130;-0,145;0,087;2

-1148;0,1;-0,093;2

-1173;-0,025;0,034;2

-1193;-0,173;0,001;2

-1216;0,089;-0,088;2

-1234;-0,096;0,058;2

-1248;-0,356;-0,031;2

-1260;-0,441;0,017;2

-1269;-0,791;0,017;2

-1273;-0,666;-0,102;2

-1278;-0,58;0,111;2

-1279;-0,404;-0,036;2

-1286;-0,35;0,025;2

-1292;-0,596;0,044;2

-1303;-0,203;-0,086;2

-1310;0,213;0,072;2

-1317;0,2;-0,031;2

-1308;0,181;0,022;2

-1286;-0,051;0,053;2

-1254;0,246;-0,087;2

-1209;0,174;0,097;2

-1159;-0,094;-0,031;2

-1101;0,266;-0,042;2

-1040;-0,129;0,068;2

-975;-0,118;-0,05;2

-910;-0,066;-0,021;2

-848;-0,089;0,013;2

-783;0,019;-0,094;2

-723;0,146;0,096;2

-657;-0,334;-0,066;2

-600;-0,312;-0,001;2

-551;-0,198;0,067;2

-532;-0,097;-0,076;2

-556;-0,431;0,183;2

-623;-0,426;-0,058;2

-710;0,19;0,035;2

-799;-0,095;0,02;2

-874;0,358;-0,13;2

-931;0,431;0,069;2

-973;0,272;0,022;2

-1004;0,376;-0,056;2

-1033;0,369;0,174;2

-1058;-0,011;-0,118;2

-1087;0,022;0,04;2

-1114;0,279;-0,002;2

-1140;0,088;-0,034;2

-1162;0,331;0,107;2

-1185;0,551;-0,122;2

-1200;0,417;-0,027;2

-1217;0,535;0,019;2

-1230;0,522;-0,075;2

-1246;0,107;0,056;2

-1257;-0,105;-0,035;2

-1271;-0,1;0,02;2

-1282;-0,518;0,038;2

-1292;-0,41;-0,078;2

-1299;-0,242;0,053;2

-1297;-0,274;-0,043;2

-1285;-0,04;0,011;2

-1261;-0,286;0,056;2

-1229;-0,604;-0,135;2

-1186;-0,465;0,059;2

-1139;-0,339;-0,013;2

-1079;-0,061;-0,028;2

-1013;0,111;0,06;2

-942;0,47;-0,059;2

-869;0,374;0,027;2

-795;-0,025;0,063;2

-719;0,386;-0,076;2

-648;0,437;0,096;2

-582;1,311;-0,088;2

-543;1,571;-0,029;2

-547;0,785;-0,068;2

-593;0,519;-0,065;2

-674;0,307;0,104;2

-760;0,623;-0,012;2

-838;0,795;-0,01;2

-898;0,425;0,089;2

-949;0,077;0,008;2

-988;-0,023;0,079;2

-1026;-0,509;0,012;2

-1054;-0,272;-0,021;2

-1080;-0,235;0,067;2

-1099;-0,137;-0,054;2

-1121;-0,077;0,148;2

-1143;-0,306;0,03;2

-1164;-0,409;-0,081;2

-1182;-0,36;0,07;2

-1198;-0,049;-0,095;2

-1215;-0,195;0,06;2

-1235;-0,479;0,089;2

-1251;-0,325;-0,074;2

-1269;-0,157;0,144;2

-1285;-0,051;-0,071;2

-1292;0,171;0,001;2

-1297;0,124;0,081;2

-1296;-0,059;-0,125;2

-1300;-0,492;0,116;2

-1299;-0,371;-0,019;2

-1297;0,1;-0,027;2

-1279;-0,284;0,098;2

-1249;-0,202;-0,15;2

-1206;-0,237;0,071;2

-1151;-0,22;-0,015;2

-1097;-0,028;-0,044;2

-1032;-0,304;0,071;2

-965;-0,224;-0,074;2

-894;-0,004;0,097;2

-823;-0,05;-0,004;2

-748;-0,112;-0,085;2

-668;-0,458;0,042;2

-593;-0,362;-0,072;2

-519;-0,507;0,052;2

-469;-0,786;0,038;2

-454;-0,098;-0,046;2

-494;-0,089;0,067;2

-573;-0,083;-0,036;2

-669;0,272;-0,004;2

-763;-0,26;0,026;2

-841;0,103;-0,078;2

-901;0,055;0,13;2

-947;-0,076;-0,025;2

-981;0,291;-0,038;2

-1014;0,101;0,096;2

-1038;0,268;-0,089;2

-1061;0,307;0,047;2

-1085;0,257;0,01;2

-1107;0,133;-0,115;2

-1132;-0,169;0,049;2

-1162;0,287;-0,039;2

-1189;0,463;0,085;2

-1212;0,692;-0,003;2

-1232;0,225;-0,078;2

-1247;-0,095;0,105;2

-1262;0,107;-0,075;2

-1270;0,086;0,008;2

-1276;-0,498;0,032;2

-1277;-0,186;-0,049;2

-1284;0,203;0,038;2

-1291;-0,287;0,038;2

-1304;-0,609;0,023;2

-1306;-0,489;0,05;2

-1293;0,058;-0,064;2

-1255;-0,061;0,096;2

-1204;-0,374;0,047;2

-1148;-0,223;-0,062;2

-1086;-0,168;0,069;2

-1028;-0,127;-0,081;2

-962;-0,415;0,072;2

-894;-0,183;-0,015;2

-824;0,195;-0,11;2

-754;0,247;0,063;2

-681;0,663;-0,076;2

-602;0,364;0,012;2

-534;-0,074;0,008;2

-480;0,261;0,015;2

-469;0,528;0,079;2

-506;0,344;-0,016;2

-577;0,189;0,001;2

-657;-0,108;0,079;2

-738;0,098;-0,106;2

-811;0,217;0,114;2

-876;0,311;0,011;2

-928;0,436;-0,057;2

-971;0,137;0,025;2

-1003;0,463;-0,137;2

-1034;0,32;0,052;2

-1066;-0,069;0,025;2

-1090;-0,068;0,002;2

-1108;-0,143;0,102;2

-1122;0,185;-0,016;2

-1137;0,405;0,008;2

-1158;0,386;0,028;2

-1184;0,041;-0,096;2

-1206;-0,528;0,12;2

-1225;-0,68;-0,065;2

-1237;-0,707;-0,024;2

-1252;-0,728;0,047;2

-1262;-0,562;-0,078;2

-1270;-0,327;0,06;2

-1272;0,038;0,005;2

-1275;0,108;0,002;2

-1270;-0,195;0,039;2

-1260;-0,596;-0,11;2

-1242;-1,031;0,063;2

-1221;-1,18;-0,046;2

-1188;-0,693;-0,027;2

-1154;-0,436;0,086;2

-1113;-0,21;0,011;2

-1060;-0,261;0,033;2

-1005;-0,77;0,011;2

-943;-0,51;-0,087;2

-876;-0,456;0,067;2

-804;-0,387;-0,002;2

-725;-0,287;0,029;2

-652;-0,489;0,056;2

-589;-0,24;-0,083;2

-550;-0,294;0,055;2

-544;-0,012;-0,049;2

-575;-0,133;0,008;2

-638;-0,271;0,047;2

-721;-0,411;-0,121;2

-798;-0,712;0,068;2

-868;-0,597;-0,024;2

-923;-0,527;-0,054;2

-970;-0,653;0,051;2

-1006;-0,395;-0,077;2

-1040;0,005;0,089;2

-1065;0,04;0,009;2

-1087;0,01;-0,074;2

-1106;-0,213;0,063;2

-1127;-0,01;-0,056;2

-1149;0,271;-0,018;2

-1170;0,057;-0,033;2

-1194;0,375;-0,066;2

-1215;0,201;0,117;2

-1234;0,291;-0,062;2

-1247;0,43;0,016;2

-1256;0,248;0,107;2

-1261;0,287;-0,062;2

-1270;-0,3;0,073;2

-1282;-0,458;-0,089;2

-1298;-0,304;0,026;2

-1306;-0,802;-0,022;2

-1312;-0,857;-0,064;2

-1311;-0,58;0,177;2

-1310;-0,683;-0,045;2

-1306;-0,464;-0,037;2

-1288;-0,113;0,032;2

-1263;0,161;-0,089;2

-1226;0,355;0,08;2

-1187;0,158;-0,023;2

-1146;0,148;-0,003;2

-1101;0,125;0,057;2

-1054;0,357;0,025;2

-1000;0,559;0,015;2

-940;0,074;0,039;2

-875;0,561;-0,052;2

-817;0,925;0,081;2

-758;1,168;-0,093;2

-705;1,112;-0,009;2

-653;1,063;0,007;2

-620;1,26;-0,055;2

-608;1,219;0,093;2

-636;1,134;-0,031;2

-688;1,097;-0,001;2

-761;0,616;0,031;2

-831;-0,013;-0,103;2

-893;0,062;0,109;2

-944;-0,023;-0,009;2

-991;-0,277;0,013;2

-1030;-0,37;0,047;2

-1064;-0,412;-0,029;2

-1091;-0,471;0,026;2

-1109;-0,267;0,03;2

-1131;-0,033;-0,08;2

-1150;-0,188;0,074;2

-1168;0,192;-0,097;2

-1184;0,321;0,029;2

-1201;-0,084;0,045;2

-1218;0,047;-0,058;2

-1238;0,045;0,092;2

-1254;0,048;0,006;2

-1270;-0,178;0,016;2

-1279;-0,697;0,067;2

-1287;-0,654;-0,085;2

-1291;-0,763;0,132;2

-1298;-0,871;-0,025;2

-1299;-0,746;0,02;2

-1287;-0,943;0,062;2

-1268;-0,733;-0,051;2

-1235;-0,265;0,049;2

-1199;-0,393;0,018;2

-1158;-0,405;-0,027;2

-1111;-0,41;0,076;2

-1056;-0,255;-0,093;2

-1004;-0,464;0,055;2

-951;-0,692;0,036;2

-897;-0,635;-0,091;2

-836;-0,576;0,054;2

-767;-0,425;-0,063;2

-696;-0,471;0,013;2

-629;-0,509;0,04;2

-578;-0,243;-0,052;2

-552;-0,273;0,048;2

-567;0,22;0,012;2

-611;0,567;-0,006;2

-682;0,517;0,152;2

-760;0,322;-0,087;2

-837;0,443;0,092;2

-890;0,741;0,002;2

-937;0,85;-0,004;2

-976;0,307;0,089;2

-1016;0,323;-0,077;2

-1050;0,54;0,041;2

-1083;0,251;-0,025;2

-1104;0,176;-0,038;2

-1126;-0,205;0,058;2

-1146;-0,387;-0,035;2

-1171;-0,069;0,013;2

-1191;0,399;-0,056;2

-1212;0,749;-0,061;2

-1228;0,565;0,072;2

-1242;0,576;-0,049;2

-1251;0,536;-0,022;2

-1264;0,342;0,032;2

-1270;0,192;-0,082;2

-1273;0,146;0,125;2

-1279;-0,446;-0,009;2

-1282;-0,728;-0,006;2

-1292;-1,192;0,072;2

-1300;-1,081;-0,072;2

-1308;-0,759;0,121;2

-1304;-0,867;-0,032;2

-1287;-0,62;-0,04;2

-1259;-0,662;0,092;2

-1221;-0,666;-0,058;2

-1176;-0,377;0,035;2

-1124;-0,558;0,002;2

-1070;-0,446;-0,045;2

-1017;-0,387;0,057;2

-966;-0,359;-0,048;2

-912;-0,419;0,082;2

-851;-0,548;0,014;2

-783;-0,483;-0,067;2

-718;-0,574;0,12;2

-656;-0,078;-0,044;2

-601;0,042;-0,001;2

-560;-0,268;0,034;2

-552;-0,101;-0,116;2

-581;-0,265;0,054;2

-647;-0,233;-0,006;2

-725;-0,194;0,021;2

-804;-0,459;0,108;2

-866;0,026;-0,049;2

-918;0,24;0,117;2

-961;-0,131;0,007;2

-1001;0,051;0,004;2

-1038;-0,163;0,142;2

-1067;-0,108;-0,123;2

-1091;-0,16;0,058;2

-1107;-0,391;-0,007;2

-1126;-0,265;-0,033;2

-1147;-0,576;0,005;2

-1167;-0,45;-0,061;2

-1186;-0,098;-0,009;2

-1205;-0,553;0,061;2

-1221;-0,28;-0,084;2

-1240;0,143;0,138;2

-1256;-0,002;-0,037;2

-1269;0,263;0,014;2

-1281;0,206;0,049;2

-1286;0,306;-0,066;2

-1286;0,137;0,068;2

-1270;0,29;-0,062;2

-1240;0,497;0,001;2

-1199;0,021;0,114;2

-1155;-0,132;-0,041;2

-1102;-0,244;0,039;2

-1052;-0,281;-0,029;2

-995;-0,188;-0,069;2

-937;-0,116;0,048;2

-871;0,187;-0,083;2

-807;0,058;0,006;2

-739;-0,014;0,034;2

-668;0,143;-0,044;2

-595;-0,013;0,085;2

-527;-0,045;-0,003;2

-476;-0,047;0,024;2

-468;-0,166;0,058;2

-505;-0,042;-0,057;2

-578;0,102;0,023;2

-668;0,067;-0,034;2

-754;-0,007;0,039;2

-833;-0,235;0,055;2

-897;-0,151;-0,091;2

-949;-0,493;0,06;2

-988;-0,264;0,029;2

-1016;-0,11;-0,011;2

-1040;-0,08;0,04;2

-1061;-0,085;-0,068;2

-1085;-0,21;0,022;2

-1111;-0,363;0,021;2

-1134;-0,242;-0,039;2

-1159;-0,031;0,105;2

-1184;0,095;-0,081;2

-1209;0,015;0,037;2

-1231;0,374;-0,02;2

-1247;0,656;-0,084;2

-1254;0,41;0,137;2

-1263;0,784;-0,041;2

-1267;1,244;0,007;2

-1276;0,581;0,078;2

-1284;0,585;-0,078;2

-1288;0,829;0,056;2

-1294;0,549;0,076;2

-1298;0,178;-0,018;2

-1302;-0,157;0,067;2

-1289;-0,292;-0,046;2

-1264;-0,289;0,058;2

-1220;-0,609;-0,052;2

-1169;-0,727;0,064;2

-1112;-0,565;0,106;2

-1054;-0,357;-0,069;2

-982;-0,287;0,078;2

-908;-0,395;0,002;2

-825;-0,103;-0,076;2

-742;-0,193;0,1;2

-666;-0,574;-0,092;2

-594;-0,412;0,001;2

-533;-0,11;0,067;2

-481;0,236;-0,093;2

-455;0,273;0,098;2

-472;-0,02;-0,017;2

-534;0,039;-0,019;2

-628;-0,268;0,082;2

-723;-0,214;-0,097;2

-804;-0,634;0,076;2

-870;-0,819;-0,059;2

-922;-0,477;-0,022;2

-966;-0,618;0,111;2

-1003;-0,465;-0,092;2

-1038;-0,368;0,052;2

-1070;-0,305;0,044;2

-1098;-0,027;-0,038;2

-1124;-0,083;0,063;2

-1152;-0,142;-0,068;2

-1175;0,003;0,02;2

-1197;0,178;0,022;2

-1216;0,311;-0,075;2

-1237;0,368;0,046;2

-1255;0,399;-0,026;2

-1277;0,359;-0,02;2

-1298;0,518;-0,004;2

-1310;0,43;-0,072;2

-1315;0,246;0,088;2

-1314;0,466;-0,064;2

-1317;0,255;-0,033;2

-1317;-0,331;0,058;2

-1324;-0,297;-0,087;2

-1330;-0,486;0,125;2

-1333;-0,615;0,019;2

-1318;-0,05;-0,001;2

-1290;0,242;0,108;2

-1251;0,079;-0,046;2

-1204;0,28;0,083;2

-1155;-0,059;0,027;2

-1106;-0,441;-0,092;2

-1055;-0,251;0,075;2

-996;-0,228;-0,046;2

-929;-0,497;0,162;2

-857;-0,594;0,044;2

-780;-0,077;-0,092;2

-699;0,255;0,077;2

-620;0,157;-0,08;2

-546;0,455;0,06;2

-499;0,561;0,078;2

-488;0,544;-0,1;2

-519;0,259;0,086;2

-585;0,002;-0,004;2

-672;0,022;-0,004;2

-758;-0,22;0,05;2

-834;-0,354;0,027;2

-890;-0,247;0,04;2

-936;-0,092;-0,019;2

-974;0,062;-0,033;2

-1009;-0,324;0,06;2

-1039;-0,378;-0,057;2

-1074;-0,028;0,041;2

-1102;-0,099;0,038;2

-1132;0,326;-0,011;2

-1153;0,002;0,053;2

-1167;-0,177;-0,007;2

-1179;0,138;-0,014;2

-1188;-0,055;-0,009;2

-1202;-0,056;-0,077;2

-1211;-0,084;0,181;2

-1219;0,128;-0,004;2

-1222;0,363;-0,003;2

-1214;0,046;0,04;2

-1190;-0,064;-0,1;2

-1147;-0,231;-0,024;2

-1086;-0,5;0,065;2

-1005;-0,455;-0,105;2

-920;-0,435;0,061;2

-830;-0,212;-0,05;2

-742;-0,085;0,035;2

-657;-0,247;0,001;2

-587;-0,273;-0,019;2

-528;-0,729;0,073;2

-510;-0,193;-0,052;2

-541;-0,241;0,003;2

-612;-0,193;0,038;2

-700;0,25;-0,065;2

-788;0,168;0,07;2

-864;0,042;-0,021;2

-931;0,158;-0,047;2

-984;0,288;-0,079;2

-1030;0,491;-0,08;2

-1068;0,397;-0,008;2

-1100;-0,237;-0,025;2

-1128;-0,238;0,036;2

-1155;-0,128;0,191;2

-1183;-0,025;-0,036;2

-1202;-0,093;0,047;2

-1218;-0,124;-0,022;2

-1228;-0,48;-0,081;2

-1242;-0,716;0,08;2

-1259;-0,42;-0,077;2

-1280;-0,354;0,046;2

-1300;-0,229;0,021;2

-1318;-0,194;-0,108;2

-1330;-0,577;0,031;2

-1341;-0,561;-0,033;2

-1352;-0,062;-0,015;2

-1355;0,209;0,061;2

-1355;0,144;-0,067;2

-1350;-0,156;0,073;2

-1335;-0,079;-0,03;2

-1310;0,513;0,014;2

-1271;0,835;0,035;2

-1224;0,434;-0,114;2

-1172;0,306;0,09;2

-1113;0,173;-0,06;2

-1049;0,247;0,09;2

-983;-0,183;0,047;2

-915;-0,237;-0,052;2

-840;-0,417;0,06;2

-762;-0,486;0,011;2

-687;-0,052;-0,023;2

-620;0,154;0,141;2

-588;0,224;-0,079;2

-596;-0,059;0,089;2

-650;-0,053;-0;2

-719;0,307;-0,036;2

-801;0,539;0,097;2

-869;0,694;0,002;2

-931;0,987;-0,004;2

-977;0,761;0,035;2

-1018;0,679;-0,075;2

-1051;0,769;0,006;2

-1084;0,639;-0,048;2

-1111;0,584;0,07;2

-1139;0,604;0,069;2

-1161;0,298;-0,121;2

-1184;-0,001;0,023;2

-1200;-0,145;0,022;2

-1216;-0,026;-0,064;2

-1228;-0,107;0,014;2

-1238;-0,361;-0,099;2

-1256;-0,731;0,046;2

-1274;-0,771;-0,067;2

-1290;-0,597;-0,157;2

-1296;-0,809;0,078;2

-1301;-0,472;-0,241;2

-1302;0,205;0,086;2

-1301;0,322;0,017;2

-1294;0,154;-0,093;2

-1274;-0,177;-0,036;2

-1241;0,232;-0,07;2

-1192;0,504;-0,023;2

-1143;0,194;-0,054;2

-1086;0,116;-0,119;2

-1022;0,046;0,063;2

-949;-0,125;-0,095;2

-874;-0,096;0,024;2

-794;-0,2;0,056;2

-716;-0,154;-0,117;2

-645;-0,307;-0,006;2

-582;-0,396;-0,1;2

-540;0,019;-0,046;2

-536;-0,133;0,084;2

-577;-0,229;-0,08;2

-649;0,112;0,161;2

-735;-0,356;0,721;2

-815;-0,205;-0,089;2

-884;0,184;1,621;2

-936;0,071;-0,094;2

-987;-0,083;-0,066;2

-1031;0,217;0,14;2

-1072;0,804;-0,115;2

-1104;0,603;0,154;2

-1131;0,547;-0,125;2

-1150;0,694;-0,008;2

-1171;0,186;0,076;2

-1185;0,1;-0,095;2

-1203;0,227;0,019;2

-1218;0,092;-0,017;2

-1232;0,32;-0,039;2

-1247;0,278;0,017;2

-1266;0,256;-0,002;2

-1282;0,035;0,067;2

-1295;-0,235;-0,107;2

-1304;-0,183;-0,127;2

-1310;-0,19;0,07;2

-1312;-0,095;-0,108;2

-1318;-0,412;0,085;2

-1329;-0,617;0,047;2

-1335;-0,105;-0,025;2

-1340;0,239;0,149;2

-1326;0,099;-0,051;2

-1291;0,245;0,01;2

-1245;0,408;0,056;2

-1186;0,587;-0,057;2

-1126;0,486;0,061;2

-1061;0,001;-0,041;2

-993;-0,004;-0,091;2

-917;-0,034;0,116;2

-843;0,545;-0,031;2

-764;0,486;0,145;2

-686;-0,03;-0,038;2

-609;-0,06;-0,054;2

-557;-0,019;0,095;2

-534;-0,252;-0,038;2

-548;-0,237;0,027;2

-596;-0,325;-0,018;2

-669;-0,063;-0,049;2

-745;0,05;0,113;2

-818;0,046;-0,03;2

-873;0,038;-0,046;2

-920;0,068;0,011;2

-954;0,451;-0,091;2

-992;0,245;-0,022;2

-1026;0,21;0,07;2

-1059;0,381;0,003;2

-1082;0,14;0,011;2

-1106;0,307;-0,052;2

-1132;0,208;0,049;2

-1161;-0,146;-0,026;2

-1184;-0,036;-0,013;2

-1205;0,21;0,078;2

-1218;0,043;-0,096;2

-1231;-0,268;0,048;2

-1242;-0,527;0,01;2

-1252;-0,094;0,016;2

-1266;-0,034;-0,028;2

-1274;-0,011;-0,041;2

-1282;0,301;0,041;2

-1279;0,194;-0,005;2

-1262;0,161;-0,071;2

-1225;0,099;0,101;2

-1179;0,118;0,054;2

-1120;0,007;0,019;2

-1051;-0,053;-0,011;2

-980;0,308;-0,067;2

-901;-0,045;0,112;2

-823;0,002;0,03;2

-740;0,309;0,044;2

-668;-0,012;0,053;2

-610;-0,019;-0,092;2

-585;-0,101;0,093;2

-592;-0,159;-0,051;2

-628;0,134;-0,003;2

-674;0,347;0,099;2

-724;0,287;-0,123;2

-773;0,031;0,027;2

-819;0,11;0,027;2

-856;0,255;-0,079;2

-891;0,147;-0,066;2

-921;0,181;-0,016;2

-953;0,204;0,055;2

-984;-0,007;0,004;2

-1014;0,209;-0,079;2

-1040;0,244;0,083;2

-1070;0,276;-0,036;2

-1092;0,263;-0,044;2

-1117;-0,201;-0,011;2

-1137;-0,287;-0,103;2

-1154;-0,22;0,084;2

-1169;-0,215;-0,017;2

-1184;0,165;-0,014;2

-1199;0,194;0,023;2

-1220;-0,294;-0,093;2

-1244;-0,113;0,06;2

-1265;0,118;0,041;2

-1283;0,066;-0,051;2

-1296;-0,263;0,134;2

-1307;-0,39;-0,027;2

-1307;-0,424;0,084;2

-1306;-0,318;0,048;2

-1305;-0,011;-0,087;2

-1304;0,135;0,183;2

-1309;0,257;-0,052;2

-1315;0,205;0,017;2

-1325;0,027;0,107;2

-1335;0,382;-0,154;2

-1337;0,401;0,03;2

-1330;0,162;0,054;2

-1312;0,107;-0,029;2

-1276;0,004;0,036;2

-1233;0,226;-0,032;2

-1182;0,028;0,199;2

-1128;0,131;-0,028;2

-1076;0,377;0,053;2

-1023;0,008;0,08;2

-972;-0,08;-0,064;2

-911;0,054;-0,02;2

-851;-0,068;0,023;2

-785;0,026;-0,046;2

-717;-0,021;0,079;2

-648;-0,009;-0,09;2

-596;-0,266;0,101;2

-580;-0,167;0,056;2

-608;0,405;-0,036;2

-662;0,319;0,031;2

-736;0,271;-0,142;2

-805;0,367;0;2

-871;0,339;0,036;2

-925;0,427;-0,053;2

-974;0,219;0,015;2

-1010;0,098;-0,091;2

-1040;0,618;-0,03;2

-1058;0,774;0,027;2

-1080;1,018;-0,089;2

-1104;1,152;0,014;2

-1131;0,939;0,002;2

-1156;0,894;0,023;2

-1178;1,014;0,152;2

-1202;0,85;-0,151;2

-1221;0,473;0,045;2

-1244;0,301;-0,019;2

-1261;0,153;-0,047;2

-1276;-0,841;0,034;2

-1287;-0,87;-0,099;2

-1292;-0,633;0,015;2

-1295;-0,884;0,086;2

-1291;-0,824;-0,148;2

-1280;-0,618;0,067;2

-1253;-0,707;-0,068;2

-1219;-0,676;-0,012;2

-1179;-0,606;-0,036;2

-1139;-0,236;-0,043;2

-1093;0,022;0,082;2

-1047;-0,144;-0,029;2

-987;-0,134;-0,037;2

-925;-0,018;0,108;2

-854;-0,002;-0,09;2

-784;-0,305;0,07;2

-712;-0,233;0,021;2

-644;-0,142;-0,073;2

-581;-0,401;0,053;2

-538;-0,427;-0,007;2

-531;-0,265;0,048;2

-564;0,016;0,021;2

-624;0,248;-0,035;2

-694;-0,081;0,119;2

-767;-0,001;-0,064;2

-833;0,308;0,068;2

-895;0,288;0,031;2

-950;0,573;-0,109;2

-995;0,7;0,096;2

-1035;0,386;-0,049;2

-1072;0,287;-0,011;2

-1100;-0,063;0,013;2

-1126;-0,203;-0,101;2

-1148;-0,489;0,079;2

-1172;-0,229;-0,003;2

-1194;0,113;-0,007;2

-1217;0,155;0,075;2

-1240;0,068;-0,059;2

-1257;-0,012;-0,046;2

-1275;-0,008;0,087;2

-1286;0,123;0,015;2

-1294;-0,112;0,084;2

-1299;-0,599;-0,059;2

-1298;-1,091;0,019;2

-1289;-0,659;0,018;2

-1273;-0,687;-0,038;2

-1250;-0,792;0,043;2

-1218;-0,695;-0,058;2

-1183;-0,338;-0,006;2

-1137;-0,269;0,044;2

-1090;-0,037;-0,002;2

-1038;0,166;0,102;2

-985;-0,096;-0,033;2

-925;-0,168;0,016;2

-861;-0,305;0,022;2

-790;-0,164;-0,086;2

-718;-0,12;0,098;2

-642;0,204;0,001;2

-579;0,611;0,008;2

-540;0,09;0,092;2

-539;0,134;-0,104;2

-573;0,124;-0,013;2

-639;0,084;0,006;2

-717;0,483;-0,112;2

-796;0,326;0,103;2

-867;0,411;-0,1;2

-926;0,907;0,035;2

-969;0,927;0,051;2

-1005;1,005;-0,062;2

-1040;0,802;0,04;2

-1070;0,783;-0,085;2

-1102;1,074;-0,002;2

-1132;0,962;-0,003;2

-1161;1,078;-0,094;2

-1185;0,84;0,067;2

-1213;0,479;0,031;2

-1233;0,456;0,002;2

-1253;0,275;0,053;2

-1266;0,469;-0,059;2

-1273;0,559;0,149;2

-1283;0,246;-0,065;2

-1293;0,109;-0,032;2

-1305;-0,02;0,075;2

-1307;-0,232;-0,103;2

-1304;-0,294;0,064;2

-1287;-0,322;-0,009;2

-1262;-0,251;-0,081;2

-1234;-0,146;0,073;2

-1199;0,13;-0,062;2

-1160;-0,048;-0,017;2

-1113;-0,485;0,048;2

-1060;-0,456;-0,094;2

-996;-0,496;0,068;2

-928;-0,47;-0,042;2

-851;-0,314;-0,033;2

-774;-0,268;0,073;2

-694;-0,39;-0,06;2

-624;-0,558;0,079;2

-566;-0,284;0,055;2

-538;-0,368;-0,017;2

-544;-0,909;0,099;2

-588;-0,863;-0,043;2

-651;-0,44;-0,004;2

-725;-0,221;0,005;2

-792;-0,007;-0,049;2

-854;0,298;0,049;2

-906;0,419;-0,044;2

-955;0,358;0,016;2

-996;-0,034;0,043;2

-1030;0,206;-0,096;2

-1057;0,217;0,071;2

-1086;0,206;-0,046;2

-1116;0,316;0,017;2

-1151;-0,164;0,05;2

-1185;-0,168;-0,064;2

-1217;-0,294;0,06;2

-1239;-0,719;-0,029;2

-1249;-0,812;0,042;2

-1262;-0,823;0,056;2

-1268;-0,387;-0,033;2

-1276;-0,278;0,061;2

-1283;-0,55;0,027;2

-1289;-0,273;-0,049;2

-1298;-0,213;0,103;2

-1302;-0,193;-0,081;2

-1309;-0,183;0,086;2

-1310;0,164;-0,022;2

-1304;0,264;-0,028;2

-1287;-0,213;0,088;2

-1260;-0,325;-0,045;2

-1222;-0,095;0,038;2

-1180;-0,339;0,024;2

-1131;-0,236;-0,047;2

-1082;-0,076;0,109;2

-1032;-0,31;-0,017;2

-976;-0,234;-0,036;2

-916;-0,228;0,011;2

-848;-0,159;-0,07;2

-778;-0,293;0,091;2

-705;-0,342;0,018;2

-643;0,005;-0,077;2

-593;0,096;0,11;2

-570;0,342;0,024;2

-581;0,349;-0,002;2

-622;0,344;0,048;2

-686;0,503;-0,033;2

-764;0,532;0,098;2

-836;0,844;-0,091;2

-901;1,065;0,018;2

-952;0,937;0,021;2

-993;1,247;-0,099;2

-1022;0,985;0,106;2

-1054;0,522;-0,079;2

-1075;0,238;-0,015;2

-1102;0,03;0,134;2

-1123;-0,001;-0,063;2

-1147;0,15;0,083;2

-1173;0,275;-0,02;2

-1202;0,254;-0,015;2

-1232;0,239;0,102;2

-1257;0,207;-0,033;2

-1273;0,072;0,061;2

-1277;0,033;0,044;2

-1280;0,213;-0,079;2

-1285;0,261;0,084;2

-1286;0,417;-0,062;2

-1287;0,488;0,099;2

-1285;0,297;0,021;2

-1290;0,272;-0,093;2

-1299;0,369;0,071;2

-1316;0,412;0,008;2

-1322;0,47;0,048;2

-1319;-0,146;0,004;2

-1294;0,154;-0,024;2

-1257;0,13;0,059;2

-1206;-0,366;-0,013;2

-1148;-0,437;0,048;2

-1088;-0,515;0,035;2

-1030;-1,224;-0,142;2

-965;-1,574;0,152;2

-906;-1,503;0,026;2

-846;-1,315;-0,022;2

-788;-1,791;0,069;2

-728;-1,534;-0,033;2

-668;-1,335;0,008;2

-616;-1,302;-0,005;2

-586;-0,903;-0,042;2

-586;-0,779;0,092;2

-619;-0,804;-0,049;2

-677;-0,759;-0,004;2

-753;-0,792;-0,02;2

-830;-0,679;-0,072;2

-899;-0,607;0,078;2

-955;-0,465;-0,05;2

-997;-0,316;0,034;2

-1031;-0,307;0,045;2

-1059;0,072;-0,055;2

-1086;0,209;0,086;2

-1108;0,3;0,008;2

-1132;0,267;0,005;2

-1150;0,346;0,135;2

-1171;0,749;-0,087;2

-1194;0,621;0,045;2

-1216;0,467;0,022;2

-1236;0,756;0,004;2

-1249;0,728;0,094;2

-1261;0,825;-0,085;2

-1269;0,772;0,002;2

-1277;0,568;-0,036;2

-1282;0,626;-0,021;2

-1291;0,305;0,077;2

-1300;0,265;0,078;2

-1308;-0,128;0,029;2

-1305;-0,089;0,021;2

-1285;-0,019;-0,054;2

-1247;-0,564;0,074;2

-1208;-0,828;-0,001;2

-1167;-0,944;-0,014;2

-1121;-1,89;0,043;2

-1067;-2,359;-0,146;2

-998;-1,661;0,026;2

-927;-1,179;-0,049;2

-855;-0,752;0,014;2

-789;-0,867;0,083;2

-719;-0,545;0,006;2

-650;-0,567;0,045;2

-585;-0,411;0,078;2

-544;-0,341;0,003;2

-541;-0,582;0,078;2

-578;-0,817;-0,061;2

-639;-0,682;0,053;2

-715;-0,444;0,049;2

-785;-0,434;-0,037;2

-851;-0,104;-0,009;2

-910;0,173;-0,078;2

-959;0,074;0,019;2

-996;-0,289;0,052;2

-1027;-0,429;-0,177;2

-1053;-0,278;0,063;2

-1081;0,095;-0,16;2

-1113;0,22;-0,011;2

-1144;0,293;0,086;2

-1171;0,541;-0,088;2

-1191;0,323;0,057;2

-1209;0,199;0,049;2

-1225;0,122;-0,037;2

-1244;-0,311;0,045;2

-1260;-0,535;-0,08;2

-1273;-0,703;0,047;2

-1284;-0,829;-0,022;2

-1291;-0,655;-0,071;2

-1298;-0,782;0,065;2

-1304;-0,345;-0,037;2

-1310;-0,219;-0,011;2

-1308;-0,613;0,021;2

-1299;-0,258;-0,081;2

-1271;-0,334;0,116;2

-1234;-0,294;-0,047;2

-1188;-0,138;0,037;2

-1133;-0,701;-0,014;2

-1072;-0,813;-0,106;2

-1012;-0,833;0,019;2

-949;-0,761;0,083;2

-890;-0,598;0,004;2

-821;-0,28;0,07;2

-747;-0,162;-0,107;2

-666;-0,158;0,089;2

-599;0,283;-0,023;2

-554;0,364;0,011;2

-549;0,115;0,063;2

-586;0,563;-0,078;2

-650;0,731;0,092;2

-725;0,466;0,081;2

-797;0,47;-0;2

-861;0,317;0,081;2

-916;0,424;-0,081;2

-962;0,355;0,025;2

-1004;0,763;0,01;2

-1040;1,199;-0,069;2

-1071;1,388;0,1;2

-1104;1,665;-0,059;2

-1130;1,836;0,034;2

-1157;2,172;0,074;2

-1173;2,404;-0,093;2

-1188;2,614;0,05;2

-1201;2,482;-0,064;2

-1217;2,162;0,05;2

-1239;2,231;0,166;2

-1262;2,462;-0,042;2

-1289;2,37;0,013;2

-1309;1,859;-0,023;2

-1323;1,756;-0,029;2

-1327;1,413;0,047;2

-1323;1,066;-0,05;2

-1314;1,237;0,06;2

-1308;1,245;-0;2

-1299;1,284;-0,04;2

-1288;0,908;0,071;2

-1267;0,542;-0,049;2

-1239;0,548;-0,032;2

-1208;-0,046;0,049;2

-1169;-0,173;0,02;2

-1118;-0,216;0,063;2

-1052;-0,497;-0,059;2

-979;-0,976;-0,006;2

-902;-1,874;0,076;2

-829;-2,168;-0,055;2

-759;-2,206;0,073;2

-697;-2,247;-0,017;2

-632;-2,348;-0,002;2

-579;-1,836;0,092;2

-548;-1,374;-0,077;2

-559;-1,154;0,016;2

-608;-1,155;0,014;2

-680;-0,569;0,027;2

-754;-0,605;0,101;2

-830;-0,56;-0,06;2

-897;-0,659;-0,022;2

-956;-0,61;0,104;2

-1002;-0,24;-0,051;2

-1039;0,107;0,06;2

-1063;-0,037;-0,051;2

-1084;-0,411;0,023;2

-1110;-0,547;0,006;2

-1132;-0,133;0,011;2

-1152;-0,329;0,111;2

-1166;-0,505;-0,03;2

-1180;-0,47;0,069;2

-1202;-0,91;0,055;2

-1225;-0,703;-0,089;2

-1248;-0,329;-0,015;2

-1258;-0,433;-0,013;2

-1267;-0,438;-0,039;2

-1275;-0,675;0,074;2

-1285;-0,961;-0,034;2

-1295;-0,587;0,036;2

-1304;-0,222;0,006;2

-1307;0,233;-0,108;2

-1307;-0,163;0,009;2

-1292;-0,526;-0,087;2

-1268;-0,148;0,039;2

-1230;-0,291;-0,001;2

-1182;-0,394;-0,096;2

-1126;-0,524;0,158;2

-1062;-0,855;-0,067;2

-997;-0,737;0,005;2

-930;-0,417;-0,038;2

-863;-0,114;-0,102;2

-795;-0,119;0,084;2

-728;0,067;0,074;2

-658;0,183;-0,016;2

-593;0,167;0,042;2

-540;0,341;-0,085;2

-522;0,435;0,015;2

-546;0,377;-0,049;2

-616;0,753;-0,035;2

-698;0,981;0,087;2

-780;1,329;-0,062;2

-849;1,406;0,082;2

-906;1,214;-0,041;2

-952;1,145;-0,067;2

-998;0,611;0,084;2

-1034;0,533;-0,066;2

-1061;0,703;-0;2

-1087;0,481;0,04;2

-1106;0,587;-0,065;2

-1134;0,684;0,072;2

-1158;0,289;-0,052;2

-1184;0,524;-0,045;2

-1203;0,481;0,067;2

-1214;0,216;-0,086;2

-1226;0,089;0,086;2

-1234;0,284;-0,036;2

-1244;0,446;-0,008;2

-1256;0,34;0,018;2

-1274;0,107;-0,08;2

-1290;0,016;0,061;2

-1308;0,185;0,002;2

-1314;0,428;-0,014;2

-1314;0,242;0,114;2

-1300;0,182;-0,075;2

-1278;0,537;0,02;2

-1246;0,463;-0,009;2

-1208;0,364;-0,119;2

-1164;-0,18;0,071;2

-1114;-0,075;-0,075;2

-1057;0,13;0,073;2

-998;-0,367;0,038;2

-934;-0,201;-0,043;2

-866;-0,536;0,071;2

-795;-0,503;-0,038;2

-719;-0,078;0,027;2

-645;-0,144;0,102;2

-584;-0,069;-0,02;2

-550;0,186;0,081;2

-552;0,441;-0,005;2

-591;0,51;-0,057;2

-655;0,097;0,104;2

-727;0,094;-0,074;2

-794;-0,025;-0,008;2

-865;-0,06;-0,065;2

-922;0,485;-0,033;2

-974;0,644;0,052;2

-1013;0,535;-0,044;2

-1043;0,355;0,024;2

-1069;0,652;0,029;2

-1089;0,97;-0,093;2

-1114;0,518;0,053;2

-1134;0,359;0,046;2

-1158;0,308;0,036;2

-1181;0,513;0,044;2

-1202;0,717;-0,088;2

-1224;0,148;0,045;2

-1239;0,063;-0,014;2

-1248;0,223;-0,067;2

-1253;-0,162;0,072;2

-1259;-0,343;-0,031;2

-1265;-0,303;0,063;2

-1270;0,027;0,016;2

-1273;0,1;-0,081;2

-1279;0,043;0,084;2

-1281;0,119;-0,055;2

-1284;0,179;0,1;2

-1279;-0,088;0,125;2

-1263;-0,139;0,002;2

-1235;-0,36;0,047;2

-1195;-0,668;-0,019;2

-1145;-0,937;0,011;2

-1094;-0,524;-0,001;2

-1040;-0,212;-0,083;2

-981;-0,596;0,063;2

-925;-0,715;-0,026;2

-861;-0,365;0,064;2

-796;-0,205;0,065;2

-726;0,013;-0,089;2

-655;0,367;0,029;2

-596;0,302;-0,04;2

-560;0,276;-0,054;2

-554;0,075;0,069;2

-590;-0,054;-0,049;2

-652;0,233;0,03;2

-730;0,458;-0,033;2

-800;0,649;-0,057;2

-862;0,65;0,117;2

-914;0,684;-0,05;2

-950;0,627;0,001;2

-989;0,38;-0,015;2

-1025;0,407;-0,045;2

-1068;0,208;0,049;2

-1112;0,111;-0,097;2

-1144;-0,051;0,055;2

-1171;-0,616;0,029;2

-1184;-0,606;-0,074;2

-1195;-0,761;0,084;2

-1206;-0,82;-0,056;2

-1221;-0,296;-0,003;2

-1231;0,175;0,07;2

-1246;0,306;-0,132;2

-1259;0,367;0,041;2

-1274;0,108;-0;2

-1286;0,213;0,085;2

-1297;-0,265;0,143;2

-1299;-0,334;-0,027;2

-1296;-0,08;0,053;2

-1289;0,057;0,049;2

-1269;0,202;-0,168;2

-1237;0,076;0,105;2

-1196;0,128;-0,098;2

-1147;0,459;-0,026;2

-1097;0,479;0,009;2

-1046;0,632;-0,059;2

-996;0,653;0,114;2

-940;0,204;0,001;2

-876;-0,324;-0,013;2

-803;-0,586;0,07;2

-724;-0,007;-0,084;2

-643;0,231;0,066;2

-561;-0,051;-0,026;2

-494;0,398;-0,043;2

-453;0,587;0,055;2

-460;0,779;-0,082;2

-513;0,799;0,087;2

-600;0,757;0,027;2

-690;1,215;-0,032;2

-773;1,267;0,018;2

-836;1,172;-0,101;2

-890;0,917;0,036;2

-938;0,538;0,005;2

-980;0,603;-0,056;2

-1014;0,501;0,131;2

-1040;0,602;0,038;2

-1065;0,667;0,014;2

-1086;0,217;0,079;2

-1112;0,149;-0,087;2

-1136;0,202;0,085;2

-1156;0,219;-0,05;2

-1174;0,312;-0,008;2

-1192;0,111;-0,011;2

-1213;0,048;-0,093;2

-1232;-0,063;0,044;2

-1249;-0,42;-0,006;2

-1252;-0,72;-0,027;2

-1255;-0,625;0,101;2

-1257;-0,505;-0,061;2

-1265;-0,397;0,024;2

-1275;-0,433;0,017;2

-1281;-0,294;-0,079;2

-1276;-0,475;0,089;2

-1266;-0,761;-0,057;2

-1238;-0,719;0,152;2

-1205;-0,366;0,052;2

-1161;0,114;-0,007;2

-1110;0,414;0,039;2

-1055;0,5;-0,004;2

-998;0,283;0,025;2

-933;-0,182;0,1;2

-862;-0,032;-0,056;2

-780;0,155;0,099;2

-702;-0,155;-0,025;2

-633;-0,011;0,006;2

-576;0,085;-0,006;2

-537;0,069;-0,056;2

-529;-0,301;-0,045;2

-562;-0,411;0,039;2

-625;-0,299;-0,085;2

-704;-0,193;0,072;2

-782;0,418;-0,073;2

-853;0,251;0,055;2

-911;-0,275;0,004;2

-960;0,018;0,005;2

-1006;0,013;0,076;2

-1043;0,124;0,038;2

-1077;0,108;0,022;2

-1099;-0,467;0,025;2

-1122;-0,225;-0,114;2

-1147;-0,106;0,124;2

-1170;-0,099;-0,095;2

-1192;0,062;-0;2

-1209;-0,047;0,085;2

-1218;0,099;-0,052;2

-1226;0,349;0,018;2

-1236;-0,02;-0,011;2

-1251;-0,263;-0,017;2

-1269;-0,4;0,088;2

-1281;-0,176;-0,063;2

-1293;0,127;0,007;2

-1298;0,191;-0,012;2

-1307;0,025;-0,082;2

-1311;0,116;0,133;2

-1304;0,475;-0,085;2

-1278;0,333;-0,005;2

-1238;-0,032;0,013;2

-1189;0,181;-0,053;2

-1138;0,394;0,07;2

-1078;0,758;-0,047;2

-1019;0,959;0,015;2

-948;0,845;0,058;2

-876;0,975;-0,07;2

-808;0,874;0,048;2

-737;1,265;-0,024;2

-671;1,167;-0,01;2

-600;0,729;0,059;2

-541;0,716;-0,135;2

-513;0,783;0,09;2

-532;0,865;-0,028;2

-587;0,797;-0,042;2

-668;1,021;0,038;2

-742;1,266;-0,056;2

-812;0,964;0,001;2

-873;0,744;-0,013;2

-929;0,969;0,006;2

-983;0,596;0,078;2

-1022;0,63;-0,052;2

-1054;1,048;0,069;2

-1079;0,531;0,052;2

-1106;0,3;-0,069;2

-1131;0,405;0,043;2

-1147;0,321;-0,078;2

-1158;0,227;0,026;2

-1169;0,133;0,017;2

-1187;0,256;-0,059;2

-1214;0,259;0,089;2

-1245;0,485;-0,011;2

-1266;0,439;-0,019;2

-1282;0,501;0,034;2

-1290;0,593;-0,084;2

-1298;0,667;0,057;2

-1305;0,317;-0,008;2

-1308;0,385;-0,037;2

-1304;0,437;0,043;2

-1299;0,112;0,035;2

-1281;0,14;-0,015;2

-1260;0,393;0,095;2

-1227;0,277;-0,102;2

-1190;0,064;0,071;2

-1143;-0,062;-0,096;2

-1098;-0,533;-0,031;2

-1042;-1,056;-0,01;2

-977;-0,688;-0,051;2

-904;-0,239;0,033;2

-828;0,134;-0,043;2

-748;0,352;-0,059;2

-678;-0,044;0,049;2

-616;-0,465;-0,073;2

-575;-0,749;0,073;2

-559;-0,626;-0,02;2

-580;-0,271;0,009;2

-634;-0,123;0,014;2

-703;-0,178;-0,072;2

-774;0,166;0,028;2

-836;-0,153;0,02;2

-892;-0,041;-0,104;2

-938;0,02;0,063;2

-983;-0,006;-0,023;2

-1028;-0,204;0,037;2

-1065;-0,475;0,026;2

-1098;-0,574;-0,106;2

-1123;-0,539;0,059;2

-1144;-0,371;-0,075;2

-1160;-0,2;0,122;2

-1176;-0,306;0,04;2

-1188;-0,316;-0,084;2

-1210;0,2;0,028;2

-1232;0,665;0,062;2

-1262;0,783;0,003;2

-1286;0,458;0,049;2

-1296;0,544;-0,04;2

-1293;0,383;0,086;2

-1291;-0,172;-0,016;2

-1292;-0,453;-0,015;2

-1303;-0,429;0,06;2

-1316;-0,397;-0,047;2

-1326;-0,168;0,011;2

-1332;-0,388;0,043;2

-1329;0,058;-0,098;2

-1315;0,383;0,092;2

-1284;0,685;-0,068;2

-1240;0,802;-0,001;2

-1181;0,843;0,016;2

-1120;1,013;-0,043;2

-1061;1,12;0,082;2

-1005;1,243;-0,057;2

-939;1,343;-0,025;2

-866;1,017;0,053;2

-781;1,031;-0,057;2

-697;0,945;-0,031;2

-626;0,997;-0,03;2

-570;1,492;-0,029;2

-543;0,867;0,055;2

-559;0,598;-0,084;2

-610;0,419;0,061;2

-685;0,117;-0,014;2

-763;-0,04;-0,014;2

-831;-0,273;0,102;2

-888;0,11;-0,081;2

-934;0,078;0,065;2

-980;-0,007;0,003;2

-1024;-0,136;-0,072;2

-1066;-0,753;0,102;2

-1104;-0,865;-0,034;2

-1129;-0,293;-0,02;2

-1153;-0,33;0,046;2

-1169;-0,287;-0,101;2

-1187;-0,522;0,066;2

-1200;-0,564;-0,006;2

-1213;-0,786;0,021;2

-1227;-0,553;0,036;2

-1249;-0,029;-0,04;2

-1271;0,022;0,075;2

-1296;-0,011;-0,064;2

-1312;0,153;-0,039;2

-1322;0,03;0,099;2

-1322;-0,057;-0,094;2

-1318;-0,008;0,039;2

-1304;0,304;-0,016;2

-1278;0,49;-0,073;2

-1247;0,205;0,027;2

-1212;0,127;-0,064;2

-1179;0,108;0,025;2

-1140;0,231;0,047;2

-1090;0,597;-0,08;2

-1027;0,186;0,16;2

-961;0,098;-0,03;2

-888;0,317;0;2

-817;0,035;0,029;2

-742;-0,217;-0,05;2

-672;-0,037;0,118;2

-605;0,068;-0,041;2

-555;0,377;-0,051;2

-537;0,216;0,043;2

-567;0,18;-0,066;2

-630;-0,364;0,072;2

-705;-0,823;-0,002;2

-786;-0,481;-0,019;2

-856;-0,331;0,058;2

-921;-0,383;-0,021;2

-973;-0,437;0,077;2

-1011;-0,469;0,036;2

-1038;-0,212;-0,081;2

-1059;-0,224;0,144;2

-1085;-0,201;-0,084;2

-1111;0,138;0,049;2

-1142;0,098;0,008;2

-1169;0,37;-0,038;2

-1196;0,507;0,071;2

-1216;0,303;-0,042;2

-1236;0,102;-0,043;2

-1252;-0,035;0,032;2

-1267;0,069;-0,073;2

-1272;0,175;0,118;2

-1278;0,868;-0,037;2

-1281;1,494;-0,038;2

-1289;2,063;0,105;2

-1302;2,842;-0,079;2

-1315;3,161;0,07;2

-1327;2,502;-0,019;2

-1333;0,507;-0,056;2

-1339;-0,974;-0,015;2

-1339;-1,45;-0,112;2

-1324;-1,622;0,043;2

-1290;-1,248;-0,014;2

-1249;-0,586;-0,087;2

-1207;0,081;0,078;2

-1168;0,173;-0,108;2

-1124;-0,288;-0,024;2

-1076;-0,557;0,075;2

-1013;-0,403;-0,076;2

-944;-0,791;-0,003;2

-868;-0,614;-0,037;2

-800;-0,048;0,039;2

-731;-0,236;0,132;2

-663;0,28;-0,048;2

-601;0,107;0,067;2

-554;-0,246;0,083;2

-548;-0,028;-0,016;2

-582;-0,277;0,09;2

-655;-0,447;-0,075;2

-736;-0,396;0,11;2

-812;-0,385;0,007;2

-876;0,223;-0,063;2

-919;1,032;0,062;2

-954;1,232;-0,052;2

-978;0,707;0,06;2

-1012;0,198;0,009;2

-1048;0,803;-0,061;2

-1090;0,558;0,047;2

-1124;0,59;0,036;2

-1156;0,981;-0,002;2

-1174;1,523;0,027;2

-1193;1,693;-0,069;2

-1208;0,922;0,034;2

-1212;0,453;-0,037;2

-1219;0,184;-0,014;2

-1225;-0,393;0,035;2

-1240;-0,514;-0,084;2

-1265;-0,391;0,091;2

-1290;-0,304;-0,017;2

-1303;0,994;-0,062;2

-1308;0,877;0,087;2

-1298;0,036;-0,06;2

-1292;-0,654;0,009;2

-1280;-1,201;0,044;2

-1266;-0,733;-0,044;2

-1239;0,069;0,099;2

-1207;0,023;-0,068;2

-1167;-0,136;-0,003;2

-1125;0,339;-0,012;2

-1083;1,548;-0,138;2

-1034;1,351;0,037;2

-970;0,593;-0,045;2

-899;0,18;-0,016;2

-820;-0,084;0,065;2

-736;-0,323;-0,117;2

-661;-0,093;0,101;2

-589;0,538;-0,008;2

-544;0,9;0,022;2

-536;0,623;0,08;2

-570;0,128;-0,087;2

-638;-0,03;0,052;2

-713;-0,051;0,055;2

-786;-0,101;-0,04;2

-846;-0,326;0,119;2

-901;-0,54;-0,102;2

-951;-0,733;0,042;2

-992;-0,989;0,058;2

-1020;-0,051;-0,079;2

-1040;0,104;0,081;2

-1058;-0,3;0,003;2

-1084;0,01;0,012;2

-1113;0,245;0,055;2

-1139;0,287;-0,071;2

-1165;0,367;0,067;2

-1185;0,607;-0,108;2

-1209;0,898;0,092;2

-1226;0,786;0,06;2

-1239;0,197;-0,039;2

-1239;-0,323;0,077;2

-1237;-0,996;0,038;2

-1239;-0,617;-0,051;2

-1256;-0,654;0,169;2

-1273;-0,691;-0,077;2

-1295;-0,457;0,042;2

-1308;-0,819;0,006;2

-1310;-0,941;-0,102;2

-1296;-1,006;0,134;2

-1265;-0,869;-0,074;2

-1229;-0,691;0,001;2

-1190;-0,708;-0,01;2

-1157;-0,515;-0,075;2

-1129;-0,691;0;2

-1093;-0,53;-0,053;2

-1044;-0,297;-0,004;2

-975;-0,413;0,021;2

-889;-0,205;-0,108;2

-805;-0,244;0,082;2

-722;-0,232;-0,064;2

-654;-0,111;-0,038;2

-603;-0,391;0,144;2

-570;-0,773;-0,082;2

-568;-0,94;0,014;2

-594;-1,117;-0,007;2

-647;-1,148;-0,103;2

-712;-0,987;0,039;2

-777;-0,423;-0,064;2

-836;-0,141;0,012;2

-890;-0,338;0,004;2

-945;-0,492;-0,055;2

-994;-0,399;0,09;2

-1041;-0,43;-0,055;2

-1074;-0,458;0,072;2

-1097;-0,19;0,069;2

-1106;-0,095;-0,068;2

-1118;0,139;0,06;2

-1131;0,244;-0,042;2

-1150;0,034;0,058;2

-1172;-0,099;0,053;2

-1199;0,027;-0,025;2

-1221;-0,329;0,034;2

-1241;-0,403;-0,058;2

-1249;-0,226;-0,034;2

-1252;-0,273;0,073;2

-1250;-0,405;-0,076;2

-1253;-0,468;0,028;2

-1260;-0,119;-0,029;2

-1268;0,243;-0,033;2

-1274;0,37;0,063;2

-1271;0,396;-0,097;2

-1262;0,195;0,045;2

-1242;0,035;0,027;2

-1216;0,215;-0,059;2

-1179;-0,038;0,043;2

-1131;-0,058;-0,03;2

-1076;0,198;-0,002;2

-1021;0,122;0,089;2

-960;0,343;-0,027;2

-899;0,4;0,06;2

-835;0,514;-0,031;2

-758;0,339;0,019;2

-686;-0,18;0,081;2

-616;-0,159;-0,117;2

-560;0,173;0,075;2

-525;0,065;0,023;2

-527;0,342;-0,082;2

-568;-0,038;0,061;2

-639;-0,599;-0,048;2

-724;-0,526;0,075;2

-805;-0,541;-0,004;2

-871;-0,579;-0,067;2

-921;-0,585;0,092;2

-961;-0,703;-0,057;2

-992;-0,521;-0,006;2

-1020;-1,083;0,033;2

-1042;-1,112;-0,109;2

-1067;-0,793;0,078;2

-1087;-1,009;-0,069;2

-1114;-0,958;-0,021;2

-1139;-0,876;0,016;2

-1164;-0,862;-0,029;2

-1186;-0,894;0,07;2

-1207;-0,936;-0,017;2

-1220;-0,63;-0,004;2

-1232;-1,053;0,074;2

-1235;-1,086;-0,087;2

-1239;-0,83;0,034;2

-1237;-0,943;0,016;2

-1243;-0,527;-0,066;2

-1249;-0,356;0,013;2

-1251;-0,046;-0,08;2

-1244;0,219;0,038;2

-1223;0,123;0,027;2

-1186;0,272;-0,039;2

-1138;0,175;0,051;2

-1069;0,04;-0,018;2

-991;-0,061;-0,002;2

-909;-0,036;0,034;2

-829;0,36;-0,086;2

-758;0,507;0,067;2

-687;0,593;0,022;2

-625;0,701;-0,003;2

-569;0,58;0,026;2

-534;0,573;-0,007;2

-534;0,504;0,071;2

-578;0,237;0,023;2

-655;0,155;0,045;2

-740;-0,106;0,072;2

-823;-0,065;-0,041;2

-892;0,21;0,085;2

-946;0,263;0,028;2

-984;0,742;-0,074;2

-1017;0,24;0,103;2

-1041;0,235;-0,128;2

-1068;0,451;-0,036;2

-1094;0,213;0,038;2

-1119;0,401;-0,039;2

-1141;0,27;0,073;2

-1162;0,204;0,007;2

-1178;0,288;-0,047;2

-1196;-0,226;0,059;2

-1209;-0,411;-0,103;2

-1222;-0,232;0,098;2

-1237;-0,062;-0,023;2

-1247;0,251;-0,123;2

-1261;0,403;0,065;2

-1273;0,836;-0,067;2

-1282;0,946;0,09;2

-1285;0,587;-0,012;2

-1276;0,686;-0,048;2

-1247;0,762;0,007;2

-1209;0,787;-0,042;2

-1160;0,155;-0,016;2

-1108;0,062;0,007;2

-1047;0,228;-0,03;2

-983;0,287;0,15;2

-914;0,255;-0,067;2

-846;0,333;-0,008;2

-778;0,328;0,028;2

-714;0,22;-0,056;2

-653;0,381;0,049;2

-594;0,417;-0,057;2

-552;0,272;0,033;2

-541;0,074;0,114;2

-576;0,287;-0,062;2

-644;-0,138;0,131;2

-726;-0,025;-0,006;2

-808;0,127;-0,019;2

-876;-0,085;0,045;2

-940;0,212;-0,059;2

-988;-0,215;0,048;2

-1027;-0,364;0,005;2

-1056;-0,551;-0,066;2

-1081;-0,87;0,131;2

-1100;-0,54;-0,047;2

-1126;-0,616;0,03;2

-1151;-0,881;-0,012;2

-1175;-1,023;-0,085;2

-1194;-0,644;0,062;2

-1211;0,039;0,007;2

-1229;-0,019;0,036;2

-1249;-0,166;0,031;2

-1267;0,136;-0,045;2

-1283;0,097;0,114;2

-1297;0,234;-0,022;2

-1306;0,168;-0,031;2

-1311;-0,057;0,068;2

-1315;0,296;-0,076;2

-1317;0,355;0,052;2

-1312;0,164;0,02;2

-1300;0,163;0,011;2

-1272;0,116;0,016;2

-1239;-0,004;-0,05;2

-1198;0,168;0,043;2

-1154;-0,23;0,005;2

-1102;-0,42;-0,068;2

-1046;-0,444;0,107;2

-977;-0,374;-0,047;2

-908;-0,373;-0,025;2

-832;-0,129;0,037;2

-761;-0,034;-0,043;2

-691;-0,192;0,068;2

-632;-0,338;-0,077;2

-595;-0,297;-0,003;2

-587;-0,11;0,063;2

-620;0,076;-0,136;2

-676;0,093;0,069;2

-750;0,3;-0,042;2

-820;0,359;-0,011;2

-880;0,035;0,043;2

-932;-0,07;-0,112;2

-973;-0,182;0,11;2

-1013;-0,392;0,01;2

-1048;-0,447;-0,039;2

-1084;-0,594;0,041;2

-1116;-0,497;-0,055;2

-1147;-0,186;0,002;2

-1171;0,053;0,033;2

-1188;0,49;-0,101;2

-1200;0,456;0,184;2

-1216;0,27;-0,038;2

-1230;0,417;0,04;2

-1248;0,326;0,054;2

-1260;0,461;-0,061;2

-1274;0,54;0,029;2

-1278;0,539;-0,06;2

-1287;0,394;-0,004;2

-1289;-0,094;0,068;2

-1293;-0,379;-0,083;2

-1294;-0,659;0,013;2

-1288;-0,401;-0,032;2

-1271;-0,257;-0,017;2

-1238;-0,282;0,082;2

-1194;-0,187;-0,096;2

-1140;-0,2;-0,074;2

-1076;-0,589;-0,005;2

-1012;-0,972;-0,011;2

-944;-0,776;0,102;2

-869;-0,618;-0,067;2

-794;-0,644;-0,067;2

-718;-0,835;0,17;2

-652;-0,435;-0,083;2

-590;-0,304;0,124;2

-540;0,075;-0,068;2

-514;0,609;-0;2

-527;0,484;0,036;2

-580;0,192;-0,095;2

-660;0,437;0,044;2

-747;0,311;-0,03;2

-823;0,427;-0,06;2

-885;0,682;0,066;2

-932;0,686;-0,078;2

-977;0,272;0,027;2

-1012;0,336;0,114;2

-1048;0,755;-0,002;2

-1075;0,532;0,01;2

-1095;0,182;-0,047;2

-1115;0,107;-0,128;2

-1135;0,022;0,002;2

-1152;0,166;-0,051;2

-1172;0,113;0,092;2

-1188;0,139;-0,083;2

-1204;0,325;-0,081;2

-1217;-0,172;0,022;2

-1232;-0,301;-0,07;2

-1235;0,07;0,075;2

-1223;-0,213;0,018;2

-1199;-0,225;-0,013;2

-1162;0,002;0,066;2

-1118;-0,139;-0,157;2

-1071;-0,177;-0,049;2

-1014;-0,282;-0,047;2

-942;-0,302;0,003;2

-864;-0,488;0,062;2

-779;-0,35;-0,086;2

-703;-0,417;0,083;2

-631;-0,316;-0,015;2

-574;-0,031;-0,042;2

-540;-0,241;0,071;2

-547;0,092;-0,044;2

-593;0,416;0,029;2

-669;0,066;0,016;2

-745;0,516;0,036;2

-813;0,47;0,076;2

-868;0,129;-0,098;2

-915;0,067;-0,017;2

-964;0,236;-0,023;2

-1004;0,683;-0,063;2

-1043;0,511;0,067;2

-1071;0,506;0;2

-1099;0,426;0,058;2

-1124;0,028;0,063;2

-1147;0,112;-0,061;2

-1169;0,154;0,035;2

-1193;0,279;0,112;2

-1212;0,74;-0,021;2

-1232;0,222;0,084;2

-1244;0,137;-0,048;2

-1248;0,239;0,053;2

-1253;-0,178;-0,245;2

-1247;-0,267;-0,024;2

-1235;-0,278;0,115;2

-1211;-0,413;-0,103;2

-1169;-0,265;-0,159;2

-1110;-0,358;0,037;2

-1042;-0,154;-0,007;2

-963;-0,237;0,001;2

-884;-0,348;-0,039;2

-801;0,147;0,011;2

-725;-0,069;0,119;2

-650;-0,197;-0,071;2

-602;0,228;0,068;2

-591;0,579;0,056;2

-625;0,784;0,007;2

-692;1,006;0,066;2

-774;0,994;-0,083;2

-848;0,745;0,036;2

-917;0,134;-0,029;2

-971;-0,027;0,074;2

-1018;-0,646;0,012;2

-1056;-0,929;-0,053;2

-1090;-0,837;0,042;2

-1115;-0,557;-0,017;2

-1138;-0,002;-0,064;2

-1159;-0,11;0,025;2

-1181;-0,428;-0,04;2

-1202;-0,25;0,116;2

-1224;-0,292;0,183;2

-1246;-0,372;0,014;2

-1265;-0,096;0,072;2

-1283;0,072;-0,053;2

-1299;-0,112;0,027;2

-1310;-0,395;0,063;2

-1318;-0,868;-0,08;2

-1323;-0,867;0,035;2

-1330;-0,629;-0,008;2

-1332;-0,254;-0,005;2

-1338;-0,374;0,087;2

-1335;-0,529;-0,205;2

-1326;-0,686;0,196;2

-1303;-0,714;-0,005;2

-1271;-0,598;-0,109;2

-1226;-0,604;0,071;2

-1177;-0,04;-0,073;2

-1116;-0,036;0,06;2

-1055;-0,19;0,007;2

-985;-0,089;-0,084;2

-913;-0,337;-0,015;2

-837;-0,267;-0,064;2

-760;-0,034;0,012;2

-686;-0,279;-0,094;2

-624;-0,562;-0,087;2

-588;-1,025;0,057;2

-591;-0,809;-0,026;2

-633;-0,43;-0,018;2

-703;-0,712;0,083;2

-777;-0,455;-0,105;2

-846;-0,213;0,066;2

-907;0,081;-0,014;2

-954;0,158;-0,031;2

-995;-0,195;0,075;2

-1030;0,04;0,031;2

-1062;0,07;0,028;2

-1089;0,027;-0,025;2

-1119;0,394;-0,146;2

-1144;0,373;0,082;2

-1168;0,078;-0,122;2

-1187;0,132;0,004;2

-1207;-0,003;0,003;2

-1223;0,468;-0,102;2

-1238;0,207;0,103;2

-1255;0,249;-0,06;2

-1267;0,439;0,026;2

-1280;0,121;0,046;2

-1285;0,029;-0,104;2

-1289;0,222;0,103;2

-1292;0,158;-0,055;2

-1297;0,186;-0,023;2

-1303;0,144;0,019;2

-1309;0,137;-0,086;2

-1300;0,024;0,037;2

-1279;0,049;0,013;2

-1242;0,28;-0,027;2

-1200;0,054;0,131;2

-1155;-0,141;-0,082;2

-1104;-0,303;0,025;2

-1053;-0,511;0,043;2

-997;-0,572;-0,104;2

-939;-0,684;0,033;2

-877;-0,661;0,09;2

-809;-0,145;-0,005;2

-736;-0,166;0,045;2

-663;-0,033;-0,074;2

-597;0,186;0,173;2

-562;0,71;-0,053;2

-567;1,083;-0;2

-622;0,873;0,077;2

-695;1,033;-0,039;2

-774;0,876;0,051;2

-840;0,713;-0,022;2

-896;0,512;-0,029;2

-937;-0,167;0,116;2

-978;-0,339;-0,055;2

-1014;0,018;0,194;2

-1051;-0,294;-0,012;2

-1084;-0,193;-0,079;2

-1115;0,018;0,085;2

-1143;0,086;-0,074;2

-1161;-0,171;0,048;2

-1179;-0,031;-0,146;2

-1187;0,017;-0,025;2

-1200;0,356;0,052;2

-1219;0,366;-0,043;2

-1237;0,303;0,015;2

-1261;0,599;0,039;2

-1278;0,409;-0,077;2

-1292;0,175;0,024;2

-1305;0,325;-0,029;2

-1317;0,25;-0,04;2

-1324;-0,171;0,063;2

-1328;-0,17;-0,1;2

-1317;-0,188;0,056;2

-1302;-0,191;0,004;2

-1275;-0,153;-0,008;2

-1247;-0,251;0,04;2

-1207;-0,037;-0,031;2

-1160;0,197;0,069;2

-1105;0,087;-0,01;2

-1046;-0,015;-0,033;2

-985;-0,111;0,048;2

-926;0,243;-0,041;2

-860;0,401;0,005;2

-796;0,101;0,003;2

-723;0,386;-0,1;2

-651;0,643;0,035;2

-594;0,813;-0,118;2

-564;1;-0,001;2

-580;0,882;0,065;2

-633;0,899;-0,058;2

-707;0,864;0,046;2

-791;0,669;0,09;2

-870;0,086;-0,004;2

-936;-0,364;0,075;2

-990;-0,642;-0,117;2

-1027;-0,681;0,046;2

-1057;-0,055;0,058;2

-1076;0,319;-0,016;2

-1094;-0,046;0,013;2

-1116;0,156;-0,062;2

-1140;0,658;0,029;2

-1166;0,61;-0,036;2

-1190;0,361;-0,039;2

-1212;0,182;0,054;2

-1226;0,045;-0,057;2

-1239;0,056;0,037;2

-1251;0,163;0,011;2

-1263;0,289;-0,087;2

-1272;0,383;0,085;2

-1283;0,273;-0,018;2

-1288;-0,093;0,037;2

-1291;-0,21;0,096;2

-1294;0,077;-0,089;2

-1293;-0,021;0,08;2

-1275;-0,084;-0,004;2

-1247;-0,159;-0,033;2

-1210;-0,369;0,024;2

-1169;-0,394;-0,068;2

-1126;-0,173;0,045;2

-1075;0,022;-0,038;2

-1014;0,053;-0,034;2

-943;-0,137;0,078;2

-869;0,345;-0,037;2

-798;0,616;0,001;2

-738;0,523;0,015;2

-681;0,533;-0,06;2

-630;0,594;0,125;2

-587;0,994;-0,046;2

-572;1,159;-0,007;2

-586;0,587;0,018;2

-639;0,852;-0,104;2

-706;0,9;0,075;2

-779;0,82;-0,061;2

-848;0,905;-0,006;2

-911;0,756;0,036;2

-961;0,757;-0,078;2

-1005;0,728;0,079;2

-1033;0,371;-0,003;2

-1061;0,13;-0,027;2

-1085;0,216;0,119;2

-1110;0,323;-0,089;2

-1139;-0,128;0,03;2

-1159;-0,754;0,001;2

-1180;-1,513;-0,05;2

-1198;-1,537;0,08;2

-1215;-0,878;-0,008;2

-1231;-0,747;0,022;2

-1240;-0,941;0,051;2

-1251;-0,269;-0,06;2

-1261;0,283;0,146;2

-1275;0,649;-0,072;2

-1291;0,776;-0,014;2

-1305;0,544;0,016;2

-1310;0,653;0,038;2

-1316;0,381;0,101;2

-1308;0,185;-0,016;2

-1297;0,204;-0,01;2

-1270;-0,266;0,032;2

-1239;-0,344;-0,074;2

-1200;-0,238;0,065;2

-1161;-0,338;-0;2

-1119;-0,029;-0,076;2

-1080;-0,046;0,105;2

-1032;0,706;-0,085;2

-979;1,349;0,061;2

-913;1,335;-0,018;2

-848;0,971;-0,064;2

-780;0,873;0,143;2

-713;1,003;-0,082;2

-645;0,751;-0,013;2

-591;0,655;-0,041;2

-553;0,475;-0,095;2

-550;0,233;0,107;2

-584;-0,047;-0,04;2

-649;-0,219;0,009;2

-726;-0,291;0,127;2

-803;-0,031;-0,105;2

-872;0,213;0,078;2

-929;0,402;-0,099;2

-975;0,611;-0,024;2

-1010;0,999;0,119;2

-1042;1,197;-0,032;2

-1064;0,903;0,076;2

-1090;0,31;0,004;2

-1115;0,394;-0,011;2

-1139;0,368;0,123;2

-1166;0,113;-0,076;2

-1188;-0,337;0,027;2

-1212;-0,365;0,062;2

-1229;-0,245;-0,061;2

-1244;-0,354;0,077;2

-1252;-0,284;-0,076;2

-1266;-0,321;0,021;2

-1277;-0,424;0,064;2

-1291;-0,291;-0,069;2

-1297;-0,206;0,061;2

-1299;-0,161;-0,043;2

-1296;0,373;0,026;2

-1281;0,268;0,002;2

-1255;0,624;-0,042;2

-1227;0,761;0,069;2

-1189;0,6;0,009;2

-1140;0,252;-0,037;2

-1090;0,116;0,169;2

-1032;0,174;-0,087;2

-973;0,736;0,055;2

-908;0,531;0,019;2

-839;0,319;-0,14;2

-768;-0,227;0,086;2

-696;-0,817;-0,07;2

-629;-0,904;0,055;2

-577;-1,301;0,008;2

-547;-1,12;0,031;2

-552;-0,657;0,068;2

-586;-0,763;-0,072;2

-655;-0,291;0,027;2

-729;-0,163;0,104;2

-805;-0,325;-0,049;2

-874;-0,082;0,08;2

-928;-0,234;0,021;2

-970;0,3;-0,008;2

-1009;0,608;0,083;2

-1041;0,556;-0,073;2

-1070;0,436;0,067;2

-1102;0,502;0,001;2

-1124;0,516;-0,054;2

-1144;0,16;0,094;2

-1166;0,768;-0,062;2

-1187;0,957;0,199;2

-1208;0,896;0,023;2

-1227;1,07;-0,047;2

-1242;0,445;0,107;2

-1252;0,31;-0,065;2

-1266;0,307;0,003;2

-1278;0,135;0,027;2

-1286;0,473;-0,031;2

-1298;0,587;0,081;2

-1306;0,564;-0,058;2

-1308;0,757;0,025;2

-1315;0,433;0,005;2

-1312;0,409;-0,114;2

-1308;0,417;0,041;2

-1286;-0,601;-0,048;2

-1256;-1,189;0,024;2

-1219;-0,822;0,067;2

-1179;-0,314;-0,045;2

-1129;-0,127;0,007;2

-1072;-0,15;-0,039;2

-1007;0,169;-0,061;2

-939;0,069;0,09;2

-865;-0,207;-0,062;2

-792;-0,752;0;2

-721;-0,914;0,018;2

-660;-0,364;-0,065;2

-612;-0,167;0,179;2

-585;-0,119;-0,082;2

-582;0,021;0,001;2

-609;-0,383;0,047;2

-664;-0,441;-0,065;2

-731;-0,331;0,078;2

-804;-0,372;0,018;2

-865;-0,612;-0,046;2

-920;-0,718;0,003;2

-962;-0,376;-0,111;2

-998;-0,264;0,009;2

-1032;-0,081;0,019;2

-1055;0,555;-0,002;2

-1077;0,892;0,061;2

-1098;0,952;-0,068;2

-1122;0,721;0,113;2

-1149;0,252;-0,044;2

-1178;0,152;-0,07;2

-1198;-0,332;0,073;2

-1214;-0,445;-0,04;2

-1222;-0,454;0,068;2

-1226;-0,656;0,033;2

-1229;-0,57;-0,076;2

-1243;-0,587;0,105;2

-1257;-0,569;-0,138;2

-1269;-0,625;-0,023;2

-1280;-0,479;0,059;2

-1286;-0,433;-0,124;2

-1277;-0,344;0,064;2

-1256;0,184;-0,01;2

-1223;0,37;0,058;2

-1182;0,397;0,026;2

-1141;0,966;-0,104;2

-1100;0,975;0,092;2

-1060;0,944;0,027;2

-1014;1,113;-0,051;2

-959;0,895;0,066;2

-893;0,709;-0,087;2

-823;0,585;0,046;2

-749;0,084;0,016;2

-677;0,113;-0,081;2

-613;0,097;0,087;2

-565;0,112;-0,03;2

-543;-0,378;0,049;2

-570;-0,889;0,056;2

-626;-0,788;-0,07;2

-699;-0,785;0,096;2

-771;-0,772;-0,042;2

-838;-1,005;-0,033;2

-887;-1,556;-0,006;2

-933;-1,209;-0,07;2

-975;-0,886;0,046;2

-1006;-1,198;0,007;2

-1040;-1,116;0,002;2

-1069;-1,309;0,058;2

-1097;-1,299;-0,047;2

-1117;-1,079;0,072;2

-1140;-1,306;-0,018;2

-1158;-1,193;0,031;2

-1179;-0,511;0,09;2

-1200;-0,233;-0,069;2

-1219;-0,021;0,152;2

-1239;0,067;-0,012;2

-1255;0,3;-0,009;2

-1268;0,349;0,11;2

-1277;0,329;-0,004;2

-1283;0,07;0,111;2

-1281;0,172;0,028;2

-1272;0,72;-0,077;2

-1255;0,304;0,035;2

-1235;-0,373;-0,01;2

-1205;-0,521;-0,001;2

-1166;-0,818;0,065;2

-1118;-0,687;-0,096;2

-1061;-0,617;0,045;2

-994;-0,849;-0,029;2

-923;-0,601;-0,095;2

-848;-0,687;0,047;2

-779;-0,101;-0,098;2

-708;-0,141;0,08;2

-646;-0,298;-0,021;2

-591;0,067;-0,061;2

-558;-0,226;0,079;2

-553;0,081;-0,059;2

-586;0,343;0,003;2

-647;0,215;-0,031;2

-726;0,419;0,006;2

-803;0,253;0,077;2

-873;0,273;0,005;2

-926;0,59;-0,007;2

-966;0,479;0,079;2

-1003;0,546;-0,063;2

-1038;0,533;0,053;2

-1071;0,177;-0,062;2

-1101;-0,475;-0,016;2

-1129;-0,588;0,062;2

-1150;-0,052;-0,096;2

-1165;-0,213;0,061;2

-1185;0,078;0,005;2

-1205;0,514;-0,078;2

-1226;0,377;0,113;2

-1239;0,31;-0,042;2

-1254;0,108;0,001;2

-1265;0,118;-0,015;2

-1280;-0,052;-0,083;2

-1293;-0,112;0,086;2

-1305;-0,117;-0,061;2

-1310;0,081;0,027;2

-1310;-0,037;0,034;2

-1306;0,005;-0,083;2

-1300;0,013;0,048;2

-1283;0,285;-0,045;2

-1262;0,407;-0,035;2

-1225;0,038;0,069;2

-1180;0,233;-0,064;2

-1122;0,604;0,031;2

-1059;0,799;0,018;2

-987;0,953;-0,005;2

-915;0,286;0,059;2

-839;0,125;-0,075;2

-770;0,223;0,057;2

-703;0,08;-0,037;2

-644;0,172;-0,018;2

-589;0,545;0,067;2

-554;0,695;-0,113;2

-550;0,55;0,016;2

-588;0,237;-0,039;2

-656;0,464;-0,055;2

-739;0,498;0,082;2

-819;0,453;-0,039;2

-884;0,583;-0,013;2

-937;0,506;0,033;2

-976;0,251;-0,094;2

-1009;-0,179;0,069;2

-1031;-0,581;-0,032;2

-1054;-0,314;-0,017;2

-1078;-0,123;0,048;2

-1104;-0,02;-0,072;2

-1137;-0,032;0,053;2

-1162;0,343;0,012;2

-1184;0,693;-0,04;2

-1196;0,292;0,032;2

-1208;0,263;-0,06;2

-1215;0,327;0,065;2

-1224;-0,028;-0,012;2

-1238;0,272;-0,043;2

-1254;0,185;0,092;2

-1270;-0,658;-0,082;2

-1288;-0,866;-0,009;2

-1299;-0,777;0,087;2

-1308;-0,758;-0,067;2

-1313;-0,731;0,062;2

-1307;-0,723;-0,026;2

-1293;-0,558;0,002;2

-1264;-0,881;0,027;2

-1226;-1,395;-0,093;2

-1180;-1,644;0,06;2

-1138;-1,29;-0;2

-1091;-1,193;-0,03;2

-1040;-1,172;0,071;2

-973;-1,053;-0,059;2

-904;-0,681;0,032;2

-837;-0,768;0,021;2

-775;-1,044;-0,039;2

-715;-0,804;0,082;2

-662;-0,451;-0,043;2

-611;-0,443;0,092;2

-577;-0,581;-0,021;2

-569;-0,386;-0,063;2

-599;-0,155;0,091;2

-656;0,044;-0,007;2

-729;0,237;0,001;2

-804;0,309;0,029;2

-866;0,642;-0,078;2

-924;0,758;0,106;2

-969;0,892;-0,108;2

-1008;1,092;0,016;2

-1040;0,747;0,017;2

-1060;1,112;-0,062;2

-1082;1,422;0,078;2

-1103;1,175;-0,046;2

-1129;1,163;0,018;2

-1154;0,778;0,042;2

-1181;0,247;-0,067;2

-1201;-0,29;0,056;2

-1224;-0,98;-0,014;2

-1244;-0,506;-0,022;2

-1266;-0,704;0,026;2

-1282;-0,425;-0,054;2

-1293;0,753;0,014;2

-1294;0,894;-0,004;2

-1295;0,802;-0,092;2

-1297;0,859;0,088;2

-1298;0,609;-0,105;2

-1294;0,511;-0,023;2

-1285;0,239;0,066;2

-1265;0,253;-0,045;2

-1244;0,131;0,063;2

-1216;-0,164;-0,027;2

-1177;-0,288;0,009;2

-1133;-0,432;0,063;2

-1075;0,166;-0,058;2

-1010;0,196;0,06;2

-938;-0,766;0,013;2

-865;-1,228;-0,016;2

-785;-1,691;0,066;2

-712;-1,761;-0,027;2

-644;-1,2;-0,002;2

-589;-0,624;0,045;2

-558;-0,062;-0,075;2

-566;0,006;0,036;2

-604;-0,447;-0,063;2

-673;-0,623;0,063;2

-748;-0,633;0,009;2

-816;-0,634;-0,085;2

-878;-0,686;0,082;2

-929;-0,518;-0,01;2

-976;-0,147;-0,026;2

-1015;0,107;0,037;2

-1050;0,541;-0,053;2

-1082;0,883;0,086;2

-1109;0,844;-0,009;2

-1132;0,511;-0,007;2

-1153;-0,159;0,055;2

-1172;-0,04;-0,132;2

-1186;-0,198;0,054;2

-1206;-0,322;-0,01;2

-1221;0,016;-0,024;2

-1239;0,578;0,115;2

-1249;1,527;-0,082;2

-1263;2,186;0,031;2

-1268;1,314;0,022;2

-1276;0,004;-0,063;2

-1283;-0,91;0,078;2

-1289;-0,942;-0,046;2

-1301;-0,807;-0,002;2

-1310;-1,023;0,024;2

-1310;-0,427;-0,054;2

-1292;0,3;0,05;2

-1254;1,448;-0,042;2

-1201;1,651;-0,037;2

-1149;0,86;0,078;2

-1094;-0,327;-0,097;2

-1045;-1,183;0,093;2

-992;-1,556;-0,036;2

-938;-1,352;-0,032;2

-874;-1,099;0,068;2

-808;-0,194;-0,065;2

-732;0,713;0,035;2

-655;1,044;0,006;2

-586;0,627;-0,049;2

-538;0,318;0,102;2

-529;0,06;-0,087;2

-565;-0,191;0,022;2

-638;-0,408;-0,011;2

-716;-0,085;0,025;2

-793;0,731;0,101;2

-858;1,325;-0,034;2

-915;1,781;0,051;2

-963;1,967;0,078;2

-1000;1,524;-0,078;2

-1035;0,508;0,166;2

-1059;-0,179;-0,01;2

-1084;-0,27;-0,018;2

-1103;-0,839;0,04;2

-1115;-0,277;-0,06;2

-1127;-0,149;0,082;2

-1146;-0,605;-0,021;2

-1167;-0,825;-0,001;2

-1197;-1,073;0,075;2

-1229;-0,827;-0,081;2

-1252;-0,441;0,112;2

-1271;-0,461;0,007;2

-1276;-0,002;-0,02;2

-1278;0,028;0,057;2

-1277;0,037;-0,032;2

-1276;-0,138;0,011;2

-1279;-0,606;0,068;2

-1282;-0,136;-0,02;2

-1270;0,124;0,076;2

-1251;0,037;-0,052;2

-1219;0,561;-0,012;2

-1183;0,469;0,026;2

-1131;0,135;-0,032;2

-1080;-0,391;0,073;2

-1022;-0,557;-0,005;2

-965;-0,516;0,057;2

-903;-0,259;0,07;2

-840;0,286;-0,042;2

-763;0,697;0,136;2

-684;0,403;0,011;2

-610;0,26;-0,02;2

-553;-0,137;0,087;2

-531;-0,718;-0,06;2

-547;-0,536;0,017;2

-607;0,004;0,031;2

-684;0,464;-0,047;2

-767;0,847;0,076;2

-840;0,97;-0,078;2

-895;1,035;0,026;2

-940;0,481;0,014;2

-975;0,404;-0,023;2

-1011;-0,035;0,054;2

-1042;-0,638;-0,008;2

-1075;-0,602;0,02;2

-1103;-0,085;0,061;2

-1130;0,877;-0,032;2

-1152;0,606;0,081;2

-1169;0,379;-0,018;2

-1186;0,44;-0,077;2

-1205;0,034;0,058;2

-1224;-0,417;-0,167;2

-1239;-0,6;0,083;2

-1256;-0,33;0,059;2

-1264;-0,081;-0,032;2

-1269;-0,026;0,063;2

-1269;0,128;-0,074;2

-1271;0,125;0,064;2

-1271;-0,357;0,012;2

-1271;-0,459;-0,038;2

-1258;-0,729;0,104;2

-1236;-1,083;-0,051;2

-1200;-0,701;-0,044;2

-1161;-0,456;0,066;2

-1112;-0,44;-0,051;2

-1059;-0,226;0,148;2

-993;0,097;0,057;2

-926;0,434;-0,012;2

-859;0,263;0,085;2

-799;-0;-0,097;2

-739;-0,069;0,116;2

-682;-0,084;-0,042;2

-631;0,145;0,02;2

-585;0,136;0,064;2

-571;0,425;-0,08;2

-591;0,737;0,057;2

-650;0,689;0,001;2

-724;0,904;-0,063;2

-800;1,013;0,079;2

-872;0,97;-0,107;2

-928;1,029;0,016;2

-979;0,942;0,03;2

-1016;1,008;-0,059;2

-1046;0,784;0,074;2

-1070;0,274;-0,08;2

-1094;-0,074;0,019;2

-1118;-0,452;0,051;2

-1145;-0,569;-0,188;2

-1169;-0,649;0,085;2

-1188;-0,246;-0,048;2

-1208;0,477;-0,005;2

-1222;0,403;0,09;2

-1236;0,304;-0,145;2

-1245;0,412;0,038;2

-1255;0,512;0,058;2

-1261;0,826;-0,022;2

-1274;0,418;0,095;2

-1289;0,179;-0,065;2

-1305;0,147;0,057;2

-1315;0,081;0,024;2

-1322;0,094;-0,022;2

-1320;-0,312;0,062;2

-1317;-0,903;-0,091;2

-1302;-1,113;-0,002;2

-1280;-1,204;0,065;2

-1247;-0,956;-0,116;2

-1208;-1,113;0,098;2

-1166;-0,948;-0,013;2

-1122;0,02;0,015;2

-1075;0,021;-0;2

-1021;0,188;-0,08;2

-957;0,279;0,11;2

-886;-0,035;-0,064;2

-815;-0,112;0,022;2

-744;-0,461;0,098;2

-677;-0,445;-0,024;2

-614;-0,394;0,025;2

-570;-0,442;-0,035;2

-553;-0,191;-0,076;2

-576;-0,432;0,057;2

-632;-0,638;-0,08;2

-709;-0,232;0,038;2

-789;-0,408;0,001;2

-860;-0,355;-0,103;2

-920;-0,45;0,079;2

-965;-0,287;-0,065;2

-1007;0,262;0,027;2

-1035;0,178;-0,006;2

-1061;0,294;-0,074;2

-1082;0,231;0,075;2

-1102;0,228;-0,022;2

-1122;0,313;-0,018;2

-1145;0,146;0,013;2

-1164;0,065;-0,082;2

-1190;0,134;0,049;2

-1214;0,06;0,024;2

-1236;0,05;-0,075;2

-1252;0,144;0,098;2

-1263;0,299;-0,049;2

-1269;0,492;0,134;2

-1279;-0,105;0,075;2

-1286;-0,079;-0,041;2

-1290;-0,06;0,109;2

-1290;0,138;-0,082;2

-1287;0,018;0,014;2

-1277;-0,361;0,033;2

-1262;-0,37;-0,129;2

-1238;-0,441;0,112;2

-1207;-0,658;-0,046;2

-1168;-0,411;0,012;2

-1120;-0,762;0,032;2

-1063;-1,037;-0,075;2

-1004;-1,122;0,069;2

-941;-0,946;-0,008;2

-871;-0,385;-0,028;2

-802;-0,043;0,109;2

-732;0,258;-0,065;2

-673;0,282;0,1;2

-620;0,232;0,085;2

-581;0,343;-0,02;2

-565;0,261;0,049;2

-581;-0,011;-0,039;2

-637;-0,037;0,027;2

-709;-0,026;0,006;2

-788;0,099;0,001;2

-856;0,102;0,079;2

-916;0,099;-0,017;2

-966;0,258;0,015;2

-1011;0,07;0,033;2

-1045;-0,19;-0,084;2

-1069;-0,627;0,054;2

-1089;-0,734;-0,028;2

-1106;-0,576;-0,018;2

-1131;-0,519;0,04;2

-1157;-0,504;-0,137;2

-1185;-0,267;0,042;2

-1205;0,234;-0,06;2

-1222;0,098;0,004;2

-1231;0,256;0,065;2

-1243;0,293;-0,047;2

-1252;0,017;0,034;2

-1266;0,607;0,011;2

-1276;1,186;-0,036;2

-1290;1,036;0,016;2

-1299;1,227;-0,074;2

-1308;0,848;0,021;2

-1307;0,721;0,021;2

-1296;0,728;-0,096;2

-1270;0,365;0,035;2

-1237;0,533;-0,105;2

-1199;0,768;0,011;2

-1164;0,012;0,025;2

-1119;-0,237;-0,089;2

-1068;0,061;0,086;2

-1012;0,523;-0,008;2

-953;0,596;-0,011;2

-897;0,302;0,141;2

-839;-0,342;-0,076;2

-782;-0,691;0,066;2

-723;-0,895;0,008;2

-673;-0,792;-0,01;2

-633;-0,895;0,057;2

-620;-0,391;0,039;2

-642;-0,214;0,05;2

-688;-0,006;0,039;2

-752;0,198;-0,056;2

-817;0,387;0,045;2

-881;0,693;-0,079;2

-937;1,114;0,009;2

-990;0,786;0,023;2

-1032;0,322;-0,02;2

-1068;0,564;0,047;2

-1096;0,714;-0,037;2

-1122;0,374;0,006;2

-1140;-0,038;0,044;2

-1155;-0,061;-0,148;2

-1174;-0,032;0,075;2

-1197;0,181;-0,008;2

-1221;0,342;-0,035;2

-1242;0,082;0,067;2

-1264;0,228;-0,101;2

-1278;0,715;0,081;2

-1291;0,568;-0,029;2

-1296;0,684;-0,029;2

-1305;0,695;0,075;2

-1303;0,576;-0,075;2

-1308;0,405;0,031;2

-1312;0,517;-0,006;2

-1321;0,337;-0,103;2

-1319;0,1;0,019;2

-1302;0,129;-0,103;2

-1267;-0,181;0,005;2

-1225;-0,318;0,004;2

-1172;0,179;-0,076;2

-1119;-0,167;0,062;2

-1066;-0,689;-0,04;2

-1002;-0,614;-0,02;2

-939;-0,453;0,064;2

-873;-0,278;-0,094;2

-807;-0,531;0,101;2

-741;-0,615;-0,03;2

-681;-0,694;-0,025;2

-625;-0,994;0,09;2

-585;-0,62;-0,031;2

-576;-0,748;0,034;2

-604;-1,201;-0;2

-666;-1,009;-0;2

-740;-0,722;0,09;2

-814;-0,767;-0,03;2

-877;-0,928;0,035;2

-936;-0,826;-0;2

-985;-0,156;-0,054;2

-1033;-0,224;0,085;2

-1073;-0,538;-0,044;2

-1107;-0,246;-0,017;2

-1132;-0,089;0,047;2

-1155;-0,199;-0,101;2

-1172;-0,367;0,079;2

-1190;-0,199;-0,019;2

-1204;0,036;0,044;2

-1217;-0,168;0,093;2

-1235;-0,302;-0,034;2

-1253;-0,681;0,081;2

-1275;-0,403;0,022;2

-1294;-0,264;-0,047;2

-1307;-0,179;0,088;2

-1316;-0,298;-0,087;2

-1323;-0,078;0,006;2

-1327;0,054;0,016;2

-1329;0,092;-0,035;2

-1323;-0,235;0,064;2

-1311;-0,143;-0,078;2

-1282;-0,07;0,07;2

-1249;-0,24;0,01;2

-1209;-0,252;-0,062;2

-1168;-0,23;0,066;2

-1121;0,092;-0,045;2

-1069;0,499;0,053;2

-1014;0,743;0,06;2

-960;0,675;-0,055;2

-900;0,667;0,072;2

-834;0,556;-0,02;2

-758;0,523;-0,011;2

-681;0,523;0,047;2

-610;0,24;-0,094;2

-550;0,379;0,073;2

-519;0,668;-0,007;2

-526;0,688;0,004;2

-577;0,387;0,099;2

-655;0,447;-0,075;2

-742;0,532;0,056;2

-822;0,416;0,001;2

-890;0,201;-0,105;2

-942;0,208;0,102;2

-985;0,202;-0,041;2

-1019;0,036;0,03;2

-1047;-0,143;0,039;2

-1071;0,106;-0,063;2

-1095;0,005;0,049;2

-1120;-0,12;-0,072;2

-1139;-0,466;-0,006;2

-1160;-0,991;0,089;2

-1179;-0,867;-0,082;2

-1199;-0,929;0,171;2

-1218;-1,05;-0,035;2

-1241;-0,74;-0,003;2

-1257;-0,612;0,086;2

-1270;-0,397;-0,077;2

-1275;-0,336;0,035;2

-1283;-0,138;-0,034;2

-1286;0,332;0,009;2

-1295;0,612;0,129;2

-1301;0,833;-0,094;2

-1304;0,811;0,009;2

-1293;0,57;-0,024;2

-1276;0,574;-0,105;2

-1242;0,268;0,091;2

-1203;0,274;-0,033;2

-1155;0,342;0,012;2

-1103;0,184;0,062;2

-1045;0,21;-0,066;2

-984;0,155;0,08;2

-924;-0,027;-0,049;2

-859;-0,149;0,014;2

-792;-0,356;0,039;2

-722;0,112;-0,048;2

-657;0,404;0,1;2

-595;0,509;-0,02;2

-546;0,486;-0,016;2

-525;0,208;0,068;2

-542;0,14;-0,067;2

-597;0,094;0,038;2

-672;0,145;-0,028;2

-758;-0,466;0,044;2

-835;-1,16;0,087;2

-902;-1,13;-0,058;2

-956;-0,53;0;2

-1002;0,178;0,037;2

-1038;0,419;-0,072;2

-1071;0,516;0,046;2

-1095;0,699;-0,054;2

-1115;0,959;0;2

-1134;0,67;0,136;2

-1154;0,364;-0,094;2

-1181;-0,052;0,076;2

-1204;-0,486;-0,089;2

-1226;-0,534;0,01;2

-1244;-0,425;0,07;2

-1259;-0,119;-0,066;2

-1271;-0,245;0,087;2

-1288;-0,306;0,012;2

-1302;0,284;-0,059;2

-1319;0,648;0,081;2

-1329;0,507;0,056;2

-1337;0,464;0,052;2

-1332;0,201;-0,01;2

-1315;-0,054;0,01;2

-1279;-0,198;0,082;2

-1238;-0,249;-0,065;2

-1190;-0,081;0,017;2

-1148;-0,028;0,018;2

-1103;-0,003;-0,08;2

-1052;0,203;0,073;2

-997;0,545;-0,058;2

-934;0,726;0,011;2

-872;0,337;0,052;2

-805;0,071;-0,099;2

-743;0,193;0,093;2

-682;0,264;-0,043;2

-624;0,369;-0,02;2

-591;0,005;0,087;2

-590;0,081;-0,038;2

-630;0,118;0,058;2

-692;-0,099;0,007;2

-767;-0,096;-0,076;2

-840;-0,383;0,076;2

-910;-0,318;-0,067;2

-969;0,066;0,002;2

-1021;0,023;0,02;2

-1063;-0,11;-0,075;2

-1098;-0,163;0,089;2

-1126;0,193;-0,073;2

-1147;0,501;-0;2

-1163;-0,161;0,012;2

-1177;-0,318;-0,109;2

-1192;-0,521;0,081;2

-1204;-0,747;-0,097;2

-1223;-0,628;0,028;2

-1239;-0,844;0,057;2

-1258;0,04;-0,101;2

-1273;0,217;0,025;2

-1293;0,278;-0,037;2

-1311;0,953;-0,017;2

-1328;0,31;0,089;2

-1339;0,184;-0,085;2

-1339;0,116;0,052;2

-1325;-0,208;0,02;2

-1302;-0,168;-0,06;2

-1274;-0,001;0,113;2

-1239;0,286;-0,049;2

-1197;0,175;0,026;2

-1148;0,278;0,033;2

-1097;0,385;-0,063;2

-1040;0,179;0,064;2

-983;-0,215;-0,042;2

-922;-0,138;0,022;2

-857;-0,056;0,035;2

-788;-0,147;-0,101;2

-722;-0,224;0,08;2

-664;-0,606;0,029;2

-610;-0,66;-0,044;2

-584;-0,58;0,067;2

-586;-0,221;-0,11;2

-627;0,267;0,057;2

-691;0,547;-0,004;2

-769;0,671;0,135;2

-843;0,781;0,148;2

-908;0,91;-0,049;2

-957;0,531;0,074;2

-1001;0,492;0,03;2

-1036;0,768;-0,052;2

-1071;-0,024;0,086;2

-1100;-0,241;-0,088;2

-1130;0,056;-0,045;2

-1154;0,004;-0,008;2

-1177;0,229;-0,012;2

-1200;0,446;0,094;2

-1221;0,562;-0,092;2

-1232;0,686;0,007;2

-1244;0,106;0,081;2

-1254;-0,41;-0,042;2

-1264;-0,386;0,091;2

-1276;-0,146;0,038;2

-1289;0,314;0,004;2

-1303;0,072;0,067;2

-1315;-0,125;-0,041;2

-1323;0,012;0,074;2

-1329;0,063;-0,02;2

-1331;0,08;-0,037;2

-1322;0,007;0,109;2

-1299;0,144;-0,093;2

-1263;0,02;0,017;2

-1224;-0,296;0,047;2

-1175;-0,104;-0,09;2

-1123;-0,15;0,1;2

-1067;-0,361;-0,061;2

-1004;-0,316;-0,007;2

-946;-0,336;0,047;2

-884;-0,011;-0,089;2

-826;0,099;0,056;2

-763;-0,045;-0,004;2

-703;-0,33;0,041;2

-655;-0,465;0,103;2

-624;-0,4;-0,072;2

-621;-0,361;-0,016;2

-646;-0,454;0;2

-697;-0,196;-0,034;2

-760;0,186;0,084;2

-827;0,742;-0,116;2

-886;1,014;0,029;2

-941;1,275;-0,008;2

-983;1,715;-0,038;2

-1025;1,54;0,102;2

-1065;1,057;-0,08;2

-1103;1,202;0,013;2

-1131;0,956;-0,008;2

-1157;0,809;-0,023;2

-1174;0,813;0,057;2

-1188;0,695;-0,065;2

-1193;0,643;0,037;2

-1202;0,318;0,024;2

-1211;0,021;-0,123;2

-1219;-0,41;0,051;2

-1232;-0,702;-0,039;2

-1249;-0,842;-0,027;2

-1272;-1,064;0,064;2

-1291;-0,687;-0,054;2

-1307;-0,754;0,065;2

-1316;-1,052;-0,003;2

-1317;-0,81;-0,025;2

-1309;-0,84;0,096;2

-1290;-0,649;-0,085;2

-1254;-0,749;0,004;2

-1212;-1,158;0,04;2

-1158;-0,853;-0,083;2

-1107;-0,682;0,117;2

-1057;-0,772;-0,072;2

-999;-0,857;0,037;2

-944;-1,133;0,039;2

-879;-0,641;-0,085;2

-816;-0,194;0,076;2

-754;0,131;-0,068;2

-693;0,181;-0,008;2

-636;0,43;0,081;2

-582;0,669;-0,108;2

-558;0,389;0,051;2

-574;0,249;-0,014;2

-631;-0,025;0,041;2

-705;-0,404;0,044;2

-786;-0,167;-0,001;2

-848;0,468;0,056;2

-909;0,752;0,003;2

-956;1,12;-0,034;2

-998;0,913;0,097;2

-1035;0,962;-0,02;2

-1068;1,444;0,046;2

-1096;1,17;-0,046;2

-1122;0,675;-0,058;2

-1147;0,686;0,097;2

-1169;0,742;-0,052;2

-1187;0,04;0,02;2

-1207;-0,054;0,044;2

-1224;0,29;-0,098;2

-1239;0,202;0,054;2

-1257;0,361;-0,042;2

-1269;0,596;-0,025;2

-1280;0,469;-0,004;2

-1288;0,798;-0,131;2

-1296;0,948;0,045;2

-1301;1,068;0,014;2

-1308;1,093;0,009;2

-1305;0,871;0,043;2

-1297;0,973;-0,045;2

-1274;1,091;-0,021;2

-1249;1,58;0,006;2

-1214;1,93;-0,067;2

-1169;0,889;0,071;2

-1125;-0,006;-0,06;2

-1072;-0,528;-0,001;2

-1017;-0,558;0,047;2

-955;-0,243;-0,083;2

-893;-0,485;0,135;2

-826;-0,866;0,004;2

-758;-1,127;-0,041;2

-700;-0,877;0,063;2

-649;-0,481;-0,117;2

-614;-0,339;0,043;2

-602;-0,444;0,005;2

-630;0,522;0,027;2

-686;1,684;0,06;2

-761;2,811;-0,115;2

-829;2,81;0,069;2

-892;1,652;-0,027;2

-935;0,132;-0,044;2

-974;-0,943;0,077;2

-1012;-0,842;-0,094;2

-1056;-0,002;0,029;2

-1097;1,019;0;2

-1131;1,502;-0,024;2

-1158;0,869;0,077;2

-1183;-0,045;-0,053;2

-1203;-0,871;0,007;2

-1214;-1,589;0,057;2

-1223;-1,34;-0,102;2

-1224;-0,778;0,03;2

-1229;-0,549;0;2

-1239;-0,701;-0,008;2

-1255;-0,368;0,078;2

-1270;0,007;-0,089;2

-1285;0,051;0,099;2

-1293;0,339;0,014;2

-1301;0,418;0,017;2

-1305;-0,049;0,072;2

-1312;-0,13;-0,09;2

-1314;-0,058;0,068;2

-1305;-0,33;-0,023;2

-1280;-0,216;-0,073;2

-1242;-0,032;0,076;2

-1201;1,242;-0,024;2

-1146;1,432;0,014;2

-1092;0,457;0,033;2

-1038;-0,074;-0,064;2

-979;-0,498;0,073;2

-920;-0,4;-0,022;2

-856;0,098;-0,023;2

-793;0,532;0,065;2

-725;0,701;-0,047;2

-660;0,561;0,082;2

-594;0,265;0,028;2

-549;0,078;-0,022;2

-531;-0,179;0,055;2

-554;0,032;-0,028;2

-615;-0,027;0,086;2

-695;0,391;0,004;2

-776;0,967;0,03;2

-850;0,983;0,082;2

-913;0,543;-0,007;2

-958;0,161;0,046;2

-995;-0,261;0,072;2

-1027;-0,265;-0,046;2

-1059;-0,7;0,06;2

-1088;-0,637;-0,081;2

-1116;-0,128;0,052;2

-1135;0,188;0,069;2

-1157;0,56;-0,045;2

-1172;0,541;0,062;2

-1187;0,251;-0,06;2

-1202;0,031;-0,033;2

-1214;-0,574;0,027;2

-1226;-0,051;-0,123;2

-1237;1,002;0,084;2

-1253;0,983;-0,038;2

-1262;0,3;0,023;2

-1269;-0,568;0,097;2

-1272;-1,196;-0,09;2

-1280;-1,46;0,057;2

-1286;-1,667;0,012;2

-1292;-1,761;0,006;2

-1284;-1,549;0,032;2

-1261;-0,795;-0,086;2

-1228;0,252;0,031;2

-1188;0,726;0,017;2

-1148;0,715;0,022;2

-1100;0,277;0,07;2

-1045;-0,383;-0,023;2

-988;-0,722;0,064;2

-928;-1,092;-0,062;2

-874;-1,06;-0,094;2

-821;-0,529;0,071;2

-763;0,107;-0,044;2

-702;0,943;-0,023;2

-648;0,878;0,058;2

-602;0,408;-0,075;2

-585;-0,276;0,028;2

-599;-0,394;-0,045;2

-643;-0,693;0,005;2

-705;-1,069;0,077;2

-777;-0,923;-0,11;2

-849;-0,913;0,003;2

-912;-0,486;-0,024;2

-966;0,817;-0,002;2

-1008;1,025;0,065;2

-1042;0,458;-0,047;2

-1072;-0,3;-0,073;2

-1102;-1,017;-0,008;2

-1124;-1,347;-0,034;2

-1143;-1,627;0,11;2

-1156;-1,261;-0,075;2

-1169;-0,871;0,01;2

-1182;-0,334;0,019;2

-1200;0,562;-0,061;2

-1216;0,768;0,082;2

-1231;0,881;0,012;2

-1242;0,025;0,02;2

-1258;-0,679;0,073;2

-1271;-0,559;-0,184;2

-1283;-0,497;0,097;2

-1290;-0,649;-0,044;2

-1291;-0,209;-0,018;2

-1295;0,292;0,039;2

-1296;1,36;-0,063;2

-1292;1,915;0,06;2

-1275;0,823;0,043;2

-1246;-0,848;-0,058;2

-1215;-1,992;0,08;2

-1176;-1,529;-0,038;2

-1137;-0,901;0,046;2

-1091;-0,895;-0,005;2

-1038;-0,631;-0,112;2

-979;-0,611;0,078;2

-913;-0,51;-0,058;2

-844;-0,222;-0,017;2

-777;0,09;0,054;2

-712;0,462;-0,052;2

-653;0,443;0,07;2

-609;0,423;0,016;2

-588;0,623;-0,025;2

-605;0,518;0,104;2

-653;0,671;-0,064;2

-723;0,84;0,074;2

-794;1,074;-0,012;2

-862;1,687;-0,024;2

-916;0,825;0,059;2

-965;0,066;-0,02;2

-1001;-0,027;0,082;2

-1035;-0,528;0,076;2

-1064;-0,732;-0,053;2

-1093;-0,896;0,117;2

-1121;-0,606;-0,06;2

-1146;-0,286;0,025;2

-1174;-0,727;0,083;2

-1196;-1,03;-0,066;2

-1218;-1,098;0,056;2

-1231;-0,893;-0,054;2

-1242;-0,42;0,049;2

-1251;-0,098;0,043;2

-1264;0,622;-0,023;2

-1271;1,035;0,099;2

-1286;0,827;-0,007;2

-1299;0,14;-0,006;2

-1312;-1,097;0,092;2

-1319;-0,966;-0,1;2

-1321;-0,954;-0,015;2

-1325;-0,892;-0,017;2

-1320;-0,136;-0,027;2

-1305;0,172;0,092;2

-1276;1,019;-0,074;2

-1237;1,387;0,041;2

-1196;1,124;-0,028;2

-1148;0,362;-0,03;2

-1103;-0,842;0,065;2

-1049;-0,989;-0,081;2

-989;-0,958;0,005;2

-919;-1,025;0,071;2

-850;-0,83;-0,086;2

-782;-0,797;0,092;2

-720;-0,436;-0,011;2

-657;0,208;0,004;2

-611;0,982;0,051;2

-586;2,385;-0,067;2

-599;1,553;0,03;2

-648;-0,171;-0,041;2

-719;-0,266;-0,019;2

-800;-0,185;0,083;2

-870;-0,769;-0,07;2

-930;-1,126;-0,045;2

-975;-1,007;-0,008;2

-1015;-0,361;-0,062;2

-1047;-0,163;0,116;2

-1076;0,175;-0,125;2

-1098;0,354;0,065;2

-1125;-0,3;0,006;2

-1149;-0,608;-0,012;2

-1176;-0,766;0,006;2

-1197;-0,713;-0,082;2

-1215;-0,569;-0,038;2

-1230;-0,42;0,037;2

-1244;0,323;-0,101;2

-1259;0,313;0,051;2

-1275;0,002;-0,024;2

-1288;-0,418;-0,05;2

-1297;-0,652;0,046;2

-1306;-0,33;-0,067;2

-1313;-0,344;0,207;2

-1318;-0,453;-0,002;2

-1317;-0,125;-0,038;2

-1305;-0,11;0,073;2

-1283;0,092;-0,074;2

-1251;0,186;0,009;2

-1218;-0,47;0,023;2

-1174;-0,898;-0,022;2

-1122;-0,769;0,097;2

-1061;-0,403;-0,042;2

-995;-0,414;0,001;2

-932;-0,79;0,058;2

-869;-0,866;-0,112;2

-802;-0,993;0,037;2

-737;-0,79;-0,054;2

-674;-0,414;0,033;2

-623;-0,144;0,043;2

-598;0,081;-0,072;2

-611;0,255;0,032;2

-661;-0,032;-0,009;2

-731;-0,345;0,041;2

-809;-0,581;0,06;2

-879;-0,429;-0,073;2

-939;-0,267;0,04;2

-988;-0,363;-0,007;2

-1030;0,436;-0,032;2

-1062;0,441;0,083;2

-1092;0,265;-0,008;2

-1114;0,19;0,031;2

-1136;-0,036;-0,029;2

-1154;-0,068;-0,07;2

-1172;-0,168;0,054;2

-1189;0,211;-0,066;2

-1206;0,622;0,021;2

-1221;0,437;-0,006;2

-1238;0,567;-0,085;2

-1249;0,327;0,093;2

-1266;-0,169;-0,028;2

-1283;-0,056;-0,018;2

-1295;-0,358;0,072;2

-1304;-0,84;-0,111;2

-1304;-1,165;0,033;2

-1298;-1,545;-0,003;2

-1277;-1,245;-0,01;2

-1250;-0,879;0,063;2

-1218;-0,669;-0,108;2

-1176;-0,283;0,027;2

-1135;0,086;0,016;2

-1092;0,36;-0,03;2

-1051;-0,145;0,005;2

-1004;-0,504;-0,027;2

-945;-0,532;0,061;2

-876;-0,722;0,076;2

-804;-0,894;-0,103;2

-728;-0,553;0,088;2

-664;-0,766;-0,042;2

-605;-0,814;0,025;2

-563;-0,23;0,048;2

-553;0,181;-0,089;2

-588;0,21;0,049;2

-652;0,388;-0,029;2

-732;0,036;-0,014;2

-808;0,162;0,037;2

-874;0,262;-0,054;2

-928;0,299;0,067;2

-976;-0,059;0,029;2

-1013;-0,185;-0,049;2

-1043;-0,357;0,077;2

-1064;-0,457;-0,058;2

-1078;-0,602;0,034;2

-1091;-0,934;-0,005;2

-1109;-0,656;-0,045;2

-1131;-0,311;0,12;2

-1155;-0,275;-0,098;2

-1177;-0,458;0,029;2

-1195;-0,57;0,03;2

-1216;-0,197;-0,089;2

-1234;0,012;0,068;2

-1251;-0,514;-0,112;2

-1259;-0,645;0,045;2

-1267;-0,834;0,013;2

-1272;-1,143;-0,073;2

-1277;-1,004;0,081;2

-1288;-1,14;-0,032;2

-1292;-0,487;0,01;2

-1289;-0,199;0,005;2

-1269;0,123;-0,065;2

-1246;0,094;0,041;2

-1212;0,237;-0,037;2

-1173;0,276;-0,045;2

-1124;0,045;0,102;2

-1067;0,235;-0,063;2

-1006;0,357;-0,009;2

-942;0,089;0,009;2

-877;0,355;-0,036;2

-812;0,261;0,077;2

-743;0,313;-0,011;2

-677;0,339;0,016;2

-619;0,242;0,034;2

-575;0,573;-0,093;2

-566;0,722;0,137;2

-585;0,46;-0,054;2

-637;1,008;-0,005;2

-701;0,877;0,049;2

-775;1,16;-0,055;2

-839;1,541;0,069;2

-896;1,136;-0,046;2

-942;0,956;0,021;2

-986;1,12;0,067;2

-1027;1,189;0,014;2

-1070;0,915;0,04;2

-1099;0,475;-0,02;2

-1126;0,62;-0,114;2

-1143;0,526;0,084;2

-1161;0,849;-0,088;2

-1174;1,192;0,024;2

-1187;1,202;0,016;2

-1199;1,29;-0,066;2

-1213;1,204;0,068;2

-1227;0,849;-0,032;2

-1242;0,444;-0,038;2

-1259;0,22;0,015;2

-1268;0,495;-0,091;2

-1280;0,519;0,041;2

-1289;0,404;-0,003;2

-1299;0,17;-0,012;2

-1310;0,153;0,065;2

-1310;0,427;-0,074;2

-1299;-0,011;0,057;2

-1270;-0,448;-0,023;2

-1234;0,022;0,008;2

-1191;0,243;0,089;2

-1149;-0,134;-0,032;2

-1097;-0,166;0,126;2

-1047;-0,063;0,001;2

-991;-0,065;-0,069;2

-933;0,009;0,091;2

-870;-0,199;-0,104;2

-807;-0,931;0,025;2

-742;-0,8;-0,016;2

-674;-0,493;-0,085;2

-619;-0,545;0,048;2

-587;0,008;-0,055;2

-588;0,673;0,005;2

-625;0,892;0,042;2

-689;0,869;-0,083;2

-761;0,33;0,113;2

-835;-0,065;-0,015;2

-898;-0,788;0,04;2

-958;-0,694;0,045;2

-1003;-0,667;-0,099;2

-1041;-0,501;0,038;2

-1068;-0,404;-0,012;2

-1093;-0,383;-0,051;2

-1112;-0,267;0,098;2

-1134;-0,603;-0,049;2

-1148;-0,451;0,023;2

-1165;-0,433;0,028;2

-1180;-0,934;-0,068;2

-1198;-0,011;0,065;2

-1215;0,243;-0,067;2

-1228;0,095;0,004;2

-1245;0,074;-0,038;2

-1258;0,715;-0,053;2

-1276;0,999;0,003;2

-1289;0,896;-0,109;2

-1294;0,407;0,002;2

-1290;-0,064;0,02;2

-1265;-0,389;-0,095;2

-1232;-0,516;0,082;2

-1187;-0,157;0,003;2

-1140;0,056;-0,009;2

-1087;0,035;0,033;2

-1032;0,044;-0,079;2

-972;0,33;0,11;2

-911;0,598;0,073;2

-846;0,639;-0,054;2

-778;0,509;0,059;2

-709;0,666;-0,065;2

-642;0,659;0,064;2

-594;0,554;-0,025;2

-563;0,493;-0,086;2

-574;0,329;0,048;2

-616;0,281;-0,038;2

-682;0,299;0,006;2

-758;0,491;-0,008;2

-833;0,481;-0,121;2

-893;0,308;0,081;2

-942;0,64;-0,026;2

-982;0,399;-0,013;2

-1015;-0,222;0,056;2

-1046;-0,213;-0,103;2

-1078;-0,223;0,064;2

-1107;-0,284;0,098;2

-1133;-0,182;-0,011;2

-1162;-0,284;0,105;2

-1184;-0,068;-0,084;2

-1207;-0,169;0,067;2

-1223;-0,186;-0;2

-1234;0,338;-0,128;2

-1239;0,061;0,064;2

-1248;0,476;-0,084;2

-1256;0,266;0,019;2

-1264;-0,099;-0,011;2

-1273;-0,039;-0,045;2

-1276;0,473;0,069;2

-1287;0,233;0,022;2

-1291;0,438;-0,004;2

-1293;0,299;0,02;2

-1281;0,515;-0,042;2

-1251;0,625;0,06;2

-1212;0,603;0,02;2

-1161;0,564;0,059;2

-1109;-0,127;0,075;2

-1049;0,05;-0,086;2

-985;0,492;0,096;2

-915;0,089;-0,006;2

-846;0,191;0,02;2

-780;0,24;0,063;2

-722;0,612;-0,054;2

-667;0,821;0,016;2

-620;0,735;0,008;2

-598;1,116;-0,031;2

-608;0,796;0,031;2

-654;0,624;-0,101;2

-719;0,81;-0,006;2

-795;0,947;0,067;2

-859;0,432;-0,023;2

-914;0,029;0,073;2

-957;0,136;-0,038;2

-998;0,293;0,015;2

-1028;0,298;0,078;2

-1058;0,709;-0,098;2

-1081;0,394;0,065;2

-1102;0,121;-0,023;2

-1123;0,029;0,011;2

-1149;0,15;0,118;2

-1167;0,411;-0,059;2

-1184;0,362;0,046;2

-1202;0,596;-0,093;2

-1218;0,823;-0,043;2

-1234;0,334;0,086;2

-1249;0,286;-0,057;2

-1259;0,202;0,013;2

-1264;0,357;0,037;2

-1267;0,659;-0,038;2

-1268;0,113;0,08;2

-1273;0,126;-0,069;2

-1282;0,392;0;2

-1286;-0,026;0,022;2

-1290;-0,126;0,013;2

-1277;0,094;0,077;2

-1254;-0,327;0,007;2

-1216;-0,707;-0,001;2

-1169;-0,735;0,084;2

-1111;-0,682;-0,022;2

-1047;-0,365;0,073;2

-977;-0,019;-0,037;2

-904;-0,204;-0,048;2

-835;-0,311;0,051;2

-766;-0,107;-0,112;2

-702;-0,504;0,052;2

-643;-1,052;0,009;2

-604;-0,939;-0,046;2

-582;-0,795;0,06;2

-598;-0,386;-0,053;2

-644;0,122;0,014;2

-713;0,157;0,003;2

-782;0,007;-0,039;2

-845;-0,061;0,05;2

-899;0,08;-0,05;2

-943;0,432;-0,007;2

-981;-0,06;0,07;2

-1022;-0,049;-0,063;2

-1060;-0,125;0,173;2

-1090;-0,499;-0,041;2

-1117;-0,152;0,03;2

-1138;0,045;0,089;2

-1159;-0,257;-0,067;2

-1175;-0,646;0,077;2

-1194;-0,74;-0,027;2

-1204;-0,773;-0,004;2

-1212;-0,829;0,087;2

-1223;-0,672;-0,061;2

-1235;-0,419;0,056;2

-1251;-0,309;0,017;2

-1265;-0;-0,082;2

-1278;-0,3;0,084;2

-1291;0,237;-0,053;2

-1300;0,541;0,017;2

-1303;0,235;-0,014;2

-1295;0,433;-0,061;2

-1268;0,419;0,093;2

-1224;0,53;-0,053;2

-1179;0,743;-0,041;2

-1131;0,441;0,039;2

-1089;0,356;-0,061;2

-1037;0,553;0,017;2

-983;0,366;-0,014;2

-918;0,353;-0,048;2

-851;0,127;0,07;2

-784;0,301;-0,04;2

-718;0,374;0,122;2

-653;0,276;-0,029;2

-602;0,346;-0,04;2

-575;0,483;0,085;2

-588;0,655;-0,049;2

-641;0,645;0,056;2

-717;0,675;0;2

-798;0,919;-0,069;2

-867;0,414;0,133;2

-926;0,187;-0,064;2

-974;0,05;0,015;2

-1016;-0,037;0,034;2

-1046;0,317;-0,052;2

-1071;0,314;0,084;2

-1090;0,446;-0,027;2

-1111;0,702;0,021;2

-1130;0,647;0,052;2

-1157;0,523;-0,108;2

-1177;0,201;0,088;2

-1197;0,05;-0,012;2

-1209;0,26;-0,046;2

-1223;0,097;0,041;2

-1235;0,31;-0,074;2

-1249;0,457;0,069;2

-1264;0,192;0,018;2

-1271;-0,156;-0,028;2

-1279;-0,761;0,121;2

-1282;-0,753;-0,099;2

-1284;-0,882;0,051;2

-1280;-1,209;0,04;2

-1265;-1,006;-0,08;2

-1246;-0,645;0,113;2

-1214;-0,421;-0,056;2

-1178;-0,325;0,032;2

-1133;-0,214;0,125;2

-1086;0,379;-0,107;2

-1024;0,011;0,118;2

-958;-0,686;-0,003;2

-883;-0,949;-0,003;2

-809;-0,88;0,081;2

-737;-0,714;-0,103;2

-672;-0,824;0,059;2

-619;-0,972;-0,04;2

-580;-0,723;-0,015;2

-570;-0,769;0,081;2

-600;-0,528;-0,091;2

-663;-0,133;0,009;2

-735;-0,054;-0,003;2

-811;0,316;-0,054;2

-870;-0,654;-0,016;2

-921;-1,11;-0,107;2

-962;-0,747;0,046;2

-1000;-0,911;0,002;2

-1032;-0,673;-0,055;2

-1068;-0,641;0,042;2

-1096;-0,244;-0,017;2

-1123;0,468;0,005;2

-1144;0,609;0,055;2

-1166;0,628;-0,009;2

-1184;0,345;-0,02;2

-1202;0,797;-0,042;2

-1215;1,064;-0,049;2

-1226;0,536;0,025;2

-1237;0,027;-0,113;2

-1249;-0,19;0,031;2

-1265;-0,388;0,015;2

-1278;-0,059;-0,027;2

-1288;0,165;0,049;2

-1297;0,65;-0,069;2

-1302;1,238;0,061;2

-1298;1,197;0,031;2

-1279;0,858;-0,014;2

-1247;0,554;0,054;2

-1202;-0,097;-0,085;2

-1153;-0,219;0,008;2

-1101;-0,436;0,031;2

-1048;-0,053;-0,094;2

-989;0,237;0,069;2

-926;0,378;-0,025;2

-858;0,533;-0,041;2

-788;0,043;0,06;2

-719;-0,275;-0,088;2

-658;-0,381;0,125;2

-607;-0,392;-0,017;2

-575;-0,176;0,054;2

-587;-0,419;0,056;2

-638;-0,299;0,002;2

-718;0,274;0,048;2

-798;0,35;0,001;2

-877;0,606;-0,044;2

-935;0,62;0,076;2

-982;0,858;-0,028;2

-1020;1,054;0,041;2

-1054;0,36;-0,026;2

-1081;0,166;-0,022;2

-1109;-0,183;0,09;2

-1132;-0,228;-0,154;2

-1155;-0,089;-0,021;2

-1180;-0,302;0,024;2

-1203;0,182;-0,059;2

-1227;0,334;0,05;2

-1247;-0,01;-0,02;2

-1266;0,009;-0,018;2

-1281;-0,426;0,087;2

-1295;-0,384;-0,087;2

-1302;0,369;0,063;2

-1308;0,832;0,013;2

-1310;0,512;-0,029;2

-1311;-0,312;0,08;2

-1311;-0,394;0,131;2

-1314;-0,505;0,051;2

-1314;-0,296;-0,014;2

-1301;0,043;-0,079;2

-1277;-0,081;0,089;2

-1239;-0,354;-0,054;2

-1197;-0,482;0,02;2

-1151;-0,135;0,033;2

-1098;0,055;-0,047;2

-1039;0,091;0,087;2

-973;0,732;-0,052;2

-904;1,013;0,024;2

-836;0,957;0,09;2

-771;0,824;-0,108;2

-710;0,739;0,06;2

-657;1,146;0,006;2

-619;1,476;0,019;2

-614;0,979;0,05;2

-638;0,595;-0,082;2

-693;0,551;0,058;2

-758;0,203;0,051;2

-823;0,354;-0,012;2

-878;0,544;0,072;2

-930;0,455;-0,073;2

-972;0,514;0,031;2

-1014;0,67;-0,057;2

-1050;0,488;-0,045;2

-1086;0,16;0,102;2

-1118;0,257;-0,031;2

-1146;0,104;0,04;2

-1173;-0,339;0,005;2

-1192;-0,093;-0,073;2

-1209;0,172;0,049;2

-1225;0,212;-0,057;2

-1237;0,318;0,031;2

-1242;0,409;0,054;2

-1253;1,039;-0,053;2

-1265;1,675;0,123;2

-1279;1,455;-0,025;2

-1295;1,264;-0,013;2

-1311;0,979;0,14;2

-1322;0,083;-0,096;2

-1328;-0,588;0,074;2

-1324;-0,758;0,006;2

-1304;-0,865;-0,034;2

-1272;-0,868;0,089;2

-1230;-0,408;-0,086;2

-1184;-0,181;0,055;2

-1140;-0,358;-0,005;2

-1091;-0,378;-0,045;2

-1042;-0,334;0,144;2

-988;-0,392;-0,052;2

-927;-0,393;-0,002;2

-857;0,116;0,001;2

-789;0,832;-0,061;2

-723;0,843;0,075;2

-670;0,648;-0,053;2

-625;0,402;-0,039;2

-612;0,123;0,06;2

-630;-0,057;-0,104;2

-687;-0,305;0,086;2

-763;-0,129;-0,025;2

-838;-0,224;-0,034;2

-902;-0,643;0,044;2

-954;-0,444;-0,077;2

-998;0,106;0,063;2

-1037;0,157;-0,029;2

-1073;-0,211;-0,062;2

-1105;-0,493;0,099;2

-1129;-0,213;-0,04;2

-1147;-0,028;0,034;2

-1164;-0,196;-0,01;2

-1179;0,015;-0,078;2

-1194;0,245;0,072;2

-1204;0,27;-0,085;2

-1218;0,62;0,03;2

-1230;0,817;0,053;2

-1247;1,152;-0,105;2

-1265;1,025;0,087;2

-1278;0,919;-0,058;2

-1289;1,047;0,005;2

-1288;0,704;0,082;2

-1278;0,589;-0,081;2

-1251;0,382;0,101;2

-1217;0,628;-0,013;2

-1178;0,524;-0,032;2

-1131;-0,469;0,095;2

-1083;-1,095;-0,098;2

-1025;-1,359;0,009;2

-968;-1,486;0,052;2

-904;-0,883;-0,013;2

-836;-1,094;0,065;2

-765;-1,117;-0,03;2

-697;-1,474;0,013;2

-632;-1,717;0,048;2

-580;-1,198;-0,071;2

-548;-1,082;0,048;2

-558;-1,411;-0,017;2

-605;-1,275;-0,02;2

-683;-0,405;0,051;2

-767;0,336;-0,097;2

-845;-0,069;0,043;2

-913;0,178;-0,007;2

-965;0,218;-0,021;2

-1009;-0,407;0,059;2

-1041;-0,751;-0,067;2

-1067;-0,716;0,037;2

-1086;-0,641;0,005;2

-1105;-0,885;-0,005;2

-1122;-0,945;0,113;2

-1147;-0,626;-0,043;2

-1169;-0,205;0,035;2

-1194;-0,187;0,012;2

-1217;-0,608;-0,086;2

-1244;-0,838;0,107;2

-1263;-0,585;-0,032;2

-1279;-0,401;0,054;2

-1288;-0,131;0,031;2

-1292;0,439;-0,061;2

-1295;0,609;0,078;2

-1291;0,844;-0,043;2

-1282;0,717;-0,033;2

-1257;0,079;0,051;2

-1223;0,056;-0,093;2

-1179;0,142;0,044;2

-1135;0,188;0,162;2

-1091;0,221;-0,024;2

-1042;0,071;0,123;2

-986;-0,012;-0,084;2

-915;-0,409;0,052;2

-843;-0,623;0,03;2

-763;-0,028;-0,011;2

-686;-0,195;0,08;2

-617;-0,356;-0,056;2

-571;-0,227;0,034;2

-558;-0,073;0,008;2

-603;0,246;-0,154;2

-684;0,354;0,081;2

-778;0,558;-0,117;2

-862;0,215;-0,007;2

-928;-0,259;0,036;2

-977;-0,5;-0,092;2

-1014;-0,647;0,099;2

-1047;-0,607;-0,006;2

-1072;-0,253;0,025;2

-1095;-0,294;0,08;2

-1114;-0,343;-0,076;2

-1136;-0,278;0,08;2

-1156;-0,343;0,017;2

-1181;-0,29;-0,02;2

-1203;-0,109;0,011;2

-1224;0,116;-0,071;2

-1238;0,545;0,038;2

-1254;0,846;0,007;2

-1269;0,953;-0,028;2

-1282;1;0,083;2

-1281;0,847;-0,07;2

-1265;0,866;-0,009;2

-1231;0,515;0,004;2

-1183;0,469;-0,077;2

-1131;0,348;0,056;2

-1069;0,272;-0,094;2

-1004;0,429;0,115;2

-934;0,409;0,034;2

-867;0,757;-0,201;2

-803;0,603;0,075;2

-734;0,541;-0,056;2

-669;0,369;-0,071;2

-610;-0,242;0,061;2

-578;-0,372;-0,099;2

-588;-0,606;0,044;2

-641;-0,796;0,01;2

-720;-0,591;-0,019;2

-803;-0,418;0,073;2

-880;-0,103;-0,136;2

-943;0,056;0,071;2

-992;0,476;-0,002;2

-1027;0,993;-0,042;2

-1056;0,86;0,105;2

-1077;0,819;0,026;2

-1101;0,851;0,086;2

-1119;0,672;0,031;2

-1141;0,269;-0,08;2

-1162;-0,127;0,063;2

-1185;-0,312;-0,033;2

-1202;-0,276;-0,036;2

-1222;-0,324;0,016;2

-1238;0,298;-0,052;2

-1250;0,442;0,1;2

-1259;0,182;-0,064;2

-1265;-0,06;-0,052;2

-1269;-0,263;0,072;2

-1268;0,141;-0,132;2

-1253;0,215;0,06;2

-1217;0,183;0,031;2

-1171;0,428;-0,023;2

-1116;0,554;0,098;2

-1056;1,045;-0,035;2

-996;1,248;0,033;2

-935;1,093;-0,028;2

-864;1;-0,023;2

-797;0,594;0,076;2

-730;0,442;-0,045;2

-668;0,287;0,008;2

-625;0,028;0,099;2

-606;0,209;-0,032;2

-633;0,371;0,066;2

-691;0,363;-0,065;2

-772;0,289;-0,02;2

-850;0,112;0,075;2

-919;0,248;-0,107;2

-974;0,158;0,138;2

-1016;0,121;-0,015;2

-1054;0,489;0,01;2

-1083;-0,014;0,041;2

-1112;0,089;-0,027;2

-1133;0,086;0,044;2

-1151;-0,01;-0,034;2

-1167;-0,049;-0,023;2

-1187;-0,27;0,059;2

-1203;-0,182;-0,048;2

-1223;-0,146;0,004;2

-1244;-0,525;0,032;2

-1266;-0,607;-0,1;2

-1286;-0,375;0,012;2

-1305;0,081;-0,064;2

-1317;0,338;0,01;2

-1327;0,341;0,068;2

-1328;0,553;-0,067;2

-1323;0,254;0,187;2

-1304;0,324;-0,019;2

-1271;0,352;0,026;2

-1231;0,188;0,067;2

-1184;0,176;-0,066;2

-1134;-0,251;0,05;2

-1072;-0,256;-0,016;2

-1006;0,331;-0,065;2

-929;0,379;0,086;2

-852;0,425;-0,039;2

-773;0,105;0,055;2

-700;0,062;0,014;2

-643;0,61;-0,037;2

-617;0,444;0,065;2

-632;0,665;-0,111;2

-676;0,75;0,04;2

-747;0,313;0,031;2

-819;0,437;-0,067;2

-887;0,427;0,068;2

-947;-0,408;-0,041;2

-996;-0,272;-0,005;2

-1041;-0,324;0,049;2

-1074;-0,121;-0,099;2

-1104;-0,411;0,05;2

-1126;-0,838;0,034;2

-1143;-1,067;-0,012;2

-1158;-1,592;0,073;2

-1173;-1,421;-0,059;2

-1189;-0,764;0,039;2

-1208;-0,302;0,025;2

-1226;-0,216;-0,054;2

-1244;-0,579;0,051;2

-1263;-0,671;-0,109;2

-1280;-0,868;0,029;2

-1301;-0,783;0,021;2

-1313;-0,404;-0,07;2

-1325;-0,222;0,108;2

-1331;-0,031;-0,033;2

-1330;-0,069;0,018;2

-1328;-0,193;0,088;2

-1324;-0,168;-0,087;2

-1308;-0,278;0,072;2

-1281;-0,362;-0,048;2

-1250;-0,307;-0,01;2

-1216;-0,645;0,044;2

-1179;-0,346;-0,084;2

-1133;-0,284;0,07;2

-1079;-0,321;0,053;2

-1013;-0,279;-0,018;2

-943;-0,065;0,072;2

-870;0,053;-0,026;2

-800;-0,123;0,036;2

-732;-0,003;0,003;2

-670;0,23;-0,068;2

-620;-0,036;0,112;2

-602;0,075;-0,023;2

-629;0,165;0,127;2

-687;-0,219;-0,006;2

-762;0,055;-0,088;2

-830;-0,136;0,07;2

-893;-0,568;-0,023;2

-950;-0,28;0,01;2

-997;0,038;0,037;2

-1038;0,09;-0,071;2

-1068;0,245;0,078;2

-1094;0,245;-0,017;2

-1115;-0,132;-0,036;2

-1140;-0,06;0,069;2

-1159;0,019;-0,059;2

-1179;-0,244;0,049;2

-1195;-0,459;-0,004;2

-1212;-0,147;0,051;2

-1226;0,059;0,069;2

-1242;0,087;-0,067;2

-1254;0,082;0,034;2

-1268;-0,125;-0,014;2

-1278;-0,382;-0,077;2

-1287;-0,484;0,108;2

-1297;-0,565;-0,109;2

-1298;-0,67;-0,031;2

-1291;-0,567;0,027;2

-1265;0,002;-0,059;2

-1223;0,275;0,078;2

-1165;0,062;-0,06;2

-1100;0,322;0,046;2

-1026;-0,057;0,059;2

-955;0,166;-0,071;2

-880;0,333;0,103;2

-810;0,382;0,005;2

-742;0,618;-0,021;2

-680;0,206;0,063;2

-630;0,198;-0,117;2

-604;0,074;0,057;2

-625;-0,394;0,004;2

-681;-0,499;-0,039;2

-758;-0,564;0,081;2

-834;0,118;-0,034;2

-897;0,455;0,02;2

-953;0,114;-0,009;2

-993;0,494;-0,069;2

-1029;0,091;0,076;2

-1058;-0,069;-0,071;2

-1088;0,036;-0,016;2

-1115;0,076;0,028;2

-1139;0,758;-0,065;2

-1167;0,719;0,027;2

-1189;0,613;-0,045;2

-1208;0,781;-0,01;2

-1219;0,691;0,065;2

-1231;0,39;-0,076;2

-1240;-0,15;0,016;2

-1252;-0,567;0,018;2

-1261;-0,298;-0,017;2

-1270;-0,354;0,074;2

-1276;-0,198;-0,058;2

-1284;-0,122;0,08;2

-1291;0,184;-0,005;2

-1301;0,406;0,125;2

-1305;0,318;0,054;2

-1295;0,248;-0,058;2

-1268;0,54;0,036;2

-1223;0,614;0,092;2

-1174;0,568;-0,059;2

-1112;0,324;-0,034;2

-1043;0,642;-0,074;2

-965;0,814;0,024;2

-891;-0,088;0,019;2

-815;0,024;0,027;2

-744;-0,358;0,063;2

-681;-0,672;-0,005;2

-642;-0,333;0,022;2

-644;-0,374;0,058;2

-693;-0,194;-0,038;2

-764;0,22;0,07;2

-836;0,045;-0,038;2

-903;-0,161;-0,054;2

-950;-0,146;0,068;2

-990;0,09;-0,074;2

-1022;0,001;0,097;2

-1051;0,301;0,017;2

-1078;0,411;-0,046;2

-1098;0,214;0,068;2

-1118;-0,068;-0,078;2

-1138;-0,321;0,037;2

-1157;-0,554;-0,028;2

-1174;0,069;-0,092;2

-1194;-0,014;0,063;2

-1212;-0,208;-0,025;2

-1230;-0,257;0,037;2

-1245;-0,404;-0,001;2

-1258;-0,291;-0,087;2

-1261;-0,563;0,038;2

-1244;-0,272;-0,046;2

-1208;0,195;-0,019;2

-1154;-0,13;0,065;2

-1090;0,022;-0,059;2

-1016;0,259;0,056;2

-939;0,222;0,029;2

-855;0,345;0,016;2

-773;0,439;0,087;2

-690;0,239;-0,077;2

-618;0,385;0,028;2

-562;0,432;0,004;2

-545;0,092;-0,074;2

-575;0,127;0,069;2

-643;0,256;-0,026;2

-722;-0,04;0,019;2

-800;-0,27;0,025;2

-860;-0,094;-0,072;2

-910;0,383;0,122;2

-946;0,393;-0,034;2

-982;0,079;0,016;2

-1013;0,031;0,034;2

-1044;0,332;-0,097;2

-1075;0,408;0,072;2

-1103;0,408;-0,008;2

-1133;0,413;-0,033;2

-1161;0,164;0,045;2

-1191;0,119;-0,118;2

-1212;0,09;0,081;2

-1231;0,083;-0,013;2

-1241;0,306;-0,037;2

-1248;0,516;0,087;2

-1254;0,411;-0,038;2

-1257;-0,259;0,026;2

-1266;-0,557;-0,04;2

-1270;-0,55;-0,088;2

-1278;-0,385;0,086;2

-1270;-0,002;-0,074;2

-1245;0,075;0,029;2

-1195;-0,097;0,003;2

-1132;0,372;-0,051;2

-1054;0,516;0,064;2

-971;0,501;-0,009;2

-888;0,663;-0,011;2

-807;0,374;0,076;2

-733;0,445;-0,09;2

-678;0,412;0,093;2

-660;0,275;-0,009;2

-685;0,266;-0,016;2

-744;-0,144;0,114;2

-817;-0,313;-0,054;3

-892;-0,326;0,01;3

-958;-0,184;-0,039;3

-1013;0,055;-0,043;3

-1055;-0,141;0,075;3

-1088;0,043;-0,061;3

-1115;0,531;-0,002;3

-1142;0,337;0,049;3

-1162;0,191;-0,023;3

-1184;0,075;0,096;3

-1199;0,314;-0,066;3

-1211;0,38;0,071;3

-1207;0,141;-0,071;3

-1195;-0,018;-0,121;3

-1161;-0,153;0,044;3

-1115;-0,123;-0,02;3

-1059;-0,118;0,018;3

-995;-0,283;0,056;3

-922;-0,091;-0,086;3

-845;-0,208;0,056;3

-774;-0,353;0,02;3

-709;-0,012;0,033;3

-674;0,027;0,059;3

-673;0,265;-0,071;3

-713;-0,001;0,038;3

-783;0,088;-0,047;3

-856;0,35;-0,058;3

-930;0,396;0,119;3

-987;0,861;-0,044;3

-1040;0,619;0,008;3

-1084;0,262;-0,001;3

-1127;0,706;-0,064;3

-1161;0,637;0,139;3

-1192;0,328;0,045;3

-1212;0,909;-0,015;3

-1229;0,838;0,046;3

-1242;0,739;-0,063;3

-1261;0,53;0,011;3

-1276;0,543;-0,002;3

-1285;0,85;0,004;3

-1292;0,907;0,058;3

-1293;0,78;-0,081;3

-1296;0,363;0,085;3

-1283;0,286;-0,001;3

-1256;0,262;0,024;3

-1214;-0,667;0,093;3

-1164;-0,944;-0,033;3

-1097;-0,585;0,045;3

-1025;-0,366;0,005;3

-939;-0,214;-0,136;3

-846;-0,152;0,071;3

-755;0,245;-0,076;3

-680;0,118;0,015;3

-632;-0,464;-0,022;3

-632;-0,217;-0,087;3

-679;-0,341;0,097;3

-757;-0,362;-0,04;3

-844;-0,259;-0,016;3

-921;-0,277;0,055;3

-983;-0,352;-0,051;3

-1024;-0,259;0,067;3

-1059;-0,031;-0,112;3

-1087;-0,17;-0,02;3

-1116;-0,109;0,09;3

-1141;0,168;-0,08;3

-1164;0,603;0,058;3

-1190;0,779;0,006;3

-1207;1,005;-0,039;3

-1224;0,756;0,052;3

-1237;0,472;-0,063;3

-1249;0,192;0,038;3

-1240;0,073;0,007;3

-1212;0,009;-0,058;3

-1163;-0,078;0,039;3

-1103;-0,119;-0,043;3

-1031;0,28;-0,01;3

-950;-0,101;0,009;3

-864;-0,41;-0,062;3

-776;-0,742;0,059;3

-698;-0,587;-0,023;3

-631;-0,09;0,036;3

-604;-0,063;0,077;3

-619;-0,385;-0,091;3

-682;-0,241;0,071;3

-761;-0,106;0,019;3

-848;0,044;-0,081;3

-926;-0,032;0,045;3

-991;-0,119;-0,045;3

-1039;-0,521;0,06;3

-1080;-0,424;-0,027;3

-1112;-0,332;-0,025;3

-1141;-0,508;0,095;3

-1161;-0,237;-0,016;3

-1177;0,595;0,085;3

-1187;0,527;0,022;3

-1196;0,455;-0,083;3

-1211;0,428;0,077;3

-1223;-0,173;-0,16;3

-1238;-0,056;0,012;3

-1250;-0,152;0,046;3

-1264;-0,032;-0,103;3

-1277;-0,168;0,063;3

-1294;-0,315;-0,027;3

-1313;-0,298;0,02;3

-1326;-0,348;0,078;3

-1325;-0,02;-0,093;3

-1301;-0,1;0,009;3

-1262;-0,136;0,054;3

-1206;0,036;0,004;3

-1141;-0,304;0,043;3

-1075;-0,324;-0,04;3

-998;0,092;0,033;3

-918;0,015;0,011;3

-839;-0,134;-0,021;3

-764;-0,272;0,089;3

-690;-0,431;-0,036;3

-624;-0,242;0,034;3

-572;-0,209;-0,001;3

-556;-0,119;-0,088;3

-588;0,197;0,052;3

-664;-0,291;-0,043;3

-750;-0,386;0,018;3

-830;-0,489;0,052;3

-892;-0,349;-0,075;3

-938;-0,159;0,087;3

-977;-0,167;-0,034;3

-1013;0,02;0,019;3

-1052;0,173;0,066;3

-1093;0,136;-0,115;3

-1136;0,328;0,038;3

-1166;0,349;-0,002;3

-1190;0,552;-0,033;3

-1200;0,22;0,057;3

-1209;-0,17;-0,1;3

-1216;-0,17;0,023;3

-1229;-0,484;-0,018;3

-1242;-0,513;-0,004;3

-1263;-0,709;0,112;3

-1282;-0,806;-0,073;3

-1295;-0,91;0,018;3

-1305;-0,881;0,025;3

-1310;-0,69;-0,09;3

-1313;-0,909;0,088;3

-1312;-0,527;0,003;3

-1305;0,038;0,023;3

-1285;-0,103;0,065;3

-1254;-0,172;-0,069;3

-1204;-0,055;0,16;3

-1140;-0,155;0,004;3

-1067;-0,027;-0,013;3

-985;0,284;0,061;3

-906;-0,118;-0,081;3

-827;-0,118;-0,012;3

-757;0,276;-0,14;3

-684;0,326;-0,054;3

-619;0,01;0,076;3

-567;0,067;-0,089;3

-547;0,179;0,042;3

-569;-0,179;-0,012;3

-632;-0,039;-0,061;3

-713;-0,195;0,099;3

-798;-0,569;-0,063;3

-875;-0,362;-0,012;3

-939;0,041;-0,001;3

-990;0,375;-0,078;3

-1028;0,36;0,066;3

-1061;0,188;-0,005;3

-1093;0,37;-0,002;3

-1125;0,376;0,065;3

-1153;0,668;-0,077;3

-1176;0,426;0,029;3

-1191;0,102;-0,002;3

-1208;0,298;-0,032;3

-1226;0,048;0,094;3

-1253;0,167;-0,067;3

-1277;0,16;0,028;3

-1299;-0,168;0,021;3

-1312;-0,095;-0,055;3

-1320;-0,384;0,181;3

-1323;-0,299;-0,004;3

-1321;-0,186;0,052;3

-1325;-0,503;-0,021;3

-1329;-0,208;-0,071;3

-1341;-0,358;0,081;3

-1352;-0,296;-0,051;3

-1355;-0,444;0,014;3

-1345;-0,692;0,047;3

-1314;-0,389;-0,068;3

-1270;-0,164;0,044;3

-1213;-0,282;-0,02;3

-1153;-0,389;0,021;3

-1083;-0,559;0,036;3

-1016;-1,007;-0,099;3

-945;-1,176;0,105;3

-872;-1,127;-0,019;3

-799;-0,827;-0,007;3

-729;-0,688;0,078;3

-670;-0,881;-0,038;3

-624;-0,704;0,075;3

-608;-0,611;0,023;3

-630;-0,724;-0,049;3

-691;-0,757;0,1;3

-761;-0,462;-0,068;3

-833;0,057;0,012;3

-889;0,533;0,058;3

-938;0,861;-0,085;3

-977;0,666;0,085;3

-1016;0,669;-0,035;3

-1051;0,719;0,007;3

-1089;0,589;0,053;3

-1119;0,707;-0,104;3

-1146;1,094;0,101;3

-1170;0,906;-0,014;3

-1195;0,682;0,018;3

-1216;0,318;0,078;3

-1237;0,369;-0,057;3

-1255;0,309;0,038;3

-1268;0,341;-0,009;3

-1277;0,367;-0,035;3

-1285;0,002;0,063;3

-1290;-0,046;-0,178;3

-1292;-0,229;0,051;3

-1304;-0,419;0,02;3

-1316;-0,139;-0,056;3

-1329;-0,645;0,078;3

-1336;-0,505;-0,09;3

-1329;-0,043;0;3

-1302;-0,28;0,023;3

-1255;-0,127;-0,05;3

-1200;-0,341;-0,02;3

-1127;-0,35;-0,019;3

-1052;-0,281;-0,014;3

-978;-0,392;0,048;3

-906;0,242;-0,069;3

-835;0,023;0,065;3

-766;0,232;-0,021;3

-695;0,356;0;3

-634;0,069;0,066;3

-587;0,038;0,018;3

-569;-0,161;0,047;3

-590;-0,309;-0,008;3

-649;-0,438;-0,071;3

-730;-0,709;0,043;3

-812;-0,379;-0,048;3

-883;-0,6;0,02;3

-937;-0,718;0,008;3

-978;-0,243;-0,064;3

-1008;-0,196;0,056;3

-1038;-0,177;-0,092;3

-1063;0,182;0,007;3

-1093;0,083;0,064;3

-1120;0,098;-0,068;3

-1151;0,02;0,058;3

-1173;-0,311;0,087;3

-1203;-0,313;-0,022;3

-1230;-0,14;0,096;3

-1256;-0,389;-0,026;3

-1276;-0,598;0,062;3

-1294;-0,638;-0,001;3

-1302;-0,353;-0,002;3

-1309;0,043;0,104;3

-1311;-0,008;-0,061;3

-1312;0,12;0,105;3

-1313;0,267;0,11;3

-1312;0,12;0,009;3

-1315;-0,018;0,076;3

-1319;0,258;-0,085;3

-1324;0,332;0,024;3

-1317;0,004;0,05;3

-1297;0,159;-0,056;3

-1265;-0,007;-0,037;3

-1214;-0,293;-0,043;3

-1150;0,139;0,016;3

-1077;0,031;0,079;3

-1003;0,211;-0,105;3

-924;0,371;0,079;3

-857;0,292;-0,011;3

-795;0,057;-0,016;3

-749;0,196;0,181;3

-712;-0,1;-0,069;3

-686;0,358;0,046;3

-669;1;-0,011;3

-674;0,648;-0,023;3

-711;0,258;0,101;3

-762;-0,213;-0,082;3

-819;-0,19;0,042;3

-870;-0,223;0,005;3

-914;-0,261;-0,063;3

-951;-0,284;0,065;3

-991;-0,442;-0,019;3

-1032;-0,342;-0,005;3

-1078;-0,023;0,026;3

-1121;0,271;-0,06;3

-1167;0,156;0,052;3

-1201;-0,328;-0,01;3

-1231;-0,616;0,042;3

-1246;-0,72;0,076;3

-1251;-0,247;-0,065;3

-1246;0,101;-0,039;3

-1247;0,388;-0,005;3

-1247;0,415;-0,025;3

-1258;0,421;0,061;3

-1272;0,009;-0,101;3

-1291;-0,152;0,041;3

-1316;-0,22;-0,053;3

-1332;0,233;-0,059;3

-1332;0,28;0,076;3

-1305;0,169;-0,083;3

-1263;-0,138;0,047;3

-1214;-0,524;0,011;3

-1165;-0,179;-0,045;3

-1123;-0,257;0,078;3

-1076;-0,125;-0,049;3

-1027;0,177;-0,002;3

-972;0,132;0,092;3

-912;0,389;-0,147;3

-841;0,637;-0,01;3

-772;0,759;-0,045;3

-692;0,923;-0,013;3

-620;0,72;0,049;3

-571;0,922;-0,082;3

-573;0,907;0,087;3

-625;0,285;-0,035;3

-707;-0,079;-0,047;3

-799;-0,427;0,063;3

-887;-0,081;-0,076;3

-959;-0,123;0,074;3

-1017;-0,45;0,12;3

-1058;-0,095;-0,036;3

-1083;0,336;0,122;3

-1096;0,231;-0,033;3

-1104;0,468;0,014;3

-1118;0,882;0,015;3

-1137;1,216;-0,065;3

-1168;0,67;0,071;3

-1202;-0,328;-0,056;3

-1238;-0,661;0,016;3

-1275;-0,668;0,046;3

-1310;-0,569;-0,076;3

-1330;-0,616;0,134;3

-1344;-0,449;0,003;3

-1332;-0,359;0,031;3

-1302;0,174;0,097;3

-1258;0,363;-0,102;3

-1208;0,428;0,086;3

-1156;0,37;-0,016;3

-1104;0,377;-0,022;3

-1065;0,134;0,112;3

-1019;0,332;-0,074;3

-975;-0,235;0,01;3

-928;-0,687;0,024;3

-867;-0,398;-0,018;3

-802;-0,527;0,119;3

-752;-0,506;-0,082;3

-738;-0,247;0,002;3

-763;-0,22;0,013;3

-819;-0,525;-0,047;3

-885;-0,396;0,052;3

-960;-0,016;-0,024;3

-1024;0,119;0,011;3

-1080;0,265;0,057;3

-1122;0,635;-0,109;3

-1147;0,528;0,076;3

-1164;0,661;-0,014;3

-1173;1,049;-0,055;3

-1187;0,999;0,062;3

-1208;1,006;-0,068;3

-1240;1,049;0,078;3

-1272;0,941;-0,012;3

-1305;0,79;0,07;3

-1329;0,48;0,055;3

-1345;0,512;-0,076;3

-1344;0,832;0,074;3

-1341;0,443;0,005;3

-1329;0,618;-0,06;3

-1321;0,082;0,095;3

-1319;-0,274;-0,053;3

-1320;-0,017;0,023;3

-1310;-0,573;0,025;3

-1294;-0,543;-0,084;3

-1270;-0,43;0,07;3

-1242;-0,265;-0,042;3

-1198;-0,031;-0,007;3

-1145;-0,255;0,083;3

-1089;-0,099;-0,076;3

-1029;-0,104;0,059;3

-983;0,114;-0,006;3

-959;-0,305;-0,061;3

-963;-0,9;0,121;3

-994;-1,056;-0,061;3

-1041;-0,715;0,027;3

-1096;-0,371;-0,001;3

-1146;-0,282;-0,052;3

-1188;-0,108;0,094;3

-1214;0,537;-0,047;3

-1227;0,531;-0,004;3

-1230;0,253;0,031;3

-1232;0,854;-0,08;3

-1235;0,535;0,072;3

-1245;0,329;-0,088;3

-1263;0,337;-0,002;3

-1283;0,016;0,074;3

-1295;0,096;-0,074;3

-1285;-0,141;0,067;3

-1259;-0,032;-0,006;3

-1217;0,338;-0,027;3

-1175;0,382;0,051;3

-1130;0,297;-0,081;3

-1091;0,235;0,092;3

-1055;0,093;-0,006;3

-1029;0,2;-0,045;3

-1006;-0,168;0,081;3

-983;-0,537;-0,123;3

-949;-0,387;0,005;3

-909;-0,396;-0;3

-863;-0,177;-0,039;3

-827;-0,328;0,131;3

-808;-0,16;-0,051;3

-821;-0,136;0,017;3

-852;-0,039;0,047;3

-904;0,154;-0,134;3

-960;0,265;0,096;3

-1015;-0,113;-0,014;3

-1058;-0,153;0,005;3

-1091;-0,201;0,107;3

-1120;-0,054;-0,048;3

-1142;0,068;0,081;3

-1163;-0,177;0;3

-1183;-0,491;-0,006;3

-1202;-0,851;0,063;3

-1221;-0,82;-0,082;3

-1236;-0,658;0,098;3

-1255;-0,186;-0,014;3

-1264;-0,064;0,005;3

-1268;-0,249;0,074;3

-1261;0,245;-0,06;3

-1255;-0,028;0,119;3

-1248;0,044;0,009;3

-1250;0,529;-0,083;3

-1253;0,401;0,087;3

-1260;0,644;-0,059;3

-1267;0,81;0,011;3

-1277;0,841;0,017;3

-1284;1,154;-0,071;3

-1285;1,004;0,06;3

-1284;0,738;-0,006;3

-1277;0,59;0,028;3

-1267;0,391;0,051;3

-1246;0,108;-0,113;3

-1214;-0,361;0,053;3

-1170;-0,02;0,044;3

-1122;0,225;-0,015;3

-1079;0,07;0,098;3

-1049;0,413;-0,083;3

-1023;0,326;0,04;3

-1003;0,004;-0,012;3

-976;-0,213;0,031;3

-943;-0,414;0,12;3

-893;0,108;-0,053;3

-830;0,361;0,024;3

-755;-0,253;-0,024;3

-686;-0,102;-0,1;3

-624;-0,054;0,058;3

-595;0,148;-0,09;3

-605;0,369;-0,013;3

-655;0,086;0,058;3

-731;0,086;-0,095;3

-817;0,023;0,069;3

-900;-0,29;-0,006;3

-969;-0,489;-0,041;3

-1027;-0,53;0,1;3

-1066;-0,221;-0,05;3

-1085;-0,347;0,069;3

-1095;-0,468;-0,09;3

-1094;0,034;-0,001;3

-1098;-0,428;0,061;3

-1107;-0,345;-0,072;3

-1129;-0,413;0,031;3

-1153;-0,265;0,002;3

-1190;-0,137;-0,053;3

-1228;-0,56;0,083;3

-1261;-0,587;-0,046;3

-1282;-0,27;-0,014;3

-1286;-0,539;0,03;3

-1278;-0,44;-0,089;3

-1271;-0,336;0,038;3

-1263;-0,669;-0,078;3

-1262;-0,117;-0,015;3

-1270;-0,017;0,037;3

-1282;-0,086;-0,06;3

-1296;0,312;0,093;3

-1299;-0,101;-0,014;3

-1290;-0,14;0,005;3

-1264;0,074;0,09;3

-1222;0,339;-0,079;3

-1165;0,153;0,064;3

-1110;-0,465;-0,005;3

-1057;-0,285;-0,039;3

-1016;-0,266;0,04;3

-982;-0,321;-0,06;3

-956;0,015;0,036;3

-922;0,121;-0,003;3

-887;-0,098;-0,068;3

-842;-0,087;0,056;3

-789;0,001;-0,035;3

-726;-0,213;-0,044;3

-672;-0,297;0,016;3

-637;-0,263;-0,039;3

-641;-0,209;0,045;3

-685;-0,281;-0,042;3

-751;0,065;0,093;3

-833;0,021;0,081;3

-910;0,252;-0,093;3

-977;0,255;0,048;3

-1034;0,053;-0,017;3

-1071;-0,061;-0,016;3

-1100;-0,001;0,164;3

-1116;-0,155;-0,085;3

-1128;-0,221;0,103;3

-1131;-0,417;0,034;3

-1137;-0,18;-0,04;3

-1144;-0,45;0,083;3

-1158;-0,5;-0,058;3

-1179;-0,341;0,006;3

-1209;-0,22;0,055;3

-1241;0,394;-0,089;3

-1271;0,358;0,082;3

-1298;0,244;-0,015;3

-1315;0,371;0,007;3

-1326;0,371;0,04;3

-1324;0,394;-0,1;3

-1319;0,14;0,07;3

-1306;-0,258;0,043;3

-1293;-0,441;0,006;3

-1282;-0,362;0,111;3

-1282;-0,068;-0,069;3

-1288;-0,32;0,045;3

-1300;-0,157;0,003;3

-1312;-0,216;-0,004;3

-1316;-0,11;0,07;3

-1305;0,107;-0,076;3

-1282;-0,02;-0,01;3

-1247;-0,26;0,022;3

-1196;-0,316;-0,023;3

-1136;-0,569;0,103;3

-1065;-0,469;-0,092;3

-994;-0,151;0,027;3

-920;-0,515;-0,051;3

-851;-0,557;-0,078;3

-786;-0,294;0,023;3

-730;0,004;-0,059;3

-694;-0,174;0,042;3

-690;-0,703;-0,092;3

-721;-0,596;-0,045;3

-773;-0,684;0,047;3

-831;-0,946;-0,04;3

-881;-0,701;-0,001;3

-921;-0,917;0,126;3

-954;-1,066;-0,087;3

-982;-0,79;0,188;3

-1012;-0,778;0,038;3

-1040;-0,522;0,042;3

-1070;-0,022;0,107;3

-1094;0,016;-0,074;3

-1116;-0,315;0,081;3

-1125;-0,036;-0,002;3

-1137;0,122;-0,058;3

-1150;-0,344;0,104;3

-1171;-0,336;0,021;3

-1194;-0,3;0,048;3

-1223;-0,496;-0,01;3

-1239;-0,268;-0,009;3

-1241;-0,502;0,062;3

-1218;-0,587;-0,025;3

-1173;-0,299;0,024;3

-1113;-0,357;0,074;3

-1046;-0,576;-0,076;3

-976;-0,608;0,071;3

-918;-0,396;-0,027;3

-880;-0,17;-0,029;3

-866;-0,096;0,064;3

-880;0,061;-0,077;3

-914;-0,094;0,069;3

-964;0,001;-0,007;3

-1012;0,373;-0,02;3

-1062;0,182;0,031;3

-1102;-0,039;-0,078;3

-1130;0,181;-0,079;3

-1143;-0,037;0,04;3

-1146;0,025;-0,073;3

-1142;0,191;0,113;3

-1143;0,251;-0,056;3

-1151;-0,342;-0,015;3

-1166;-0,342;0,036;3

-1188;0,087;-0,068;3

-1208;0,067;0,11;3

-1214;-0,173;-0,047;3

-1196;0,308;-0,048;3

-1153;0,417;0,088;3

-1094;0,404;-0,078;3

-1025;0,246;0,069;3

-948;0,251;-0,005;3

-867;0,358;-0,039;3

-789;0,208;0,087;3

-718;-0,026;-0,029;3

-662;-0,459;-0,195;3

-625;-0,375;-0,006;3

-625;-0,111;-0,056;3

-659;-0,274;0,101;3

-721;-0,442;-0,002;3

-784;-0,281;0,024;3

-852;-0,319;0,037;3

-902;-0,445;-0,071;3

-947;-0,367;0,022;3

-982;-0,73;-0,049;3

-1020;-0,822;-0,179;3

-1054;-0,891;0,072;3

-1089;-0,742;-0,069;3

-1120;-0,72;0,078;3

-1143;-0,386;-0,031;3

-1157;-0,156;0,023;3

-1171;-0,356;0,06;3

-1179;-0,398;-0,123;3

-1189;-0,405;0,089;3

-1198;-0,177;0,017;3

-1209;0,154;-0,021;3

-1220;-0,238;0,082;3

-1235;-0,078;-0,085;3

-1253;0,089;0,065;3

-1272;-0,178;0,002;3

-1291;-0,338;-0,066;3

-1302;-0,205;0,059;3

-1300;-0,181;-0,078;3

-1281;-0,009;0,086;3

-1247;0,075;0,048;3

-1202;0,223;-0,06;3

-1144;0,005;0,082;3

-1084;0,159;-0,04;3

-1028;0,093;-0,002;3

-980;0,16;0,09;3

-936;0,084;-0,113;3

-891;-0,355;0,119;3

-841;-0,461;-0,035;3

-781;-0,286;0,014;3

-718;-0,513;0,027;3

-656;-0,137;-0,058;3

-602;0,038;0,075;3

-558;-0,185;-0;3

-552;-0,115;-0,052;3

-592;-0,437;0,058;3

-674;-0,238;-0,104;3

-766;-0,179;0,035;3

-846;-0,07;-0,022;3

-906;0,466;-0,056;3

-944;0,684;0,079;3

-976;0,276;-0,059;3

-1010;0,318;-0,045;3

-1044;0,391;0,009;3

-1078;0,793;-0,069;3

-1113;0,593;0,066;3

-1141;0,157;-0,022;3

-1175;0,221;-0,017;3

-1203;0,652;0,053;3

-1226;0,708;-0,068;3

-1241;0,578;0,074;3

-1252;0,179;0,004;3

-1258;0,253;-0,016;3

-1266;0,06;0,07;3

-1272;0,202;0,035;3

-1278;-0,217;0,015;3

-1286;-0,152;-0,016;3

-1293;0,259;-0,057;3

-1311;-0,175;0,093;3

-1332;-0,139;-0,076;3

-1353;0,217;0,013;3

-1369;0,048;0,015;3

-1372;0,256;-0,064;3

-1366;0,105;0,13;3

-1341;-0,353;-0,072;3

-1306;-0,065;0,015;3

-1262;-0,199;0,136;3

-1220;0,02;-0,07;3

-1184;-0,141;0,079;3

-1153;-0,158;-0,065;3

-1125;-0,148;0,025;3

-1088;-0,481;0,043;3

-1048;-0,043;-0,103;3

-995;0,021;0,073;3

-944;-0,153;-0,037;3

-879;-0,137;0,018;3

-808;-0,307;0,068;3

-731;-0,247;-0,064;3

-665;-0,344;0,074;3

-607;-0,229;0,01;3

-575;0,319;-0,072;3

-576;0,352;0,086;3

-622;0,016;-0,016;3

-703;0,323;0,095;3

-794;0,359;0,038;3

-872;0,781;-0,072;3

-927;0,758;0,037;3

-960;0,326;-0,057;3

-976;0,326;-0,027;3

-997;0,003;0,038;3

-1018;-0,151;-0,107;3

-1055;0,078;0,095;3

-1092;0,139;-0,044;3

-1134;0,446;-0,022;3

-1172;0,667;0,056;3

-1204;0,612;-0,059;3

-1220;0,471;0,019;3

-1229;-0,06;0,086;3

-1227;-0,107;-0,033;3

-1220;0,06;0,072;3

-1214;0,232;-0,05;3

-1213;0,412;0,012;3

-1218;0,038;0,001;3

-1230;-0,281;-0,071;3

-1251;-0,448;0,087;3

-1276;-0,199;-0,109;3

-1300;-0,262;0,002;3

-1323;-0,643;0,013;3

-1332;-0,308;-0,07;3

-1340;-0,215;0,147;3

-1333;0,008;-0,092;3

-1320;0,189;0,03;3

-1288;-0,04;0,056;3

-1246;0,077;-0,076;3

-1194;0,025;0,093;3

-1151;-0,55;-0,012;3

-1115;-0,022;-0,025;3

-1081;0,038;0,096;3

-1047;0,199;-0,073;3

-1016;0,231;0,069;3

-974;-0,047;0,008;3

-918;0,287;-0,058;3

-856;0,347;0,057;3

-791;0,286;-0,07;3

-738;0,653;0,014;3

-716;0,502;0,025;3

-734;0,68;-0,094;3

-780;0,523;0,093;3

-845;0,004;-0,056;3

-910;0,175;0,025;3

-970;0,158;0,049;3

-1011;0,277;-0,061;3

-1045;0,062;0,068;3

-1061;0,03;-0,025;3

-1072;0,168;0,047;3

-1081;-0,031;0,06;3

-1099;0,059;-0,07;3

-1120;0,062;0,07;3

-1149;0,006;0,001;3

-1179;0,311;-0,089;3

-1205;0,35;0,064;3

-1216;0,54;-0,04;3

-1198;0,5;0,064;3

-1161;0,277;0,017;3

-1098;0,002;-0,043;3

-1029;-0,168;0,087;3

-951;-0,083;-0,058;3

-877;0,006;0,027;3

-811;-0,197;0,018;3

-752;-0,05;-0,064;3

-721;-0,085;0,071;3

-720;-0,045;-0,058;3

-758;-0,037;-0,004;3

-817;-0,024;0,065;3

-884;0,301;-0,068;3

-944;0,168;0,097;3

-996;0,164;-0,033;3

-1032;0,294;-0,007;3

-1062;0,166;0,076;3

-1082;0,144;-0,102;3

-1099;0,151;0,085;3

-1115;-0,288;-0,021;3

-1136;-0,237;-0,044;3

-1160;0,017;0,201;3

-1187;-0,038;-0,076;3

-1213;0,319;0,02;3

-1240;-0,041;0,019;3

-1262;-0,111;-0,048;3

-1277;0,228;0,079;3

-1275;0,312;0,013;3

-1250;0,675;0,019;3

-1213;0,344;0,016;3

-1157;0,062;-0,06;3

-1099;0,071;0,034;3

-1037;0,016;-0,063;3

-980;-0,103;0,116;3

-925;-0,4;0,048;3

-874;-0,575;-0,081;3

-821;-0,232;0,053;3

-770;-0,307;-0,005;3

-711;-0,286;-0,02;3

-658;-0,298;0,108;3

-628;-0,431;-0,1;3

-634;-0,207;0,051;3

-676;-0,16;-0;3

-736;-0,105;-0,023;3

-802;-0,077;0,071;3

-866;0,317;-0,038;3

-920;0,08;0,043;3

-978;-0,691;0,026;3

-1030;-0,43;-0,052;3

-1080;-0,333;0,098;3

-1119;-0,436;-0,013;3

-1146;0,022;-0,004;3

-1165;-0,108;0,039;3

-1181;0,164;-0,07;3

-1190;0,37;0,022;3

-1195;0,37;-0,024;3

-1200;0,405;0,035;3

-1203;0,67;0,066;3

-1216;1,069;-0,057;3

-1230;0,799;0,142;3

-1253;0,493;-0;3

-1267;0,496;-0,006;3

-1283;0,239;0,085;3

-1286;0,342;-0,058;3

-1285;0,285;0,034;3

-1270;0,076;-0,006;3

-1243;0,224;-0,023;3

-1203;-0,064;0,099;3

-1163;0,07;-0,05;3

-1124;0,232;0,016;3

-1088;-0,306;0,002;3

-1058;-0,016;-0,091;3

-1028;-0,381;0,093;3

-1005;-0,613;-0,089;3

-976;-0,373;0,012;3

-948;-0,419;0,038;3

-920;-0,184;-0,071;3

-900;-0,286;0,066;3

-882;-0,193;-0,016;3

-873;-0,013;-0,032;3

-878;-0,248;0,071;3

-897;-0,342;-0,061;3

-929;0,005;0,073;3

-961;-0,35;-0,107;3

-995;-0,212;0,013;3

-1022;0,088;0,08;3

-1044;0,026;-0,095;3

-1062;0,267;-0,001;3

-1072;0,309;0,002;3

-1085;-0,069;0,008;3

-1099;0,098;0,11;3

-1125;0,44;0,018;3

-1153;0,183;0,02;3

-1185;0,098;0,033;3

-1211;0,293;-0,07;3

-1234;0,038;0,027;3

-1250;0,001;-0,048;3

-1263;0,109;0,023;3

-1272;-0,025;0,018;3

-1276;0,607;-0,067;3

-1277;0,349;0,074;3

-1277;0,456;-0,013;3

-1279;0,947;0;3

-1284;0,233;0,069;3

-1296;0,272;-0,083;3

-1304;0,289;0,073;3

-1318;0,061;-0,003;3

-1332;0,225;-0,015;3

-1348;0,132;0,083;3

-1354;0,213;-0,137;3

-1343;0,394;0,038;3

-1315;0,542;-0,015;3

-1282;0,916;-0,05;3

-1239;0,793;0,064;3

-1198;0,469;-0,067;3

-1152;0,318;0,044;3

-1111;0,089;0,035;3

-1071;0,235;-0,066;3

-1037;0,178;0,042;3

-1003;0,023;-0,014;3

-965;0,012;0,012;3

-921;-0,297;0,059;3

-864;-0,121;-0,094;3

-802;-0,134;0,004;3

-742;-0,266;-0,006;3

-684;0,07;-0,018;3

-640;-0,276;0,028;3

-617;-0,162;-0,114;3

-628;-0,029;0,055;3

-663;0,145;0,004;3

-718;0,281;-0,045;3

-771;0,117;0,081;3

-822;0,122;-0,085;3

-865;-0,076;0,013;3

-900;-0,233;0,052;3

-931;0,091;-0,082;3

-963;-0,157;0,076;3

-990;-0,345;-0,089;3

-1014;-0,141;0,102;3

-1033;-0,479;0,023;3

-1048;-0,262;-0,069;3

-1060;-0,462;0,065;3

-1072;-0,404;0,064;3

-1087;-0,122;-0,022;3

-1099;0,129;0,054;3

-1117;0,534;-0,07;3

-1134;0,388;0,073;3

-1156;0,173;-0,018;3

-1173;0,59;-0,004;3

-1189;0,493;0,1;3

-1198;0,374;-0,074;3

-1206;0,135;0,056;3

-1210;0,28;0,002;3

-1218;0,709;-0,041;3

-1222;0,61;0,089;3

-1230;0,464;-0,059;3

-1233;0,511;0,044;3

-1237;0,311;0,005;3

-1240;0,614;-0,052;3

-1246;0,477;0,074;3

-1251;0,371;-0,057;3

-1260;0,317;0,008;3

-1275;0,167;0,049;3

-1288;0,337;-0,072;3

-1303;0,292;0,127;3

-1307;0,158;-0,039;3

-1298;0,302;-0,002;3

-1274;0,275;0,059;3

-1237;0,41;-0,089;3

-1189;0,672;0,024;3

-1132;0,326;-0,017;3

-1073;0,117;-0,028;3

-1007;0,261;0,047;3

-944;0,372;-0,073;3

-884;0,201;0,042;3

-828;-0,025;0,023;3

-770;-0,046;-0,068;3

-725;-0,242;0,089;3

-694;-0,362;-0,028;3

-695;-0,328;0,004;3

-724;-0,407;0,038;3

-774;-0,071;-0,066;3

-828;-0,125;0,096;3

-873;-0,413;-0,043;3

-910;-0,496;0,013;3

-936;-0,233;0,053;3

-958;0,324;-0,099;3

-976;0,39;0,071;3

-1002;-0,087;-0,02;3

-1032;0,229;0,001;3

-1073;0,333;0,09;3

-1106;0,53;-0,088;3

-1133;0,61;0,066;3

-1148;0,47;-0,113;3

-1160;0,464;-0,052;3

-1164;0,452;0,088;3

-1177;0,506;-0,056;3

-1190;0,608;0,057;3

-1206;0,191;0,005;3

-1219;0,125;-0,086;3

-1233;0,595;0,071;3

-1239;0,632;-0,05;3

-1227;0,596;0,026;3

-1206;0,298;-0,025;3

-1171;0,152;-0,084;3

-1138;0,107;0,063;3

-1103;0,088;-0,037;3

-1067;0,11;0,031;3

-1028;-0,095;0,067;3

-975;-0,133;-0,081;3

-912;-0,082;0,061;3

-833;0,042;0,02;3

-756;0,259;-0,002;3

-678;-0,153;0,073;3

-610;-0,203;-0,08;3

-556;-0,062;0,07;3

-525;0,05;-0,006;3

-524;0,207;-0,044;3

-562;0,19;0,071;3

-620;0,485;-0,019;3

-692;0,462;0,032;3

-763;0,161;0,019;3

-830;0,444;-0,078;3

-892;0,578;0,08;3

-948;0,46;-0,064;3

-1000;0,303;-0,02;3

-1040;-0,128;0,02;3

-1068;-0,35;-0,067;3

-1088;-0,471;0,056;3

-1104;-0,291;-0,039;3

-1125;-0,007;0,002;3

-1148;-0,021;0,057;3

-1171;0,161;-0,03;3

-1198;0,032;0,067;3

-1221;-0,182;-0,039;3

-1243;-0,102;0;3

-1258;-0,094;0,072;3

-1264;0,173;-0,089;3

-1259;0,386;0,026;3

-1250;0,486;-0,008;3

-1222;0,658;-0,094;3

-1194;0,507;0,095;3

-1154;0,376;-0,066;3

-1108;-0,027;-0,032;3

-1049;-0,412;-0,028;3

-972;-0,174;-0,019;3

-888;-0,19;0,076;3

-790;0,075;-0,044;3

-690;0,333;0,004;3

-585;0,149;0,034;3

-488;0,33;-0,083;3

-414;0,268;-0,039;3

-383;0,263;-0,036;3

-405;0,48;0,039;3

-464;0,171;-0,048;3

-546;-0,171;-0,081;3

-630;-0,415;0,143;3

-714;-0,271;-0,157;3

-786;-0,181;-0,025;3

-851;-0,251;0,122;3

-905;0,158;-0,222;3

-957;0,375;0,16;3

-1007;0,341;0,018;3

-1043;0,486;-0,071;3

-1073;0,466;0,08;3

-1091;0,518;-0,074;3

-1104;0,613;0,001;3

-1114;0,457;0,056;3

-1132;0,432;-0,078;3

-1152;0,002;0,206;3

-1183;-0,216;-0,038;3

-1214;-0,086;0,126;3

-1245;-0,263;0,047;3

-1266;-0,135;-0,071;3

-1273;0,024;0,062;3

-1256;-0,084;-0,01;3

-1226;-0,032;-0,046;3

-1185;-0,146;0,082;3

-1138;-0,196;-0,101;3

-1087;-0,386;0,054;3

-1036;-0,433;0,005;3

-972;0,081;0,122;3

-899;-0,063;0,059;3

-822;-0,031;0,064;3

-738;-0,51;0,037;3

-664;-0,678;0,011;3

-598;-0,264;-0,025;3

-546;-0,178;0,105;3

-513;0,137;-0,056;3

-506;0,202;-0,102;3

-538;0,16;-0,106;3

-593;0,453;-0,071;3

-660;0,243;0,072;3

-724;0,148;-0,036;3

-790;0,277;0,007;3

-855;0,183;0,081;3

-919;0,431;-0,075;3

-975;0,459;0,086;3

-1021;0,342;0,002;3

-1055;0,554;-0,039;3

-1082;0,363;-0,03;3

-1108;0,379;-0,07;3

-1131;0,206;0,063;3

-1159;0,14;-0,024;3

-1185;0,329;-0,046;3

-1215;0,67;0,097;3

-1242;0,556;-0,047;3

-1269;0,249;0,019;3

-1285;0,053;0,016;3

-1301;0,149;-0,087;3

-1308;0,292;0,06;3

-1314;0,325;-0,058;3

-1312;0,247;-0,033;3

-1306;0,021;0,028;3

-1286;0,098;-0,1;3

-1261;0,212;0,074;3

-1230;0,197;0,03;3

-1195;0,176;-0,028;3

-1156;0,087;0,068;3

-1111;-0,172;-0,079;3

-1055;-0,288;0,047;3

-993;-0,367;0,036;3

-924;0,02;-0,044;3

-846;0,145;0,093;3

-766;0,471;-0,056;3

-681;0,421;0,049;3

-610;-0,031;0,013;3

-554;-0,054;0,101;3

-524;-0,234;0,038;3

-536;-0,376;-0,074;3

-585;-0,203;-0,03;3

-662;-0,063;0,029;3

-741;0,275;-0,077;3

-815;0,06;0,064;3

-876;0,262;0,012;3

-923;0,495;-0,011;3

-957;0,174;0,087;3

-979;0,145;-0,108;3

-1003;0,113;0,092;3

-1029;-0,036;0,085;3

-1063;0,036;-0,003;3

-1095;0,005;0,09;3

-1133;-0,049;-0,105;3

-1172;-0,108;0,051;3

-1214;-0,089;0,012;3

-1252;0,054;-0,021;3

-1283;-0,2;0,143;3

-1305;-0,232;-0,08;3

-1314;-0,291;0,014;3

-1318;-0,756;-0,117;3

-1322;-0,932;-0,059;3

-1327;-1,042;0,2;3

-1331;-1,036;0,04;3

-1338;-0,695;0,02;3

-1338;-0,526;0,047;3

-1335;0,051;-0,03;3

-1315;0,18;0,076;3

-1288;0,077;0,005;3

-1248;-0,013;0,026;3

-1204;-0,412;0,055;3

-1155;-0,245;-0,121;3

-1103;-0,174;0,084;3

-1041;-0,039;-0,004;3

-973;0,176;-0,005;3

-900;-0,291;0,052;3

-832;-0,544;-0,056;3

-766;-0,35;0,053;3

-695;-0,172;0,098;3

-627;0,013;-0,034;3

-568;-0,196;0,042;3

-529;-0,188;-0,063;3

-534;-0,326;0,022;3

-581;-0,185;0,054;3

-657;0,109;-0,08;3

-741;-0,026;0,21;3

-821;-0,255;-0,107;3

-889;-0,283;0,003;3

-947;-0,25;0,029;3

-994;-0,07;-0,096;3

-1032;-0,134;0,118;3

-1067;-0,291;0,046;3

-1093;-0,24;-0,053;3

-1120;-0,573;0,13;3

-1148;-0,472;-0,055;3

-1178;-0,482;0,09;3

-1205;-0,535;-0,003;3

-1234;-0,512;0,059;3

-1255;-0,465;0,191;3

-1282;-0,162;-0,15;3

-1306;-0,266;0,062;3

-1328;-0,856;0,034;3

-1341;-0,846;-0,024;3

-1349;-0,268;0,063;3

-1352;0,17;-0,072;3

-1355;0,479;0,059;3

-1353;0,261;0,032;3

-1343;0,592;-0,06;2

-1322;0,562;0,08;2

-1296;0,537;0,006;2

-1263;0,198;0,007;2

-1225;-0,054;0,088;2

-1182;0,166;-0,023;2

-1129;0,362;0,091;2

-1074;0,125;-0,008;2

-1014;0,263;-0,009;2

-954;0,437;0,061;2

-888;0,305;-0,058;2

-817;-0,005;0,025;2

-740;0,004;0,053;2

-664;0,312;-0,023;2

-594;-0,019;0,088;2

-531;-0,359;-0,066;2

-493;-0,325;0,054;2

-493;-0,314;0,028;2

-547;-0,19;-0,054;2

-641;-0,289;0,069;2

-749;-0,508;-0,06;2

-852;-0,558;-0,192;2

-933;-0,422;0,123;2

-1000;-0,58;0,09;2

-1051;-0,874;0,272;2

-1089;-0,208;-0,051;2

-1107;0,453;0,058;2

-1120;-0,066;0,441;2

-1126;-0,256;-0,558;2

-1141;0,032;0,245;2

-1161;0,202;0,081;2

-1186;0,404;-0,106;2

-1188;0,332;0,114;2

-1154;0,272;0,661;2

-1087;-0,027;-0,242;2

-995;-0,195;0,364;2

-891;-0,228;-0,654;2

-783;-0,513;-1,35;2

-661;-0,44;0,452;2

-525;-0,428;-0,699;2

-373;-0,848;-0,082;2

-198;-0,605;0,443;2

-6;-0,677;0,01;2

206;-0,509;-0,803;2

422;-0,258;0,143;2

638;-0,405;0,989;2

826;-0,276;-0,27;2

983;0,012;0,603;2

1097;-0,053;-1,071;2

1174;-0,109;-0,411;2

1197;-0,256;-0,25;2

1146;-0,186;0,153;2

956;-0,029;0,34;2

628;0,007;0,079;2

277;0,014;0,278;2

3;-0,162;0,251;2

-147;-0,244;0,092;2

-214;-0,51;0,386;2

-238;-0,77;0,103;2

-241;-0,724;0,094;2

-252;-0,562;0,485;2

-270;-0,476;-0,422;2

-297;-0,345;1,357;2

-330;-0,352;-0,289;2

-376;-0,271;0,168;2

-429;-0,313;-0,652;2

-482;-0,46;-0,195;2

-533;-0,361;0,352;2

-578;-0,318;-0,089;2

-620;-0,203;0,097;2

-661;-0,28;0,207;2

-703;-0,392;0,162;2

-743;-0,218;-0,05;2

-786;-0,124;-0,02;2

-826;0,042;-0,039;2

-868;0,078;0,161;2

-909;-0,016;-0,073;2

-949;-0,126;0,004;2

-990;-0,1;0,145;2

-1026;0,054;0,028;2

-1063;0,094;0,093;2

-1100;0,024;1,174;2

-1137;0,235;-0,076;2

-1167;-0,074;0,096;2

-1201;-0,227;-0,109;2

-1230;-0,289;0,011;2

-1258;-0,396;-0,371;2

-1277;-0,128;-0,084;2

-1288;-0,396;0,016;2

-1286;-0,291;-0,067;2

-1268;-0,306;0,069;2

-1241;-0,322;0,101;2

-1201;-0,073;-0,041;2

-1155;-0,071;-0,061;2

-1099;-0,099;-0,002;2

-1033;-0,149;0,014;2

-954;-0,347;0,056;2

-850;-0,023;-0,126;2

-736;0,223;0,082;2

-623;0,155;-0,069;2

-540;0,047;-0,072;2

-528;0,033;0,004;2

-601;0,031;-0,908;2

-720;-0,272;0,079;2

-843;-0,395;-0,121;2

-950;0,168;-0,214;2

-1035;0,157;-0,147;2

-1107;0,211;0,662;2

-1161;0,324;0,419;2

-1208;0,437;0,54;2

-1241;0,6;-0,725;2

-1273;0,275;0,199;2

-1294;0,221;1,126;2

-1317;0,365;-0,359;2

-1334;0,607;-0,247;2

-1349;0,491;0,231;2

-1359;0,309;0,612;2

-1360;0,254;-0,411;2

-1337;0,757;0,18;2

-1288;0,794;-0,233;2

-1220;0,905;-0,034;2

-1143;0,473;0,019;2

-1065;0,505;0,1;2

-977;0,815;0,789;2

-884;0,612;0,156;2

-772;0,348;-0,1;2

-644;0,49;0,003;2

-511;0,487;0,025;2

-405;0,693;-0,148;2

-368;0,472;0,042;2

-424;0,19;-0,12;2

-545;0,251;-0,058;2

-686;0,407;0,083;2

-816;0,427;-0,094;2

-919;0,211;1,359;2

-1004;0,186;-0,12;2

-1066;0,262;-0,027;2

-1120;-0,282;0,086;2

-1161;0,1;-0,025;2

-1193;-0,236;0,059;2

-1229;-0,369;0,015;2

-1259;-0,029;-0,002;2

-1291;-0,022;0,21;2

-1319;0,045;-0,124;2

-1346;0,059;0,029;2

-1370;-0,209;0,033;2

-1395;0,064;-0,049;2

-1418;0,1;0,101;2

-1437;-0,086;-0,028;2

-1454;0,061;0,028;2

-1466;0;0,015;2

-1478;0,139;-0,062;2

-1474;0,197;0,152;2

-1457;0,345;0,001;2

-1422;0,698;0,016;2

-1379;0,452;-0,047;2

-1328;0,307;-0,078;2

-1272;0,252;-0;2

-1202;0,193;0,043;2

-1118;0,235;-0,006;2

-1014;-0,107;0,078;2

-897;-0,255;-0,009;2

-778;-0,174;0,076;2

-677;-0,238;0,008;2

-627;-0,054;-0,012;2

-651;-0,233;0,11;2

-740;-0,221;-0,065;2

-853;-0,351;0,077;2

-964;-0,468;0,076;2

-1053;-0,043;-0,022;2

-1124;0,249;0,127;2

-1172;0,347;0,039;2

-1211;0,367;0,024;2

-1243;0,428;0,011;2

-1272;0,467;-0,056;2

-1294;0,552;0,076;2

-1318;0,688;-0,074;2

-1347;0,616;-0,003;2

-1372;0,55;0,047;2

-1402;0,607;-0,079;2

-1425;0,389;0,065;2

-1445;0,436;0,009;2

-1463;0,376;0,01;2

-1473;-0,077;0,084;2

-1485;0,044;-0,065;2

-1491;0,251;0,092;2

-1501;0,082;0,029;2

-1504;0,169;-0,011;2

-1504;0,234;0,014;2

-1491;0,172;-0,062;2

-1466;0,191;0,044;2

-1423;0,007;0,029;2

-1367;0,027;-0,067;2

-1305;0,06;0,061;2

-1239;0,144;-0,006;2

-1166;0,37;0,031;2

-1083;0,176;0,034;2

-992;0,473;0,013;2

-886;0,603;0,115;2

-782;0,219;-0,036;2

-689;0,187;0,05;2

-635;0,338;0,056;2

-633;0,512;-0,072;2

-685;0,36;0,075;2

-771;0,579;-0,089;2

-863;0,617;0,012;2

-945;0,238;0,101;2

-1013;0,13;-0,073;2

-1067;-0,022;0,14;2

-1105;0,129;-0,005;2

-1140;0,583;-0,034;2

-1165;0,265;0,084;2

-1193;0,251;-0,038;2

-1220;0,21;0,037;2

-1251;-0,087;0,002;2

-1278;0,285;-0,07;2

-1303;0,148;0,203;2

-1331;-0,057;-0,082;2

-1347;0,06;0,024;2

-1362;-0,028;0,002;2

-1370;-0,061;-0,076;2

-1373;-0,385;0,104;2

-1381;-0,578;-0,094;2

-1390;-0,189;-0,004;2

-1405;-0,298;0,092;2

-1416;-0,297;-0,094;2

-1436;-0,382;0,019;2

-1448;-0,26;-0,011;2

-1463;-0,02;0,042;2

-1474;-0,133;0,067;2

-1479;0,132;-0,017;2

-1464;0,089;0,078;2

-1430;-0,188;-0;2

-1380;0,242;-0,018;2

-1321;0,23;0,099;2

-1260;0;-0,106;2

-1201;-0,083;0,037;2

-1147;-0,02;0,005;2

-1094;0,305;-0,108;2

-1042;0,467;0,075;2

-981;0,439;-0,027;2

-918;0,263;-0,025;2

-834;0,464;0,049;2

-750;0,565;-0,106;2

-674;0,291;0,082;2

-631;0,277;-0,03;2

-638;0,299;-0,047;2

-690;0,145;0,061;2

-760;0,198;-0,109;2

-842;0,204;0,07;2

-910;0,243;0,016;2

-966;0,29;-0,02;2

-1007;0,045;0,101;2

-1045;-0,003;-0,095;2

-1077;0,36;0,126;2

-1113;0,442;-0,047;2

-1149;0,536;-0,047;2

-1180;0,472;0,09;2

-1211;0,643;-0,095;2

-1234;0,632;0,051;2

-1257;0,349;-0,057;2

-1274;0,47;-0,084;2

-1285;0,692;0,064;2

-1295;0,435;-0,059;2

-1302;0,732;-0,011;2

-1314;0,426;-0,009;2

-1326;-0,009;-0,088;2

-1341;0,129;0,083;2

-1349;-0,009;-0,009;2

-1343;-0,208;0,077;2

-1327;-0,425;0,059;2

-1297;-0,548;-0,083;2

-1259;-0,449;0,09;2

-1214;-0,365;0,036;2

-1159;0,01;-0,042;2

-1092;-0,121;0,056;2

-1020;-0,004;-0,037;2

-939;0,55;0,003;2

-856;0,625;-0,017;2

-765;0,717;-0,06;2

-681;0,58;0,065;2

-619;0,686;-0,081;2

-607;0,791;0,054;2

-639;0,188;-0,007;2

-706;0,151;-0,104;2

-782;0,155;0,083;2

-857;-0,192;-0,062;2

-923;-0,189;-0,013;2

-984;-0,386;0,037;2

-1035;-0,178;-0,157;2

-1079;-0,109;0,045;2

-1110;-0,265;0,005;2

-1132;-0,288;0,014;2

-1154;-0,291;0,099;2

-1170;0,054;-0,054;2

-1187;0,301;0,05;2

-1202;0,296;0,042;2

-1224;0,588;-0,063;2

-1244;0,577;0,037;2

-1270;0,46;-0,036;2

-1296;0,177;0,024;2

-1316;0,207;-0,027;2

-1332;0,222;-0,064;2

-1342;0,248;0,046;2

-1349;0,377;-0,001;2

-1352;-0,127;0,011;2

-1352;-0,435;-0,027;2

-1350;-0,054;-0,074;2

-1346;-0,278;0,093;2

-1348;-0,685;-0,043;2

-1346;-0,502;0,026;2

-1346;-0,645;0,086;2

-1331;-0,315;-0,087;2

-1310;0,042;0,034;2

-1277;-0,446;-0,029;2

-1236;-0,375;0,007;2

-1186;-0,341;0,05;2

-1131;0,355;-0,062;2

-1061;0,813;0,011;2

-984;0,435;-0,023;2

-896;0,523;-0,062;2

-804;0,498;0,083;2

-709;0,399;-0,052;2

-619;0,511;0,064;2

-555;0,52;0,004;2

-534;0,335;-0,08;2

-570;0,105;0,088;2

-641;0,33;-0,004;2

-728;0,36;0,015;2

-811;-0,3;0,074;2

-881;-0,318;-0,071;2

-938;-0,108;0,077;2

-987;0,106;-0,021;2

-1020;0,33;0,024;2

-1052;0,155;0,079;2

-1075;0,126;-0,041;2

-1100;-0,009;0,049;2

-1125;0,273;0,017;2

-1154;0,596;-0,034;2

-1180;0,437;0,08;2

-1199;0,421;-0,036;2

-1218;0,526;0,035;2

-1236;0,54;-0,014;2

-1257;0,398;-0,026;2

-1274;0,205;0,078;2

-1289;0,19;-0,051;2

-1299;0,198;0,039;2

-1302;0,284;0,044;2

-1310;0,264;-0,09;2

-1319;-0,018;0,13;2

-1330;-0,196;-0,033;2

-1333;-0,062;0,01;2

-1333;-0,346;0,079;2

-1318;-0,475;-0,111;2

-1290;-0,208;0,092;2
[truncated: 1,719,541 more chars]
